# Atroposelective Construction of Axially Chiral Tetraarylethenes Via NHC‐Catalyzed Desymmetrization

**DOI:** 10.1002/advs.75488

**Published:** 2026-04-30

**Authors:** Yang‐Ze Zheng, Ting‐Rui Luan, Ming‐Hao Song, Zhaofeng Sun, Chuan‐Jun Lu, Long‐Long Xi, Ren‐Rong Liu

**Affiliations:** ^1^ College of Pharmaceutical Sciences Guizhou University Guiyang Guizhou China; ^2^ College of Chemistry and Chemical Engineering Qingdao University Qingdao China; ^3^ School of Rehabilitation Sciences and Engineering University of Health and Rehabilitation Sciences Qingdao China

**Keywords:** atroposelective synthesis, axially chiral tetraarylethenes, desymmetrization, NHC‐catalyzed, organocatalysis

## Abstract

We report the first NHC‐catalyzed atroposelective synthesis of axially chiral tetraarylethenes (TAEs) via oxidative desymmetrization of prochiral TAE dialdehydes. This catalytic strategy operates under mild conditions and accommodates a broad range of phenolic and nitrogen nucleophiles, delivering diverse axially chiral TAEs in good yields and up to 99% ee. The method features excellent functional‐group tolerance, enables late‐stage modification of complex natural products and pharmaceuticals, and is readily scalable. Furthermore, the resulting axially chiral TAEs display distinct photophysical behavior, pronounced aggregation‐induced emission (AIE), and strong circularly polarized luminescence (CPL). This work establishes a general and efficient platform for constructing functional chiral TAEs with promising optoelectronic potential.

## Graphical abstract

We report the first NHC‐catalyzed atroposelective synthesis of axially chiral TAEs via oxidative desymmetrization of prochiral TAE dialdehydes. This catalytic strategy operates under mild conditions and accommodates a broad range of phenolic and nitrogen nucleophiles, delivering diverse axially chiral TAEs in good yields and up to 99% ee. Furthermore, the resulting axially chiral TAEs display distinct photophysical behavior and pronounced aggregation‐induced emission.

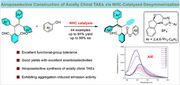

## Introduction

Over the past two decades, organocatalysis has undergone remarkable expansion, establishing itself as a powerful platform for modern synthetic chemistry [1, 2]. Within this field, N‐heterocyclic carbenes (NHCs) have become one of the most versatile and influential classes of Lewis base catalysts (For reviews on NHC catalysis, see ref. [3, 4, 5, 6, 7, 8, 9, 10, 11, 12, 13, 14, 15]). Their distinctive reactivity, centered on the generation of the Breslow intermediate, enables aldehydes to be converted from relatively inert carbonyl compounds into potent nucleophiles or radical progenitors. This fundamental reactivity switch has unlocked a diverse array of transformations, including acylation, oxidative esterification, and previously inaccessible remote C─H functionalizations [16, 17, 18, 19, 20]. More recently, the fusion of NHC catalysis with photo‐ and metalla‐photocatalytic activation modes has further expanded its synthetic scope [21, 22, 23], providing new avenues for constructing structurally complex molecules with high precision.

**SCHEME 1:**
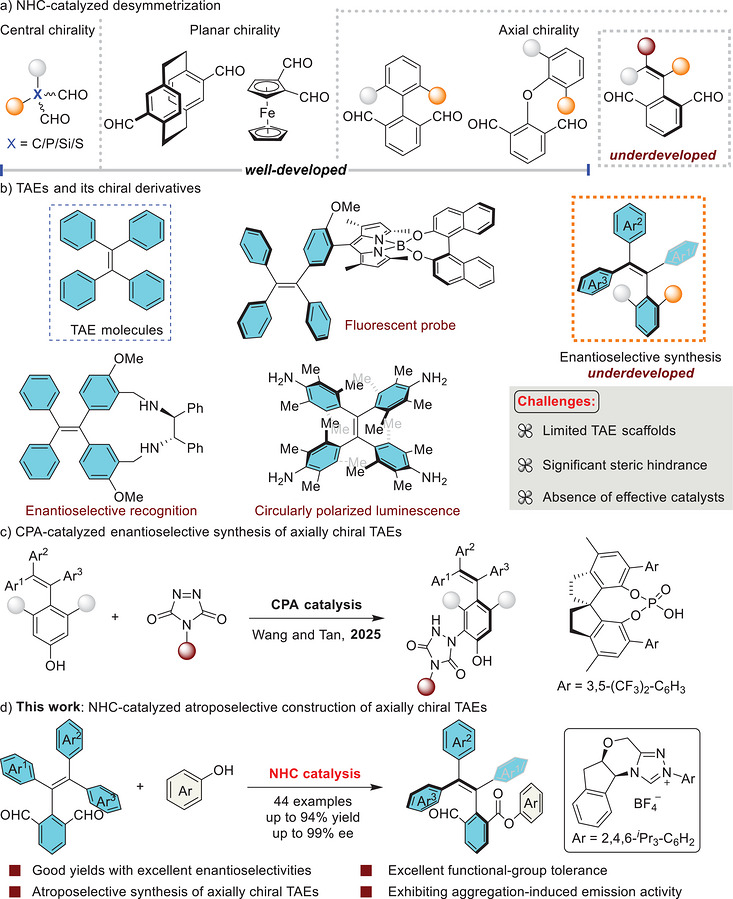
Background introduction and our strategy for synthesizing axially chiral TAEs.

Chiral NHC catalysis has likewise emerged as a robust strategy for asymmetric synthesis, enabling the stereocontrolled formation of central, axial, and planar chirality (For reviews on enantioselective NHC catalysis, see ref. [24, 25, 26]). For example, NHC‐catalyzed desymmetrization and enantioselective oxidative esterification of prochiral aldehydes have proven to be exceptionally effective for generating stereocenters at carbon, phosphorus, silicon, and sulfur (Scheme 1a). (For NHC‐catalyzed enantioselective construction of central chirality, see ref. [27, 28, 29, 30]). More recently, independent studies by Jin, Veselý, and co‐workers have demonstrated elegant NHC‐enabled routes to planar chiral ferrocenes and [2.2]paracyclophanes (For NHC‐catalyzed enantioselective construction of planar chirality, see ref. [31, 32]). In parallel, NHC‐mediated desymmetric esterification has been established as a powerful tool for the atroposelective synthesis of axially chiral biaryls and C─O diaryl ethers (For NHC‐catalyzed enantioselective construction of axial chirality, see ref. [33, 34, 35, 36, 37, 38]). Despite these notable advances, an important gap remains: the application of NHC‐catalyzed desymmetrization to the construction of axially chiral styrenes has not yet been realized.

Tetraarylethenes (TAEs) constitute a versatile class of π‐functional molecules that play key roles in chemical sensing [39, 40], aggregation‐induced emission (AIE) [41, 42, 43, 44, 45, 46], and circularly polarized luminescence (CPL) [47, 48] (Scheme 1b). Early studies mainly focused on introducing chirality through centrally chiral side chains or axially chiral substituents [49, 50, 51, 52], enabling applications in CPL and helical self‐assembly. Later, Zheng and co‐workers achieved helically chiral TAEs by installing 2,6‐dimethyl groups on the aryl rings [53, 54, 55, 56], obtaining systems capable of highly enantioselective molecular recognition. However, these strategies typically depend on chiral precursors or post‐synthetic resolution. As a result, catalytic asymmetric routes to chiral TAEs remain scarce, largely due to the absence of efficient catalysts, significant steric hindrance, and the limited compatibility of TAE frameworks.

In parallel, advances in atroposelective catalysis have provided diverse tactics for constructing axially chiral styrenes [57, 58, 59, 60, 61, 62]. Very recently, the Tan group developed CPA‐catalyzed asymmetric syntheses of axially chiral TAEs (Scheme 1c), which were further applied to chiral liquid‐crystal materials [63]. Despite these developments, new catalytic strategies for TAE atropisomer construction remain highly desirable. Herein, we disclose the first NHC‐catalyzed atroposelective synthesis of axially chiral TAEs via desymmetric esterification of prochiral TAE dialdehydes (Scheme 1d). This reaction operates under mild conditions, accommodates a wide range of substrates, and provides products with excellent enantioselectivity. Moreover, the resulting chiral TAEs exhibit pronounced AIE behavior and strong CPL activity.

### Results and Discussion

To validate the feasibility of our design, we commenced our investigation using TAE dialdehyde **1a** and 2‐naphthol **2a** as model substrates under NHC catalysis with DQ as the oxidant at room temperature (Table 1). Under the initial conditions, **NHC‐1** (10 mol%) as the catalyst, DQ (1.5 equiv.) as the oxidant, and K_2_CO_3_ as the base in toluene at room temperature for 12 h, the desired desymmetrization product **3a** was obtained in 47% yield with 65% ee (entry 1). This preliminary result indicated that our proposed desymmetrization strategy was indeed viable for inducing atroposelective axial chirality in TAE scaffolds. We next examined the effect of different bases (entries 2–4) and found that replacing K_2_CO_3_ with Cs_2_CO_3_ led to a remarkable enhancement in enantioselectivity, affording **3a** with 87% ee (entry 4). Encouraged by this improvement, we proceeded to explore a range of NHC pre‐catalysts (**NHC‐1** to **NHC‐8**) with varying steric and electronic properties (entries 5–11). To our delight, **NHC‐5** emerged as the optimal catalyst, delivering the desired product **3a** in good yield and excellent enantioselectivity (57% yield, 99% ee, entry 8). Subsequent optimization of the solvent revealed that MTBE provided superior results compared to toluene, Et_2_O, and 1,4‐dioxane, affording **3a** in 68% yield with 99% ee (entries 12–14). Finally, extending the reaction time to 48 h further improved the yield to 76%, while maintaining outstanding enantioselectivity (entry 15), no diesterification product was observed under the standard reaction conditions. These results clearly demonstrate that the developed NHC‐catalyzed oxidative desymmetrization is highly efficient and capable of providing excellent stereochemical control.

**TABLE 1:** Optimization of the reaction conditions.

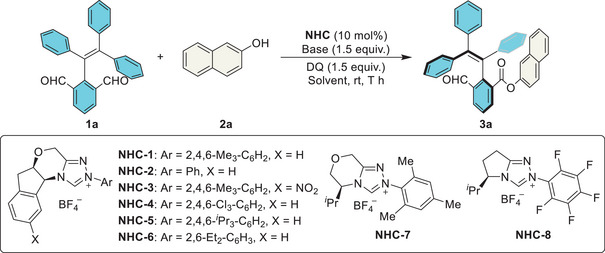						
Entry	NHC	Base	Solvent	Time (h)	Yield of 3a (%)^a^	Ee of 3a (%)^b^
1	**NHC‐1**	K_2_CO_3_	Toluene	12	47	65
2	**NHC‐1**	KOAc	Toluene	12	10	79
3	**NHC‐1**	KOH	toluene	12	63	83
4	**NHC‐1**	Cs_2_CO_3_	toluene	12	52	87
5	**NHC‐2**	Cs_2_CO_3_	toluene	12	15	15
6	**NHC‐3**	Cs_2_CO_3_	toluene	12	21	77
7	**NHC‐4**	Cs_2_CO_3_	toluene	12	20	53
8	**NHC‐5**	Cs_2_CO_3_	toluene	12	57	99
9	**NHC‐6**	Cs_2_CO_3_	toluene	12	39	75
10	**NHC‐7**	Cs_2_CO_3_	toluene	12	32	85
11	**NHC‐8**	Cs_2_CO_3_	toluene	12	Nr	—
12	**NHC‐5**	Cs_2_CO_3_	Et_2_O	12	53	89
13	**NHC‐5**	Cs_2_CO_3_	1,4‐dioxane	12	47	93
14	**NHC‐5**	Cs_2_CO_3_	MTBE	12	68	99
15	**NHC‐5**	Cs_2_CO_3_	MTBE	48	80(76)^c^	99

Unless otherwise specified, the reaction conditions were as follows: **1a** (0.10 mmol), **2a** (0.15 mmol), 10 mol% **NHC**, base (0.15 mmol), and DQ (1.5 equiv.), in 1.5 mL of solvent at rt for 12–48 h under nitrogen.

^a^ Determined by ^1^H‐NMR analysis.

^b^ Determined by chiral HPLC analysis.

^c^ Isolated yield in the parentheses.

**SCHEME 2:**
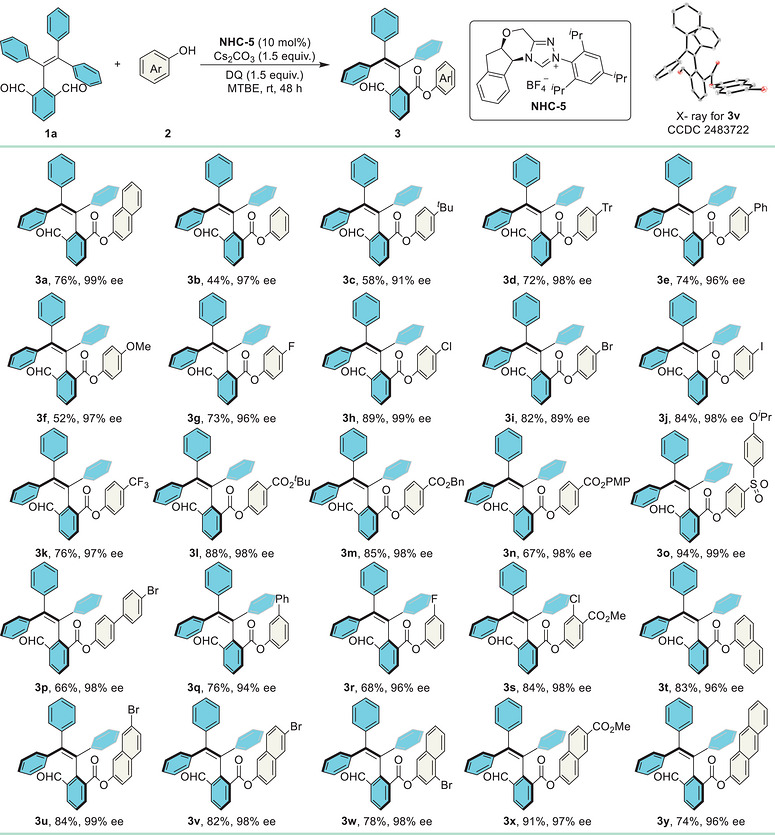
Reaction scope of aromatic alcohols. Reaction conditions: **1a** (0.10 mmol), **2** (0.15 mmol), 10 mol% **NHC‐5**, Cs_2_CO_3_ (0.15 mmol), and DQ (1.5 equiv.), in 1.5 mL of MTBE at rt for 48 h under nitrogen; isolated yield by silica gel chromatography; ee values were determined by chiral HPLC.

With the optimal catalyst **NHC‐5** in hand, we next examined the substrate scope of the NHC‐catalyzed enantioselective construction of axially chiral TAEs (Scheme 2). A wide range of aromatic substrates bearing diverse substituents were well tolerated, affording the corresponding products in good yields and with excellent enantioselectivities. For instance, both 2‐naphthol and phenol performed smoothly, delivering products **3a** and **3b** in 44–76% yield and 97–99% ee. *Para*‐substituted substrates bearing electron‐donating groups (*^t^* Bu, Tr, Ph, OMe) also reacted efficiently to give **3c**‐**3f** in good yields and with outstanding enantioselectivities (up to 98% ee). In addition, substrates containing electron‐withdrawing substituents (F, Cl, Br, I, CF_3_, CO_2_R, SO_2_R) were equally compatible, affording the desired axially chiral TAEs **3g**‐**3p** in 66–94% yields and 89–99% ee. Moreover, substrates bearing *meta*‐substituents or disubstituted patterns were suitable, providing **3q**‐**3s** in good to excellent yields with consistently high stereocontrol. Notably, 1‐naphthol participated well in the reaction, furnishing the chiral TAE **3t** in 83% yield and 96% ee. Gratifyingly, 2‐naphthols bearing substituents at different positions as well as 2‐hydroxyanthracene, also delivered the desired products **3u**‐**3y**, further demonstrating the broad applicability and robustness of this catalytic system. The absolute configuration of the axially chiral TAE was unambiguously assigned by single‐crystal X‐ray diffraction analysis of **3v**. Meanwhile, aliphatic alcohols such as MeOH, EtOH, and BnOH were examined under the optimized conditions, but no desired products were detected.

**SCHEME 3:**
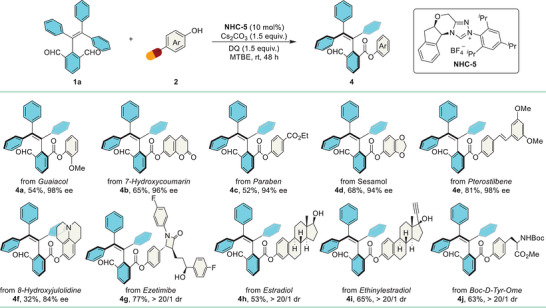
Reaction scope of natural or bioactive phenols. Reaction conditions: 1a (0.10 mmol), 2 (0.15 mmol), 10 mol% NHC‐5, Cs_2_CO_3_ (0.15 mmol), and DQ (1.5 equiv.), in 1.5 mL of MTBE at rt for 48 h under nitrogen; isolated yield by silica gel chromatography; ee values were determined by chiral HPLC; dr values were determined by ^1^H NMR.

To further underscore the wide functional‐group compatibility and the extensive substrate adaptability of this method, we applied the catalytic system to a collection of derivatives originating from structurally intricate natural products and pharmacologically relevant molecules (Scheme 3). A diverse set of substrates, including guaiacol, 7‐hydroxycoumarin, paraben, sesamol, pterostilbene, 8‐hydroxyjulolidine, ezetimibe, estradiol, ethinylestradiol, and Boc‐*D*‐Tyl‐OMe, proved fully amenable to the reaction conditions, delivering the corresponding chiral products **4a**‐**4j** in consistently good yields and excellent stereochemical outcomes (84–98% ee, > 20:1 dr).

**SCHEME 4:**
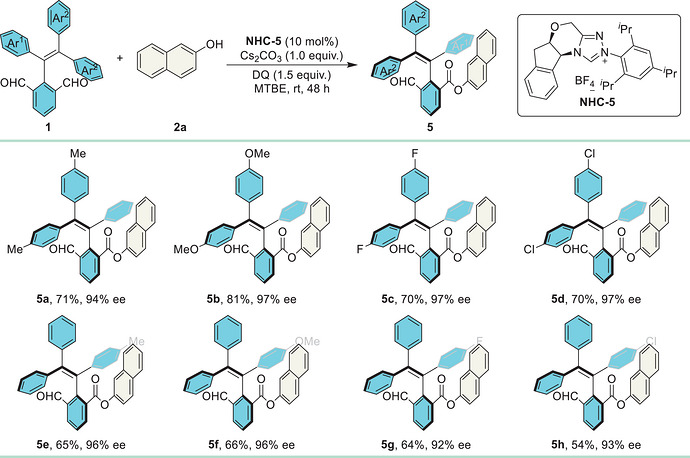
Reaction scope of prochiral TAE structures. Reaction conditions: **1** (0.10 mmol), **2a** (0.15 mmol), 10 mol% **NHC‐5**, Cs_2_CO_3_ (0.15 mmol), and DQ (1.5 equiv.), in 1.5 mL of MTBE at rt for 48 h under nitrogen; isolated yield by silica gel chromatography; ee values were determined by chiral HPLC.

Next, we expanded our investigation of the substrate scope by examining a series of TAE dialdehydes **1** incorporating diverse aryl substituents, using 2‐naphthol **2a** as the model substrate (Scheme 4). We first assessed substrates in which the aryl ring positioned distal to the benzaldehyde unit carries *para*‐substituents. Functional groups such as methyl, methoxy, and halogens (F, Cl) were readily accommodated under the reaction conditions, furnishing products **5a**‐**5d** in 70%–81% yields with high enantioinduction (94%–97% ee). We then turned our attention to TAE frameworks bearing *para*‐substituents on the aryl ring directly bonded to the same carbon. These variants also performed smoothly, delivering axially chiral TAEs **5e**‐**5 h** in 54%–66% yields while maintaining excellent stereocontrol (92%–96% ee).

**SCHEME 5:**
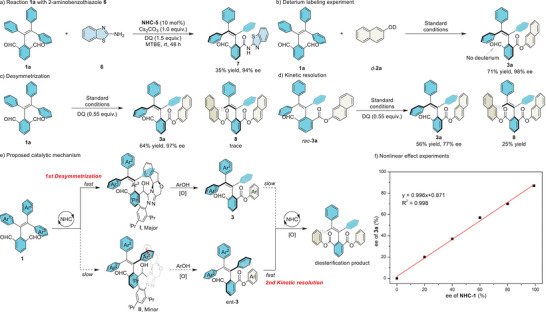
Reaction of 2‐aminobenzothiazole and mechanistic studies.

In addition to employing phenols as nucleophiles, we further assessed the compatibility of nitrogen‐based nucleophiles (Scheme 5a). Under the optimized conditions, 2‐aminobenzothiazole **6** participated in the reaction to deliver axially chiral TAE **7** in 35% yield with excellent enantioselectivity (94%). We further explored the transformation of dialdehyde **1a** into axially chiral benzonitrile derivatives through an atroposelective imine umpolung approach [64, 65]. Unfortunately, the reaction consistently stalled at the imine intermediate and did not proceed to form the desired benzonitrile products. To further elucidate the mechanism of the NHC‐catalyzed desymmetrization, a set of control experiments was carried out [33, 34, 35, 66]. When deuterated *d*‐**2a** was subjected to the standard reaction conditions (Scheme 5b), no deuterium incorporation was detected in the formyl group of **3a**, indicating that the formation of the Breslow intermediate is essentially irreversible under these conditions. Conducting the reaction between **1a** and **2a** with only 0.55 equivalents of DQ (Scheme 5c) provided **3a** in 64% yield and 97% ee, along with a trace amount of the diesterification product **8**. In contrast, treating *rac*‐**3a** with **2a** under the same 0.55 equivalents of DQ conditions (Scheme 5d) furnished enantioenriched **3a** in 56% yield with 77% ee, and product **8** was obtained in 25% yield. These combined results support a mechanistic model in which the initial NHC‐mediated desymmetrization of the prochiral dialdehyde is the primary stereo‐controlling step, followed by a secondary kinetic‐resolution process that further enhances the enantioselectivity. Control experiments suggest that the introduction of the chiral NHC catalyst leads to the reversible formation of two diastereomeric Breslow intermediates (**I** and **II**) (Scheme 5e). Owing to steric differentiation, intermediate **I** is preferentially generated and is readily oxidized under mild conditions, whereas formation of **II** is disfavored. The resulting activated species subsequently reacts with aromatic alcohol **2** as the acyl‐accepting partner to furnish product **3**, accompanied by the release of the free NHC catalyst. Enantioselectivity is further amplified through a secondary kinetic resolution process, in which the less favored enantiomer (ent‐**3**) undergoes an additional stereoselective esterification. Moreover, the observed linear correlation between the ee of **NHC‐1** and that of product **3a** supports the involvement of a single NHC unit in the enantio‐determining esterification step (Scheme 1f; Figures S1; Table S1).

**FIGURE 1:**
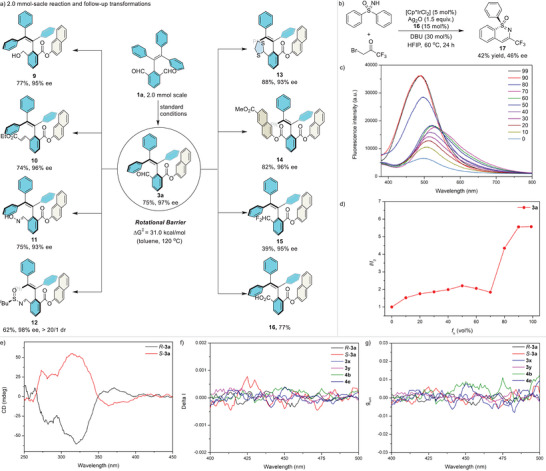
Synthetic transformations and optical property investigations. (a) Synthetic transformations. (b) Practical application of **16** as a ligand for asymmetric reaction. (c) The PL intensity of **3a** in THF/H_2_O mixed solvents increased with an increased H_2_O volume fraction (*f*_h_) to 99% (c = 10 *µ*M, *λ*_ex_ = 320 nm). (d) Relative PL intensity of **3a**. (e) CD spectra of *R*‐**3a** and *S*‐**3a** in THF (1.0 × 10^−3^ m) at room temperature. (f) CPL spectra of *R*‐**3a**, *S*‐**3a**, **3x**, **3y**, **4b**, and **4e** in THF (1.0 × 10^−3^ m) at room temperature. (g) g_lum_ values−wavelength curve for *R*‐**3a**, *S*‐**3a**, **3x**, **3y**, **4b**, and **4e**.

To highlight the synthetic practicality of this protocol, a 2.0 mmol‐scale preparation of **3a** was conducted (Figure 1a). The reaction proceeded smoothly to furnish **3a** in 75% yield with a slight diminution in enantioselectivity, the rotational barrier of **3a** was experimentally determined to be 31.0 kcal/mol in toluene at 120°C. We next explored downstream functionalization of the axially chiral product **3a**. Reduction of the aldehyde group with NaBH_4_ produced chiral benzyl alcohol **9** in 77% yield with 95% ee. A Wittig olefination also proved efficient, affording alkene **10** in 74% yield with high geometric and enantiomeric purity (> 20:1 E/Z, 96% ee). Condensation of **3a** with hydroxylamine hydrochloride or (*R*)‐tert‐butylsulfinamide afforded oxime **11** and sulfinamide **12**, respectively, in up to 75% yield and up to 98% ee. Reaction with 1,3‐ethanedithiol produced thioacetal **13** in good yield, though with a slight decrease in enantioselectivity. Esterification of **3a** under *rac*‐NHC catalysis afforded **14** in 82% yield while preserving its optical purity. In addition, aldehyde **3a** was subjected to deoxyfluorination, delivering the fluorinated product **15** in 39% yield without any detectable erosion of enantioselectivity. Subsequent oxidation of the aldehyde functionality proceeded efficiently to give the corresponding carboxylic acid **16** in 77% yield. Notably, the axially chiral TAE carboxylic acid **16** was successfully applied as a chiral ligand in an Ir(III)‐catalyzed asymmetric C─H activation/desymmetrization of sulfoximines (Figure 1b), affording the sulfur‐stereogenic sulfoximine **17** in 42% yield with 46% ee.

Moreover, the AIE behavior of the newly developed axially chiral TAE framework was examined (Figures 1c, d). Compound **3a** displayed only weak fluorescence in THF/H_2_O mixtures when the water fraction (*f*_h_) was below 70% (Figure S2). However, the emission intensity increased progressively with higher *f*_h_ values. Once *f*_h_ > 80%, a pronounced fluorescence band emerged at 490 nm, clearly demonstrating the strong AIE characteristics of these chiral TAE derivatives (Figure S3). A detailed study of the photophysical behavior of representative axially chiral TAEs **3a**, **3x**, **3y**, **4b**, and **4e** was conducted. The enantiomeric pair *R*‐**3a** and *S*‐**3a** showed opposite Cotton effects in their CD spectra (Figure 1e; Figure S4), validating the preservation of enantiopurity and confirming their distinct chiroptical signatures. CPL analysis further revealed well‐defined circularly polarized emission for *R*‐**3a**, *S*‐**3a**, **3x**, **3y**, **4b**, and **4e** (Figure 1f; Figure S5). The corresponding |g_lum_| values, extracted from their CPL spectra (Figure 1g; Figure S6), illustrate meaningful differences in chiroptical performance. Notably, compound **3a** exhibited the largest dissymmetry factor (|g_lum_| = 1.67 × 10^−3^), highlighting its strong potential for use in CPL‐active optoelectronic materials.

### Conclusions

In summary, we have developed an efficient NHC‐catalyzed oxidative desymmetrization strategy for constructing axially chiral TAEs with broad substrate scope, high yields, and excellent enantioselectivity. This method enables late‐stage functionalization, scales smoothly, and delivers chiral scaffolds with distinct photophysical and chiroptical properties, highlighting their potential in advanced optoelectronic applications.

## Conflicts of Interest

The authors declare no conflicts of interest.

## Supporting information




**Supporting file**: advs75488‐sup‐0001‐SuppMat.pdf
