# Supplementary material for: Atroposelective Construction of Axially Chiral Tetraarylethenes Via NHC‐Catalyzed Desymmetrization
Source: Adv Sci (Weinh). 2026 Apr 30;13(41):e75488. doi: 10.1002/advs.75488 (PMC13335694; doi:10.1002/advs.75488)
Supplement: Supplementary file 1 — Supporting file: advs75488‐sup‐0001‐SuppMat.pdf [file ADVS-13-e75488-s001.pdf]

Supporting Information

Atroposelective Construction of Axially Chiral Tetraarylethenes via NHC-Catalyzed Desymmetrization

Yang-Ze Zheng,[a],[b] Ting-Rui Luan,[b] Ming-Hao Song,[b] Zhaofeng Sun,[c] Chuan-Jun Lu,[b]* Long-Long Xi,[b]* Ren-Rong Liu[a],[b]*

[a] Yang-Ze Zheng, Prof. Dr. Ren-Rong Liu
College of Pharmaceutical Sciences, Guizhou University, Guiyang, Guizhou 550025, China
[b] Yang-Ze Zheng, Ting-Rui Luan, Ming-Hao Song, Dr. Chuan-Jun Lu, Prof. Dr. Long-Long Xi, Prof. Dr. Ren-Rong Liu
College of Chemistry and Chemical Engineering, Qingdao University, Qingdao 266071, China
[c] Dr. Zhaofeng Sun
School of Rehabilitation Sciences and Engineering, University of Health and Rehabilitation Sciences, Qingdao 266113, China
E-mail: chuanjunlu@qdu.edu.cn; xill@qdu.edu.cn; renrongliu@qdu.edu.cn

Table of Contents

1. General Information 1

2. Synthesis of Substrates 2

3. Experimental Procedures and Characterization of Products. 8

4. Reaction of 2-Aminobenzothiazole and Control Experiments 35

5. Synthetic Transformations 41

6. Copies of Optical Spectra 50

7. Copies of ^{1}H NMR, ^{13}C NMR and ^{19}F NMR 53

8. Copies of HPLC Spectra 120

9. Crystallographic Data 167

10. Reference 178

1. General Information

Unless stated otherwise, all reagents were purchased from commercial sources and used without further purification. Solvents were dried and distilled before use by standard procedures. Reactions were monitored by thin layer chromatography (TLC) using silica gel plates. Flash column chromatography was performed over silica gel (200-300 mesh). NMR spectra were recorded on a Bruker Avance operating at for ^{1}H NMR at 400 MHz or 600 MHz, ^{13}C NMR at 101 MHz or 151 MHz, ^{19}F NMR at 565 MHz or 376 MHz, and chemical shifts (δ) are reported in ppm relative to those of residual solvent signals: $CDCl_3$ (^{1}H NMR δ 7.26, ^{13}C NMR δ 77.00). $(CD_3)_2SO$ (^{1}H NMR δ 2.50, ^{13}C NMR δ 39.50). All coupling constants (*J*) are reported in Hz. The following abbreviations were used to describe peak splitting patterns when appropriate: s = singlet, d = doublet, t = triplet, q = quartet, m = multiplet, br = broad. HRMS were recorded on Waters Xevo G2-XS QT of mass spectrometer. Optical properties were recorded in analytical grade solvent (THF). UV-vis absorption spectra were recorded using a Metrohm AUTOLAB UV-visible spectrophotometer at room temperature. Fluorescence spectra were recorded using an Metrohm AUTOLAB fluorescence spectrophotometer. Circular dichroism (CD) spectra were measured on a JASCO Corporation J-1500 spectrophotometer. The circularly polarized luminescence spectra were measured on a JASCO Corporation CPL-300 spectrophotometer. The enantiomeric excesses of the products were determined by HPLC analysis on Shimadzu LC-20AT, using Chiralpak AD-H (4.6 mm Φ × 250 mmL), IA (4.6 mm Φ × 250 mmL), OD-H (4.6 mm Φ × 250 mmL), IC (4.6 mm Φ × 250 mmL) AS-H (4.6 mm Φ × 250 mmL) columns purchased from Daicel Chemical Industries. Unless otherwise noted, materials obtained from commercial suppliers were used without further purification.

2. Synthesis of Substrates

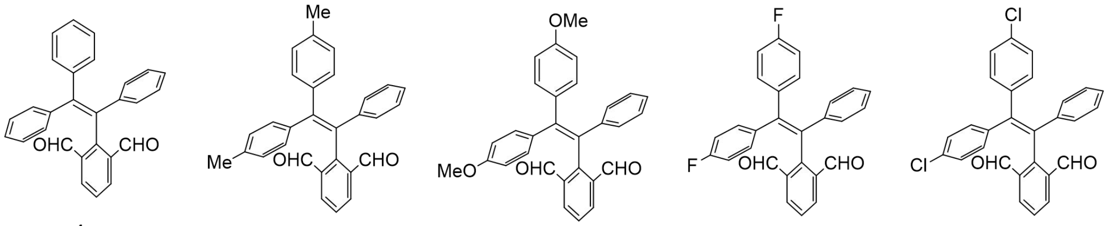

1a 1b 1c 1d 1e

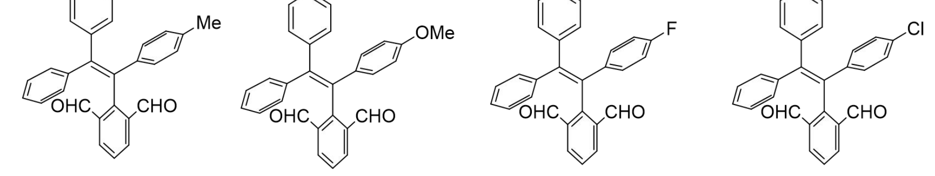

1f 1g 1h 1i

Procedure A:

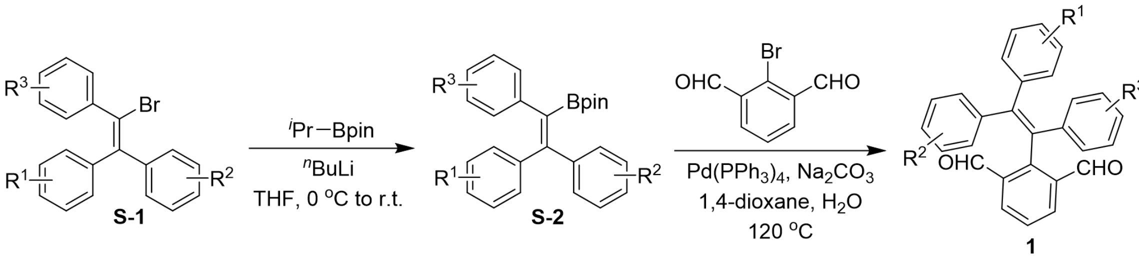

1

Under a nitrogen atmosphere, THF was added to a round-bottomed flask containing **S-1**[1] (8 mmol). The mixture was cooled to -78 °C, then ^{n}BuLi (1.1 equiv.) was added followed by 2-Isopropoxy-4,4,5,5-tetramethyl-1,3,2-dioxaborolane (1.2 equiv.). The mixture was stirred for 20 minutes. The temperature was raised to room temperature and H_2O (40 mL) was added. The mixture was stirred for 15 minutes, the aqueous phase was extracted with ethyl acetate (20 mL × 3) and the combined organic layer was washed with brine (10 mL × 3), then dried over Na_2SO_4. The solvent was evaporated and the residue was purified by silica gel column chromatography. Under a nitrogen atmosphere, 1,4-dioxane was added to a pressure-resistant bottle containing **S-2** (1.5 equiv.), 2-Bromoisophthalaldehyde (1 equiv.) and $Pd(PPh_3)_4$ (0.05 equiv.), followed by the addition of a sodium carbonate aqueous solution. The mixture was stirred at 120 °C for 24 h, The aqueous phase was extracted with ethyl acetate (20 mL × 3) and the combined organic layer was washed with brine (10 mL × 3), then dried over Na_2SO_4. The solvent was evaporated and the residue was purified by silica gel column chromatography to give the corresponding product **1**.

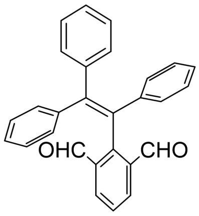

1a

2-(1,2,2-triphenylvinyl)isophthalaldehyde (1a) was synthesized by following Procedure A. The crude material was purified by column chromatography to provide **1a** as a yellow solid (338 mg, 87% yield).

^{1}H NMR (600 MHz, $CDCl_3$) δ 10.47 (s, 2H), 8.04 (d, *J* = 7.7 Hz, 2H), 7.47 – 7.44 (m, 1H), 7.22 – 7.18 (m, 3H), 7.14 – 7.11 (m, 5H), 7.05 – 7.03 (m, 3H), 7.00 – 6.98 (m, 2H), 6.91 – 6.88 (m, 2H).

^{13}C NMR (151 MHz, $CDCl_3$) δ 191.0, 148.7, 146.7, 142.0, 141.6, 141.4, 134.9, 133.4, 131.5, 130.8, 130.4, 129.9, 128.3, 128.2, 128.1, 127.6, 127.4.

HRMS: (ESI) m/z: $[M+H]^+$ Calcd for $C_{28}H_{21}O_2^+$ 389.1536; Found 389.1529.

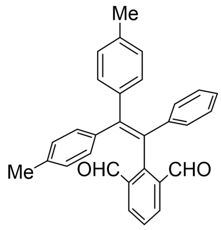

1b

2-(1-phenyl-2,2-di-p-tolylvinyl)isophthalaldehyde (1b) was synthesized by following Procedure A. The crude material was purified by column chromatography to provide **1b** as a yellow solid (291 mg, 70% yield).

^{1}H NMR (600 MHz, $CDCl_3$) δ 10.45 (s, 2H), 8.04 (d, *J* = 7.7 Hz, 2H), 7.45 (m, 1H), 7.11 (m, 3H), 6.99 – 6.96 (m, 6H), 6.84 – 6.82 (m, 2H), 6.77 – 6.75 (m, 2H), 2.31 (s, 3H), 2.17 (s, 3H).

^{13}C NMR (151 MHz, $CDCl_3$) δ 191.2, 149.4, 147.0, 142.2, 139.2, 138.7, 137.5, 137.2, 134.9, 133.3, 130.8, 130.4, 130.3, 129.9, 128.8, 128.3, 128.0, 127.2, 21.2, 21.1.

HRMS: (ESI) m/z: $[M+H]^+$ Calcd for $C_{30}H_{25}O_2^+$ 417.1849; Found 417.1841.

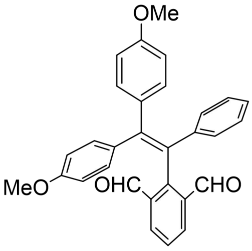

1c

2-(2,2-bis(4-methoxyphenyl)-1-phenylvinyl)isophthalaldehyde (1c) was synthesized by following Procedure A. The crude material was purified by column chromatography to provide **1c** as a yellow solid (360 mg, 80% yield).

^{1}H NMR (600 MHz, $CDCl_3$) δ 10.42 (s, 2H), 8.03 (d, *J* = 7.7 Hz, 2H), 7.44 (t, *J* = 7.7 Hz, 1H), 7.12 – 7.08 (m, 3H), 7.03 – 7.01 (m, 2H), 6.98 – 6.96 (m, 2H), 6.79 (d, *J* = 8.7 Hz, 2H), 6.72 (d, *J* = 8.7 Hz, 2H), 6.56 (d, *J* = 8.7 Hz, 2H), 3.77 (s, 3H), 3.67 (s, 3H).

^{13}C NMR (151 MHz, $CDCl_3$) δ 191.2, 191.0, 159.0, 158.6, 149.6, 146.4, 142.5, 135.0, 134.6, 134.5, 134.0, 133.3, 132.3, 131.6, 130.4, 129.4, 128.4, 127.9, 127.0, 113.49, 113.47, 55.1, 55.0.

HRMS: (ESI) m/z: $[M+H]^+$ Calcd for $C_{30}H_{25}O_4^+$ 449.1747; Found 449.1741.

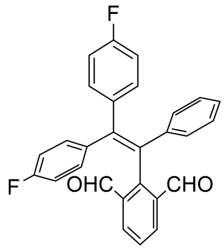

1d

2-(2,2-bis(4-fluorophenyl)-1-phenylvinyl)isophthalaldehyde (1d) was synthesized by following Procedure A. The crude material was purified by column chromatography to provide **1d** as a yellow solid (373 mg, 88% yield).

^{1}H NMR (600 MHz, $CDCl_3$) δ 10.38 (s, 2H), 8.04 (d, *J* = 7.7 Hz, 2H), 7.51 – 7.48 (m, 1H), 7.14 – 7.12 (m, 3H), 7.09 – 7.06 (m, 2H), 6.98 – 6.95 (m, 2H), 6.91 – 6.88 (m, 2H), 6.86 – 6.83 (m, 2H), 6.76 – 6.72 (m, 2H).

^{13}C NMR (151 MHz, $CDCl_3$) δ 190.8, 162.1 (d, *J* = 248.7 Hz), 161.7 (d, *J* = 248.8 Hz), 148.0, 144.2, 141.2, 137.9 (d, *J* = 3.5 Hz), 137.3 (d, *J* = 3.6 Hz), 134.9, 133.9, 132.6 (d, *J* = 8.2 Hz), 132.3, 131.6 (d, *J* = 8.0 Hz), 130.3, 128.4, 128.4, 127.6, 115.4 (d, *J* = 21.7 Hz), 115.3 (d, *J* = 21.6 Hz).

^{19}F NMR (565 MHz, $CDCl_3$) δ -112.85, -112.92.

HRMS: (ESI) m/z: $[M+H]^+$ Calcd for $C_{28}H_{19}F_2O_2^+$ 425.1348; Found 425.1338.

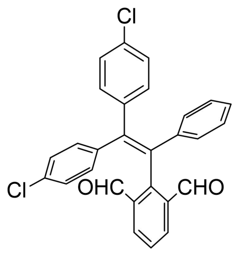

1e

2-(2,2-bis(4-chlorophenyl)-1-phenylvinyl)isophthalaldehyde (1e) was synthesized by following Procedure A. The crude material was purified by column chromatography to provide **1e** as a yellow solid (319 mg, 70% yield).

^{1}H NMR (600 MHz, $CDCl_3$) δ 10.36 (s, 2H), 8.04 (d, J = 7.7 Hz, 2H), 7.52 (t, J = 7.7 Hz, 1H), 7.18 – 7.16 (m, 2H), 7.14 (dd, J = 5.0, 1.9 Hz, 3H), 7.04 – 7.01 (m, 4H), 6.98 – 6.95 (m, 2H), 6.79 (d, J = 8.5 Hz, 2H).

^{13}C NMR (151 MHz, $CDCl_3$) δ 190.7, 147.6, 143.7, 140.9, 140.2, 139.6, 134.9, 134.1, 133.8, 133.6, 133.1, 132.20, 132.17, 131.2, 130.3, 128.6, 128.53, 128.48, 127.8.

HRMS: (ESI) m/z: $[M+H]^+$ Calcd for $C_{28}H_{19}Cl_2O_2^+$ 457.0757; Found 457.0749.

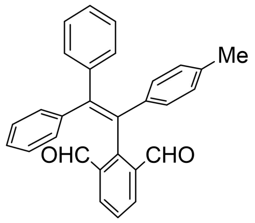

1f

2-(2,2-diphenyl-1-(p-tolyl)vinyl)isophthalaldehyde (1f) was synthesized by following Procedure A. The crude material was purified by column chromatography to provide **1f** as a yellow solid (251 mg, 65% yield).

^{1}H NMR (600 MHz, $CDCl_3$) δ 10.45 (s, 2H), 8.03 (d, J = 7.7 Hz, 2H), 7.46 – 7.43 (m, 1H), 7.23 – 7.19 (m, 3H), 7.15 – 7.12 (m, 2H), 7.04 – 7.02 (m, 3H), 6.93 – 6.91 (m, 2H), 6.89 – 6.85 (m, 4H), 2.24 (s, 3H).

^{13}C NMR (151 MHz, $CDCl_3$) δ 191.1, 149.0, 146.2, 142.1, 141.7, 138.9, 137.4, 135.0, 133.2, 131.4, 130.8, 130.2, 129.9, 129.0, 128.2, 128.12, 128.06, 127.5, 127.3, 21.1.

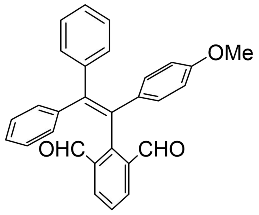

1g

2-(1-(4-methoxyphenyl)-2,2-diphenylvinyl)isophthalaldehyde (1g) was synthesized by following Procedure A. The crude material was purified by column chromatography to provide **1g** as a yellow solid (350 mg, 78% yield).

^{1}H NMR (400 MHz, $(CD_3)_2SO$) δ 10.38 (s, 2H), 7.96 (d, *J* = 7.7 Hz, 2H), 7.56 (t, *J* = 7.7 Hz, 1H), 7.26 – 7.21 (m, 3H), 7.16 (dd, *J* = 7.5, 2.03 Hz, 2H), 7.05 (d, *J* = 7.4 Hz, 3H), 6.92 – 6.86 (m, 4H), 6.71 – 6.67 (m, 2H), 3.65 (s, 3H).

^{13}C NMR (101 MHz, $(CD_3)_2SO$) δ 191.4, 158.1, 147.9, 144.4, 142.3, 141.9, 134.7, 134.1, 133.3, 131.7, 131.4, 130.5, 129.5, 128.5, 128.2, 128.1, 127.3, 127.1, 113.5, 55.0.

HRMS: (ESI) m/z: $[M+H]^+$ Calcd for $C_{29}H_{23}O_3^+$ 419.1642; Found 419.1636.

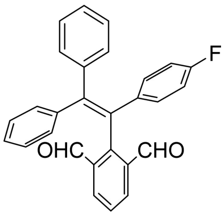

1h

2-(1-(4-fluorophenyl)-2,2-diphenylvinyl)isophthalaldehyde (1h) was synthesized by following Procedure A. The crude material was purified by column chromatography to provide **1h** as a yellow solid (333 mg, 82% yield).

^{1}H NMR (600 MHz, $CDCl_3$) δ 10.42 (s, 2H), 8.03 (d, *J* = 7.7 Hz, 2H), 7.49 – 7.46 (m, 1H), 7.24 – 7.20 (m, 3H), 7.13 – 7.10 (m, 2H), 7.04 (dd, *J* = 4.9, 1.8 Hz, 3H), 6.97 – 6.94 (m, 2H), 6.89 – 6.86 (m, 2H), 6.83 – 6.79 (m, 2H).

^{13}C NMR (151 MHz, $CDCl_3$) δ 190.8, 161.6 (d, *J* = 249.1 Hz), 148.2, 146.7, 141.9, 141.3, 137.7 (d, *J* = 3.5 Hz), 134.9, 133.8, 132.2 (d, *J* = 8.0 Hz), 130.8, 130.5, 129.8, 128.3, 128.3, 128.2, 127.8, 127.5, 115.4 (d, *J* = 21.6 Hz).

^{19}F NMR (565 MHz, $CDCl_3$) δ -113.12.

HRMS: (ESI) m/z: $[M+H]^+$ Calcd for $C_{28}H_{20}FO_2^+$ 407.1442; Found 407.1436.

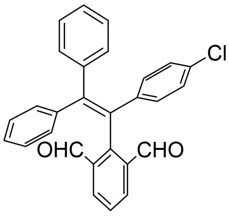

1i

2-(1-(4-chlorophenyl)-2,2-diphenylvinyl)isophthalaldehyde (1i) was synthesized by following Procedure A. The crude material was purified by column chromatography to provide **1i** as a yellow solid (308 mg, 73% yield).

^{1}H NMR (600 MHz, $CDCl_3$) δ 10.43 (m, 2H), 8.04 (dd, *J* = 7.7, 2.3 Hz, 2H), 7.50 – 7.44 (m, 1H), 7.24 – 7.20 (m, 3H), 7.13 – 7.11 (m, 3H), 7.09 – 7.08 (m, 1H), 7.05 – 7.03 (m, 3H), 7.01 – 6.94 (m, 1H), 6.92 – 6.87 (m, 3H).

^{13}C NMR (151 MHz, $CDCl_3$) δ 191.0, 190.7, 147.9, 147.0, 141.8, 141.1, 140.0, 135.0, 133.9, 133.4, 133.3, 131.7, 130.83, 130.76, 130.41, 130.38, 129.9, 129.8, 128.5, 128.4, 128.3, 128.18, 128.15, 127.9, 127.7, 127.6, 127.4.

HRMS: (ESI) m/z: $[M+H]^+$ Calcd for $C_{28}H_{20}ClO_2^+$ 423.1146; Found 423.1139.

3. Experimental Procedures and Characterization of Products.

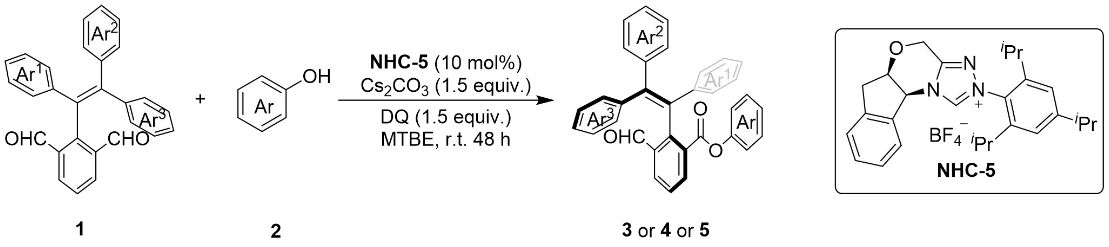

Procedure B:

TAE dialdehyde **1** (0.1 mmol), aromatic alcohol **2** (0.15 mmol, 1.5 equiv.), **NHC-5** (5.0 mg, 0.01 mmol, 10 mol%), Cs_2CO_3 (48.8 mg, 0.15 mmol, 1.5 equiv.), and DQ (57.2 mg, 0.15 mmol, 1.5 equiv. were placed in the reaction tube under the N_2 atmosphere, and anhydrous MTBE (1.5 mL) was added to the reaction mixture via a syringe, and stir at room temperature for 48 h. After the reaction is complete, the crude mixture is purified by column chromatography on silica gel to obtain the corresponding product **3** or **4** or **5**.

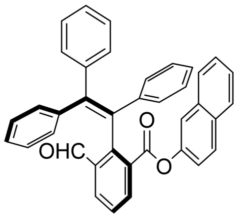

(*R*)-naphthalen-2-yl 3-formyl-2-(1,2,2-triphenylvinyl)benzoate (3a) was synthesized by following Procedure B. The crude material was purified by column chromatography (SiO_2, Toluene) to provide **3a** as a yellow solid (40.3 mg, 76% yield).

^{1}H NMR (400 MHz, $CDCl_3$) δ 10.56 (s, 1H), 8.21 (d, *J* = 7.6 Hz, 1H), 8.01 (d, *J* = 7.8 Hz, 1H), 7.83 (m, 2H), 7.79 – 7.75 (m, 1H), 7.49 m, 3H), 7.32 (d, *J* = 2.4 Hz, 1H), 7.14 (m, 9H), 7.12 – 7.08 (m, 4H), 7.04 – 7.00 (m, 2H), 6.97 (d, *J* = 8.9 Hz, 1H).

^{13}C NMR (101 MHz, $CDCl_3$) δ 191.5, 165.2, 148.1, 147.9, 144.5, 142.7, 142.1, 141.4, 135.4, 135.1, 133.6, 133.3, 132.4, 131.5, 131.4, 131.2, 131.0, 130.2, 129.3, 128.0, 127.9, 127.73, 127.71, 127.60, 127.56, 127.3, 127.2, 127.0, 126.6, 125.8, 120.9, 118.5.

HRMS: (ESI) m/z: $[M+H]^+$ Calcd for $C_{38}H_{27}O_3^+$ 531.1955; Found 531.1964.

Optical: $[\alpha]_{25}^{D}$ = -3.4 (c = 0.11, CH_2Cl_2, 99% ee).

HPLC (OD-H, ^{i}PrOH/n-hexane = 20/80, flow rate = 0.6 mL/min, 254 nm) t_R = 8.8 min (major), 9.3 min (minor).

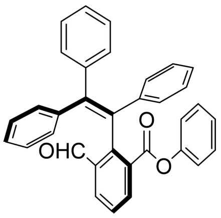

(*R*)-phenyl 3-formyl-2-(1,2,2-triphenylvinyl)benzoate (3b) was synthesized by following Procedure B. The crude material was purified by column chromatography (SiO_2, Toluene) to provide **3b** as a yellow solid (21.2 mg, 44% yield).

^{1}H NMR (400 MHz, $CDCl_3$) δ 10.53 (s, 1H), 8.15 (d, *J* = 7.7 Hz, 1H), 7.99 (d, *J* = 7.9 Hz, 1H), 7.45 (t, *J* = 7.8 Hz, 1H), 7.38 – 7.31 (m, 2H), 7.25 – 7.20 (m, 1H), 7.14 (m, 3H), 7.11 (m, 4H), 7.09 (d, *J* = 1.6 Hz, 2H), 7.07 (m, 4H), 7.02 – 6.96 (m, 2H), 6.86 (d, *J* = 8.4 Hz, 2H).

^{13}C NMR (101 MHz, $CDCl_3$) δ 191.5, 165.0, 150.5, 147.9, 144.4, 142.6, 142.1, 141.3, 135.4, 135.1, 133.3, 132.4, 131.3, 131.2, 131.0, 130.1, 129.4, 128.0, 127.9, 127.7, 127.6, 127.3, 127.2, 126.9, 126.0, 121.5.

HRMS: (ESI) m/z: $[M+H]^+$ Calcd for $C_{34}H_{25}O_3^+$ 481.1978; Found 481.1971.

Optical: $[\alpha]_{25}^{D}$ = -1.4 (c = 0.06, CH_2Cl_2, 97% ee).

HPLC (IA, ^{i}PrOH/n-hexane = 20/80, flow rate = 1.0 mL/min, 254 nm) t_R = 5.9 min (major), 12.5 min (minor).

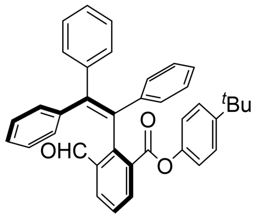

(*R*)-4-(tert-butyl)phenyl 3-formyl-2-(1,2,2-triphenylvinyl)benzoate (3c) was synthesized by following Procedure B. The crude material was purified by column chromatography (SiO_2, Toluene) to provide **3c** as a yellow solid (31.1 mg, 58% yield).

^{1}H NMR (400 MHz, $CDCl_3$) δ 10.53 (s, 1H), 8.13 (d, *J* = 7.7 Hz, 1H), 7.98 (d, *J* = 7.5 Hz, 1H), 7.44 (t, *J* = 7.7 Hz, 1H), 7.35 (d, *J* = 7.7 Hz, 2H), 7.18 – 7.13 (m, 3H), 7.13 – 7.09 (m, 7H), 7.08 – 7.05 (m, 3H), 7.02 – 6.98 (m, 2H), 6.78 (d, *J* = 7.5 Hz, 2H), 1.31 (s, 9H).

^{13}C NMR (101 MHz, $CDCl_3$) δ 191.5, 165.3, 148.8, 148.1, 147.8, 144.3, 142.7, 142.1, 141.3, 135.4, 135.0, 133.3, 132.5, 131.4, 131.1, 131.0, 130.1, 127.94, 127.89, 127.7, 127.5, 127.25, 127.20, 126.9, 126.3, 120.8, 34.4, 31.4.

HRMS: (ESI) m/z: $[M+H]^+$ Calcd for $C_{38}H_{33}O_3^+$ 537.2424; Found 537.2432.

Optical: $[\alpha]_{25}^{D}$ = -6.6 (c = 0.17, CH_2Cl_2, 91% ee).

HPLC (IA, ^{i}PrOH/n-hexane = 20/80, flow rate = 1.0 mL/min, 254 nm) t_R = 4.4 min (major), 5.1 min (minor).

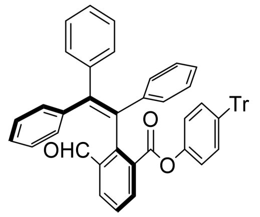

(*R*)-4-tritylphenyl 3-formyl-2-(1,2,2-triphenylvinyl)benzoate (3d) was synthesized by following Procedure B. The crude material was purified by column chromatography (SiO_2, Toluene) to provide **3d** as a yellow solid (51.9 mg, 72% yield).

^{1}H NMR (400 MHz, $CDCl_3$) δ 10.50 (s, 1H), 8.08 (d, *J* = 7.3 Hz, 1H), 7.96 (d, *J* = 7.8 Hz, 1H), 7.42 (t, *J* = 7.7 Hz, 1H), 7.24 (s, 3H), 7.23 – 7.19 (m, 10H), 7.19 – 7.15 (m, 5H), 7.14 (s, 1H), 7.13 (s, 1H), 7.10 (m, 2H), 7.07 – 7.02 (m, 8H), 7.00 – 6.96 (m, 2H), 6.77 (d, *J* = 6.7 Hz, 2H).

^{13}C NMR (101 MHz, $CDCl_3$) δ 191.3, 164.7, 150.3, 147.9, 144.5, 142.6, 142.0, 141.3, 138.4, 135.3, 135.1, 133.1, 132.0, 131.4, 131.3, 131.0, 130.1, 128.0, 127.7, 127.6, 127.33, 127.31, 127.0, 123.7, 90.1.

HRMS: (ESI) m/z: $[M+H]^+$ Calcd for $C_{53}H_{39}O_3^+$ 723.2894; Found 723.2888.

Optical: $[\alpha]_{25}^{D}$ = -4.8 (c = 0.15, CH_2Cl_2, 98% ee).

HPLC (OD-H, ^{i}PrOH/n-hexane = 20/80, flow rate = 0.6 mL/min, 254 nm) t_R = 7.9 min (major), 8.7 min (minor).

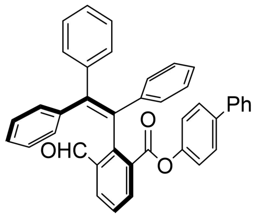

(*R*)- [1,1'-biphenyl]-4-yl 3-formyl-2-(1,2,2-triphenylvinyl)benzoate (3e) was synthesized by following Procedure B. The crude material was purified by column chromatography (SiO_2, Toluene) to provide **3e** as a yellow solid (41.1 mg, 74% yield).

^{1}H NMR (400 MHz, $CDCl_3$) δ 10.54 (s, 1H), 8.17 (d, *J* = 7.7 Hz, 1H), 8.00 (d, *J* = 7.8 Hz, 1H), 7.58 – 7.54 (m, 4H), 7.47 – 7.41 (m, 3H), 7.38 – 7.34 (m, 1H), 7.16 (d, *J* = 1.4 Hz, 1H), 7.14 (s, 1H), 7.14 – 7.07 (m, 11H), 7.01 (m, 2H), 6.94 – 6.91 (m, 2H).

^{13}C NMR (101 MHz, $CDCl_3$) δ 191.5, 165.1, 149.9, 147.9, 144.4, 142.7, 142.1, 141.3, 140.2, 139.2, 135.4, 135.1, 133.3, 132.3, 131.4, 131.3, 131.0, 130.1, 128.8, 128.1, 128.0, 127.9, 127.7, 127.6, 127.4, 127.30, 127.25, 127.1, 127.0, 121.7.

HRMS: (ESI) m/z: $[M+H]^+$ Calcd for $C_{40}H_{29}O_3^+$ 557.2111; Found 557.2105.

Optical: $[\alpha]_{25}^{D}$ = -6.8 (c = 0.15, CH_2Cl_2, 96% ee).

HPLC (IA, ^{i}PrOH/n-hexane = 20/80, flow rate = 1.0 mL/min, 254 nm) t_R = 7.1 min (major), 15.7 min (minor).

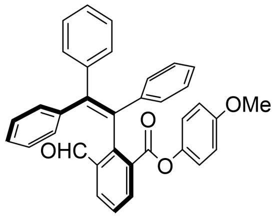

(*R*)-4-methoxyphenyl 3-formyl-2-(1,2,2-triphenylvinyl)benzoate (3f) was synthesized by following Procedure B. The crude material was purified by column chromatography (SiO_2, Toluene) to provide **3f** as a yellow solid (26.5 mg, 52% yield).

^{1}H NMR (400 MHz, $CDCl_3$) δ 10.52 (s, 1H), 8.12 (d, *J* = 7.7 Hz, 1H), 7.97 (d, *J* = 6.4 Hz, 1H), 7.44 (t, *J* = 7.8 Hz, 1H), 7.17 – 7.13 (m, 3H), 7.12 – 7.08 (m, 7H), 7.07 – 7.05 (m, 3H), 7.00 – 6.96 (m, 2H), 6.85 (m, 2H), 6.76 (m, 2H), 3.79 (s, 3H).

^{13}C NMR (101 MHz, $CDCl_3$) δ 191.5, 165.4, 157.3, 147.8, 144.3, 143.9, 142.7, 142.1, 141.3, 135.4, 135.0, 133.3, 132.5, 131.4, 131.1, 131.0, 130.1, 127.94, 127.91, 127.6, 127.5, 127.3, 127.2, 126.9, 122.2, 114.4, 55.6.

HRMS: (ESI) m/z: $[M+H]^+$ Calcd for $C_{35}H_{27}O_4^+$ 511.1904; Found 511.1900.

Optical: $[\alpha]_{25}^{D}$ = -9.3 (c = 0.18, CH_2Cl_2, 97% ee).

HPLC (OD-H, ^{i}PrOH/n-hexane = 20/80, flow rate = 0.6 mL/min, 254 nm) t_R = 8.8 min (major), 9.3 min (minor).

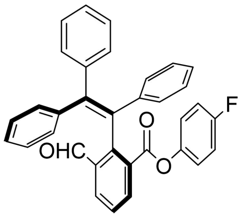

(*R*)-4-fluorophenyl 3-formyl-2-(1,2,2-triphenylvinyl)benzoate (3g) was synthesized by following Procedure B. The crude material was purified by column chromatography (SiO_2, Toluene) to provide **3g** as a yellow solid (36.3 mg, 73% yield).

^{1}**H NMR** (400 MHz, $CDCl_3$) δ 10.51 (s, 1H), 8.12 (d, *J* = 7.7 Hz, 1H), 7.99 (d, *J* = 7.8 Hz, 1H), 7.45 (t, *J* = 7.7 Hz, 1H), 7.15 (m, 3H), 7.11 – 7.10 (m, 3H), 7.09 – 7.06 (m, 6H), 7.05 – 6.95 (m, 5H), 6.82 – 6.78 (m, 2H).

^{13}**C NMR** (101 MHz, $CDCl_3$) δ 191.4, 165.0, 160.3 (d, *J* = 244.6 Hz), 147.9, 146.2 (d, *J* = 2.9 Hz), 144.5, 142.6, 142.0, 141.3, 135.3, 135.1, 133.2, 132.1, 131.3, 131.0, 130.1, 128.0, 127.7, 127.6, 127.33, 127.29, 127.0, 122.9 (d, *J* = 8.5 Hz), 116.0 (d, *J* = 23.5 Hz).

^{19}**F NMR** (565 MHz, $CDCl_3$) δ -116.47.

HRMS: (ESI) m/z: $[M+H]^+$ Calcd for $C_{34}H_{24}FO_3^+$ 499.1704; Found 499.1700.

Optical: $[\alpha]_{25}^{D}$ = -8.7 (c = 0.18, CH_2Cl_2, 96% ee).

HPLC (IA, ^{i}PrOH/n-hexane = 20/80, flow rate = 1.0 mL/min, 254 nm) t_R = 5.4 min (major), 12.0 min (minor).

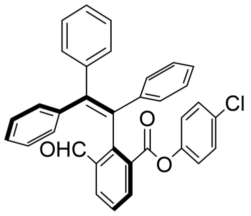

(*R*)-4-chlorophenyl 3-formyl-2-(1,2,2-triphenylvinyl)benzoate (3h) was synthesized by following Procedure B. The crude material was purified by column chromatography (SiO_2, Toluene) to provide **3h** as a yellow solid (45.7 mg, 89% yield).

^{1}**H NMR** (600 MHz, $CDCl_3$) δ 10.52 (s, 1H), 8.12 (d, *J* = 7.7 Hz, 1H), 7.99 (d, *J* = 7.9 Hz, 1H), 7.46 (t, *J* = 7.8 Hz, 1H), 7.31 (s, 1H), 7.30 (s, 1H), 7.17 – 7.14 (m, 3H), 7.11 – 7.08 (m, 5H), 7.07 (s, 5H), 6.97 (t, *J* = 1.5 Hz, 1H), 6.96 (d, *J* = 1.8 Hz, 1H), 6.79 (s, 1H), 6.77 (s, 1H).

^{13}**C NMR** (151 MHz, $CDCl_3$) δ 191.4, 164.8, 148.9, 147.9, 144.5, 142.6, 142.0, 141.3, 135.3, 135.1, 133.1, 132.0, 131.4, 131.3, 131.0, 130.1, 129.4, 128.0, 127.7, 127.6, 127.33, 127.30, 127.0, 122.8.

HRMS: (ESI) m/z: $[M+H]^+$ Calcd for $C_{34}H_{24}ClO_3^+$ 515.1408; Found 515.1404.

Optical: $[\alpha]_{25}^{D}$ = -8.7 (c = 0.16, CH_2Cl_2, 99% ee).

HPLC (OD-H, ^{i}PrOH/n-hexane = 20/80, flow rate = 0.8 mL/min, 254 nm) t_R = 5.6 min (major), 6.1 min (minor).

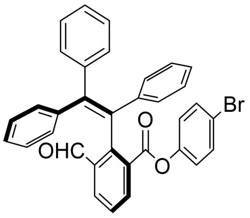

(*R*)-4-bromophenyl 3-formyl-2-(1,2,2-triphenylvinyl)benzoate (3i) was synthesized by following Procedure B. The crude material was purified by column chromatography (SiO_2, Toluene) to provide **3i** as a yellow solid (45.8 mg, 82% yield).

^{1}H NMR (400 MHz, $CDCl_3$) δ 10.52 (s, 1H), 8.11 (d, *J* = 7.7 Hz, 1H), 7.99 (d, *J* = 7.8 Hz, 1H), 7.46 (d, *J* = 8.8 Hz, 3H), 7.17 – 7.13 (m, 3H), 7.12 – 7.06 (m, 10H), 6.97 (m, 2H), 6.73 (d, *J* = 8.7 Hz, 2H).

^{13}C NMR (101 MHz, $CDCl_3$) δ 191.3, 164.7, 144.5, 142.6, 142.0, 141.3, 135.3, 135.1, 133.1, 132.4, 132.0, 131.4, 131.3, 131.0, 130.1, 128.0, 127.7, 127.6, 127.35, 127.32, 127.0, 123.3, 119.1.

HRMS: (ESI) m/z: $[M+H]^+$ Calcd for $C_{34}H_{24}BrO_3^+$ 559.0903; Found 559.0898.

Optical: $[\alpha]_{25}^{D}$ = -99.1 (c = 0.61, CH_2Cl_2, 89% ee).

HPLC (IA, ^{i}PrOH/n-hexane = 20/80, flow rate = 1.0 mL/min, 254 nm) t_R = 7.3 min (major), 29.4 min (minor).

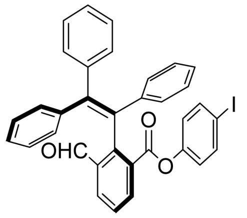

(*R*)-4-iodophenyl 3-formyl-2-(1,2,2-triphenylvinyl)benzoate (3j) was synthesized by following Procedure B. The crude material was purified by column chromatography (SiO_2, Toluene) to provide **3j** as a yellow solid (50.9 mg, 84% yield).

^{1}H NMR (400 MHz, $CDCl_3$) δ 10.51 (s, 1H), 8.10 (d, *J* = 6.2 Hz, 1H), 7.99 (d, *J* = 7.8 Hz, 1H), 7.65 (d, *J* = 8.7 Hz, 2H), 7.45 (t, *J* = 7.7 Hz, 1H), 7.18 – 7.13 (m, 3H), 7.11 – 7.07 (m, 5H), 7.06 (s, 5H), 6.97 – 6.94 (m, 2H), 6.61 (d, *J* = 8.8 Hz, 2H).

^{13}C NMR (101 MHz, $CDCl_3$) δ 191.5, 164.9, 148.4, 147.9, 146.5, 144.5, 144.3, 142.6, 142.1, 141.3, 135.4, 135.1, 133.4, 132.5, 132.1, 131.3, 131.2, 131.08, 131.06, 130.2, 127.9, 127.9, 127.7, 127.6, 127.5, 127.3, 127.2, 127.0, 126.0, 120.2.

HRMS: (ESI) m/z: $[M+H]^+$ Calcd for $C_{34}H_{24}IO_3^+$ 607.0765; Found 607.0757.

Optical: $[\alpha]_{25}^{D}$ = -8.9 (c = 0.17, CH_2Cl_2, 98% ee).

HPLC (OD-H, ^{i}PrOH/n-hexane = 20/80, flow rate = 0.6 mL/min, 254 nm) t_R = 7.8

min (major), 8.6min (minor).

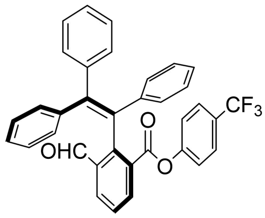

(*R*)-4-(trifluoromethyl)phenyl 3-formyl-2-(1,2,2-triphenylvinyl)benzoate (3k) was synthesized by following Procedure B. The crude material was purified by column chromatography (SiO_2, Toluene) to provide **3k** as a yellow solid (41.6 mg, 76% yield).

^{1}H NMR (400 MHz, $CDCl_3$) δ 10.52 (s, 1H), 8.14 (d, J = 7.7 Hz, 1H), 8.01 (d, J = 7.8 Hz, 1H), 7.62 (d, J = 8.4 Hz, 2H), 7.47 (t, J = 7.8 Hz, 1H), 7.20 – 7.14 (m, 3H), 7.12 – 7.08 (m, 5H), 7.07 (s, 5H), 6.99 – 6.95 (m, 4H).

^{13}C NMR (101 MHz, $CDCl_3$) δ 191.3, 164.5, 152.9, 148.0, 144.7, 142.6, 142.0, 141.3, 135.3, 135.2, 133.0, 131.8, 131.6, 131.3, 131.0, 130.1, 128.0, 127.72, 127.65, 127.39, 127.37, 127.0, 126.8, 126.8 (q, J = 3.7 Hz), 123.8 (q, J = 272.7 Hz), 122.0.

^{19}F NMR (565 MHz, $CDCl_3$) δ -62.13.

HRMS: (ESI) m/z: $[M+H]^+$ Calcd for $C_{35}H_{24}F_3O_3^+$ 549.1672; Found 549.1667.

Optical: $[\alpha]_{25}^{D}$ = -7.7(c = 0.17, CH_2Cl_2, 97% ee).

HPLC (IA, ^{i}PrOH/n-hexane = 20/80, flow rate = 1.0 mL/min, 254 nm) t_R = 4.8 min (major), 9.8 min (minor).

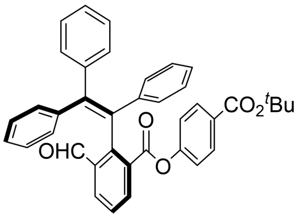

(*R*)-4-(tert-butoxycarbonyl)phenyl 3-formyl-2-(1,2,2-triphenylvinyl)benzoate (3l) was synthesized by following Procedure B. The crude material was purified by column chromatography (SiO_2, Toluene) to provide **3l** as a yellow solid (52.4 mg, 88% yield).

^{1}H NMR (400 MHz, $CDCl_3$) δ 10.52 (s, 1H), 8.14 (d, J = 7.7 Hz, 1H), 8.01 – 7.97 (m, 3H), 7.48 – 7.44 (m, 1H), 7.16 – 7.13 (m, 3H), 7.11 – 7.08 (m, 5H), 7.07 (s, 5H), 6.96 (m, 2H), 6.91 (d, J = 8.7 Hz, 2H), 1.59 (s, 9H).

^{13}C NMR (101 MHz, $CDCl_3$) δ 191.3, 164.8, 164.5, 153.7, 147.9, 144.5, 142.5, 142.0, 141.3, 135.4, 135.1, 133.2, 132.0, 131.4, 131.3, 130.95, 130.87, 130.1, 129.7, 128.0, 127.7, 127.6, 127.32, 127.27, 127.0, 121.3, 81.2, 28.1.

HRMS: (ESI) m/z: $[M+H]^+$ Calcd for $C_{39}H_{33}O_5^+$ 581.2323; Found 581.2316.

Optical: $[\alpha]_{25}^{D}$ = -5.7 (c = 0.15, CH_2Cl_2, 98% ee).

HPLC (IA, ^{i}PrOH/n-hexane = 20/80, flow rate = 1.0 mL/min, 254 nm) t_R = 4.6 min (major), 6.5 min (minor).

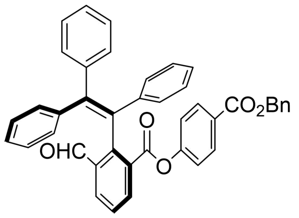

(*R*)-4-((benzyloxy)carbonyl)phenyl 3-formyl-2-(1,2,2-triphenylvinyl)benzoate (3m) was synthesized by following Procedure B. The crude material was purified by column chromatography (SiO_2, Toluene) to provide **3m** as a yellow solid (52.1 mg, 85% yield).

^{1}H NMR (400 MHz, $CDCl_3$) δ 10.52 (s, 1H), 8.13 (d, *J* = 7.7 Hz, 1H), 8.09 (d, *J* = 8.8 Hz, 2H), 8.00 (d, *J* = 6.3 Hz, 1H), 7.47 – 7.43 (m, 3H), 7.41 – 7.33 (m, 3H), 7.17 – 7.14 (m, 3H), 7.11 (m, 4H), 7.09 (s, 1H), 7.07 (s, 5H), 6.99 – 6.96 (m, 2H), 6.95 – 6.92 (m, 2H), 5.37 (s, 2H).

^{13}C NMR (101 MHz, $CDCl_3$) δ 191.3, 165.5, 164.4, 154.1, 148.0, 144.5, 142.5, 142.0, 141.3, 135.8, 135.4, 135.2, 133.1, 131.9, 131.5, 131.3, 131.2, 130.9, 130.1, 128.6, 128.3, 128.1, 128.0, 127.8, 127.7, 127.6, 127.33, 127.29, 127.0, 121.5, 66.8.

HRMS: (ESI) m/z: $[M+H]^+$ Calcd for $C_{42}H_{31}O_5^+$ 615.2166; Found 615.2156.

Optical: $[\alpha]_{25}^{D}$ = -9.6 (c = 0.19, CH_2Cl_2, 98% ee).

HPLC (IA, ^{i}PrOH/n-hexane = 20/80, flow rate = 1.0 mL/min, 254 nm) t_R = 7.4 min (major), 20.0 min (minor).

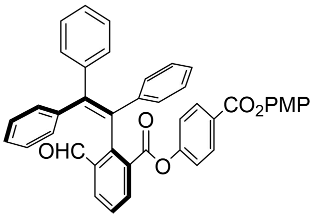

(*R*)-4-((4-methoxyphenoxy) carbonyl) phenyl 3-formyl-2-(1,2,2-triphenylvinyl) benzoate (3n) was synthesized by following Procedure B. The crude material was purified by column chromatography (SiO_2, Toluene) to provide **3n** as a yellow solid

(42.2 mg, 67% yield).

¹H NMR (400 MHz, $CDCl_3$) δ 10.53 (s, 1H), 8.2 (s, 1H), 8.2 (s, 1H), 8.15 (d, *J* = 6.2 Hz, 1H), 8.01 (d, *J* = 6.4 Hz, 1H), 7.48 (t, *J* = 7.7 Hz, 1H), 7.18 – 7.14 (m, 3H), 7.13 (s, 1H), 7.13 – 7.11 (m, 4H), 7.10 – 7.10 (m, 1H), 7.08 (s, 6H), 7.01 – 6.97 (m, 4H), 6.96 (s, 1H), 6.94 (s, 1H), 3.83 (s, 3H).

¹³C NMR (101 MHz, $CDCl_3$) δ 191.3, 164.6, 164.4, 157.3, 154.5, 148.0, 144.6, 144.2, 142.5, 142.0, 141.3, 135.4, 135.2, 133.1, 131.9, 131.7, 131.5, 131.3, 131.0, 130.1, 128.0, 127.7, 127.6, 127.4, 127.33, 127.31, 127.0, 122.3, 121.7, 114.5, 55.6.

HRMS: (ESI) m/z: $[M+H]^+$ Calcd for $C_{42}H_{31}O_6^+$ 631.2115; Found 631.2106.

Optical: $[\alpha]_{25}^{D}$ = -6.4 (c = 0.16, CH_2Cl_2, 98% ee).

HPLC (OD-H ^{i}PrOH/n-hexane = 20/80, flow rate = 1.0 mL/min, 254 nm) t_R = 15.2 min (major), 18.7 min (minor).

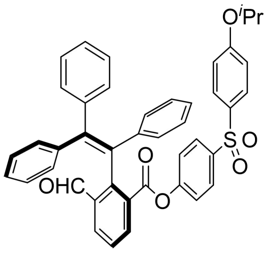

(*R*)-4-((4-isopropoxyphenyl) sulfonyl) phenyl 3-formyl-2-(1,2,2-triphenylvinyl) benzoate (3o) was synthesized by following Procedure B. The crude material was purified by column chromatography (SiO_2, Toluene) to provide **3o** as a yellow solid (63.7 mg, 94% yield).

¹H NMR (400 MHz, $CDCl_3$) δ 10.49 (s, 1H), 8.10 (dd, *J* = 7.7, 1.5 Hz, 1H), 7.99 (dd, *J* = 7.8, 1.5 Hz, 1H), 7.91 (s, 1H), 7.89 (s, 1H), 7.85 (s, 1H), 7.83 (s, 1H), 7.45 (m, 1H), 7.15 (d, *J* = 1.9 Hz, 1H), 7.14 – 7.10 (m, 2H), 7.10 – 7.06 (m, 5H), 7.05 (t, *J* = 2.5 Hz, 5H), 6.96 – 6.92 (m, 6H), 4.61 (m, 1H), 1.35 (s, 3H), 1.34 (s, 3H).

¹³C NMR (101 MHz, $CDCl_3$) δ 191.2, 164.3, 162.0, 153.6, 148.0, 144.6, 142.5, 141.9, 141.2, 140.0, 135.3, 135.2, 133.0, 132.0, 131.7, 131.5, 131.2, 130.9, 130.0, 129.9, 128.9, 128.0, 127.7, 127.6, 127.4, 127.3, 127.0, 122.3, 115.8, 70.4, 21.7.

HRMS: (ESI) m/z: $[M+H]^+$ Calcd for $C_{43}H_{35}O_6S^+$ 679.2149; Found 679.2139.

Optical: $[\alpha]_{25}^{D}$ = -125.4 (c = 0.74, CH_2Cl_2, 99% ee).

HPLC (IA, ^{i}PrOH/n-hexane = 20/80, flow rate = 1.0 mL/min, 254 nm) t_R = 11.6 min (major), 16.1 min (minor).

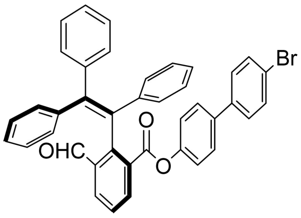

(*R*)-4'-bromo-[1,1'-biphenyl]-4-yl 3-formyl-2-(1,2,2-triphenylvinyl)benzoate (3p) was synthesized by following Procedure B. The crude material was purified by column chromatography (SiO_2, Toluene) to provide **3p** as a yellow solid (42.9 mg, 66% yield).

^{1}H NMR (400 MHz, $CDCl_3$) δ 10.54 (s, 1H), 8.16 (dd, *J* = 7.7, 1.5 Hz, 1H), 8.00 (dd, *J* = 7.9, 1.5 Hz, 1H), 7.58 – 7.54 (m, 2H), 7.53 – 7.50 (m, 2H), 7.47 (td, *J* = 7.8, 0.8 Hz, 1H), 7.43 – 7.40 (m, 2H), 7.17 – 7.14 (m, 3H), 7.13 – 7.11 (m, 4H), 7.09 (td, *J* = 6.0, 3.1 Hz, 6H), 7.02 – 6.99 (m, 2H), 6.94 – 6.91 (m, 2H).

^{13}C NMR (101 MHz, $CDCl_3$) δ 191.4, 165.1, 150.1, 147.9, 144.5, 142.6, 142.1, 141.3, 139.2, 137.9, 135.4, 135.1, 133.2, 132.2, 131.9, 131.4, 131.3, 131.0, 130.1, 128.7, 127.98, 127.96, 127.7, 127.6, 127.32, 127.27, 127.0, 121.9, 121.7.

HRMS: (ESI) m/z: $[M+H]^+$ Calcd for $C_{40}H_{28}BrO_3^+$ 635.1216; Found 635.1208.

Optical: $[\alpha]_{25}^{D}$ = -76.3 (c = 0.61, CH_2Cl_2, 98% ee).

HPLC (OD-H, ^{i}PrOH/n-hexane = 20/80, flow rate = 0.6 mL/min, 254 nm) t_R = 9.0 min (major), 9.9 min (minor).

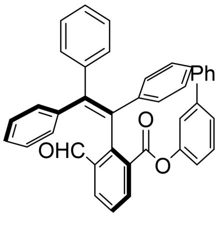

(*R*)-[1,1'-biphenyl]-3-yl 3-formyl-2-(1,2,2-triphenylvinyl) benzoate (3q) was synthesized by following Procedure B. The crude material was purified by column chromatography (SiO_2, Toluene) to provide **3q** as a yellow solid (43.4 mg, 76% yield).

^{1}H NMR (400 MHz, $CDCl_3$) δ 10.54 (s, 1H), 8.18 (dd, *J* = 7.7, 1.5 Hz, 1H), 8.00 (m, 1H), 7.56 – 7.52 (m, 2H), 7.45 (m, 5H), 7.40 – 7.37 (m, 1H), 7.15 (dt, *J* = 5.4, 1.5 Hz, 3H), 7.14 – 7.11 (m, 4H), 7.11 – 7.09 (m, 2H), 7.09 – 7.06 (m, 4H), 7.01 (m, 3H), 6.87 (m, 1H).

^{13}C NMR (101 MHz, $CDCl_3$) δ 191.4, 165.1, 150.8, 147.9, 144.5, 142.8, 142.6, 142.1, 141.3, 140.0, 135.3, 135.1, 133.2, 132.4, 131.4, 131.2, 131.0, 130.2, 129.7, 128.7, 128.0, 127.9, 127.7, 127.6, 127.3, 127.2, 127.1, 127.0, 124.7, 120.3, 120.2.

HRMS: (ESI) m/z: $[M+H]^+$ Calcd for $C_{40}H_{29}O_5^+$ 557.2111; Found 557.2103.

Optical: $[\alpha]_{25}^{D}$ = -67.6 (c = 0.5, CH_2Cl_2, 94% ee).

HPLC (IA, ^{i}PrOH/n-hexane = 20/80, flow rate = 1.0 mL/min, 254 nm) t_R = 5.2 min (major), 5.7 min (minor).

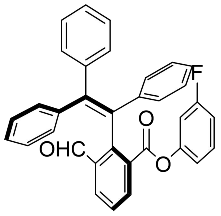

(*R*)-3-fluorophenyl 3-formyl-2-(1,2,2-triphenylvinyl) benzoate (3r) was synthesized by following Procedure B. The crude material was purified by column chromatography (SiO_2, Toluene) to provide **3r** as a yellow solid (33.8 mg, 68% yield).

^{1}H NMR (400 MHz, $CDCl_3$) δ 10.52 (s, 1H), 8.11 (dd, *J* = 7.7, 1.5 Hz, 1H), 7.99 (dd, *J* = 7.8, 1.5 Hz, 1H), 7.46 (m, 1H), 7.30 (m, 1H), 7.18 – 7.13 (m, 3H), 7.13 – 7.09 (m, 5H), 7.07 (s, 5H), 6.99 – 6.92 (m, 3H), 6.69 (dd, *J* = 8.2, 2.2 Hz, 1H), 6.57 (m, 1H).

^{13}C NMR (101 MHz, $CDCl_3$) δ 191.4, 164.6, 162.8 (d, *J* = 247.8 Hz), 151.2 (d, *J* = 10.7 Hz), 144.6, 142.6, 142.0, 141.3, 135.3, 135.1, 133.1, 132.0, 131.4, 131.33, 131.30, 131.0, 130.2, 130.12, 130.09, 128.0, 127.72, 127.69, 127.6, 127.3 (d, *J* = 3.7 Hz), 127.0, 117.3 (d, *J* = 3.5 Hz), 113.1 (d, *J* = 20.9 Hz), 109.7 (d, *J* = 24.5 Hz).

^{19}F NMR (565 MHz, $CDCl_3$) δ -110.74.

HRMS: (ESI) m/z: $[M+H]^+$ Calcd for $C_{34}H_{24}FO_3^+$ 499.1704; Found 499.1698.

Optical: $[\alpha]_{25}^{D}$ = -7.6 (c = 0.17, CH_2Cl_2, 96% ee).

HPLC (IA, ^{i}PrOH/n-hexane = 20/80, flow rate = 1.0 mL/min, 254 nm) t_R = 4.9 min (major), 7.2 min (minor).

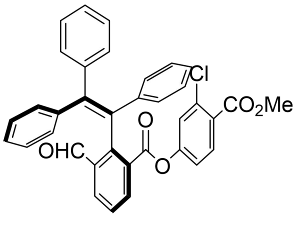

(*R*)-3-chloro-4-(methoxycarbonyl) phenyl 3-formyl-2-(1,2,2-triphenylvinyl) benzoate (3s) was synthesized by following Procedure B. The crude material was

purified by column chromatography (SiO_2, Toluene) to provide **3s** as a yellow solid (48.1 mg, 84% yield).

^{1}H NMR (400 MHz, $CDCl_3$) δ 10.50 (s, 1H), 8.09 (dd, *J* = 7.7, 1.5 Hz, 1H), 7.99 (dd, *J* = 7.8, 1.5 Hz, 1H), 7.86 (d, *J* = 8.6 Hz, 1H), 7.46 (m, 1H), 7.20 – 7.14 (m, 3H), 7.10 (m, 5H), 7.08 – 7.03 (m, 5H), 6.97 – 6.93 (m, 2H), 6.92 (d, *J* = 2.3 Hz, 1H), 6.85 (dd, *J* = 8.6, 2.3 Hz, 1H), 3.92 (s, 3H).

^{13}C NMR (101 MHz, $CDCl_3$) δ 191.1, 165.2, 164.1, 152.9, 148.0, 144.7, 142.5, 141.9, 141.2, 135.2, 134.8, 132.9, 132.5, 131.6, 131.3, 130.9, 130.1, 128.0, 127.98, 127.8, 127.6, 127.39, 127.36, 127.1, 124.4, 119.9, 52.4.

HRMS: (ESI) m/z: $[M+H]^+$ Calcd for $C_{36}H_{26}ClO_5^+$ 573.1463; Found 573.1459.

Optical: $[\alpha]_{25}^{D}$ = -13.8 (c = 0.22, CH_2Cl_2, 98% ee).

HPLC (IA, ^{i}PrOH/n-hexane = 20/80, flow rate = 1.0 mL/min, 254 nm) t_R = 6.0 min (major), 16.6 min (minor).

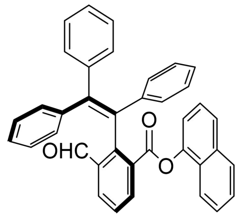

(*R*)-Naphthalen-1-yl 3-formyl-2-(1,2,2-triphenylvinyl) benzoate (3t) was synthesized by following Procedure B. The crude material was purified by column chromatography (SiO_2, Toluene) to provide **3t** as a yellow solid (44.0 mg, 83% yield).

^{1}H NMR (400 MHz, $CDCl_3$) δ 10.58 (s, 1H), 8.42 (dd, *J* = 7.7, 1.5 Hz, 1H), 8.05 (dd, *J* = 7.8, 1.5 Hz, 1H), 7.88 – 7.84 (m, 1H), 7.75 (d, *J* = 8.4 Hz, 1H), 7.56 – 7.47 (m, 2H), 7.43 (dd, *J* = 8.3, 7.5 Hz, 1H), 7.39 – 7.35 (m, 2H), 7.13 (m, 6H), 7.10 – 7.06 (m, 7H), 7.01 – 6.98 (m, 3H).

^{13}C NMR (101 MHz, $CDCl_3$) δ 191.5, 164.8, 148.4, 146.5, 144.4, 142.7, 142.0, 141.2, 135.7, 135.2, 134.6, 133.1, 131.9, 131.5, 131.3, 131.0, 130.1, 128.0, 127.9, 127.73, 127.69, 127.3, 127.2, 127.0, 126.7, 126.6, 126.4, 126.2, 125.3, 121.0, 118.0.

HRMS: (ESI) m/z: $[M+H]^+$ Calcd for $C_{38}H_{27}O_3^+$ 531.1955; Found 531.1953.

Optical: $[\alpha]_{25}^{D}$ = -20.2 (c = 0.21, CH_2Cl_2, 96% ee).

HPLC (OD-H, ^{i}PrOH/n-hexane = 5/95, flow rate = 0.8 mL/min, 254 nm) t_R = 10.1 min (major), 11.1 min (minor).

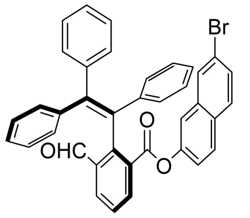

(*R*)-7-bromonaphthalen-2-yl 3-formyl-2-(1,2,2-triphenylvinyl) benzoate (3u) was synthesized by following Procedure B. The crude material was purified by column chromatography (SiO_2, Toluene) to provide **3u** as a yellow solid (51.9 mg, 84% yield).

^{1}H NMR (400 MHz, $CDCl_3$) δ 10.55 (s, 1H), 8.19 (dd, *J* = 7.6, 1.5 Hz, 1H), 8.01 (dd, *J* = 7.8, 1.5 Hz, 1H), 7.92 (d, *J* = 1.9 Hz, 1H), 7.78 (d, *J* = 8.9 Hz, 1H), 7.70 (d, *J* = 8.7 Hz, 1H), 7.55 – 7.46 (m, 2H), 7.22 (d, *J* = 2.2 Hz, 1H), 7.16 (dd, *J* = 6.2, 2.2 Hz, 2H), 7.13 (d, *J* = 5.6 Hz, 6H), 7.11 – 7.08 (m, 5H), 6.99 (m, 3H).

^{13}C NMR (101 MHz, $CDCl_3$) δ 191.4, 165.0, 148.9, 147.9, 144.5, 142.6, 142.0, 141.3, 135.4, 135.1, 134.7, 133.2, 132.2, 131.4, 131.0, 130.1, 129.8, 129.5, 129.4, 129.2, 127.99, 127.95, 127.7, 127.6, 127.34, 127.28, 127.0, 121.4, 120.8, 117.8.

HRMS: (ESI) m/z: $[M+H]^+$ Calcd for $C_{38}H_{26}BrO_3^+$ 609.1060; Found 609.1054.

Optical: $[\alpha]_{25}^{D}$ = -8.7 (c = 0.17, CH_2Cl_2, 99% ee).

HPLC (OD-H, ^{i}PrOH/n-hexane = 20/80, flow rate = 0.6 mL/min, 254 nm) t_R = 9.1 min (major), 9.7 min (minor).

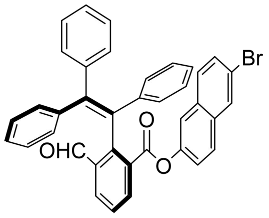

(*R*)-6-bromonaphthalen-2-yl 3-formyl-2-(1,2,2-triphenylvinyl) benzoate (3v) was synthesized by following Procedure B. The crude material was purified by column chromatography (SiO_2, Toluene) to provide **3v** as a yellow solid (49.8 mg, 82% yield).

^{1}H NMR (400 MHz, $CDCl_3$) δ 10.54 (s, 1H), 8.19 (d, *J* = 7.7 Hz, 1H), 8.03 – 7.99 (m, 2H), 7.72 (d, *J* = 8.9 Hz, 1H), 7.63 (d, *J* = 8.8 Hz, 1H), 7.56 (dd, *J* = 8.8, 1.9 Hz, 1H), 7.48 (t, *J* = 7.7 Hz, 1H), 7.29 (d, *J* = 2.2 Hz, 1H), 7.22 – 7.11 (m, 9H), 7.10 (s, 4H), 7.03 – 6.96 (m, 3H).

^{13}C NMR (101 MHz, $CDCl_3$) δ 191.4, 165.1, 148.3, 147.9, 144.6, 142.6, 142.0, 141.4, 135.4, 135.1, 133.2, 132.4, 132.2, 132.0, 131.40, 131.36, 131.0, 130.1, 130.0, 129.8,

129.2, 128.4, 127.98, 127.95, 127.7, 127.6, 127.34, 127.28, 127.0, 122.0, 119.7, 118.6.

HRMS: (ESI) m/z: $[M+H]^+$ Calcd for $C_{38}H_{26}BrO_3^+$ 609.1060; Found 609.1051.

Optical: $[\alpha]_{25}^{D}$ = -8.8 (c = 0.15, CH_2Cl_2, 98% ee).

HPLC (OD-H, ^{i}PrOH/n-hexane = 20/80, flow rate = 0.6 mL/min, 254 nm) t_R = 9.0 min (major), 9.9 min (minor).

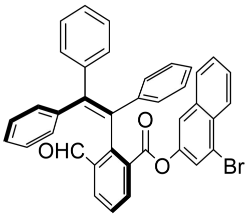

(*R*)-4-bromonaphthalen-2-yl 3-formyl-2-(1,2,2-triphenylvinyl) benzoate (3w) was synthesized by following Procedure B. The crude material was purified by column chromatography (SiO_2, Toluene) to provide **3w** as a yellow solid (47.4 mg, 78% yield).

^{1}H NMR (400 MHz, $CDCl_3$) δ 10.54 (s, 1H), 8.21 (dd, *J* = 8.4, 1.3 Hz, 1H), 8.16 (dd, *J* = 7.6, 1.5 Hz, 1H), 8.01 (dd, *J* = 7.8, 1.5 Hz, 1H), 7.75 (dd, *J* = 8.0, 1.6 Hz, 1H), 7.60 – 7.53 (m, 2H), 7.50 – 7.46 (m, 1H), 7.32 (d, *J* = 2.2 Hz, 1H), 7.21 – 7.17 (m, 3H), 7.16 – 7.12 (m, 6H), 7.10 (m, 5H), 7.00 (m, 2H).

^{13}C NMR (101 MHz, $CDCl_3$) δ 191.3, 165.1, 147.9, 147.3, 144.8, 142.6, 142.0, 141.3, 135.2, 135.1, 134.1, 133.0, 132.1, 131.5, 131.4, 131.0, 130.2, 128.1, 128.02, 127.99, 127.8, 127.6, 127.4, 127.3, 127.2, 127.1, 127.0, 125.1, 123.1, 118.7.

HRMS: (ESI) m/z: $[M+H]^+$ Calcd for $C_{38}H_{26}BrO_3^+$ 609.1060; Found 609.1052.

Optical: $[\alpha]_{25}^{D}$ = -10.4 (c = 0.18, CH_2Cl_2, 98% ee).

HPLC (OD-H, ^{i}PrOH/n-hexane = 20/80, flow rate = 0.6 mL/min, 254 nm) t_R = 8.6 min (major), 9.4 min (minor).

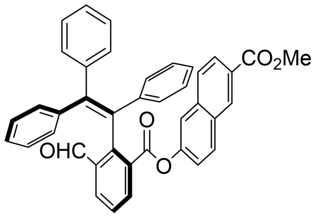

(*R*)-methyl 6-((3-formyl-2-(1,2,2-triphenylvinyl)benzoyl)oxy)-2-naphthoate (3x) was synthesized by following Procedure B. The crude material was purified by column chromatography (SiO_2, Toluene) to provide **3x** as a yellow solid (53.5 mg,

91% yield).

^{1}H NMR (400 MHz, $CDCl_3$) δ 10.54 (s, 1H), 8.60 (d, *J* = 1.7 Hz, 1H), 8.19 (dd, *J* = 7.7, 1.5 Hz, 1H), 8.08 (dd, *J* = 8.6, 1.7 Hz, 1H), 8.01 (dd, *J* = 7.8, 1.5 Hz, 1H), 7.93 (d, *J* = 8.7 Hz, 1H), 7.80 (d, *J* = 8.6 Hz, 1H), 7.48 (m, 1H), 7.35 (d, *J* = 2.2 Hz, 1H), 7.17 – 7.14 (m, 3H), 7.13 (d, *J* = 4.4 Hz, 5H), 7.09 (m, 5H), 7.02 (m, 3H), 3.98 (s, 3H).

^{13}C NMR (101 MHz, $CDCl_3$) δ 191.3, 167.0, 165.0, 149.9, 147.9, 144.6, 142.6, 142.0, 141.3, 135.9, 135.4, 135.2, 133.1, 132.1, 131.41, 131.39, 131.0, 130.9, 130.8, 130.5, 130.1, 128.0, 127.9, 127.8, 127.7, 127.6, 127.4, 127.34, 127.29, 127.0, 126.0, 121.8, 118.6, 52.3.

HRMS: (ESI) m/z: $[M+H]^+$ Calcd for $C_{40}H_{29}O_5^+$ 589.2010; Found 589.1999.

Optical: $[\alpha]_{25}^{D}$ = -7.0 (c = 0.14, CH_2Cl_2, 97% ee).

HPLC (OD-H, ^{i}PrOH/n-hexane = 20/80, flow rate = 0.6 mL/min, 254 nm) t_R = 11.6 min (major), 13.1 min (minor).

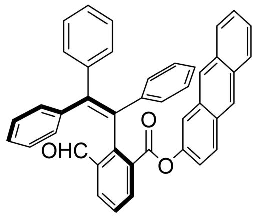

(*R*)-anthracen-2-yl 3-formyl-2-(1,2,2-triphenylvinyl) benzoate (3y) was synthesized by following Procedure B. The crude material was purified by column chromatography (SiO_2, Toluene) to provide **3y** as a yellow solid (42.9 mg, 74% yield).

^{1}H NMR (400 MHz, $CDCl_3$) δ 10.56 (s, 1H), 8.42 (s, 1H), 8.34 (s, 1H), 8.23 (dd, *J* = 7.7, 1.5 Hz, 1H), 8.04 – 7.96 (m, 4H), 7.52 – 7.46 (m, 4H), 7.20 – 7.13 (m, 10H), 7.12 – 7.09 (m, 3H), 7.06 – 7.02 (m, 2H), 6.94 (dd, *J* = 9.3, 2.3 Hz, 1H).

^{13}C NMR (101 MHz, $CDCl_3$) δ 191.5, 165.2, 147.9, 147.7, 144.5, 142.7, 142.1, 141.4, 135.5, 135.1, 133.3, 132.4, 132.0, 131.5, 131.41, 131.35, 131.3, 131.0, 130.2, 130.1, 129.8, 129.7, 128.2, 128.1, 128.05, 127.99, 127.94, 127.85, 127.7, 127.6, 127.3, 127.2, 127.0, 126.4, 125.9, 125.8, 125.4, 121.3, 117.9.

HRMS: (ESI) m/z: $[M+H]^+$ Calcd for $C_{42}H_{29}O_3^+$ 581.2111; Found 581.2101.

Optical: $[\alpha]_{25}^{D}$ = -1.6 (c = 0.08, CH_2Cl_2, 96% ee).

HPLC (OD-H, ^{i}PrOH/n-hexane = 20/80, flow rate = 0.6 mL/min, 254 nm) t_R = 10.9 min (major), 12.3 min (minor).

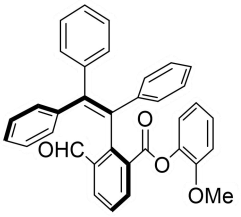

(*R*)-2-methoxyphenyl 3-formyl-2-(1,2,2-triphenylvinyl) benzoate (4a) was synthesized by following Procedure B. The crude material was purified by column chromatography (SiO_2, Toluene) to provide **4a** as a yellow solid (27.5 mg, 54% yield).

^{1}H NMR (600 MHz, $(CD_3)_2SO$) δ 9.52 (s, 1H), 7.27 (d, *J* = 7.8 Hz, 1H), 6.98 (d, *J* = 7.8 Hz, 1H), 6.62 (t, *J* = 7.8 Hz, 1H), 6.28 (t, *J* = 7.8 Hz, 1H), 6.19 (d, *J* = 2.7 Hz, 3H), 6.15 – 6.08 (m, 10H), 6.01 (d, *J* = 7.1 Hz, 2H), 5.98 (d, *J* = 7.7 Hz, 1H), 5.79 (m, 2H), 2.78 (s, 3H).

^{13}C NMR (101 MHz, $(CD_3)_2SO$) δ 191.4, 163.9, 150.8, 146.6, 143.0, 142.4, 142.0, 140.9, 139.0, 135.6, 134.8, 133.5, 131.5, 131.4, 130.9, 130.4, 129.4, 128.3, 128.04, 127.98, 127.6, 127.22, 127.19, 126.9, 122.6, 120.9, 120.5, 119.2, 115.6, 112.9, 112.3, 55.9.

HRMS: (ESI) m/z: $[M+H]^+$ Calcd for $C_{35}H_{27}O_4^+$ 511.1904; Found 511.1901.

Optical: $[\alpha]_{25}^{D}$ = -18.5 (c = 0.24, CH_2Cl_2, 98% ee).

HPLC (OD-H, ^{i}PrOH/n-hexane = 20/80, flow rate = 0.8 mL/min, 254 nm) t_R = 6.4 min (major), 6.8 min (minor).

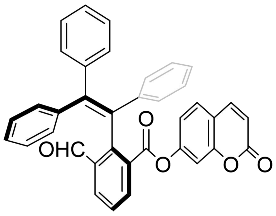

(*R*)-2-oxo-2*H*-chromen-7-yl 3-formyl-2-(1,2,2-triphenylvinyl) benzoate (4b) was synthesized by following Procedure B. The crude material was purified by column chromatography (SiO_2, Toluene) to provide **4b** as a yellow solid (35.6 mg, 65% yield).

^{1}H NMR (600 MHz, $CDCl_3$) δ 10.51 (s, 1H), 8.13 (dd, *J* = 7.7, 1.5 Hz, 1H), 8.01 (dd, *J* = 7.8, 1.5 Hz, 1H), 7.68 (d, *J* = 9.6 Hz, 1H), 7.49 – 7.45 (m, 2H), 7.16 – 7.12 (m, 4H), 7.12 – 7.05 (m, 9H), 6.97 – 6.95 (m, 2H), 6.85 (d, *J* = 2.2 Hz, 1H), 6.81 (dd, *J* = 8.4, 2.2 Hz, 1H), 6.40 (d, *J* = 9.6 Hz, 1H).

^{13}C NMR (151 MHz, $CDCl_3$) δ 191.2, 164.3, 160.2, 154.6, 152.9, 148.1, 144.6, 142.7, 142.5, 141.9, 141.2, 135.4, 135.2, 133.0, 131.7, 131.6, 131.3, 130.9, 130.1, 128.5, 128.01, 127.99, 127.8, 127.7, 127.40, 127.36, 127.1, 118.3, 116.8, 116.2, 110.4.

HRMS: (ESI) m/z: $[M+H]^+$ Calcd for $C_{37}H_{25}O_5^+$ 549.1697; Found 549.1692.

Optical: $[\alpha]_{25}^{D}$ = -0.8 (c = 0.05, CH_2Cl_2, 96% ee).

HPLC (OD-H, ^{i}PrOH/n-hexane = 20/80, flow rate = 0.8 mL/min, 254 nm) t_R = 17.1 min (major), 19.4 min (minor).

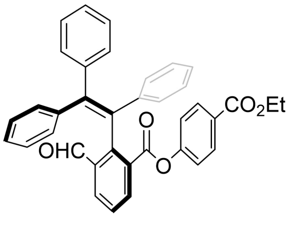

(*R*)-4-(ethoxycarbonyl) phenyl 3-formyl-2-(1,2,2-triphenylvinyl) benzoate (4c) was synthesized by following Procedure B. The crude material was purified by column chromatography (SiO_2, Toluene) to provide **4c** as a yellow solid (28.7 mg, 52% yield).

^{1}H NMR (600 MHz, $CDCl_3$) δ 10.51 (s, 1H), 8.13 (dd, *J* = 7.7, 1.5 Hz, 1H), 8.05 – 8.03 (m, 2H), 7.99 (dd, *J* = 7.8, 1.5 Hz, 1H), 7.46 (td, *J* = 7.8, 0.8 Hz, 1H), 7.16 – 7.13 (m, 3H), 7.12 – 7.08 (m, 5H), 7.07 (s, 5H), 6.97 – 6.95 (m, 2H), 6.93 – 6.90 (m, 2H), 4.37 (d, *J* = 7.1 Hz, 2H), 1.39 (t, *J* = 7.1 Hz, 3H).

^{13}C NMR (151 MHz, $CDCl_3$) δ 191.3, 165.7, 164.4, 154.0, 148.0, 144.5, 142.5, 142.0, 141.3, 135.4, 135.2, 133.1, 132.0, 131.5, 131.3, 131.04, 130.96, 130.1, 128.2, 128.0, 127.7, 127.6, 127.35, 127.31, 127.0, 121.5, 61.1, 14.3.

HRMS: (ESI) m/z: $[M+H]^+$ Calcd for $C_{37}H_{29}O_5^+$ 553.2010; Found 553.2001.

Optical: $[\alpha]_{25}^{D}$ = -7.3 (c = 0.15, CH_2Cl_2, 94% ee).

HPLC (OD-H, ^{i}PrOH/n-hexane = 20/80, flow rate = 0.8 mL/min, 254 nm) t_R = 6.3 min (major), 6.9 min (minor).

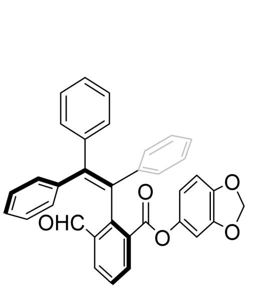

(*R*)-benzo[*d*][1,3]dioxol-5-yl 3-formyl-2-(1,2,2-triphenylvinyl)benzoate (4d) was synthesized by following Procedure B. The crude material was purified by column chromatography (SiO_2, Toluene) to provide **4d** as a yellow solid (35.6 mg, 68%

yield).

^{1}H NMR (600 MHz, $CDCl_3$) δ 10.51 (s, 1H), 8.10 (dd, *J* = 7.7, 1.5 Hz, 1H), 7.97 (dd, *J* = 7.8, 1.5 Hz, 1H), 7.46 – 7.42 (m, 1H), 7.17 – 7.13 (m, 3H), 7.12 – 7.09 (m, 5H), 7.07 (m, 5H), 6.97 (dd, *J* = 8.0, 1.7 Hz, 2H), 6.74 (d, *J* = 8.3 Hz, 1H), 6.31 (dd, *J* = 8.4, 2.3 Hz, 1H), 6.27 (d, *J* = 2.3 Hz, 1H), 5.97 (d, *J* = 1.4 Hz, 2H).

^{13}C NMR (151 MHz, $CDCl_3$) δ 191.5, 165.4, 147.93, 147.88, 145.5, 144.7, 144.4, 142.6, 142.1, 141.3, 135.3, 135.0, 133.2, 132.3, 131.4, 131.2, 131.0, 130.1, 128.0, 127.9, 127.7, 127.5, 127.3, 127.2, 127.0, 113.8, 107.9, 103.6, 101.7.

HRMS: (ESI) m/z: $[M+H]^+$ Calcd for $C_{35}H_{25}O_5^+$ 525.1697; Found 525.1692.

Optical: $[\alpha]_{25}^{D}$ = -22.7 (c = 0.26, CH_2Cl_2, 94% ee).

HPLC (OD-H, ^{i}PrOH/n-hexane = 10/90, flow rate = 0.8 mL/min, 254 nm) t_R = 8.9 min (major), 9.9 min (minor).

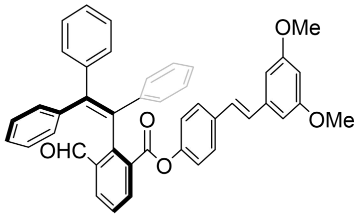

(*R*)-(*E*)-4-(3,5-dimethoxystyryl)phenyl 3-formyl-2-(1,2,2-triphenylvinyl)benzoate (4e) was synthesized by following Procedure B. The crude material was purified by column chromatography (SiO_2, Toluene) to provide **4e** as a yellow solid (52.0 mg, 81% yield).

^{1}H NMR (600 MHz, $CDCl_3$) δ 10.53 (s, 1H), 8.15 (dd, *J* = 7.7, 1.5 Hz, 1H), 7.99 (dd, *J* = 7.8, 1.4 Hz, 1H), 7.49 – 7.44 (m, 3H), 7.15 (dd, *J* = 9.1, 3.7 Hz, 3H), 7.13 – 7.09 (m, 7H), 7.09 – 7.04 (m, 4H), 7.01 – 6.97 (m, 3H), 6.86 (d, *J* = 8.6 Hz, 2H), 6.67 (d, *J* = 2.3 Hz, 2H), 6.42 (t, *J* = 2.2 Hz, 1H), 3.84 (s, 6H).

^{13}C NMR (151 MHz, $CDCl_3$) δ 191.4, 164.9, 160.9, 149.9, 147.8, 144.4, 142.6, 142.0, 141.3, 139.0, 135.4, 135.1, 135.0, 133.3, 132.3, 131.3, 131.2, 131.0, 130.1, 129.0, 128.0, 127.95, 127.92, 127.7, 127.6, 127.4, 127.3, 127.2, 126.9, 121.7, 104.5, 100.0, 55.3.

HRMS: (ESI) m/z: $[M+H]^+$ Calcd for $C_{44}H_{35}O_5^+$ 643.2479; Found 643.2469.

Optical: $[\alpha]_{25}^{D}$ = -9.6 (c = 0.19, CH_2Cl_2, 98% ee).

HPLC (OD-H, ^{i}PrOH/n-hexane = 20/80, flow rate = 0.8 mL/min, 254 nm) t_R = 15.2 min (major), 17.2 min (minor).

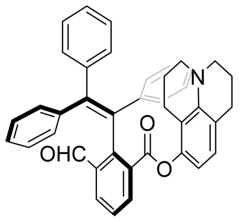

(*R*)-2,3,6,7-tetrahydro-1*H*,5*H*-pyrido[3,2,1-ij]quinolin-8-yl 3-formyl-2-(1,2,2-triphenylvinyl) benzoate (4f) was synthesized by following Procedure B. The crude material was purified by column chromatography (SiO_2, Toluene) to provide **4f** as a yellow solid (18.4 mg, 32% yield).

^{1}H NMR (600 MHz, $(CD_3)_2SO$) δ 10.41 (s, 1H), 8.24 (dd, *J* = 7.7, 1.4 Hz, 1H), 7.89 (dd, *J* = 7.8, 1.4 Hz, 1H), 7.58 (t, *J* = 7.7 Hz, 1H), 7.19 (dd, *J* = 5.2, 1.9 Hz, 3H), 7.10 – 7.04 (m, 8H), 6.98 – 6.96 (m, 2H), 6.90 – 6.87 (m, 2H), 6.69 (d, *J* = 8.1 Hz, 1H), 5.98 (d, *J* = 8.1 Hz, 1H), 3.06 (t, *J* = 5.6 Hz, 2H), 2.98 (m, 2H), 2.64 (t, *J* = 6.5 Hz, 2H), 2.11 (m, 1H), 1.84 (m, 2H), 1.78 (m, 1H), 1.69 – 1.61 (m, 2H).

^{13}C NMR (151 MHz, $(CD_3)_2SO$) δ 191.3, 164.0, 146.84, 146.77, 143.6, 143.0, 142.5, 142.1, 140.7, 135.6, 134.9, 133.3, 131.6, 131.3, 131.0, 130.4, 129.4, 128.3, 128.1, 127.94, 127.89, 127.5, 127.24, 127.21, 126.9, 126.8, 126.4, 118.7, 113.1, 108.4, 79.2, 49.2, 48.6, 40.0, 26.9, 21.4, 20.8, 20.5.

HRMS: (ESI) m/z: $[M+H]^+$ Calcd for $C_{40}H_{34}NO_3^+$ 576.2533; Found 576.2526.

Optical: $[\alpha]_{25}^{D}$ = -6.5 (c = 0.15, CH_2Cl_2, 84% ee).

HPLC (IA, ^{i}PrOH/n-hexane = 20/80, flow rate = 1.0 mL/min, 254 nm) t_R = 5.2 min (major), 9.3 min (minor).

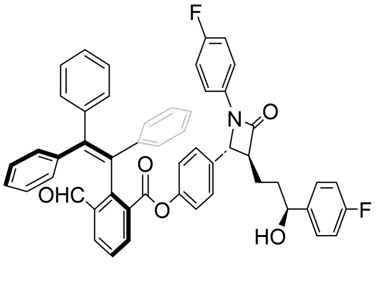

(*R*)-4-((2*R*,3*S*)-1-(4-fluorophenyl)-3-((*S*)-3-(4-fluorophenyl)-3-hydroxypropyl)-4-oxoazetidin-2-yl)phenyl 3-formyl-2-(1,2,2-triphenylvinyl)benzoate (4g) was synthesized by following Procedure B. The crude material was purified by column chromatography (SiO_2, Toluene) to provide **4g** as a yellow solid (61.2 mg, 77% yield), dr values were determined by ^{1}H NMR.

^{1}H NMR (600 MHz, $CDCl_3$) δ 10.50 (s, 1H), 8.11 (dd, *J* = 7.7, 1.5 Hz, 1H), 7.98 (dd, *J* = 7.8, 1.5 Hz, 1H), 7.45 (m, 1H), 7.30 – 7.27 (m, 4H), 7.23 – 7.20 (m, 2H), 7.18 –

7.11 (m, 4H), 7.10 (d, J = 1.4 Hz, 2H), 7.09 (d, J = 2.0 Hz, 2H), 7.06 (t, J = 2.4 Hz, 5H), 7.01 (t, J = 8.7 Hz, 2H), 6.99 – 6.97 (m, 2H), 6.93 (dd, J = 9.1, 8.2 Hz, 2H), 6.84 (d, J = 8.5 Hz, 2H), 4.71 (dd, J = 7.2, 5.1 Hz, 1H), 4.63 (d, J = 2.3 Hz, 1H), 3.10 – 3.06 (m, 1H), 2.01 – 1.87 (m, 4H), 1.78 (s, 1H).

^{13}C NMR (151 MHz, $CDCl_3$) δ 191.3, 167.3, 164.9, 162.1 (d, J = 245.6 Hz), 159.0 (d, J = 243.7 Hz), 150.5, 147.9, 144.5, 142.5, 141.9, 141.3, 140.0 (d, J = 2.9 Hz), 135.3, 135.2, 135.1, 133.6, 133.1, 132.0, 131.4, 131.3, 131.0, 130.1, 127.9 (d, J = 3.5 Hz), 127.7, 127.6, 127.34, 127.32, 127.29, 127.0, 126.8, 122.4, 118.3 (d, J = 7.7 Hz), 115.9 (d, J = 22.5 Hz), 115.3 (d, J = 21.3 Hz), 72.9, 60.7, 60.3, 36.5, 24.9.

^{19}F NMR (565 MHz, $CDCl_3$) δ -114.69, -117.53.

HRMS: (ESI) m/z: $[M+H]^+$ Calcd for $C_{52}H_{40}F_2NO_5^+$ 796.2869; Found 796.2855.

Optical: $[\alpha]_{25}^{D}$ = -8.2 (c = 0.19, CH_2Cl_2, > 20/1 dr).

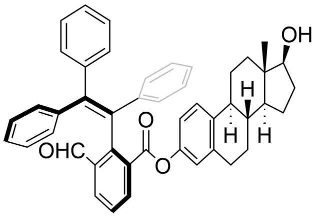

(*R*)-(8*R*,9*S*,13*S*,14*S*,17*S*)-17-hydroxy-13-methyl-7,8,9,11,12,13,14,15,16,17-decahydro-6*H*-cyclopenta[a]phenanthren-3-yl 3-formyl-2-(1,2,2-triphenylvinyl)benzoate (4h) was synthesized by following Procedure B. The crude material was purified by column chromatography (SiO_2, Toluene) to provide **4h** as a yellow solid (34.8 mg, 53% yield), dr values were determined by ^{1}H NMR.

^{1}H NMR (600 MHz, $(CD_3)_2SO$) δ 10.41 (s, 1H), 8.13 (dd, J = 7.7, 1.4 Hz, 1H), 7.88 (dd, J = 7.8, 1.4 Hz, 1H), 7.56 (t, J = 7.7 Hz, 1H), 7.27 (d, J = 8.6 Hz, 1H), 7.17 (d, J = 2.5 Hz, 2H), 7.12 (s, 1H), 7.10 (s, 1H), 7.09 (s, 1H), 7.08 (s, 1H), 7.07 – 7.05 (m, 2H), 7.03 (d, J = 8.5 Hz, 1H), 7.01 – 6.98 (m, 2H), 6.93 – 6.90 (m, 2H), 6.65 (dd, J = 8.4, 2.5 Hz, 1H), 6.51 – 6.48 (m, 2H), 6.43 (d, J = 2.6 Hz, 1H), 2.75 (dd, J = 9.1, 4.4 Hz, 2H), 2.71 – 2.66 (m, 2H), 2.31 – 2.25 (m, 1H), 2.23 – 2.20 (m, 1H), 2.17 – 2.12 (m, 1H), 2.05 (m, 1H), 1.89 – 1.86 (m, 2H), 1.78 – 1.75 (m, 1H), 1.57 (m, 2H), 1.37 (m, 2H), 1.13 – 1.07 (m, 2H), 0.65 (s, 3H).

^{13}C NMR (151 MHz, $(CD_3)_2SO$) δ 191.2, 164.9, 154.9, 147.9, 146.3, 143.2, 142.4, 142.0, 141.0, 137.9, 137.8, 137.1, 135.2, 134.7, 133.3, 132.2, 131.0, 130.5, 130.4, 129.5, 128.13, 128.05, 127.9, 127.6, 127.2, 126.9, 126.2, 126.0, 121.3, 118.5, 114.9, 112.7, 79.2, 49.5, 43.5, 42.8, 38.7, 36.6, 29.9, 29.2, 28.9, 26.9, 26.1, 22.8, 11.3.

HRMS: (ESI) m/z: $[M+H]^+$ Calcd for $C_{46}H_{43}O_4^+$ 659.3156; Found 659.3143.

Optical: $[\alpha]_{25}^{D}$ = -9.4 (c = 0.29, CH_2Cl_2, > 20/1 dr).

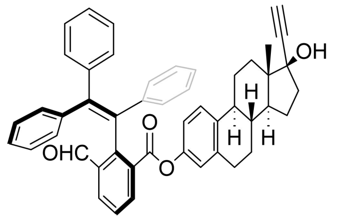

(*R*)-(8*R*,9*S*,13*S*,14*S*,17*R*)-17-ethynyl-17-hydroxy-13-methyl-7,8,9,11,12,13,14,15,16,17-decahydro-6*H*-cyclopenta[a]phenanthren-3-yl 3-formyl-2-(1,2,2-triphenylvinyl)benzoate (4i) was synthesized by following Procedure B. The crude material was purified by column chromatography (SiO_2, Toluene) to provide **4i** as a yellow solid (44.3 mg, 65% yield), dr values were determined by ^{1}H NMR.

^{1}H NMR (400 MHz, $CDCl_3$) δ 10.52 (s, 1H), 8.12 (dd, *J* = 7.7, 1.6 Hz, 1H), 7.97 (dd, *J* = 7.9, 1.6 Hz, 1H), 7.44 (t, *J* = 7.8 Hz, 1H), 7.15 (dd, *J* = 5.3, 2.2 Hz, 3H), 7.12 – 7.08 (m, 7H), 7.08 – 7.04 (m, 3H), 7.03 – 6.97 (m, 2H), 6.65 – 6.54 (m, 2H), 6.50 (d, *J* = 2.6 Hz, 1H), 2.83 – 2.80 (m, 2H), 2.61 (d, *J* = 2.6 Hz, 1H), 2.37 – 2.32 (m, 2H), 2.26 – 2.21 (m, 1H), 2.08 – 2.01 (m, 2H), 1.90 (t, *J* = 6.6 Hz, 2H), 1.79 – 1.69 (m, 4H), 1.44 – 1.40 (m, 2H), 1.27 (s, 1H), 0.89 (s, 3H).

^{13}C NMR (151 MHz, $CDCl_3$) δ 191.6, 165.5, 153.4, 148.1, 144.4, 142.7, 142.1, 141.3, 138.2, 138.1, 135.4, 133.2, 131.4, 131.1, 131.0, 130.1, 127.94, 127.89, 127.6, 127.5, 127.3, 127.2, 126.9, 126.5, 126.4, 121.4, 118.4, 115.2, 112.7, 87.4, 79.8, 74.1, 49.4, 47.0, 43.6, 38.92, 38.88, 32.6, 29.5, 26.9, 26.1, 22.8, 12.6.

HRMS: (ESI) m/z: $[M+H]^+$ Calcd for $C_{48}H_{43}O_4^+$ 683.3156; Found 683.3144.

Optical: $[\alpha]_{25}^{D}$ = -5.2 (c = 0.18, CH_2Cl_2, > 20/1 dr).

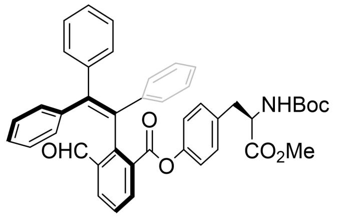

(*R*)-(*R*)-4-(2-((tert-butoxycarbonyl)amino)-3-methoxy-3-oxopropyl)phenyl 3-formyl-2-(1,2,2-triphenylvinyl)benzoate (4j) was synthesized by following Procedure B. The crude material was purified by column chromatography (SiO_2, Toluene) to provide **4j** as a yellow solid (44.3 mg, 65% yield), dr values were determined by ^{1}H NMR.

^{1}H NMR (400 MHz, $CDCl_3$) δ 10.51 (s, 1H), 8.11 (dd, *J* = 7.7, 1.5 Hz, 1H), 7.97 (dd, *J* = 7.8, 1.5 Hz, 1H), 7.44 (t, *J* = 7.7 Hz, 1H), 7.16 – 7.05 (m, 16H), 6.99 – 6.95 (m, 2H), 6.79 (d, *J* = 8.5 Hz, 2H), 4.57 (d, *J* = 7.5 Hz, 1H), 3.70 (s, 3H), 3.07 (m, 2H), 1.42 (s, 9H).

^{13}C NMR (101 MHz, $CDCl_3$) δ 191.4, 172.2, 164.9, 149.5, 147.8, 144.4, 142.6, 142.1, 141.3, 135.4, 135.1, 133.9, 133.3, 132.4, 131.3, 131.2, 131.0, 130.2, 130.1, 127.94, 127.92, 127.7, 127.6, 127.3, 127.2, 126.9, 121.5, 54.3, 52.2, 37.7, 28.3.

HRMS: (ESI) m/z: $[M+Na]^+$ Calcd for $C_{43}H_{40}NO_7^+$ 704.2619; Found 704.2610.

Optical: $[\alpha]_{25}^{D}$ = -10.0 (c = 0.23, CH_2Cl_2, > 20/1 dr).

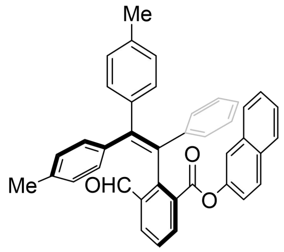

(*R*)-naphthalen-2-yl 3-formyl-2-(1-phenyl-2,2-di-p-tolylvinyl)benzoate (5a) was synthesized by following Procedure B. The crude material was purified by column chromatography (SiO_2, Toluene) to provide **5a** as a yellow solid (39.6 mg, 71% yield).

^{1}H NMR (600 MHz, $CDCl_3$) δ 10.52 (s, 1H), 8.19 (dd, *J* = 7.6, 1.5 Hz, 1H), 8.00 (dd, *J* = 7.8, 1.5 Hz, 1H), 7.84 – 7.80 (m, 2H), 7.76 (dd, *J* = 7.9, 1.5 Hz, 1H), 7.48 (m, 3H), 7.30 (d, *J* = 2.4 Hz, 1H), 7.16 – 7.11 (m, 3H), 7.01 – 6.98 (m, 5H), 6.98 – 6.95 (m, 2H), 6.94 – 6.92 (m, 2H), 6.89 – 6.87 (m, 2H), 2.26 (s, 3H), 2.21 (s, 3H).

^{13}C NMR (151 MHz, $CDCl_3$) δ 191.7, 165.3, 148.3, 148.1, 144.7, 141.8, 139.9, 139.3, 136.99, 136.96, 135.4, 135.1, 133.6, 132.5, 132.2, 131.5, 131.4, 131.2, 130.9, 130.2, 129.3, 128.7, 128.6, 127.73, 127.70, 127.6, 127.4, 126.7, 126.5, 125.8, 120.9, 118.6, 21.2.

HRMS: (ESI) m/z: $[M+H]^+$ Calcd for $C_{40}H_{31}O_3^+$ 559.2268; Found 559.2262.

Optical: $[\alpha]_{25}^{D}$ = -26.0 (c = 0.26, CH_2Cl_2, 94% ee).

HPLC (IA, ^{i}PrOH/n-hexane = 20/80, flow rate = 0.8 mL/min, 254 nm) t_R = 15.2 min (major), 32.8 min (minor).

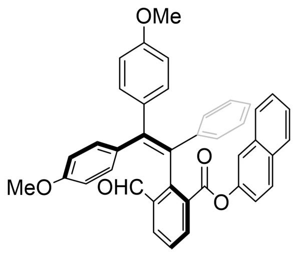

(*R*)-naphthalen-2-yl2-(2,2-bis(4-methoxyphenyl)-1-phenylvinyl)-3-formylbenzoate) (5b) was synthesized by following Procedure B. The crude material was purified by column chromatography (SiO_2, Toluene) to provide **5b** as a yellow solid (47.8 mg, 81% yield).

^{1}H NMR (400 MHz, $CDCl_3$) δ 10.50 (s, 1H), 8.18 (dd, *J* = 7.7, 1.5 Hz, 1H), 8.00 (dd, *J* = 7.8, 1.5 Hz, 1H), 7.85 – 7.74 (m, 4H), 7.49 – 7.47 (m, 2H), 7.28 (d, *J* = 2.4 Hz, 1H), 7.15 – 7.13 (m, 2H), 7.07 (s, 1H), 7.05 (s, 1H), 7.03 – 6.99 (m, 5H), 6.93 (dd, *J* = 8.9, 2.3 Hz, 1H), 6.66 (d, *J* = 8.8 Hz, 2H), 6.63 (s, 1H), 6.61 (s, 1H), 3.74 (s, 3H), 3.70 (s, 3H).

^{13}C NMR (101 MHz, $CDCl_3$) δ 191.8, 165.5, 158.7, 158.6, 148.5, 148.1, 144.1, 142.0, 135.34, 135.28, 135.1, 134.7, 133.6, 132.6, 132.4, 131.7, 131.6, 131.5, 131.4, 131.2, 129.3, 127.8, 127.7, 127.5, 127.3, 126.6, 126.5, 125.7, 120.9, 118.5, 113.32, 113.25, 55.1, 55.0.

HRMS: (ESI) m/z: $[M+H]^+$ Calcd for $C_{40}H_{31}O_5^+$ 591.2166; Found 591.2160.

Optical: $[\alpha]_{25}^{D}$ = -16.8 (c = 0.22, CH_2Cl_2, 97% ee).

HPLC (IC, ^{i}PrOH/n-hexane = 20/80, flow rate = 0.8 mL/min, 254 nm) t_R = 9.5 min (major), 8.5 min (minor).

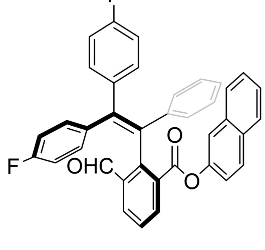

(*R*)-naphthalen-2-yl 2-(2,2-bis(4-fluorophenyl)-1-phenylvinyl)-3-formylbenzoate (5c) was synthesized by following Procedure B. The crude material was purified by column chromatography (SiO_2, Toluene) to provide **5c** as a yellow solid (39.6 mg, 70% yield).

^{1}H NMR (600 MHz, $CDCl_3$) δ 10.47 (s, 1H), 8.20 (dd, *J* = 7.7, 1.5 Hz, 1H), 8.01 (dd, *J* = 7.8, 1.5 Hz, 1H), 7.83 (m, 2H), 7.77 – 7.74 (m, 1H), 7.52 – 7.47 (m, 3H), 7.21 – 7.02 (m, 8H), 6.99 – 6.97 (m, 2H), 6.91 (dd, *J* = 8.8, 2.3 Hz, 1H), 6.84 – 6.81 (m, 2H),

6.80 – 6.76 (m, 2H).

^{13}C NMR (151 MHz, $CDCl_3$) δ 191.2, 165.3, 161.9 (d, *J* = 247.9 Hz), 161.8 (d, *J* = 248.3 Hz), 148.0, 147.5, 142.3, 141.0, 138.5 (d, *J* = 3.2 Hz), 137.8 (d, *J* = 3.4 Hz), 135.4, 135.0, 133.8, 133.6, 132.7 (d, *J* = 8.0 Hz), 132.4, 131.9 (d, *J* = 8.1 Hz), 131.5, 131.4, 129.4, 127.9, 127.81, 127.77, 127.6, 127.2, 126.7, 125.9, 120.8, 118.5, 115.2 (d, *J* = 21.3 Hz), 115.1 (d, *J* = 21.4 Hz).

^{19}F NMR (376 MHz, $CDCl_3$) δ -113.32, -113.70.

HRMS: (ESI) m/z: $[M+H]^+$ Calcd for $C_{38}H_{25}F_2O_3^+$ 567.1766; Found 567.1761.

Optical: $[\alpha]_{25}^{D}$ = -9.5 (c = 0.19, CH_2Cl_2, 97% ee).

HPLC (IA, ^{i}PrOH/n-hexane = 20/80, flow rate = 0.8 mL/min, 254 nm) t_R = 7.7 min (major), 10.0 min (minor).

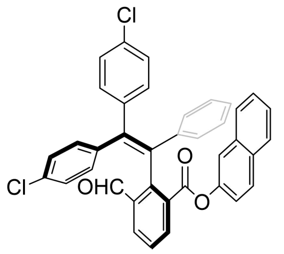

(*R*)-naphthalen-2-yl 2-(2,2-bis(4-chlorophenyl)-1-phenylvinyl)-3-formylbenzoate (5d) was synthesized by following Procedure B. The crude material was purified by column chromatography (SiO_2, Toluene) to provide **5d** as a yellow solid (41.8 mg, 70% yield).

^{1}H NMR (600 MHz, $CDCl_3$) δ 10.45 (s, 1H), 8.22 (dd, *J* = 7.7, 1.5 Hz, 1H), 8.02 (dd, *J* = 7.8, 1.5 Hz, 1H), 7.83 (m, 2H), 7.78 – 7.73 (m, 1H), 7.55 – 7.45 (m, 3H), 7.22 – 7.08 (m, 5H), 7.08 – 7.04 (m, 2H), 7.03 (d, *J* = 8.6 Hz, 2H), 6.99 (m, 5H), 6.91 (dd, *J* = 8.8, 2.3 Hz, 1H).

^{13}C NMR (151 MHz, $CDCl_3$) δ 191.0, 165.2, 147.9, 147.1, 141.8, 140.8, 140.7, 140.1, 135.4, 134.9, 134.6, 133.6, 133.42, 133.36, 132.30, 132.28, 131.7, 131.5, 131.4, 131.3, 129.5, 128.4, 128.3, 128.0, 127.8, 127.5, 127.4, 126.7, 125.9, 120.7, 118.5.

HRMS: (ESI) m/z: $[M+H]^+$ Calcd for $C_{38}H_{25}Cl_2O_3^+$ 599.1175; Found 599.1169.

Optical: $[\alpha]_{25}^{D}$ = -23.1 (c = 0.27, CH_2Cl_2, 97% ee).

HPLC (IA, ^{i}PrOH/n-hexane = 20/80, flow rate = 1.0 mL/min, 254 nm) t_R = 6.4 min (major), 8.1 min (minor).

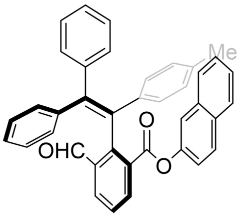

(*R*)-naphthalen-2-yl 2-(2,2-diphenyl-1-(p-tolyl)vinyl)-3-formylbenzoate (5e) was synthesized by following Procedure B. The crude material was purified by column chromatography (SiO_2, Toluene) to provide **5e** as a yellow solid (35.3 mg, 65% yield).

^{1}H NMR (400 MHz, $CDCl_3$) δ 10.54 (s, 1H), 8.18 (dd, *J* = 7.7, 1.5 Hz, 1H), 8.00 (dd, *J* = 7.8, 1.5 Hz, 1H), 7.86 – 7.81 (m, 2H), 7.77 – 7.73 (m, 1H), 7.50 – 7.44 (m, 3H), 7.31 (d, *J* = 2.4 Hz, 1H), 7.16 – 7.11 (m, 7H), 7.08 (m, 3H), 6.98 (dd, *J* = 8.9, 2.4 Hz, 1H), 6.93 (d, *J* = 8.2 Hz, 2H), 6.89 (d, *J* = 8.3 Hz, 2H), 2.31 (s, 3H).

^{13}C NMR (101 MHz, $CDCl_3$) δ 191.6, 165.3, 148.11, 148.07, 143.9, 142.8, 142.3, 138.6, 136.7, 135.3, 135.1, 133.6, 133.2, 132.5, 131.5, 131.3, 131.1, 131.0, 130.2, 129.3, 128.5, 128.0, 127.7, 127.55, 127.51, 127.2, 127.1, 126.6, 125.8, 120.9, 118.6, 21.2.

HRMS: (ESI) m/z: $[M+H]^+$ Calcd for $C_{39}H_{29}O_3^+$ 545.2111; Found 545.2106.

Optical: $[\alpha]_{25}^{D}$ = -6.4 (c = 0.15, CH_2Cl_2, 96% ee).

HPLC (OD-H, ^{i}PrOH/n-hexane = 3/97, flow rate = 0.8 mL/min, 254 nm) t_R = 10.8 min (major), 9.7 min (minor).

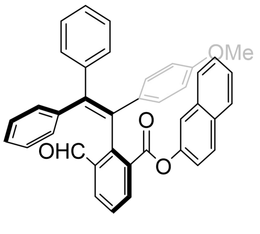

(*R*)-naphthalen-2-yl3-formyl-2-(1-(4-methoxyphenyl)-2,2-diphenylvinyl)benzoate (5f) was synthesized by following Procedure B. The crude material was purified by column chromatography (SiO_2, Toluene) to provide **5f** as a yellow solid (36.9 mg, 66% yield).

^{1}H NMR (400 MHz, $CDCl_3$) δ 10.53 (s, 1H), 8.18 (dd, *J* = 7.7, 1.5 Hz, 1H), 7.99 (dd, *J* = 7.8, 1.5 Hz, 1H), 7.83 (dd, *J* = 8.9, 6.4 Hz, 2H), 7.77 – 7.75 (m, 1H), 7.52 – 7.48 (m, 2H), 7.46 (d, *J* = 6.7 Hz, 1H), 7.32 (d, *J* = 2.3 Hz, 1H), 7.15 – 7.11 (m, 7H), 7.09 – 7.06 (m, 3H), 7.01 (dd, *J* = 8.8, 2.3 Hz, 1H), 6.92 (d, *J* = 8.8 Hz, 2H), 6.66 (d, *J* = 8.9 Hz, 2H), 3.76 (s, 3H).

^{13}C NMR (101 MHz, $CDCl_3$) δ 191.6, 165.3, 158.4, 148.1, 143.4, 142.8, 142.3, 135.3, 135.1, 134.0, 133.6, 132.9, 132.7, 132.5, 131.5, 131.1, 131.0, 130.2, 129.3, 128.00, 127.95, 127.7, 127.6, 127.5, 127.14, 127.08, 126.6, 125.8, 121.0, 120.9, 118.6, 113.1, 55.2.

HRMS: (ESI) m/z: $[M+H]^+$ Calcd for $C_{39}H_{29}O_4^+$ 561.2060; Found 561.2054.

Optical: $[\alpha]_{25}^{D}$ = -5.5 (c = 0.14, CH_2Cl_2, 96% ee).

HPLC (OD-H, ^{i}PrOH/n-hexane = 5/95, flow rate = 0.8 mL/min, 254 nm) t_R = 14.6 min (major), 13.8 min (minor).

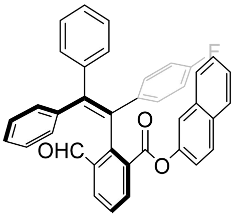

(*R*)-naphthalen-2-yl 2-(1-(4-fluorophenyl)-2,2-diphenylvinyl)-3-formylbenzoate (5g) was synthesized by following Procedure B. The crude material was purified by column chromatography (SiO_2, Toluene) to provide **5g** as a yellow solid (35.0 mg, 64% yield).

^{1}H NMR (400 MHz, $CDCl_3$) δ 10.53 (s, 1H), 8.23 (d, *J* = 7.6 Hz, 1H), 8.03 – 8.00 (m, 1H), 7.85 (d, *J* = 8.8 Hz, 2H), 7.78 (d, *J* = 7.8 Hz, 1H), 7.53 – 7.46 (m, 3H), 7.35 (d, *J* = 2.2 Hz, 1H), 7.17 – 7.14 (m, 3H), 7.13 – 7.09 (m, 7H), 7.04 – 6.97 (m, 3H), 6.82 (m, 2H).

^{13}C NMR (101 MHz, $CDCl_3$) δ 191.2, 165.2, 161.5 (d, *J* = 248.0 Hz), 148.0, 147.6, 144.5, 142.4, 141.9, 137.5, 135.5, 135.0, 133.6, 133.1, 133.0, 132.2 (d, *J* = 6.2 Hz), 131.5, 130.9, 130.1, 129.5, 128.1, 128.0, 127.8, 127.6, 127.4, 127.3, 126.7, 125.9, 120.7, 118.5, 114.7 (d, *J* = 21.4 Hz), 77.2.

^{19}F NMR (376 MHz, $CDCl_3$) δ -114.00.

HRMS: (ESI) m/z: $[M+H]^+$ Calcd for $C_{38}H_{26}FO_3^+$ 549.1860; Found 549.1853.

Optical: $[\alpha]_{25}^{D}$ = -16.9 (c = 0.26, CH_2Cl_2, 92% ee).

HPLC (OD-H, ^{i}PrOH/n-hexane = 20/80, flow rate = 0.8 mL/min, 254 nm) t_R = 9.5 min (major), 16.7 min (minor).

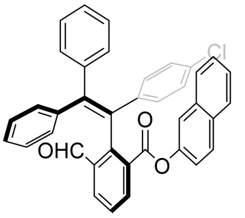

(*R*)-naphthalen-2-yl 2-(1-(4-chlorophenyl)-2,2-diphenylvinyl)-3-formylbenzoate (5h) was synthesized by following Procedure B. The crude material was purified by column chromatography (SiO_2, Toluene) to provide **5h** as a yellow solid (30.4 mg, 54% yield).

^{1}H NMR (400 MHz, $CDCl_3$) δ 10.51 (s, 1H), 8.22 (dd, *J* = 7.7, 1.5 Hz, 1H), 8.02 – 8.00 (m, 1H), 7.85 (d, *J* = 8.9 Hz, 2H), 7.52 – 7.48 (m, 3H), 7.29 (d, *J* = 2.4 Hz, 1H), 7.16 (dd, *J* = 7.0, 1.3 Hz, 3H), 7.13 – 7.11 (m, 2H), 7.10 (d, *J* = 3.6 Hz, 6H), 7.09 (s, 1H), 7.01 (dd, *J* = 8.9, 2.4 Hz, 2H), 6.94 (d, *J* = 8.6 Hz, 2H).

^{13}C NMR (101 MHz, $CDCl_3$) δ 191.1, 165.2, 148.0, 147.3, 145.0, 142.3, 141.7, 139.9, 135.5, 135.1, 133.6, 132.8, 132.7, 132.3, 132.1, 131.5, 131.4, 131.2, 131.0, 130.9, 130.2, 130.1, 129.5, 128.1, 128.02, 127.95, 127.9, 127.8, 127.7, 127.6, 127.5, 126.7, 125.9, 120.7, 118.5, 77.2.

HRMS: (ESI) m/z: $[M+H]^+$ Calcd for $C_{38}H_{26}ClO_3^+$ 565.1565; Found 565.1557.

Optical: $[\alpha]_{25}^{D}$ = -16.0 (c = 0.26, CH_2Cl_2, 93% ee).

HPLC (IA, ^{i}PrOH/n-hexane = 20/80, flow rate = 0.8 mL/min, 254 nm) t_R = 8.8 min (major), 17.8 min (minor).

4. Reaction of 2-Aminobenzothiazole and Control Experiments

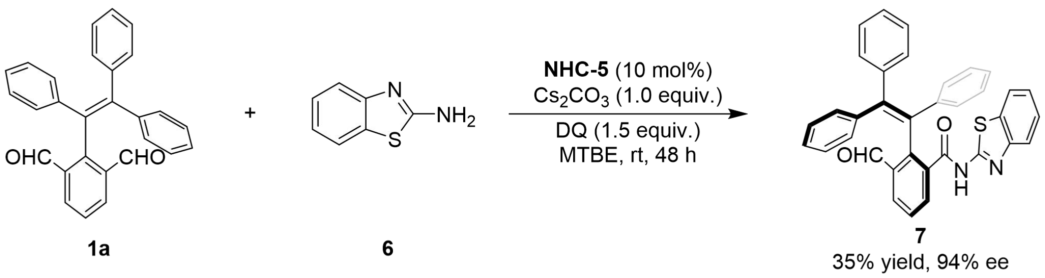

1a **6** **7**

35% yield, 94% ee

TAE dialdehyde **1a** (38.8 mg, 0.1 mmol, 1.0 equiv.), 2-aminobenzothiazole **6** (22.5 mg, 0.15 mmol, 1.5 equiv.), **NHC-5** (5.0 mg, 0.01 mmol, 10 mol%), Cs_2CO_3 (48.8 mg, 0.15 mmol, 1.5 equiv.), and DQ (57.2 mg, 0.15 mmol, 1.5 equiv.) were placed in the reaction tube under the N_2 atmosphere, and anhydrous MTBE (1.5 mL) was added to the reaction mixture via a syringe, and stir at room temperature for 48 h. After the reaction is complete, the crude mixture is purified by column chromatography (PE/DCM = 2:1) to provide **7** as a brown solid (18.7 mg, 35% yield). **^{1}H NMR** (600 MHz, $CDCl_3$) δ 10.50 (s, 1H), 7.80 – 7.77 (m, 2H), 7.40 (dd, J = 7.6, 1.4 Hz, 1H), 7.26 – 7.23 (m, 1H), 7.17 – 7.15 (m, 6H), 7.12 – 7.10 (m, 2H), 7.06 – 7.03 (m, 4H), 7.02 – 6.95 (m, 5H), 6.95 – 6.92 (m, 1H), 6.86 (d, J = 8.1 Hz, 1H). **^{13}C NMR** (151 MHz, $CDCl_3$) δ 191.3, 166.4, 158.9, 147.4, 146.9, 145.0, 142.3, 142.0, 141.6, 135.3, 134.8, 133.0, 132.1, 131.4, 131.2, 131.1, 130.4, 130.2, 127.9, 127.84, 127.76, 127.6, 127.4, 127.2, 127.0, 125.8, 124.0, 121.2, 120.7. **HRMS:** (ESI) m/z: $[M+H]^+$ Calcd for $C_{35}H_{25}N_2O_2S^+$ 537.1631; Found 537.1628. **Optical:** $[\alpha]_{25}^{D}$ = -0.7 (c = 0.15, CH_2Cl_2, 94% ee). **HPLC** (AD-H, ^{i}PrOH/n-hexane = 20/80, flow rate = 0.8 mL/min, 254 nm) t_R = 9.7 min (major), 14.1 min (minor).

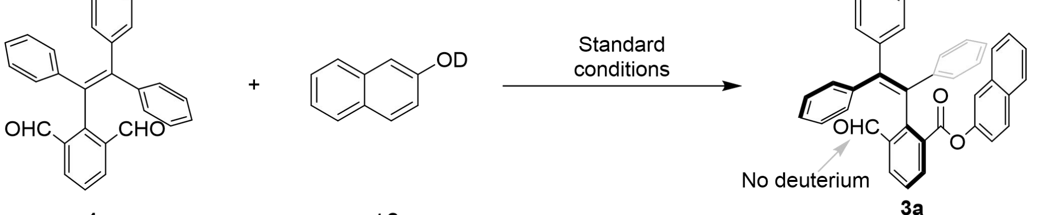

1a *d*-**2a** **3a**

71% yield, 98% ee

An oven dried flask (20 mL) was rinsed with MeOH-*d₄* twice, and then charged with **2a** (144.1 mg, 1 mmol). MeOH-d_4 (99.8% D, 1 mL) was added and the mixture was concentrated using rotatory evaporator (this process was repeated for six times).

The residual solvent was evaporated with high vacuum and resulted in quantitative (> 99%) yield of the title compound *d*-**2a**.

TAE dialdehyde **1a** (38.8 mg, 0.1 mmol, 1 equiv.), *d*-**2a** (21.7 mg, 0.15 mmol, 1.5 equiv.), **NHC-5** (5.0 mg, 0.01 mmol, 10 mol%), Cs_2CO_3 (48.8 mg, 1.5 mmol, 1.5 equiv.), and DQ (57.2 g, 0.15 mmol, 1.5 equiv.) were placed in the reaction tube under the N_2 atmosphere, and anhydrous MTBE (1.5 mL) was added to the reaction mixture via a syringe, and stir at room temperature for 48 h. After completion of the reaction, the crude mixture was purified by column chromatography on silica gel to afford the corresponding product **3a** (37.6 mg, 71% yield, 98% ee). **^{1}H NMR** (400 MHz, $CDCl_3$) δ 10.56 (s, 1H), 8.21 (d, *J* = 7.7 Hz, 1H), 8.01 (d, *J* = 7.8 Hz, 1H), 7.83 (t, *J* = 8.2 Hz, 2H), 7.79 – 7.74 (m, 1H), 7.52 – 7.45 (m, 3H), 7.32 (d, *J* = 2.3 Hz, 1H), 7.19 – 7.11 (m, 10H), 7.11 – 7.08 (m, 3H), 7.02 (d, *J* = 7.0 Hz, 2H), 6.97 (dd, *J* = 8.9, 2.3 Hz, 1H).

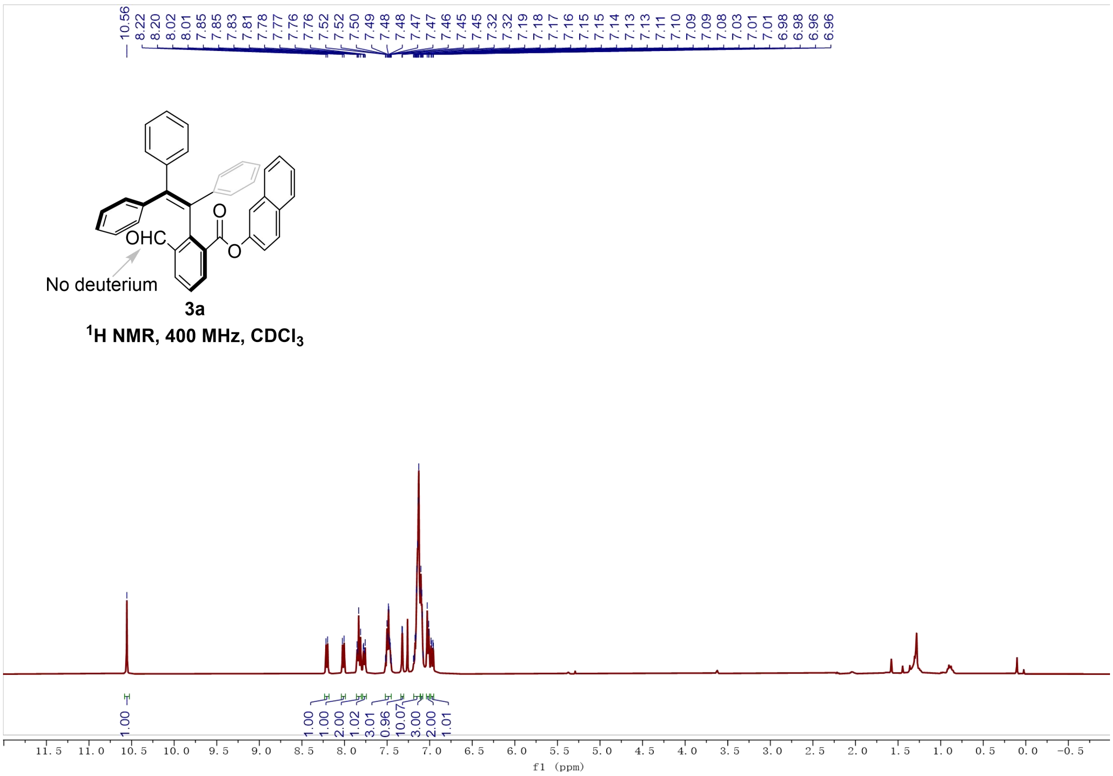

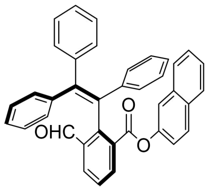

3a

HPLC conditions: Chiralpak OD, 20% ^{i}PrOH/Hx eluent, 0.6 mL/min, 254 nm

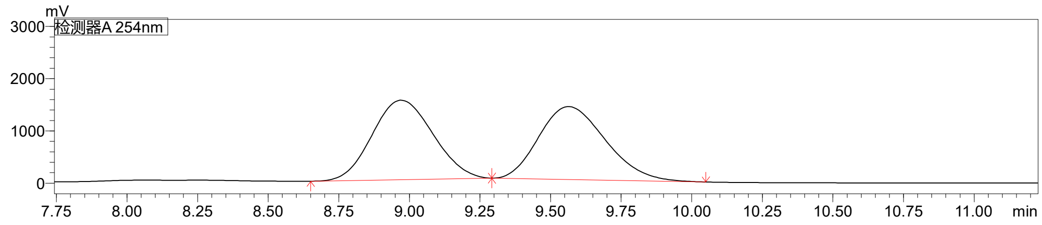

Peak#	Ret. Time	Height	Area%
1	8.970	1523471	49.911
2	9.563	1398257	50.089

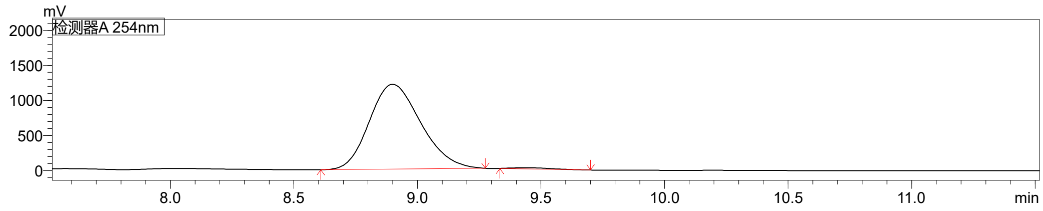

Peak#	Ret. Time	Height	Area%
1	8.899	1210371	99.011
2	9.435	16595	0.989

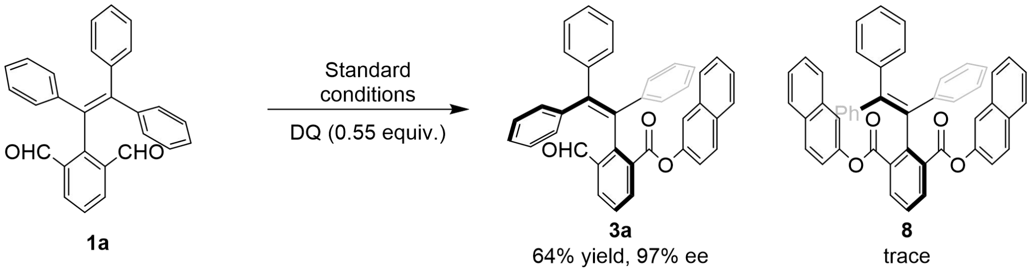

TAE dialdehyde **1a** (380 mg, 0.1 mmol, 1 equiv.), **2a** (21.7 mg, 0.15 mmol, 1.5 equiv.), **NHC-5** (5.0 mg, 0.01 mmol, 10 mol%), Cs_2CO_3 (48.8 mg, 1.5 mmol, 1.5 equiv.), and DQ (22.5 mg, 0.055 mmol, 0.55 equiv.) were placed in the reaction tube under the N_2 atmosphere, and anhydrous MTBE (1.5 mL) was added to the reaction mixture via a syringe, and stir at room temperature for 48 h. After completion of the

reaction, the crude mixture was purified by column chromatography on silica gel to afford the corresponding product **3a** (33.9 mg, 64% yield, 97% ee) and **8** (<5%).

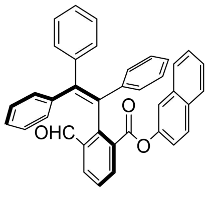

3a

HPLC conditions: Chiralpak OD, 20% ^{i}PrOH/Hx eluent, 0.6 mL/min, 254 nm

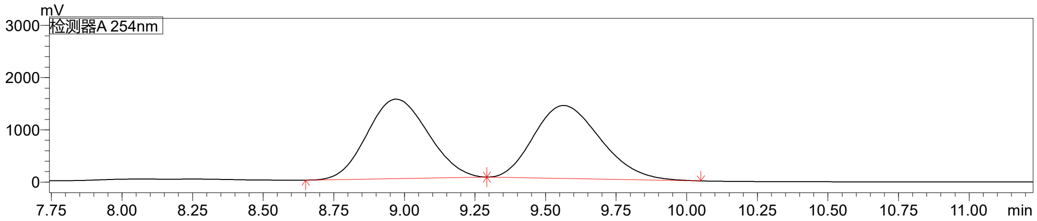

Peak#	Ret. Time	Height	Area%
1	8.970	1523471	49.911
2	9.563	1398257	50.089

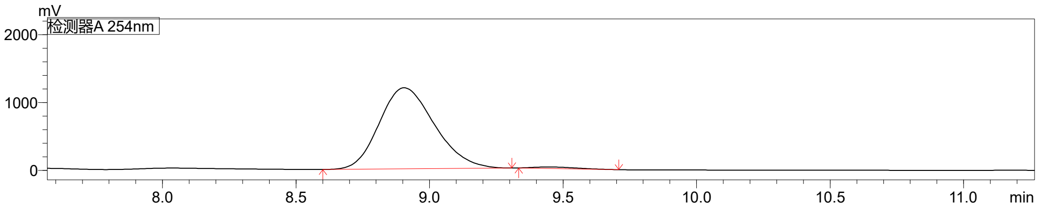

Peak#	Ret. Time	Height	Area%
1	8.905	1194856	98.609
2	9.447	22312	1.391

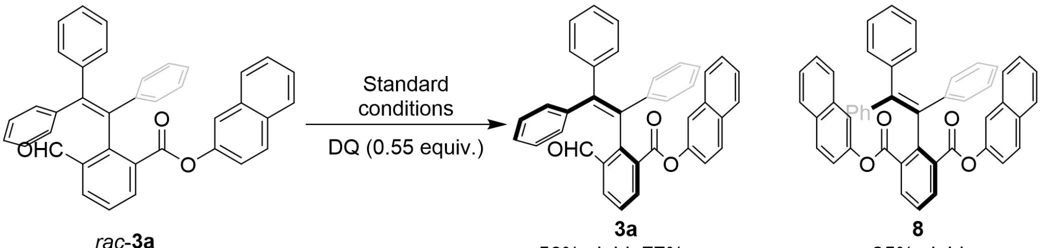

TAE dialdehyde *rac*-**3a** (38.8 mg, 0.1 mmol, 1 equiv.), **2a** (21.7 mg, 0.15 mmol, 1.5 equiv.), **NHC-5** (5.0 mg, 0.01 mmol, 10 mol%), Cs_2CO_3 (48.8 mg, 1.5 mmol, 1.5 equiv.), and DQ (22.5 mg, 0.055 mmol, 0.55 equiv.) were placed in the reaction tube under the N_2 atmosphere, and anhydrous MTBE (1.5 mL) was added to the reaction

mixture via a syringe, and stir at room temperature for 48 h. After completion of the reaction, the crude mixture was purified by column chromatography on silica gel to afford the corresponding product **3a** (29.6 mg, 56% yield, 77% ee) and **8** (16.8 mg, 25% yield). **^{1}H NMR** (400 MHz, $CDCl_3$) δ 8.10 (d, *J* = 7.8 Hz, 2H), 7.84 (dd, *J* = 9.1, 4.2 Hz, 4H), 7.78 (dd, *J* = 7.1, 2.2 Hz, 2H), 7.52 – 7.45 (m, 5H), 7.43 (d, *J* = 2.3 Hz, 2H), 7.25 – 7.22 (m, 2H), 7.16 – 7.09 (m, 11H), 7.08 – 7.03 (m, 4H). **^{13}C NMR** (101 MHz, $CDCl_3$) δ 165.5, 148.3, 143.0, 133.7, 133.45, 133.41, 132.2, 131.5, 131.3, 130.5, 129.3, 127.8, 127.7, 127.64, 127.61, 127.3, 127.0, 126.7, 126.53, 126.47, 125.7, 121.0, 118.6.

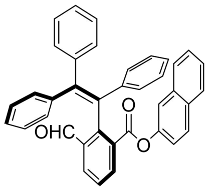

3a

HPLC conditions: Chiralpak OD, 20% ^{i}PrOH/Hx eluent, 0.6 mL/min, 254 nm

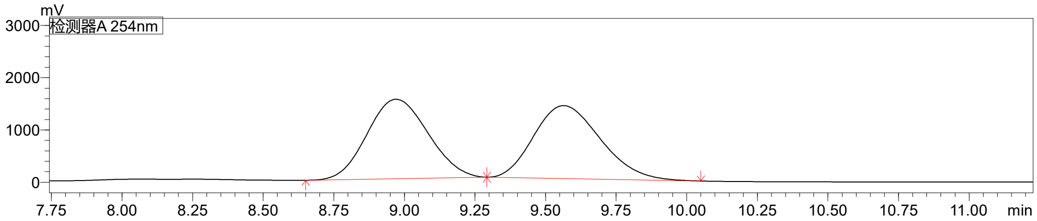

Peak#	Ret. Time	Height	Area%
1	8.970	1523471	49.911
2	9.563	1398257	50.089

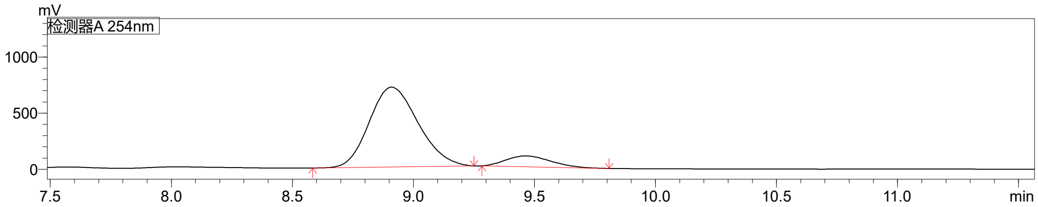

Peak#	Ret. Time	Height	Area%
1	8.910	711680	88.983
2	9.463	96166	11.017

Non-linear effect studies

The reaction was carried on for several times with the mixture of **NHC-1** and **ent-NHC-1** in different proportions. The ee value of **3a** was determined on a chiral HPLC analysis.

Table S1. Ee of **3a** using ligand **NHC-1** with varying ee

ee of **L1**	ee of **3a**
0	0
20	20
40	37
60	57
80	70
99	87

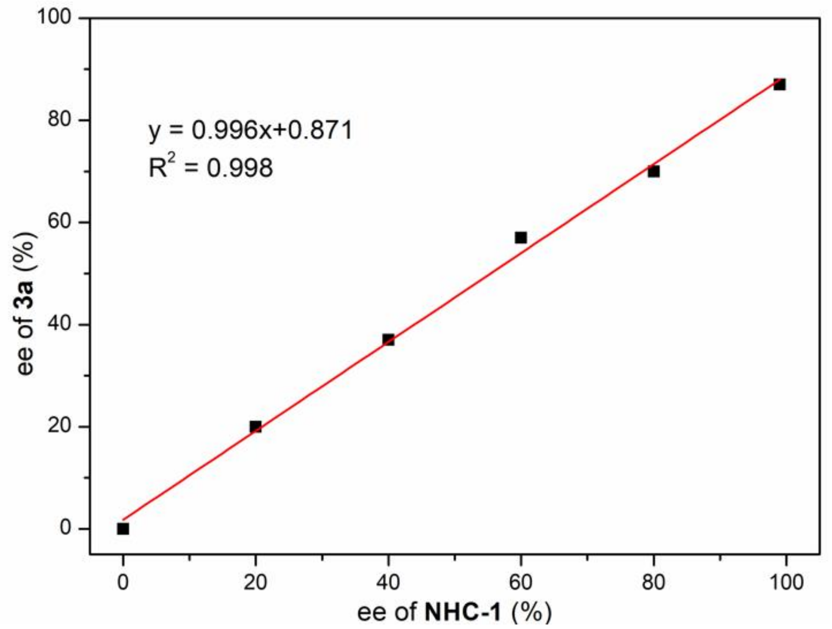

Fig. S1. Non-linear study between **3a** and **NHC-1**

5. Synthetic Transformations

Table S2. Racemization of **3a** in toluene at 120 °C

Time (min)	ee (%)	$\ln(e_0/e)$
0	97	0
30	82	0.168
60	70	0.3262
120	47	0.7246
180	37	0.9638
240	25	1.3558
300	18	1.6843

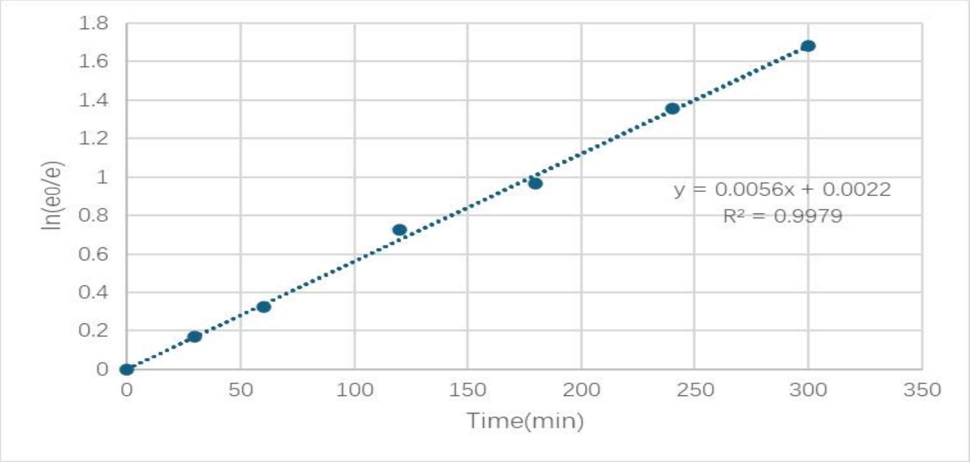

Kracemisation = 0.0056 h^{-1}

Kenantiomerisation = 0.0028 h^{-1}

$$\Delta G^{\ddagger}\text{enaniomerization} = RT \times \ln \frac{k_B \times T}{h \times k_{enantiomerisation}}$$

$\Delta G^{\ddagger}$enaniomerization=129.79 KJ/mol=31.0 Kcal/mol

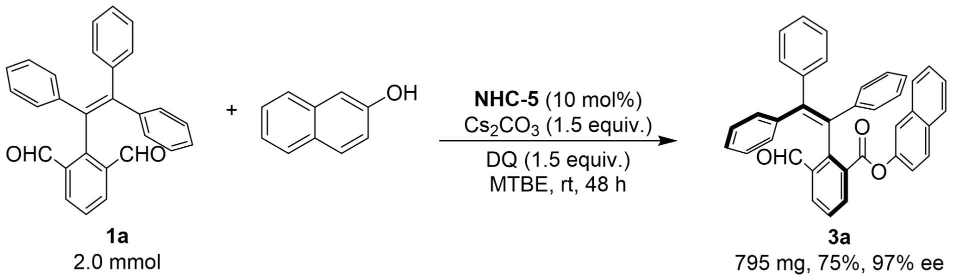

TAE dialdehyde **1a** (776.9 mg, 2.0 mmol, 1 equiv.), 2-naphthol **2a** (432.5 mg, 3.0 mmol, 1.5 equiv.), **NHC-5** (100.0 mg, 0.2 mmol, 10 mol%), Cs_2CO_3 (977.4 mg, 3.0 mmol, 1.5 equiv.), and DQ (1.22 g, 3.0 mmol, 1.5 equiv.) were placed in the reaction tube under the N_2 atmosphere, and anhydrous MTBE (30 mL) was added to the reaction mixture via a syringe, and stir at room temperature for 48 h. After completion of the reaction, the crude mixture was purified by column chromatography on silica gel to afford the corresponding product **3a** (795 mg, 75% yield, 97% ee).

HPLC conditions: Chiralpak OD, 20% ^{i}PrOH/Hx eluent, 0.6 mL/min, 254 nm

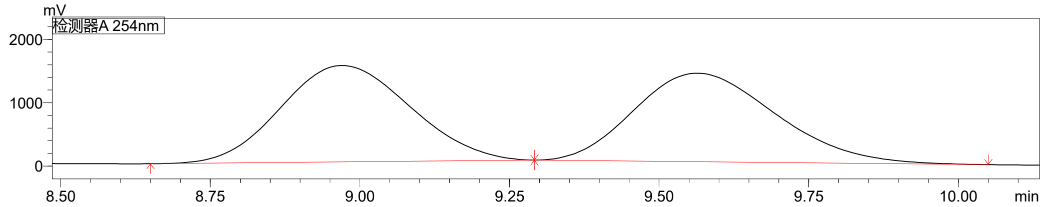

Peak#	Ret. Time	Height	Area%
1	8.970	1523471	49.911
2	9.563	1398257	50.089

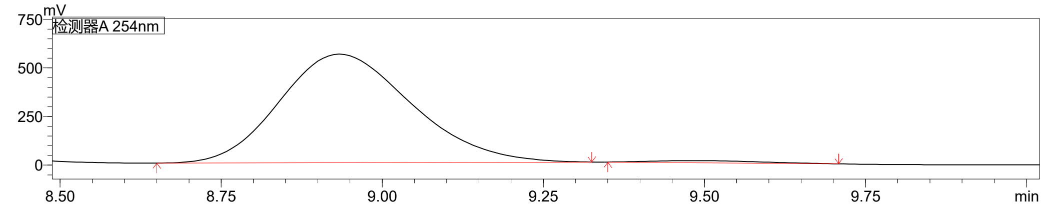

Peak#	Ret. Time	Height	Area%
1	8.933	558068	98.466
2	9.484	10865	1.534

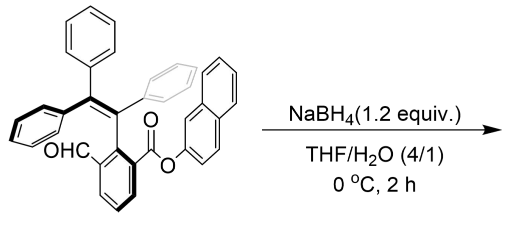

3a **9**

To a solution of **3a** (53.0 mg, 0.10 mmol, 1.0 equiv.) in THF/H_2O (4:1 v/v ,10.0 mL) was added $NaBH_4$ (4.5 mg, 0.12 mmol, 1.2 equiv.) under air. The mixture was stirred for another 2 h and quenched with saturated aqueous NH_4Cl and extracted with ethyl acetate. The organic layer was combined, dried over anhydrous Na_2SO_4 and concentrated. The crude product was purified by silica gel column chromatography (PE/EA = 5:1) to afford **9** as colorless solid (41.0 mg, 77%, 95% ee). **^{1}H NMR** (400 MHz, $CDCl_3$) δ 7.92 (dd, *J* = 7.8, 1.4 Hz, 1H), 7.83 (dd, *J* = 8.9, 6.2 Hz, 2H), 7.79 – 7.73 (m, 1H), 7.69 (dd, *J* = 7.8, 1.4 Hz, 1H), 7.51 – 7.43 (m, 2H), 7.43 – 7.39 (m, 2H), 7.12 (m, 10H), 7.10 (dd, *J* = 6.9, 1.3 Hz, 3H), 7.08 – 7.02 (m, 4H), 4.79 (m, 1H), 4.69 (m, 1H). **^{13}C NMR** (101 MHz, $CDCl_3$) δ 166.1, 148.4, 142.9, 142.7, 142.5, 141.5, 140.3, 136.0, 133.7, 132.0, 131.6, 131.4, 131.24, 131.18, 130.1, 129.7, 129.2, 127.8, 127.71, 127.68, 127.63, 127.58, 127.5, 127.1, 126.8, 126.6, 126.4, 125.6, 121.2, 118.6, 62.8. **HRMS:** (ESI) m/z: $[M+H]^+$ Calcd for $C_{38}H_{29}O_3^+$ 533.2111; Found 533.2106. **Optical:** $[\alpha]_{25}^{D}$ = -1.4 (c = 0.20, CH_2Cl_2, 95% ee). **HPLC** (IA, ^{i}PrOH/n-hexane = 20/80, flow rate = 0.8 mL/min, 254 nm) t_R = 22.0 min (major), 18.0 min (minor).

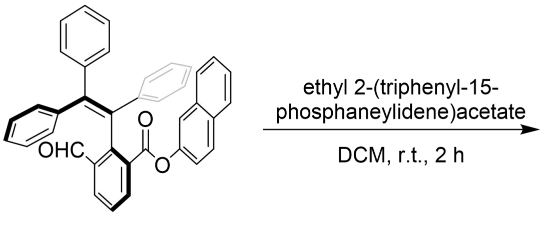

3a **10**

To a dried reaction vial with a stir bar was added the **3a** (53.0 mg, 0.1 mmol, 1.0 equiv.) and DCM (1.5 mL) Subsequently, ethyl 2-(triphenyl-15-phosphaneylidene) acetate (52.3 mg, 0.15 mmol, 1.5 equiv.) was added After stirring at room temperature for 2 h until the starting material consumed, the reaction mixture was concentrated under reduced pressure and the residue was subjected to the preparative thin layer chromatography (PE/DCM = 2:1) to afford the desired product **10** (44.4 mg, 74%, 96% ee). **^{1}H NMR** (600 MHz, $CDCl_3$) δ 7.99 (dd, *J* = 7.7, 1.3 Hz, 1H), 7.84 – 7.77 (m, 2H), 7.77 – 7.72 (m, 1H), 7.56 (dt, *J* = 7.7, 1.2 Hz, 1H), 7.49 – 7.43 (m, 2H), 7.35 –

7.30 (m, 2H), 7.19 (m, 1H), 7.16 – 7.14 (m, 2H), 7.12 – 7.03 (m, 11H), 6.97 – 6.93 (m, 3H), 5.88 (m, 1H), 4.03 (m, 1H), 3.93 (m, 1H), 1.13 (t, J = 7.1 Hz, 3H). **^{13}C NMR** (151 MHz, $CDCl_3$) δ 165.9, 165.1, 148.3, 143.5, 143.3, 143.2, 143.1, 142.9, 141.3, 136.50, 136.46, 134.6, 133.6, 131.40, 131.38, 131.35, 131.3, 131.1, 130.8, 130.5, 130.1, 129.2, 127.8, 127.75, 127.71, 127.69, 127.6, 127.5, 127.42, 127.38, 127.35, 127.0, 126.8, 126.5, 126.4, 126.2, 125.6, 121.3, 121.2, 118.6, 60.0, 14.0. **HRMS:** (ESI) m/z: $[M+H]^+$ Calcd for $C_{42}H_{33}O_4^+$ 601.2373; Found 601.2366. **Optical:** $[\alpha]_{25}^{D}$ = -29.8 (c = 0.32, CH_2Cl_2, 96% ee). **HPLC** (AD-H, ^{i}PrOH/n-hexane = 20/80, flow rate = 0.8 mL/min, 254 nm) t_R = 10.1 min (major), 11.3 min (minor).

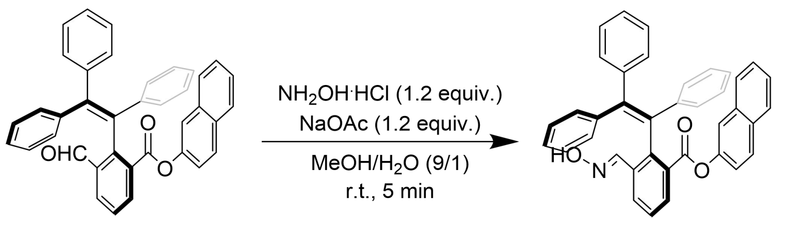

3a (53.0 mg, 0.10 mmol, 1.0 equiv.), $NH_2OH{\cdot}HCl$ (8.5 mg, 0.12 mmol, 1.2 equiv.) and NaOAc (10.0 mg, 0.12 mmol, 1.2 equiv.) were stirred at 25 °C for 5min in a 9:1 mixture of MeOH/ H_2O (1.5 mL) under air. The progress of the reaction was monitored by TLC. After completion, the reaction mixture was cooled to room temperature and treated with saturated aqueous NH_4Cl (1 mL). The organic layer was combined, dried over anhydrous Na_2SO_4 and concentrated. The crude product was purified by silica gel column chromatography (PE/EA = 1:1) to afford the desired product **11** as colorless oil (40.8 mg, 75%, 93% ee). **^{1}H NMR** (600 MHz, $CDCl_3$) δ 8.58 (s, 1H), 8.23 (s, 1H), 8.02 (dd, J = 7.7, 1.4 Hz, 1H), 7.91 (dd, J = 7.9, 1.4 Hz, 1H), 7.85 – 7.82 (m, 2H), 7.77 (dd, J = 8.0, 1.5 Hz, 1H), 7.51 – 7.46 (m, 2H), 7.38 – 7.35 (m, 2H), 7.15 (dd, J = 4.7, 1.6 Hz, 2H), 7.15 – 7.13 (m, 6H), 7.11 (m, 5H), 7.03 (t, J = 1.5 Hz, 1H), 7.02 – 6.99 (m, 2H). **^{13}C NMR** (151 MHz, $CDCl_3$) δ 165.7, 148.8, 148.2, 144.1, 143.3, 143.0, 142.4, 141.3, 135.0, 133.6, 132.2, 132.0, 131.7, 131.4, 131.3, 131.1, 130.1, 129.8, 129.3, 127.8, 127.7, 127.64, 127.60 127.58, 127.4, 127.1, 127.0, 126.7, 126.5, 125.7, 121.0, 118.6. **HRMS:** (ESI) m/z: $[M+H]^+$ Calcd for $C_{38}H_{28}NO_3^+$ 546.2064; Found 546.2057. **Optical:** $[\alpha]_{25}^{D}$ = -0.2 (c = 0.04, CH_2Cl_2, 93% ee). **HPLC** (OJ-H, ^{i}PrOH/n-hexane = 20/80, flow rate = 0.8 mL/min, 254 nm) t_R = 14.5 min (major), 20.5 min (minor).

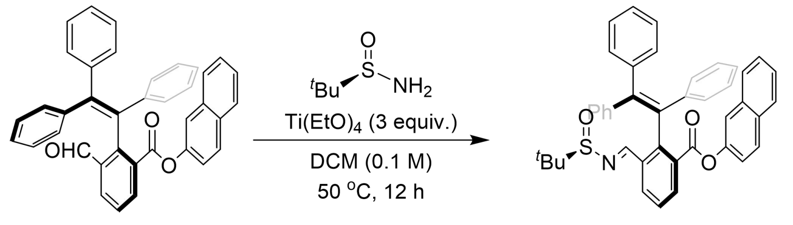

3a **12**

The **3a** (53.0 mg, 0.1 mmol, 1.0 equiv.), (R)-^{t}BuSONH$_2$ (36.5 mg, 0.3 mmol, 3.0 equiv.), and Ti(OEt)$_4$ (105 μL, 3.5 mmol, 5.0 equiv.) were mixed and stirred at 2 ml DCM at 50 °C. After reflux overnight, add 10 ml of water to the solution and filter through sand core funnel. The filtrate was extracted with DCM for three times and the organic phase was collected. The organic phase was dried with anhydrous sodium sulfate and concentrated in vacuo to give a residue, which was purified by flash chromatography to afford the products **12** as white solid (39.2 mg, 63%, 98% ee, > 20/1 dr). **^{1}H NMR** (400 MHz, CDCl$_3$) δ 9.21 (s, 1H), 8.26 (dd, J = 8.0, 1.4 Hz, 1H), 8.14 (dd, J = 7.6, 1.4 Hz, 1H), 7.83 (dd, J = 8.9, 6.1 Hz, 2H), 7.77 (dd, J = 7.2, 2.1 Hz, 1H), 7.50 – 7.43 (m, 3H), 7.34 (d, J = 2.4 Hz, 1H), 7.22 (m, 2H), 7.18 – 7.15 (m, 2H), 7.14 – 7.10 (m, 5H), 7.09 (d, J = 1.9 Hz, 1H), 7.06 – 7.03 (m, 3H), 7.01 – 6.97 (m, 3H), 1.23 (s, 9H). **^{13}C NMR** (101 MHz, CDCl$_3$) δ 165.5, 161.7, 148.2, 146.6, 143.6, 142.8, 142.3, 141.3, 133.94, 133.87, 133.6, 133.0, 132.3, 131.5, 131.4, 131.3, 131.0, 130.3, 129.3, 127.9, 127.73, 127.65, 127.58, 127.53, 127.4, 126.9, 126.8, 126.7, 126.5, 125.7, 121.0, 118.6, 58.1, 22.7. **HRMS:** (ESI) m/z: [M+H]$^+$ Calcd for $C_{42}H_{36}NO_3S^+$ 634.2410; Found 634.2404. **Optical:** $[\alpha]_{25}^{D}$ = -25.9 (c = 0.26, CH$_2$Cl$_2$, 98% ee). **HPLC** (IA, ^{i}PrOH/n-hexane = 20/80, flow rate = 0.8 mL/min, 254 nm) t$_R$ = 6.8 min (major), 6.5 min (minor).

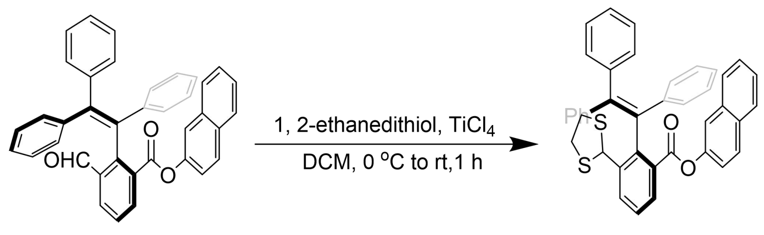

3a **13**

To a dried reaction vial with a stir bar was added the **3a** (53.0 mg, 0.1 mmol，1.0 equiv.), TiCl$_4$ (19.0 mg，0.1 mmol，1.0 equiv.) and 1, 2-ethanedithiol (28.3 mg，0.3 mmol，3.0 equiv.) in 1.0 mL DCM at 0 °C. The mixture was stirred at room temperature for 1 h, then poured into water. The organic layer was washed with water and brine, dried, and concentrated in vacuo. The residue was purified by column

chromatography (PE/DCM = 1:2) to afford the desired product **13** (53.3 mg, 88%, 93% ee). **^{1}H NMR** (400 MHz, $(CD_3)_2SO$) δ 7.99 (d, *J* = 1.5 Hz, 1H), 7.96 (d, *J* = 1.5 Hz, 1H), 7.93 (dd, *J* = 8.2, 2.0 Hz, 1H), 7.86 (dd, *J* = 7.67, 1.34 Hz, 1H), 7.56 – 7.51 (m, 3H), 7.46 (t, *J* = 7.83 Hz, 1H), 7.18 (d, *J* = 2.2 Hz, 2H), 7.16 – 7.13 (m, 5H), 7.12 – 7.07 (m, 4H), 7.05 – 7.01 (m, 4H), 6.98 – 6.95 (m, 2H), 6.05 (s, 1H), 3.60 – 3.42 (m, 2H), 3.32 – 3.18 (m, 2H). **^{13}C NMR** (101 MHz, $(CD_3)_2SO$) δ 165.4, 148.1, 142.6, 142.3, 142.0, 141.8, 141.3, 141.0, 135.6, 133.9, 133.2, 131.0, 130.9, 130.6, 129.9, 129.7, 129.4, 128.1, 128.0, 127.8, 127.7, 127.6, 127.5, 127.2, 127.0, 126.8, 125.9, 121.3, 118.4, 59.8, 51.3, 20.8, 14.1. **HRMS:** (ESI) m/z: $[M+H]^+$ Calcd for $C_{40}H_{31}O_2S_2^+$ 607.1760; Found 607.1751. **Optical:** $[\alpha]_{25}^{D}$ = -5.8 (c = 0.23, CH_2Cl_2, 93% ee). **HPLC** (IA, ^{i}PrOH/n-hexane = 30/70, flow rate = 0.8 mL/min, 254 nm) t_R = 7.9 min (major), 6.6 min (minor).

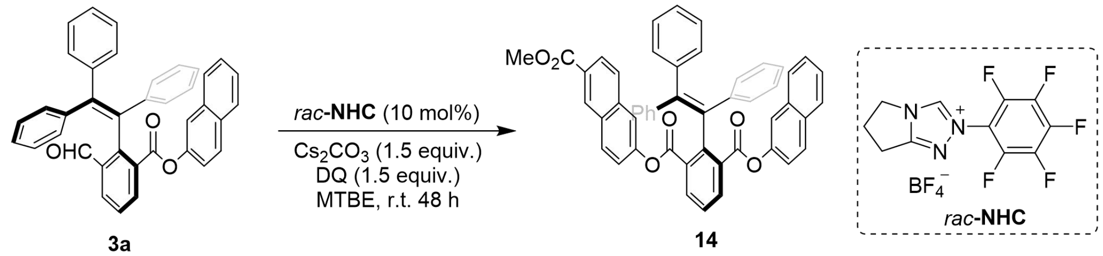

TAE dialdehyde **3a** (53.0 mg, 0.1 mmol, 1.0 equiv.), methyl 6-hydroxy-2-naphthoate (30.4 mg，0.15 mmol, 1.5 equiv.), *rac*-**NHC** (3.5 mg, 0.01 mmol, 10 mol%), Cs_2CO_3 (48.8 mg, 0.15 mmol, 1.5 equiv.), and DQ (57.2 mg, 0.15 mmol, 1.5 equivalent) were placed in the reaction tube under the N_2 atmosphere, and anhydrous MTBE (1.5 mL) was added to the reaction mixture via a syringe, and stir at room temperature for 48 h. After the reaction is complete, the crude mixture is purified by column chromatography on silica gel to obtain the corresponding product **14** (59.8 mg, 82%, 96% ee). **^{1}H NMR** (600 MHz, $CDCl_3$) δ 8.62 (d, *J* = 1.7 Hz, 1H), 8.13 – 8.08 (m, 3H), 7.95 (d, *J* = 8.9 Hz, 1H), 7.86 – 7.81 (m, 3H), 7.78 (dd, *J* = 8.0, 1.5 Hz, 1H), 7.49 (m, 4H), 7.43 (d, *J* = 2.2 Hz, 1H), 7.26 – 7.24 (m, 2H), 7.18 – 7.15 (m, 5H), 7.12 (m, 6H), 7.10 – 7.04 (m, 4H), 3.99 (d, *J* = 1.0 Hz, 3H). **^{13}C NMR** (151 MHz, $CDCl_3$) δ 167.0, 165.5, 165.3, 150.1, 148.3, 146.0, 143.1, 142.9, 141.3, 141.2, 135.9, 135.8, 133.6, 133.5, 133.5, 133.4, 133.1, 132.2, 131.4, 131.2, 130.8, 130.48, 130.46, 129.3, 127.9, 127.7, 127.6, 127.4, 127.3, 127.0, 126.7, 126.53, 126.50, 126.0, 125.7, 121.9, 121.0, 118.6, 118.5, 52.3. **HRMS:** (ESI) m/z: $[M+H]^+$ Calcd for

$C_{50}H_{35}O_6^+$ 731.2428; Found 731.2413. **Optical:** $[\alpha]_{25}^{D}$ = -0.2 (c = 0.18, CH_2Cl_2, 96% ee). **HPLC** (OD-H, ^{i}PrOH/n-hexane = 10/90, flow rate = 0.8 mL/min, 254 nm) t_R = 18.2 min (major), 15.7 min (minor).

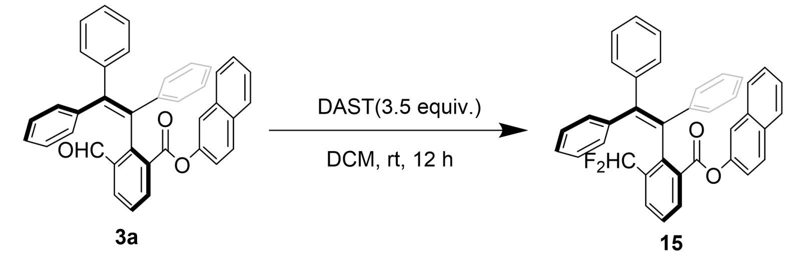

3a 15

To a dry Schlenk tube containing compound **3a** (53.0 mg, 0.10 mmol, 1.0 equiv.) in dry CH_2Cl_2 (0.1mL), and (*N*,*N*-diethylamino) sulfur trifluoride (DAST) (11.3 mg, 0.35 mmol, 3.5 equiv.). The mixture was stirred overnight at rt. The resulting mixture was CHF_2 treated with 2 drops of water and loaded onto celite. After evaporation, the reaction mixture was purified using silica gel flash column chromatography (PE/EA=5:1) as eluent to afford **15** as a pale-yellow liquid (21.6 mg, 39%, 95% ee). **^{1}H NMR** (600 MHz, $CDCl_3$) δ 8.11 – 8.06 (m, 1H), 7.85 – 7.79 (m, 3H), 7.77 (dd, *J* = 8.0, 1.6 Hz, 1H), 7.48 (m, 3H), 7.36 (d, *J* = 2.3 Hz, 1H), 7.16 – 7.08 (m, 13H), 7.04 – 7.00 (m, 3H), 6.89 (m, 1H). **^{13}C NMR** (151 MHz, $CDCl_3$) δ 165.5, 148.2, 143.5(t, *J* = 6.0 Hz), 142.52, 142.49, 141.1, 134.3, 134.1, 134.0(t, *J* = 22.0 Hz), 133.8, 133.6, 132.6, 132.2, 131.5, 131.4, 131.2, 130.3, 129.52, 129.48, 129.3, 127.9, 127.82, 127.81, 127.7, 127.63, 127.60, 127.3, 127.0, 126.8, 126.5, 125.7, 121.0, 118.6, 114.3, 112.7(t, *J* = 237.9 Hz), 111.2. **^{19}F NMR** (565 MHz, $CDCl_3$) δ -107.27 (dd, *J* = 303.7, 55.7 Hz), -115.16 (dd, *J* = 303.7, 55.1 Hz). **HPLC** (IA, ^{i}PrOH/n-hexane = 10/90, flow rate = 0.8 mL/min, 254 nm) t_R = 9.7 min (major), 14.3 min (minor).

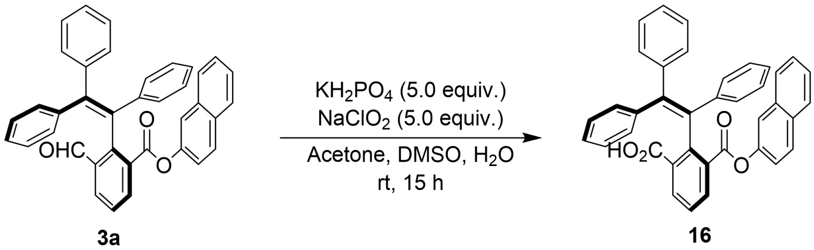

3a 16

A round bottom flask (25 ml) was charged with **3a** (106.0 mg, 0.2 mmol, 1.0 equiv.), followed by acetone (4.0 ml), DMSO (3.2 ml), and water (6 ml) addition. Then, KH_2PO_4 (136.1 mg, 1.0 mmol, 5.0 equiv.) and $NaClO_2$ (90.5 mg, 1.0 mmol, 5.0 equiv.) were added in one portion at room temperature. The mixture was stirred at room temperature overnight (15 hours). Once the starting material was no longer

detected by (TLC), the volatile solvents were concentrated under reduced pressure. The resulting mixture was diluted with diluted hydrochloric acid (1 M, 8 ml) and extracted with EtOAc organic phases were collected, washed with brine (3 x 20 ml) and dried under anhydrous $MgSO_4$. After filtration of the solid, the filtrate was concentrated under reduced pressure. The crude product was purified by column chromatography (eluting by hexane/EtOAc=3:1) affording product **16** as a white foam (84.1 mg, 77%). **^{1}H NMR** (400 MHz, $CDCl_3$) δ 8.04 (dd, J = 7.8, 1.4 Hz, 1H), 7.89 – 7.74 (m, 4H), 7.47 (m, 2H), 7.42 – 7.29 (m, 2H), 7.16 – 7.11 (m, 4H), 7.09 – 6.99 (m, 11H), 6.96 (d, J = 9.2 Hz, 2H). **^{13}C NMR** (101 MHz, $CDCl_3$) δ 148.2, 143.1, 141.3, 133.8, 133.6, 132.1, 131.5, 131.1, 130.4, 129.2, 127.7, 127.6, 127.2, 126.9, 126.6, 126.5, 126.4, 125.7, 121.0, 118.6.

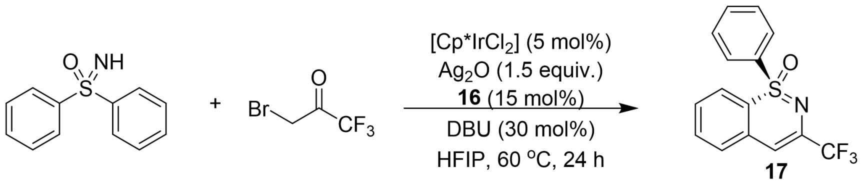

Iminodiphenyl-λ^6-sulfanone (21.7 mg, 0.1 mmol, 1 equiv.), 3-bromo-1,1,1-trifluoropropan-2-one (23.0 mg, 0.12 mmol, 1.2 equiv.), $[Cp^*IrCl_2]_2$ (4.0 mg, 0.005 mmol, 5 mol%), Ag_2O (34.8 mg, 0.15 mmol, 1.5 equiv.), DBU (4.6 mg, 0.03 mmol, 30 mol%.) and **16** (8.0 mg, 0.015 mmol, 15 mol%.) were placed in the reaction tube under the N_2 atmosphere, and anhydrous HFIP (1.0 mL) was added to the reaction mixture via a syringe, and stir at 60 °C for 24 h. After completion of the reaction, the crude mixture was purified by column chromatography on silica gel to afford the corresponding product (19.5 mg, 42% yield, 46% ee).[2] **HPLC** (IA, ^{i}PrOH/n-hexane = 10/90, flow rate = 1.0 mL/min, 254 nm) t_R = 14.1 min (major), 9.4 min (minor).

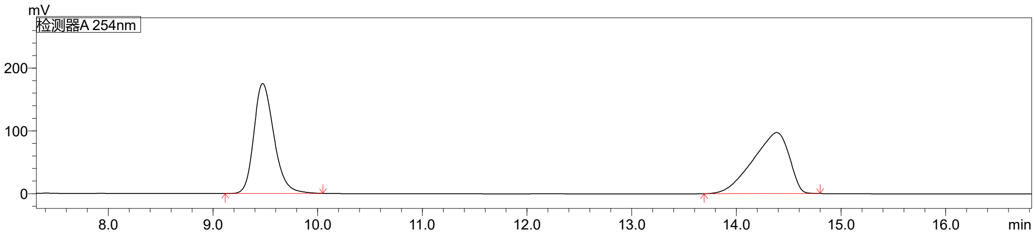

Peak#	Ret. Time	Height	Area%
1	9.475	175515	49.940
2	14.386	97340	50.060

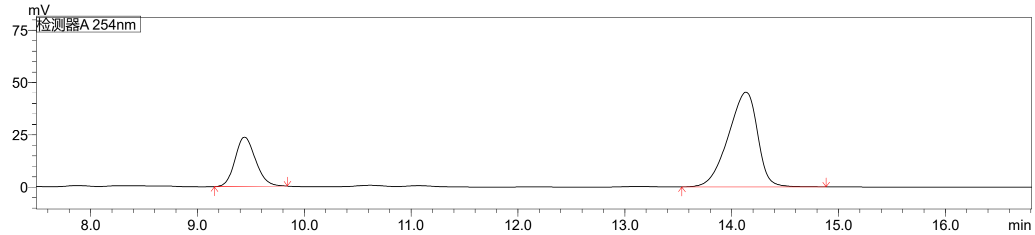

Peak#	Ret. Time	Height	Area%
1	9.441	23627	26.741
2	14.134	45362	73.259

6. Copies of Optical Spectra

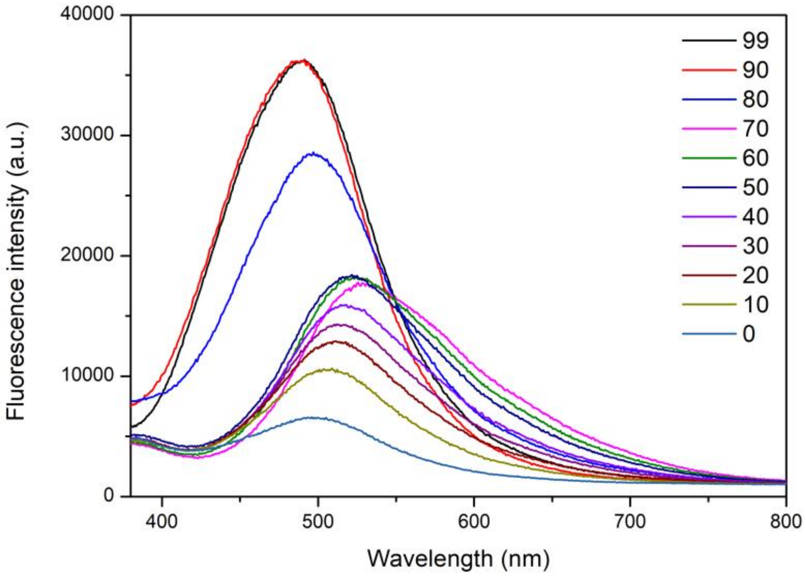

Figure S2. The PL intensity of **3a** in THF/H_2O mixed solvents increased with an increased H_2O volume fraction (f_h) to 99% (c = 10 μM, λ_{ex} = 320 nm).

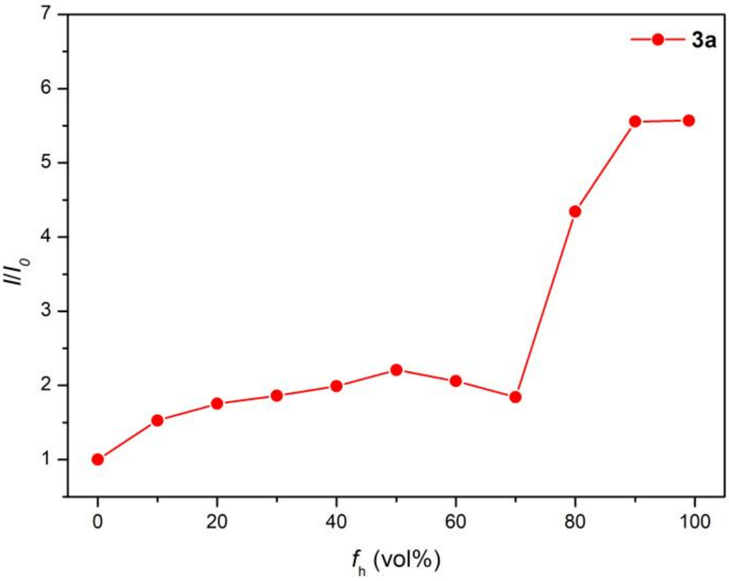

Figure S3. Relative PL intensity of **3a**.

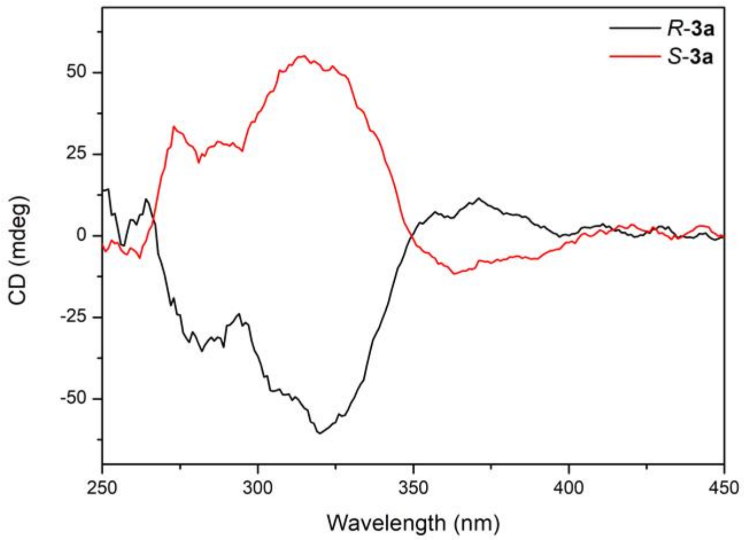

Figure S4. CD spectra of *R*-**3a** and *S*-**3a** in THF (1.0×10^{-3} M) at room temperature.

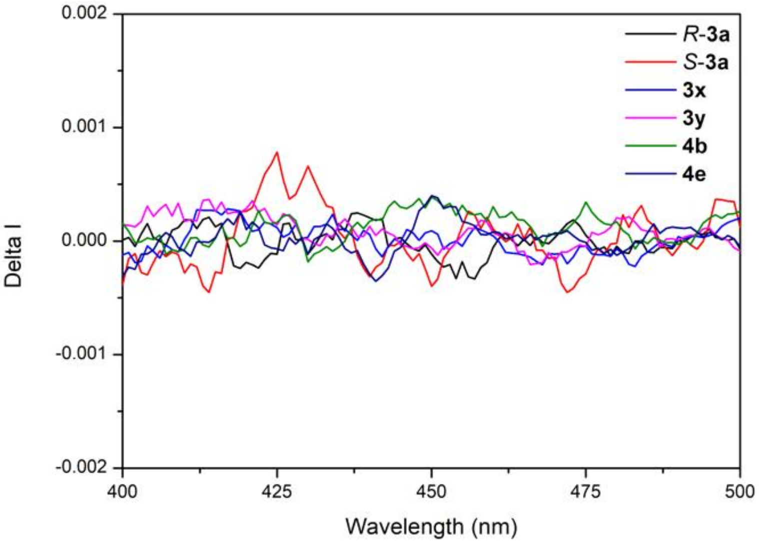

Figure S5. CPL spectra of *R*-**3a**, *S*-**3a**, **3x**, **3y**, **4b**, and **4e** in THF (1.0×10^{-3} M) at room temperature.

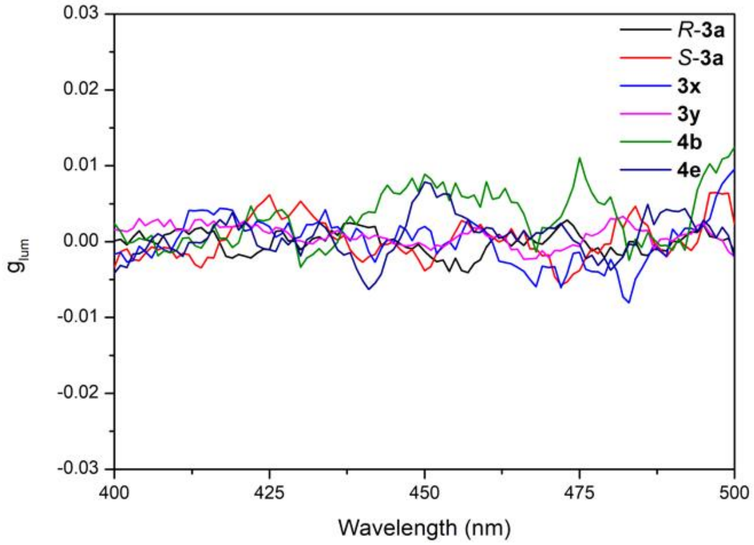

Figure S6. g_{lum} values−wavelength curve for *R*-**3a**, *S*-**3a**, **3x**, **3y**, **4b**, and **4e**.

7. Copies of ^{1}H NMR, ^{13}C NMR and ^{19}F NMR

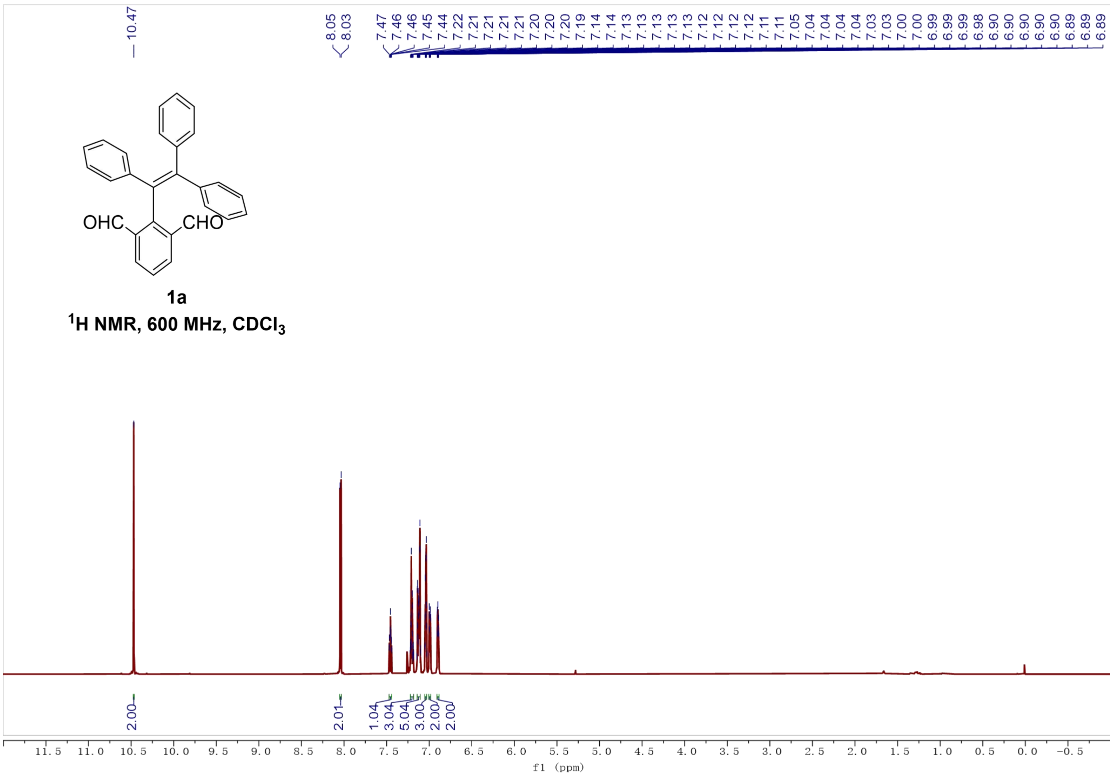

1a

^{1}H NMR, 600 MHz, $CDCl_3$

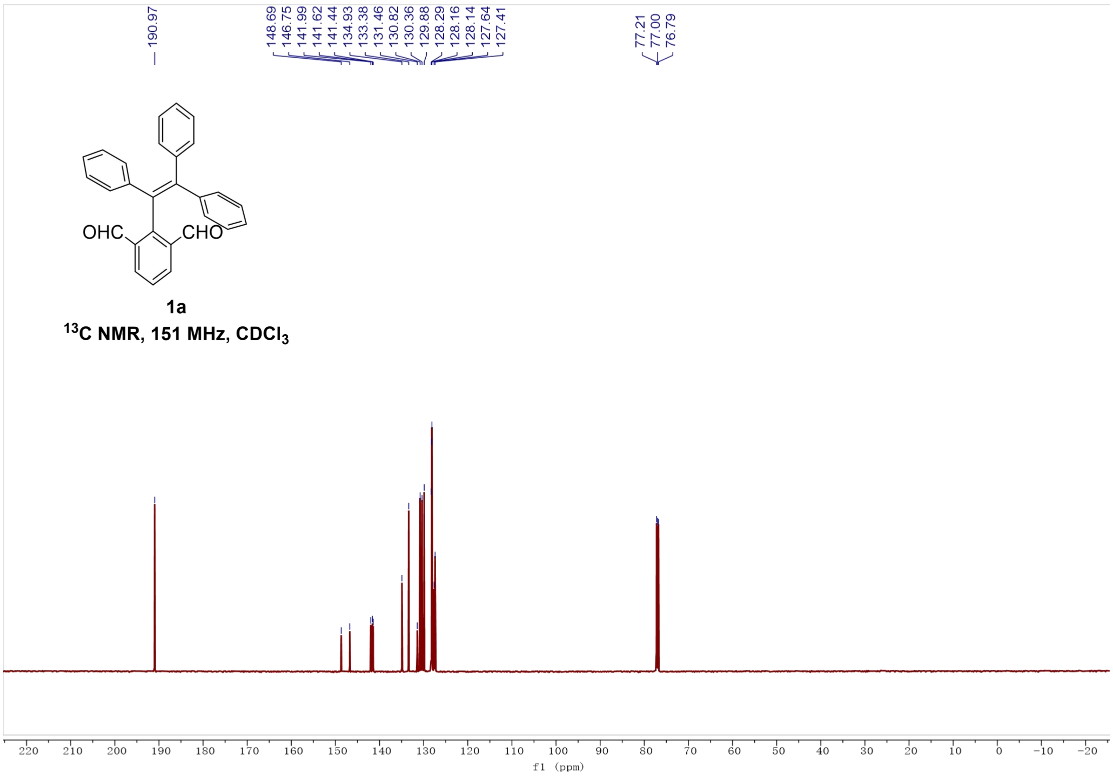

1a

^{13}C NMR, 151 MHz, $CDCl_3$

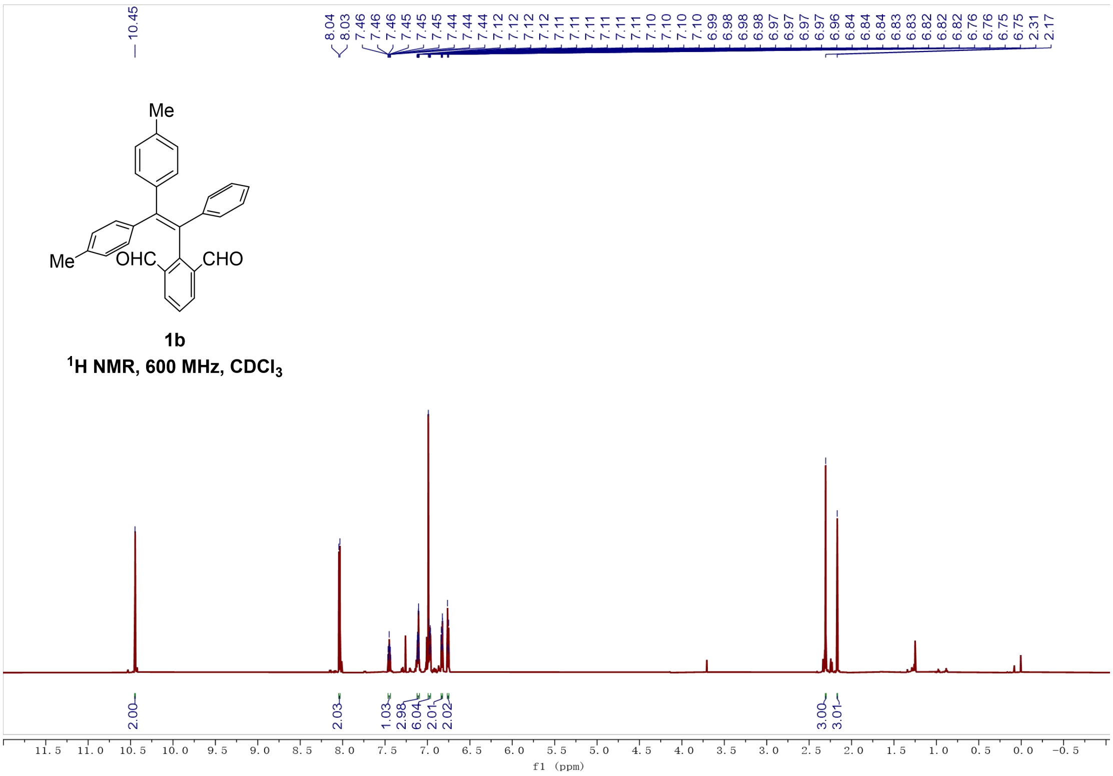
1b
^{1}H NMR, 600 MHz, CDCl$_3$
f1 (ppm)

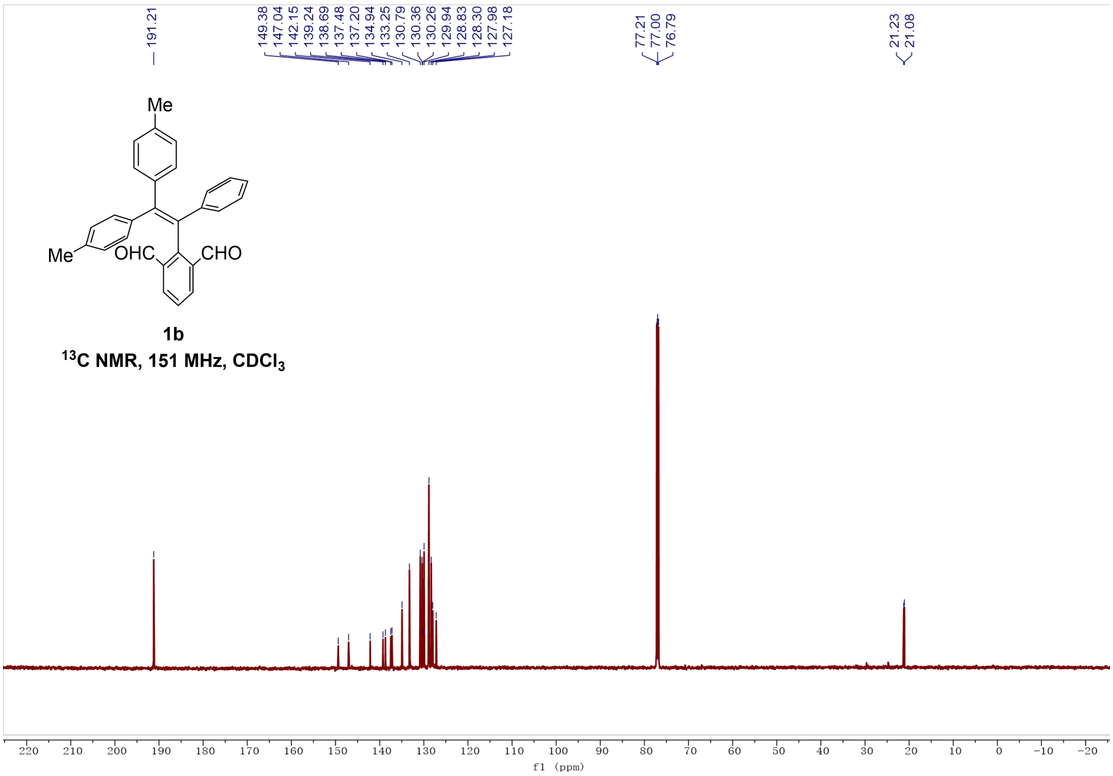
1b
^{13}C NMR, 151 MHz, CDCl$_3$
f1 (ppm)

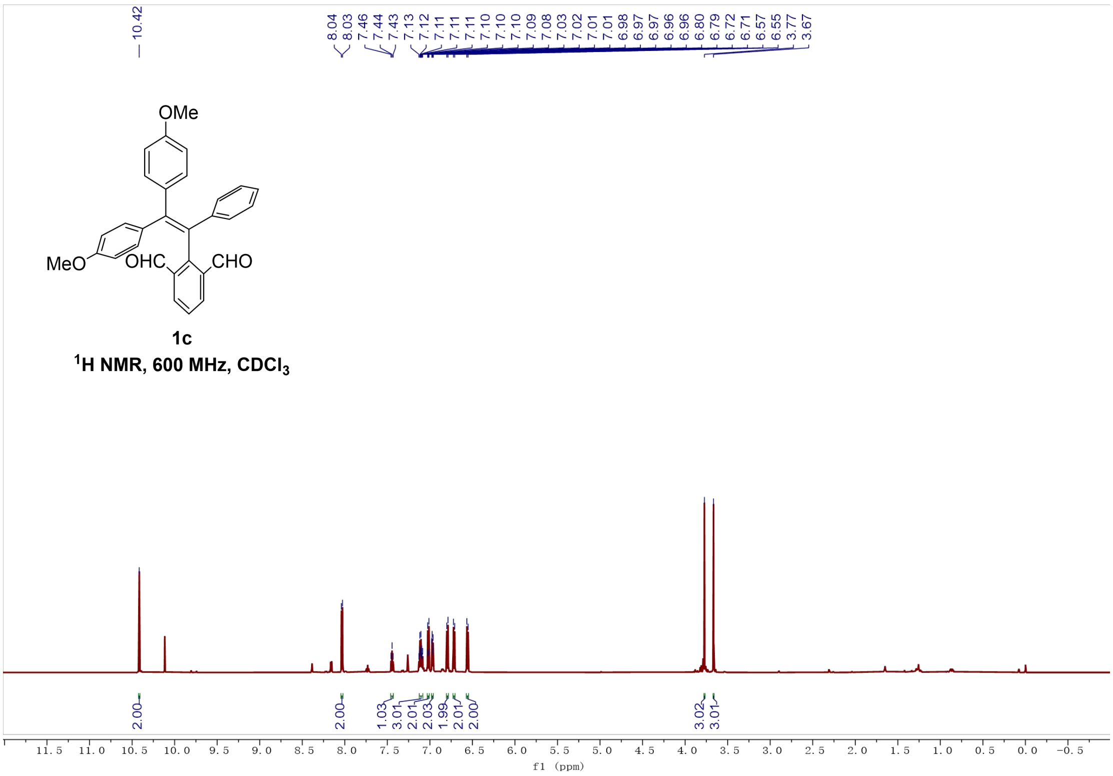

1c

^{1}H NMR, 600 MHz, $CDCl_3$

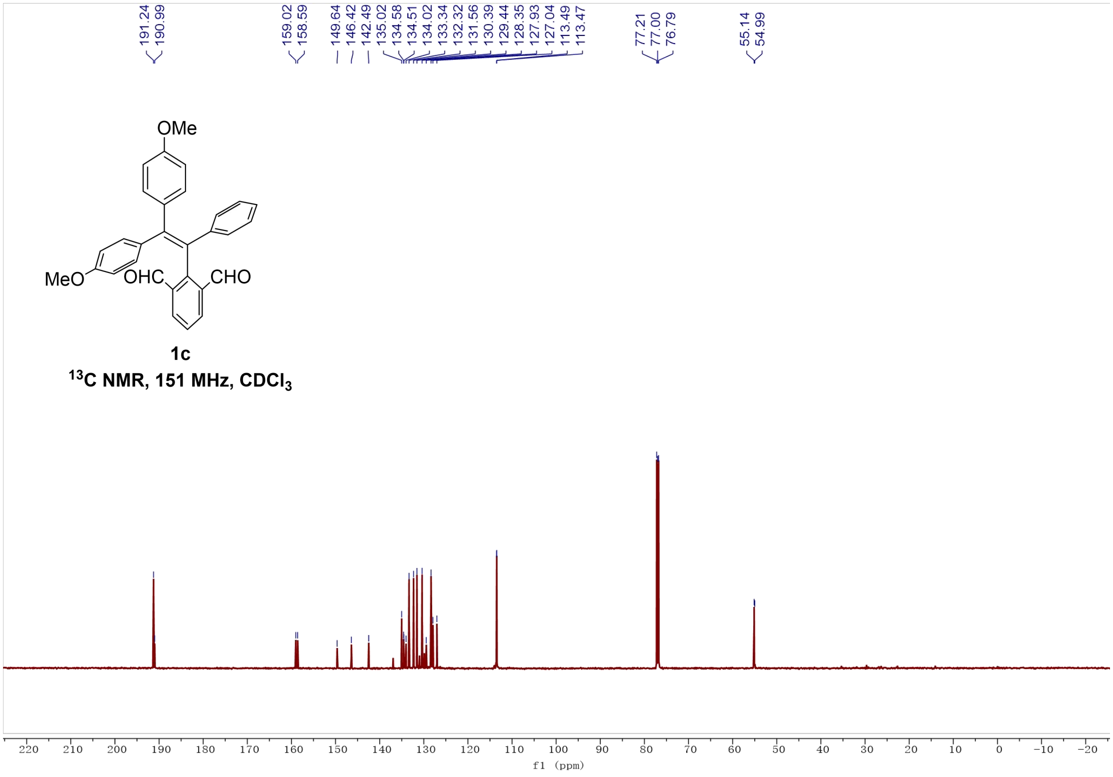

1c

^{13}C NMR, 151 MHz, $CDCl_3$

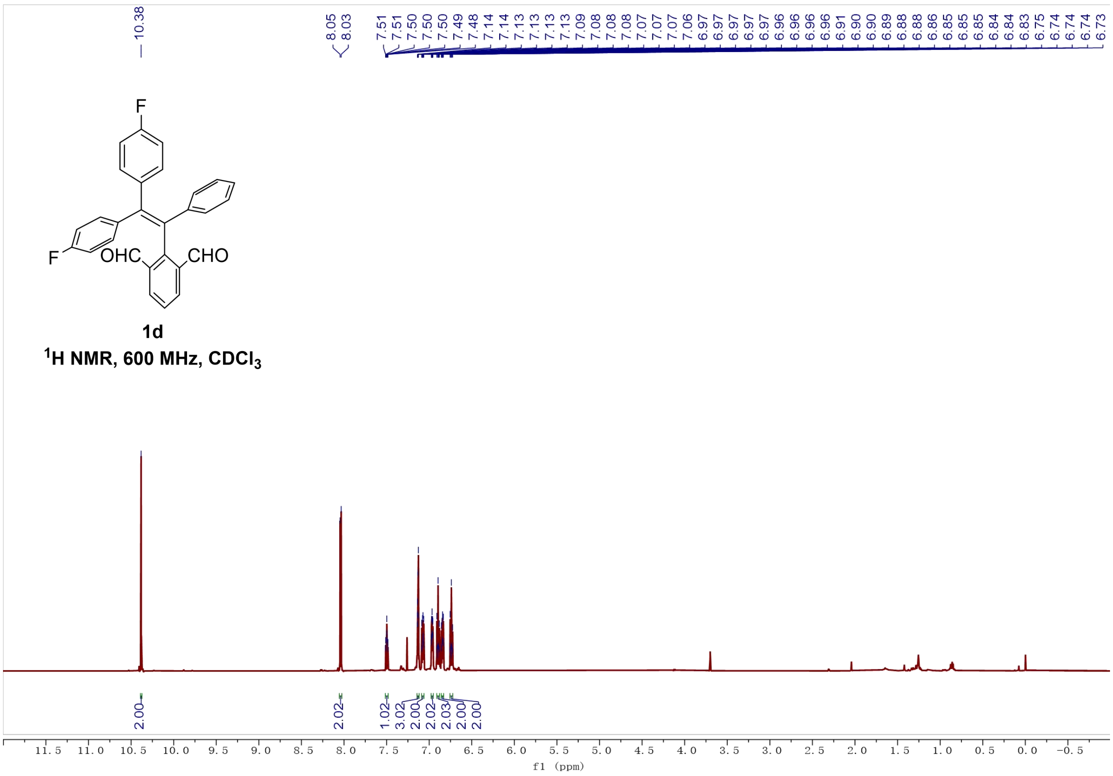
10.38
8.05
8.03
F
OHC
CHO
1d
1H NMR, 600 MHz, CDCl3
f1 (ppm)

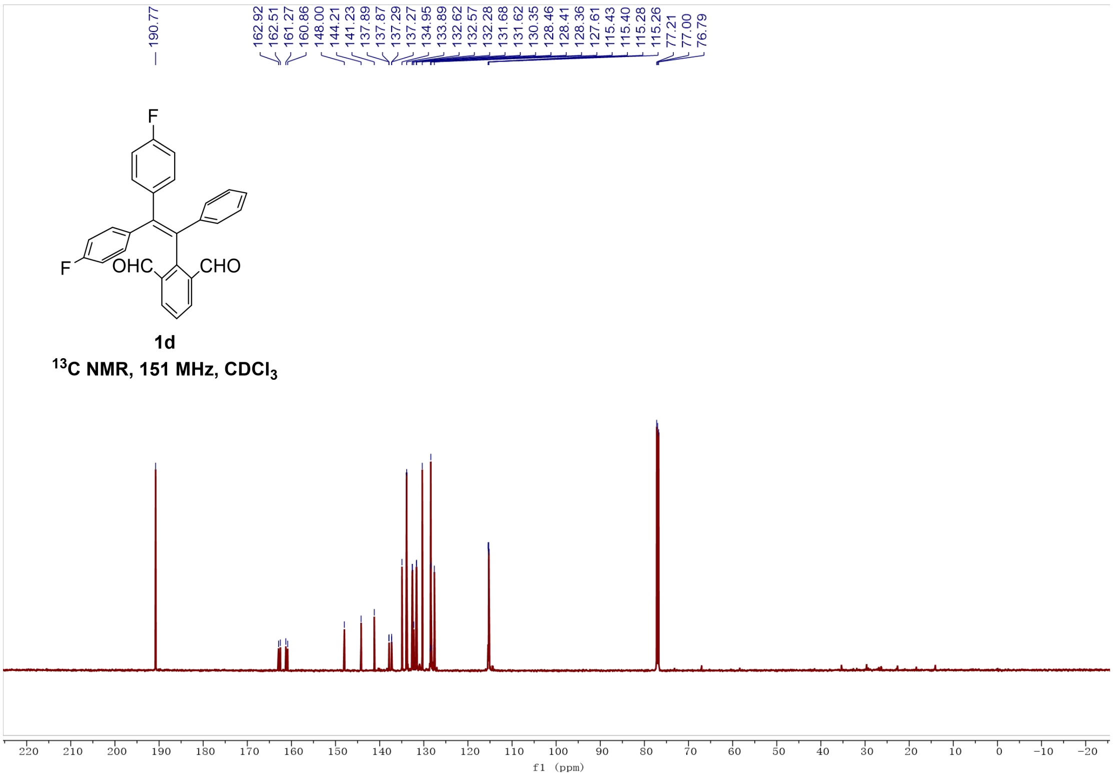
190.77
F
OHC
CHO
1d
13C NMR, 151 MHz, CDCl3
f1 (ppm)

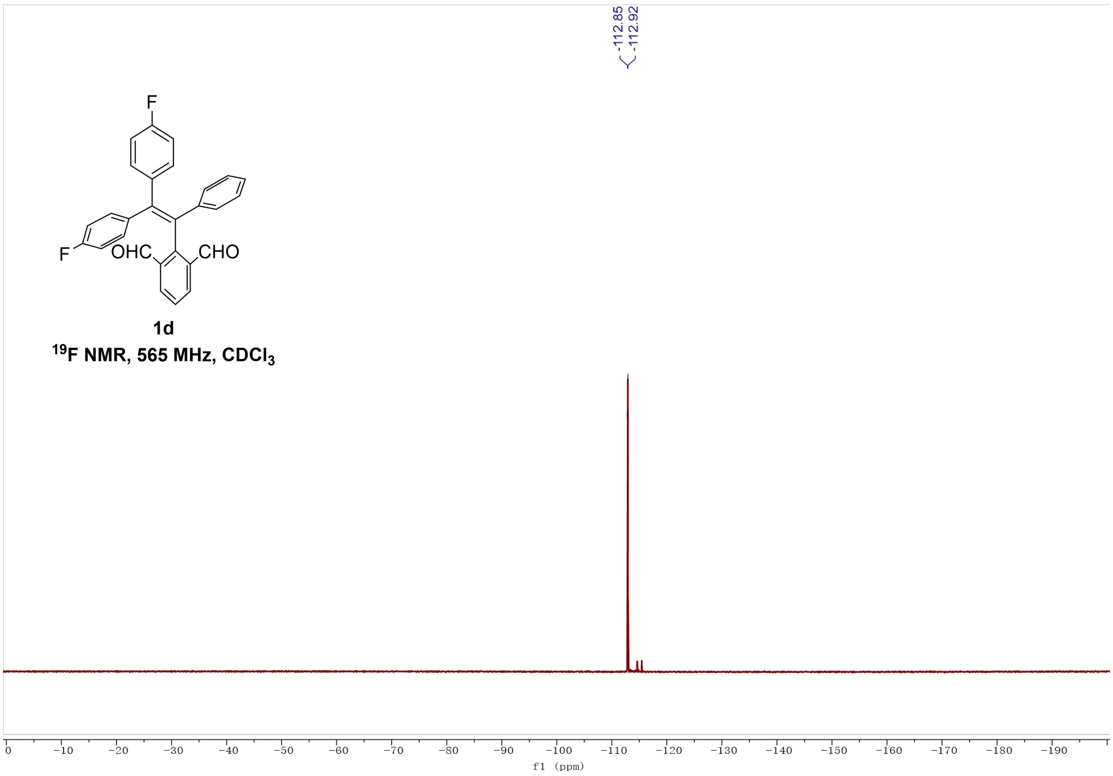

1d
^{19}F NMR, 565 MHz, $CDCl_3$

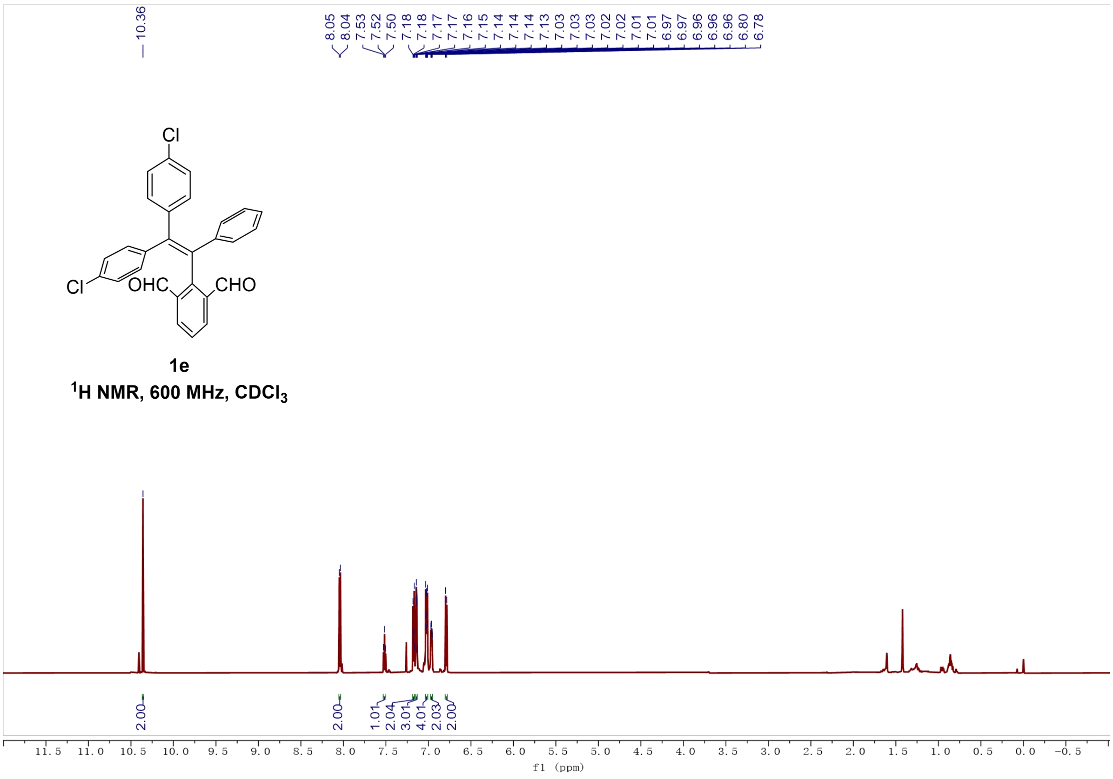

1e
^{1}H NMR, 600 MHz, $CDCl_3$

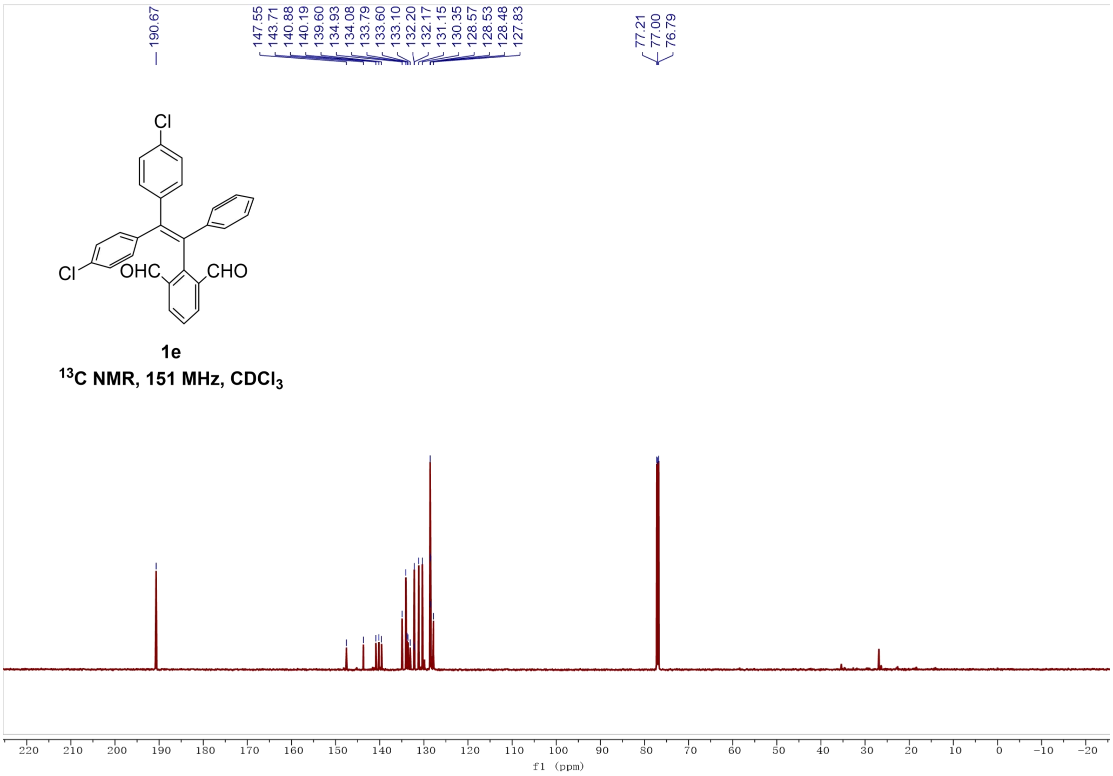

1e

^{13}C NMR, 151 MHz, $CDCl_3$

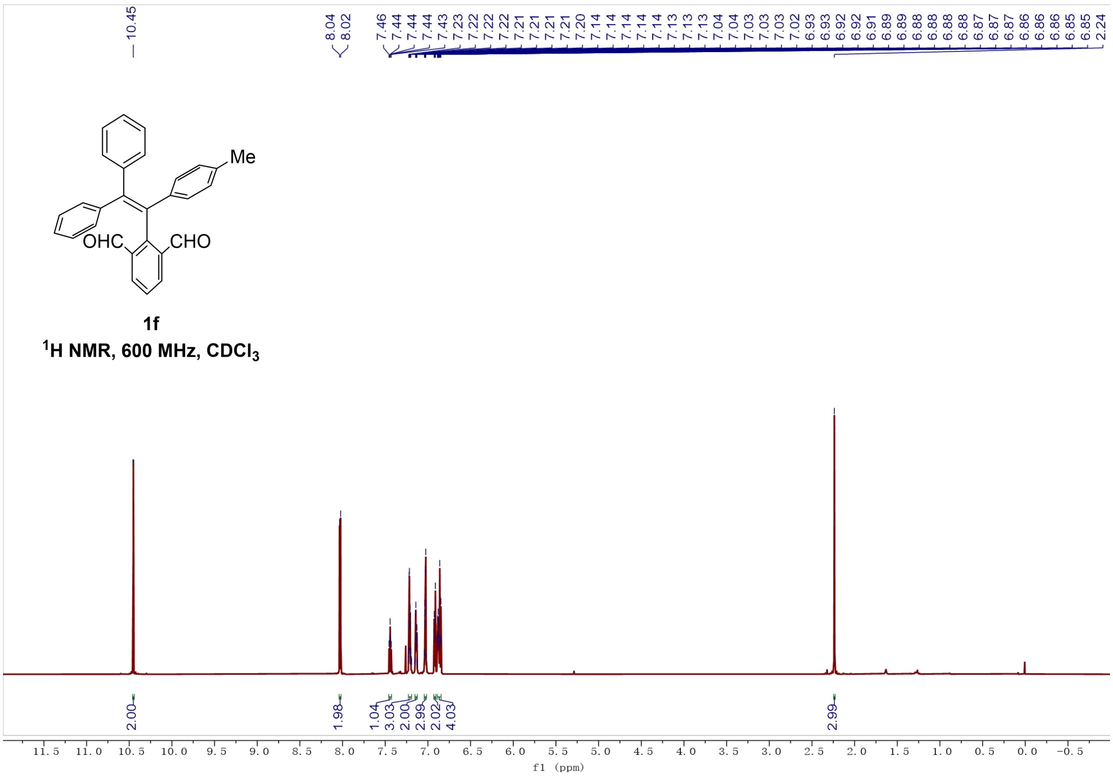

1f

^{1}H NMR, 600 MHz, $CDCl_3$

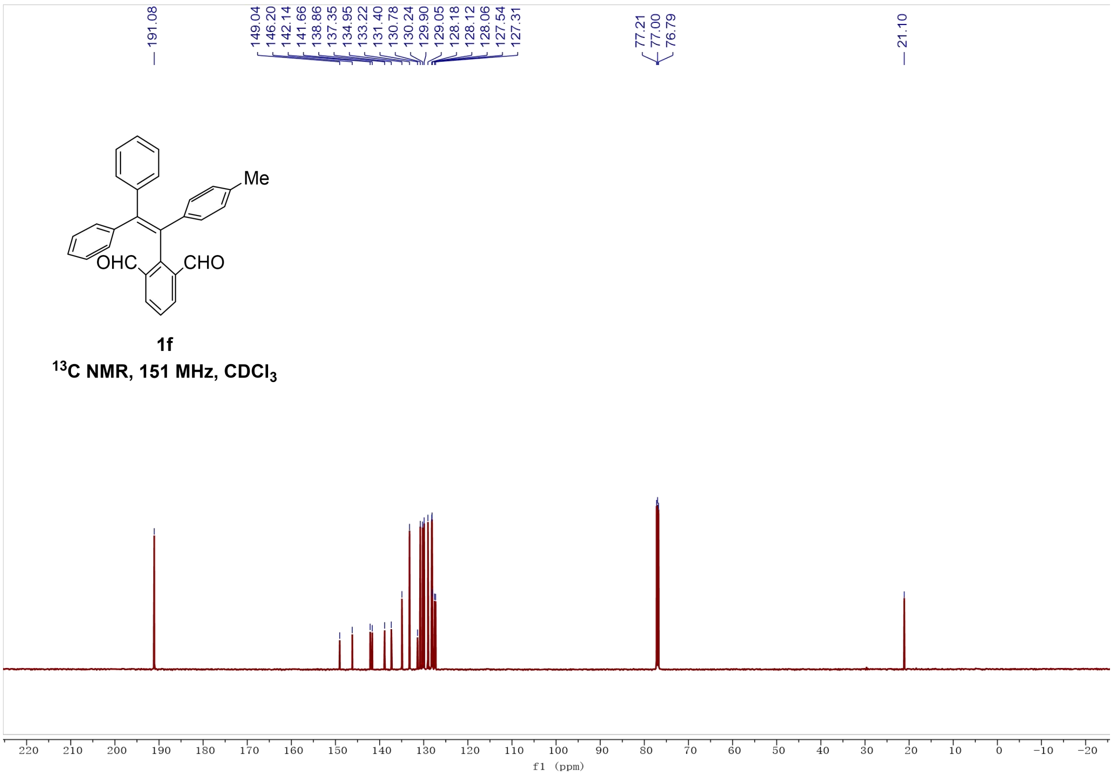

1f

^{13}C NMR, 151 MHz, $CDCl_3$

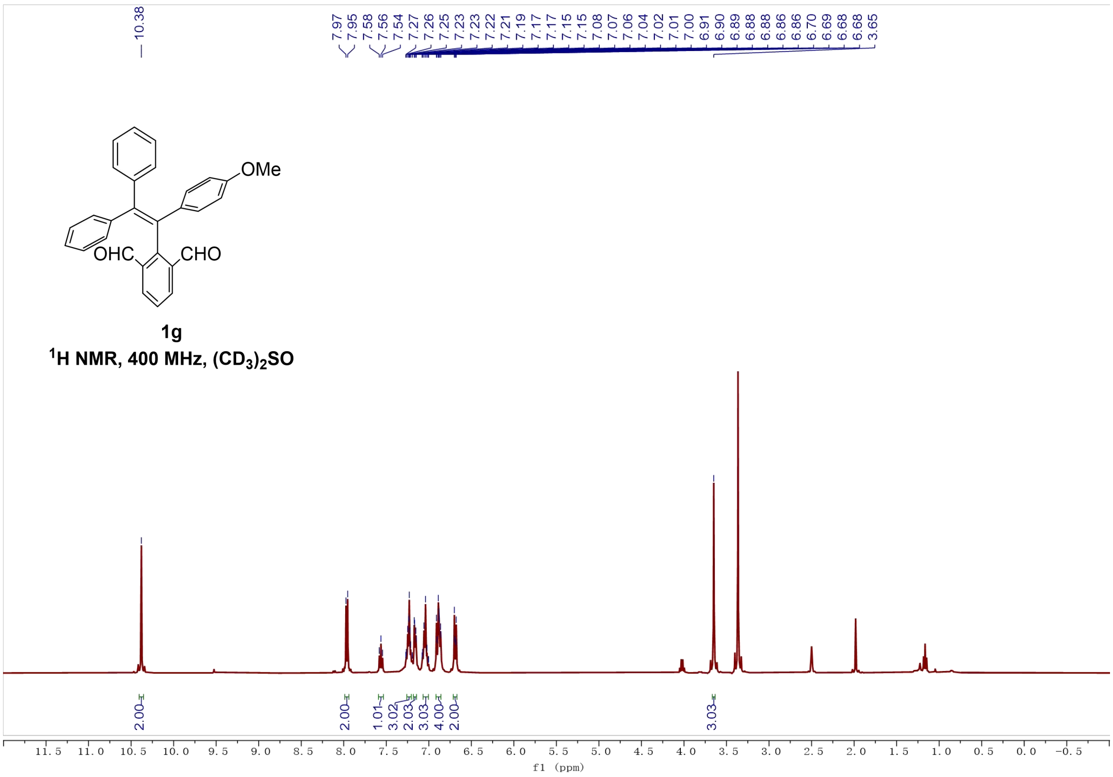

1g

^{1}H NMR, 400 MHz, $(CD_3)_2SO$

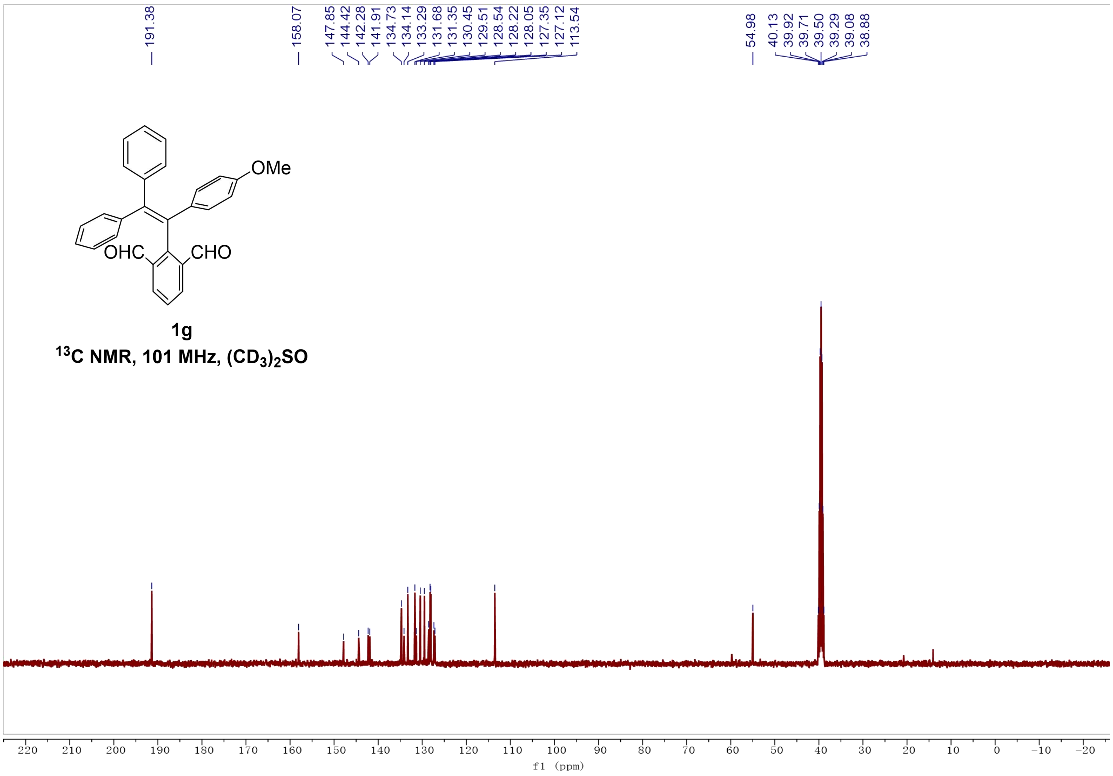
191.38
158.07
147.85
144.42
142.28
141.91
134.73
134.14
133.29
131.68
131.35
130.45
129.51
128.54
128.22
128.05
127.35
127.12
113.54
54.98
40.13
39.92
39.71
39.50
39.29
39.08
38.88
OMe
OHC
CHO
1g
13C NMR, 101 MHz, (CD3)2SO
f1 (ppm)

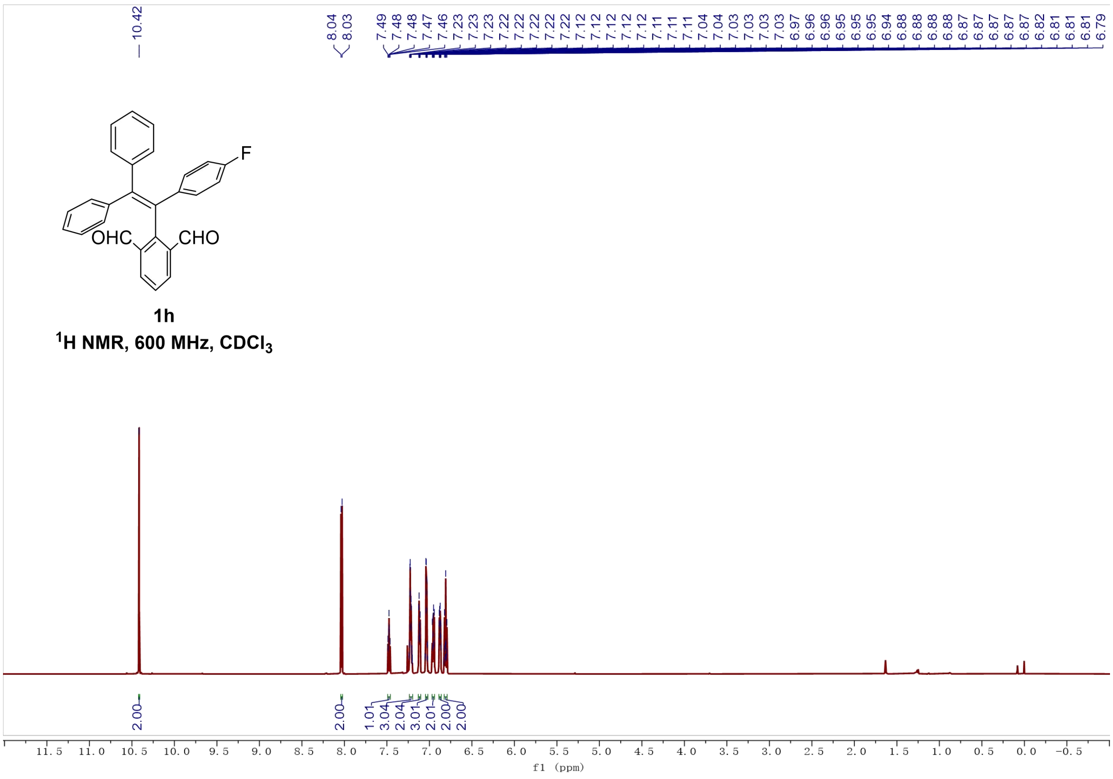
10.42
8.04
8.03
7.49
7.48
7.48
7.47
7.46
7.23
7.23
7.23
7.22
7.22
7.22
7.22
7.22
7.12
7.12
7.12
7.12
7.12
7.11
7.11
7.11
7.04
7.03
7.03
7.03
7.03
6.97
6.96
6.96
6.95
6.95
6.95
6.94
6.88
6.88
6.88
6.87
6.87
6.87
6.87
6.82
6.81
6.81
6.81
6.79
F
OHC
CHO
1h
1H NMR, 600 MHz, CDCl3
2.00
2.00
1.01
3.04
2.04
3.01
2.01
2.00
2.00
f1 (ppm)

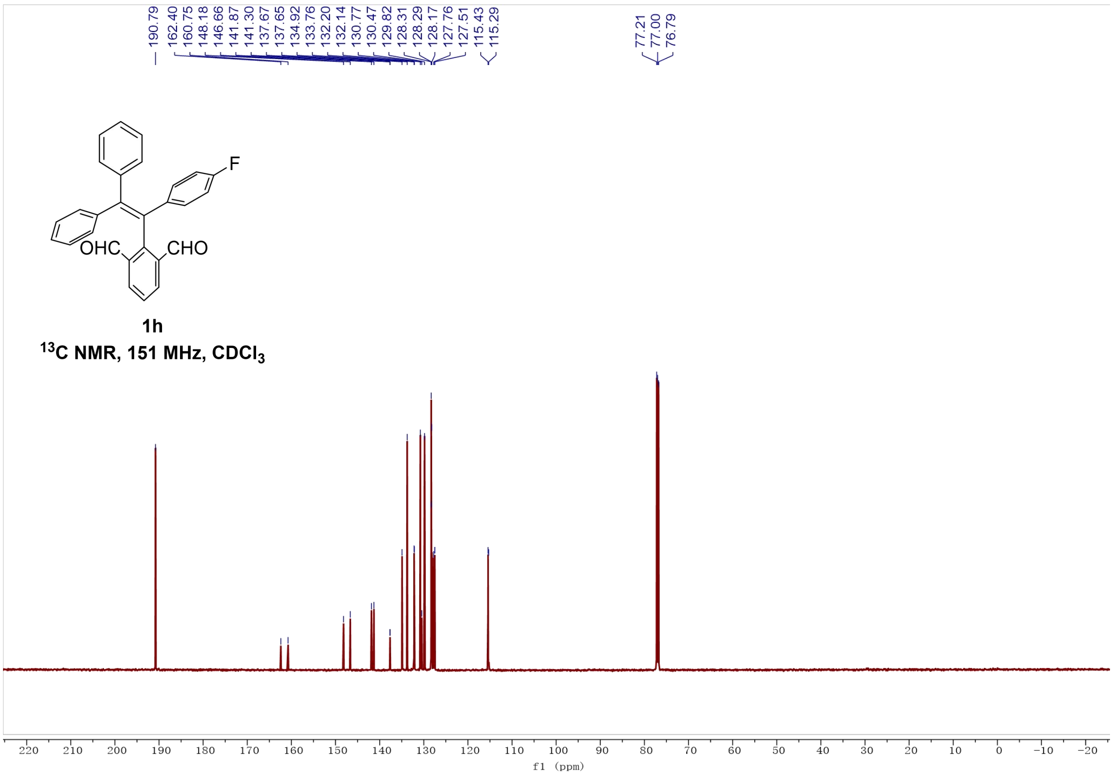

1h

^{13}C NMR, 151 MHz, $CDCl_3$

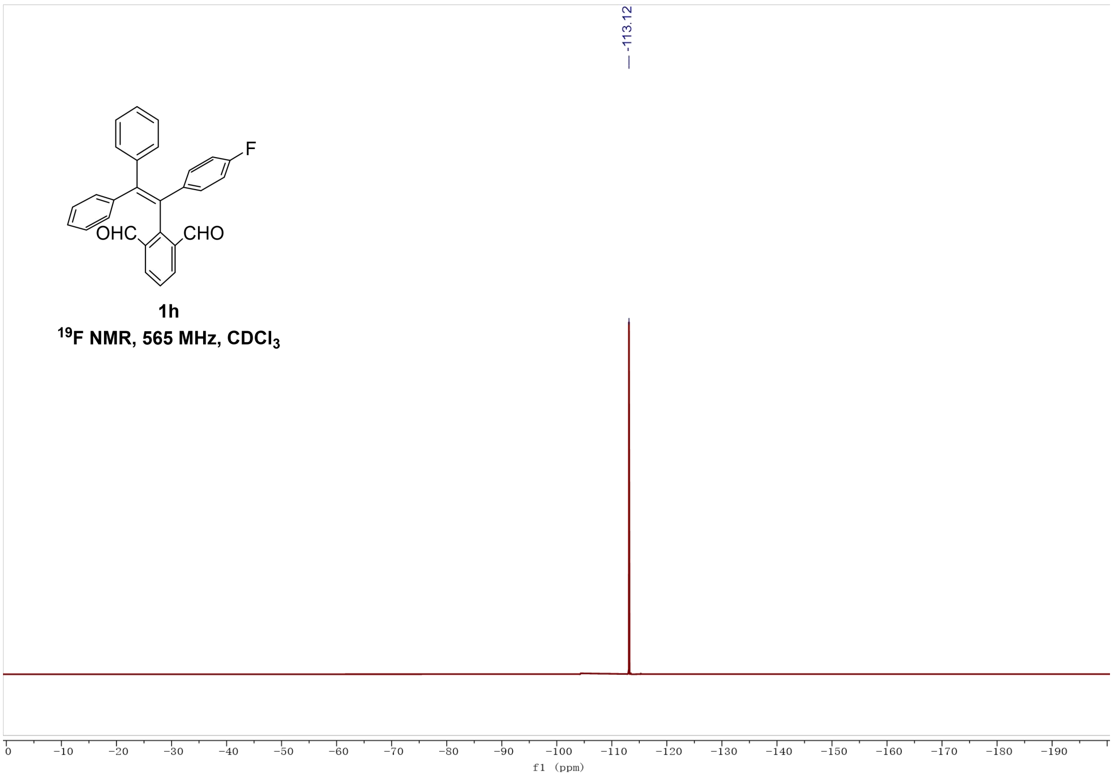

1h

^{19}F NMR, 565 MHz, $CDCl_3$

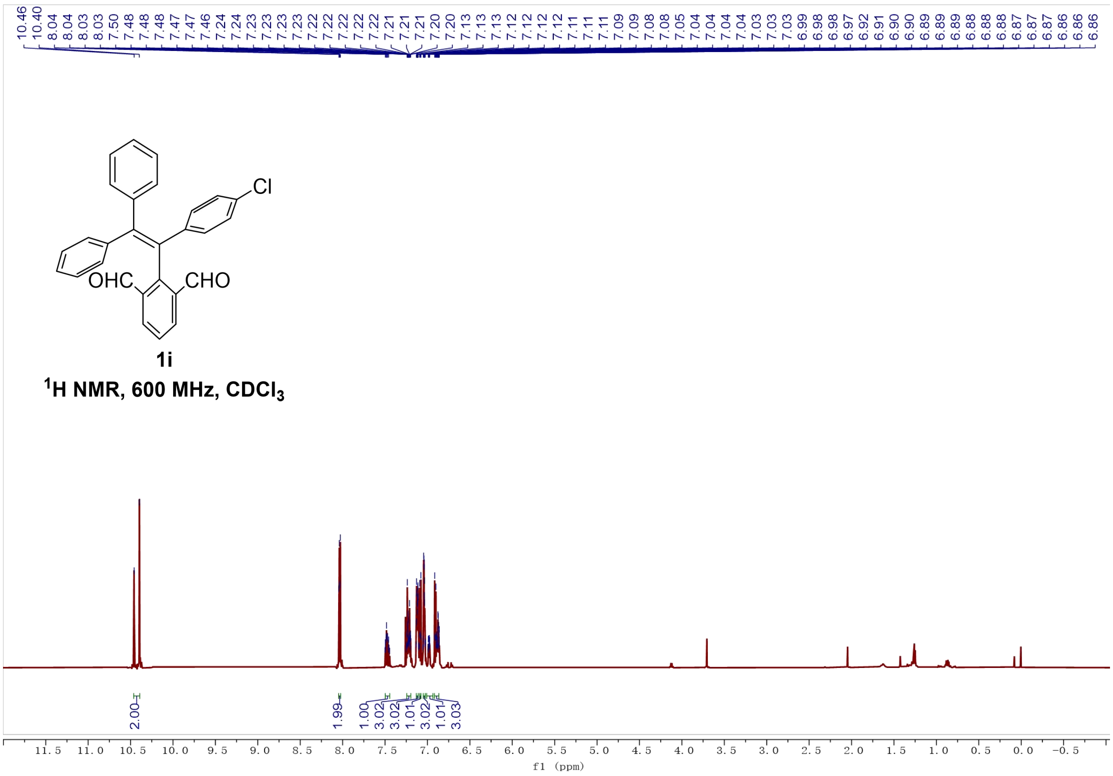
10.46 10.40 8.04 8.04 8.03 7.50 7.48 7.48 7.47 7.46 7.24 7.24 7.23 7.23 7.23 7.22 7.22 7.22 7.22 7.21 7.21 7.21 7.20 7.20 7.13 7.13 7.12 7.12 7.12 7.12 7.11 7.11 7.09 7.08 7.05 7.04 7.04 7.04 7.03 7.03 6.99 6.98 6.98 6.97 6.92 6.91 6.90 6.89 6.89 6.88 6.88 6.88 6.87 6.87 6.86 6.86
Cl
OHC
CHO
1i
^{1}H NMR, 600 MHz, $CDCl_3$
2.00
1.99
1.00
3.02
3.02
1.01
3.02
1.01
3.03
f1 (ppm)

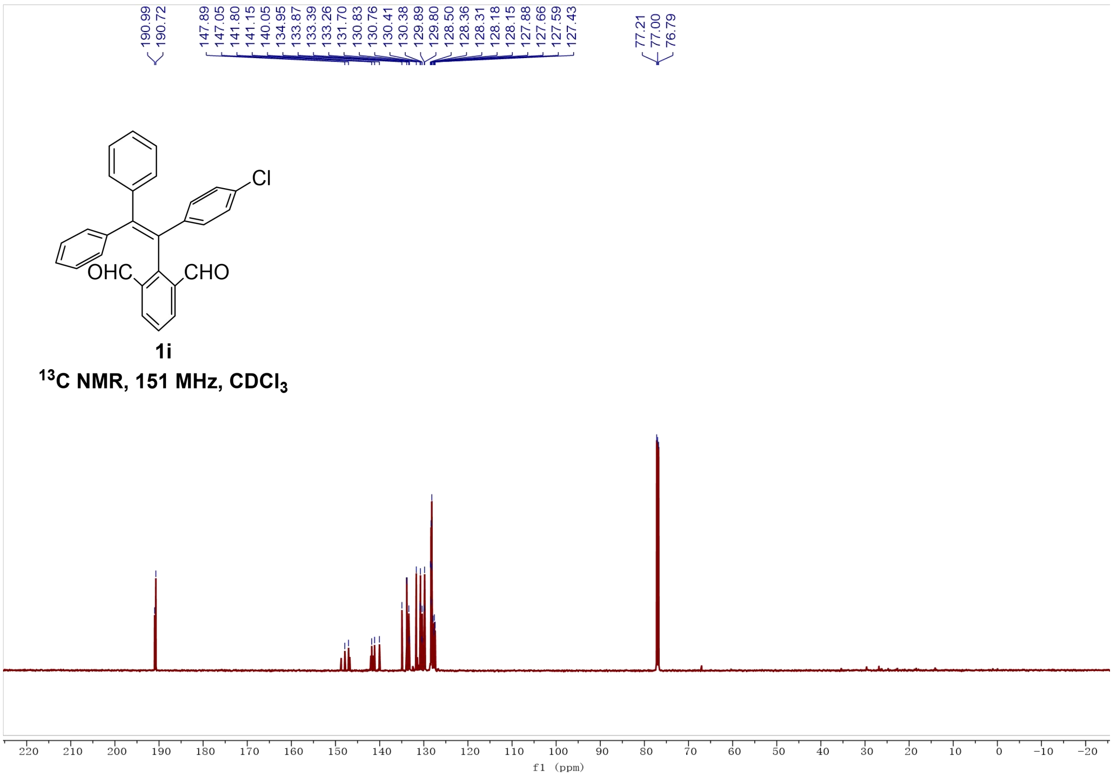
190.99 190.72
147.89 147.05 141.80 141.15 140.05 134.95 133.87 133.39 133.26 131.70 130.83 130.76 130.41 130.38 129.89 129.80 128.50 128.36 128.31 128.18 128.15 127.88 127.66 127.59 127.43
77.21 77.00 76.79
Cl
OHC
CHO
1i
^{13}C NMR, 151 MHz, $CDCl_3$
f1 (ppm)

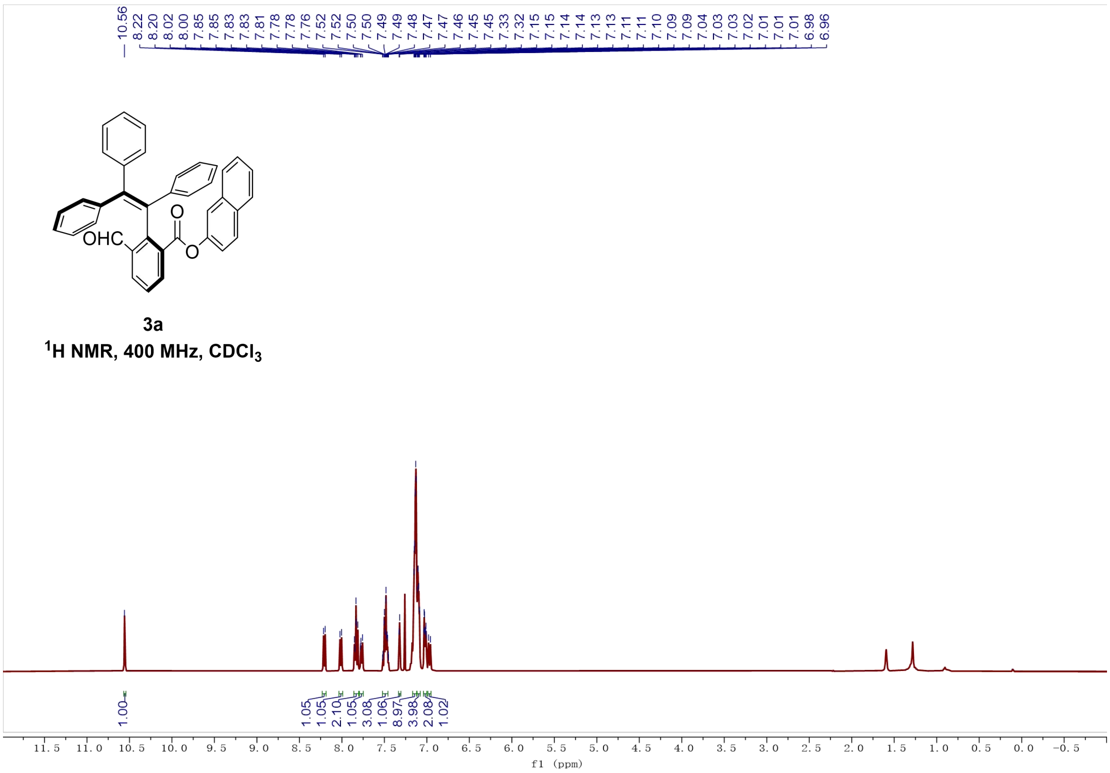
10.56 8.22 8.20 8.02 8.00 7.85 7.83 7.83 7.81 7.78 7.76 7.52 7.52 7.50 7.50 7.49 7.49 7.48 7.47 7.46 7.45 7.45 7.33 7.32 7.15 7.15 7.14 7.13 7.13 7.11 7.11 7.10 7.09 7.09 7.04 7.03 7.03 7.02 7.01 7.01 7.01 6.98 6.96
OHC
O
O
3a
1H NMR, 400 MHz, CDCl3
1.00 1.05 1.05 2.10 1.05 3.08 1.06 8.97 3.98 2.08 1.02
f1 (ppm)

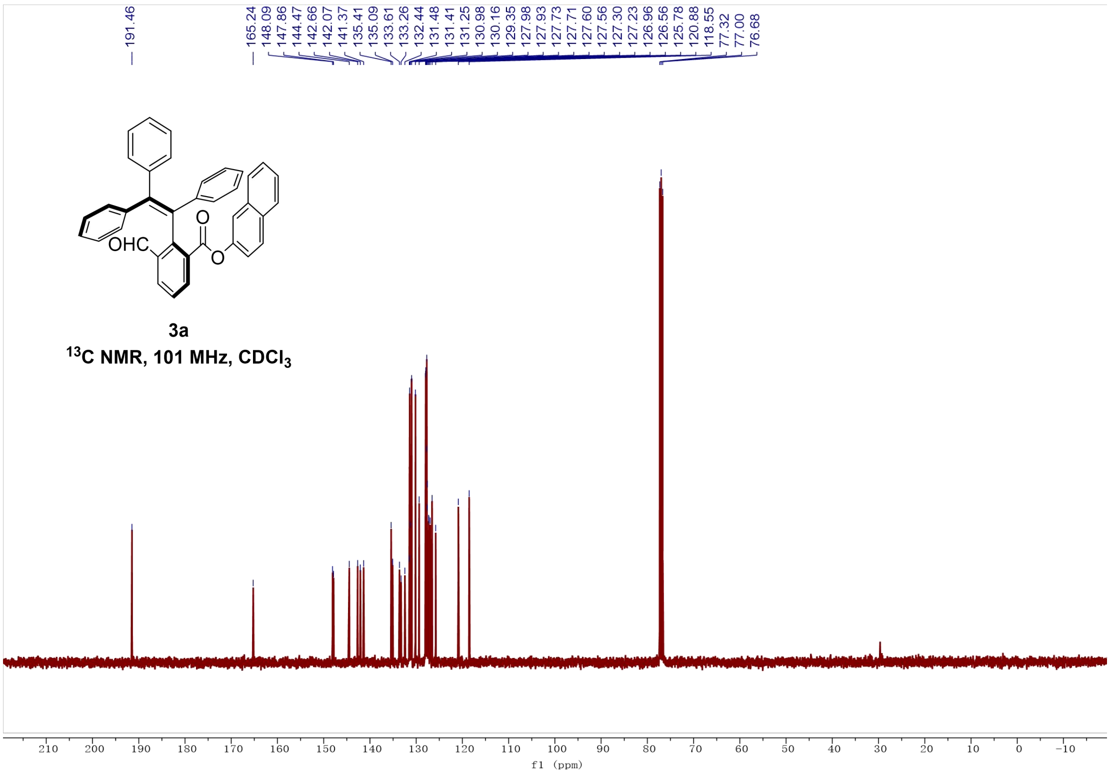
191.46 165.24 148.09 147.86 144.47 142.66 142.07 141.37 135.41 135.09 133.61 133.26 132.44 131.48 131.41 131.25 130.98 130.16 129.35 127.98 127.93 127.73 127.71 127.60 127.56 127.30 127.23 126.96 126.56 125.78 120.88 118.55 77.32 77.00 76.68
OHC
O
O
3a
13C NMR, 101 MHz, CDCl3
f1 (ppm)

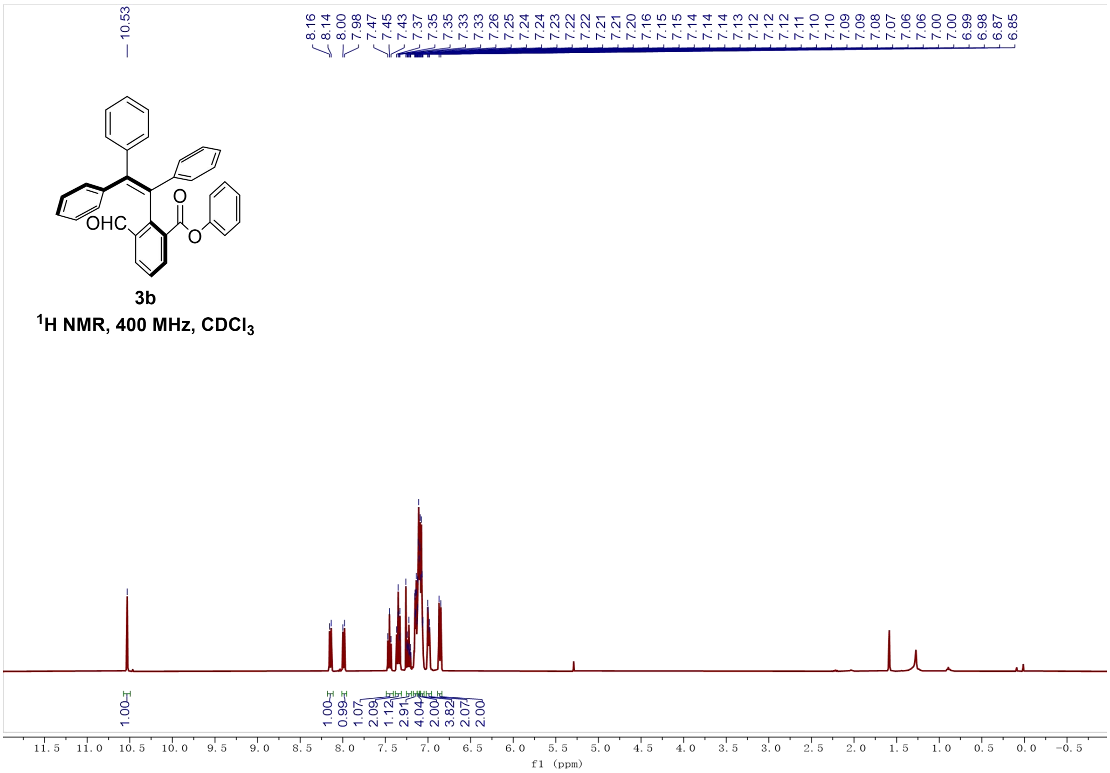
10.53
8.16
8.14
8.00
7.98
7.47
7.45
7.43
7.37
7.35
7.35
7.33
7.33
7.26
7.25
7.24
7.24
7.23
7.22
7.22
7.21
7.21
7.20
7.16
7.15
7.15
7.14
7.14
7.14
7.13
7.12
7.12
7.12
7.11
7.10
7.10
7.09
7.09
7.08
7.07
7.06
7.00
7.00
6.99
6.98
6.87
6.85
OHC
O
O
3b
1H NMR, 400 MHz, CDCl3
1.00
1.00
0.99
1.07
2.09
1.12
2.91
4.04
2.00
3.82
2.07
2.00
f1 (ppm)

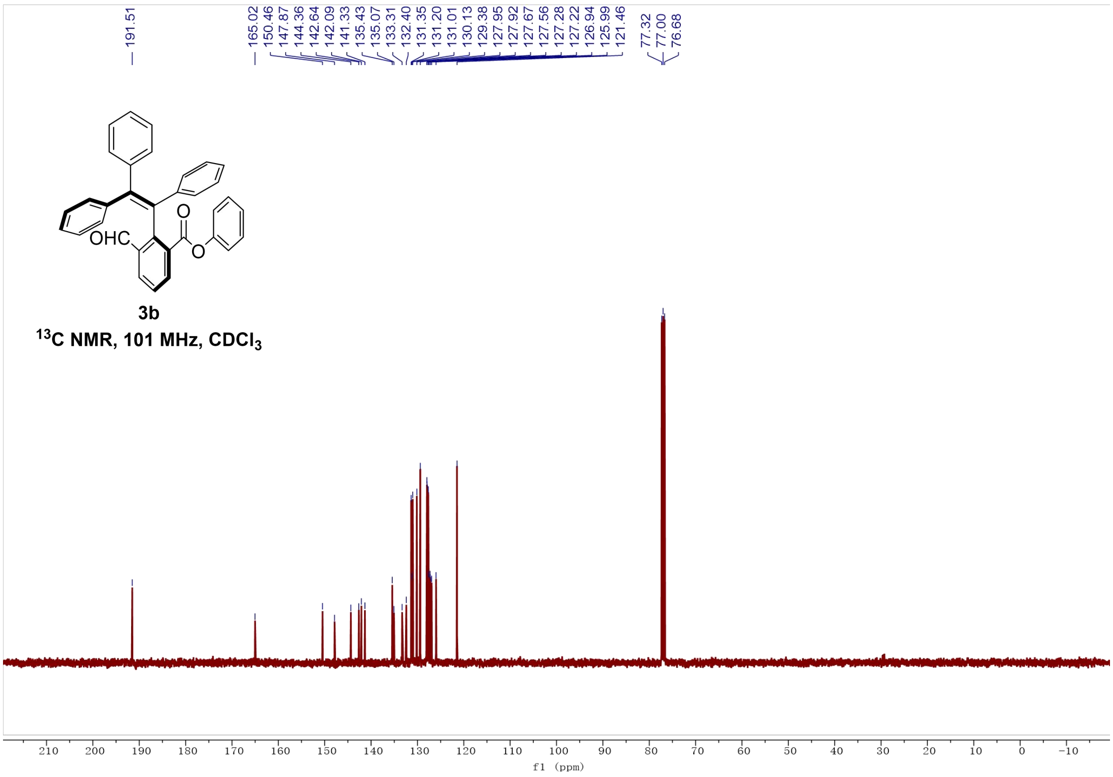
191.51
165.02
150.46
147.87
144.36
142.64
142.09
141.33
135.43
135.07
133.31
132.40
131.35
131.20
131.01
130.13
129.38
127.95
127.92
127.67
127.56
127.28
127.22
126.94
125.99
121.46
77.32
77.00
76.68
OHC
O
O
3b
13C NMR, 101 MHz, CDCl3
f1 (ppm)

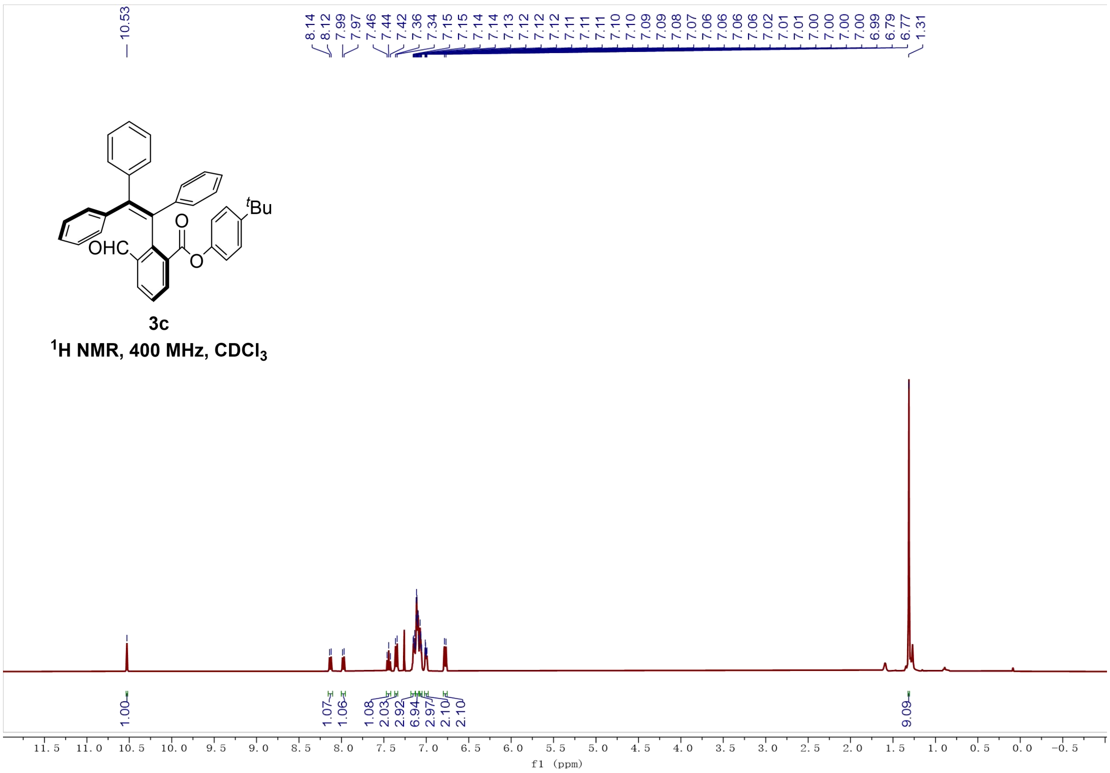
10.53
8.14
8.12
7.99
7.97
7.46
7.44
7.42
7.36
7.34
7.15
7.15
7.14
7.14
7.13
7.12
7.12
7.11
7.11
7.11
7.10
7.09
7.08
7.07
7.06
7.06
7.06
7.02
7.01
7.00
7.00
7.00
6.99
6.79
6.77
1.31
OHC
O
O
tBu
3c
1H NMR, 400 MHz, CDCl3
1.00
1.07
1.06
1.08
2.03
2.92
6.94
2.97
2.10
2.10
9.09
f1 (ppm)

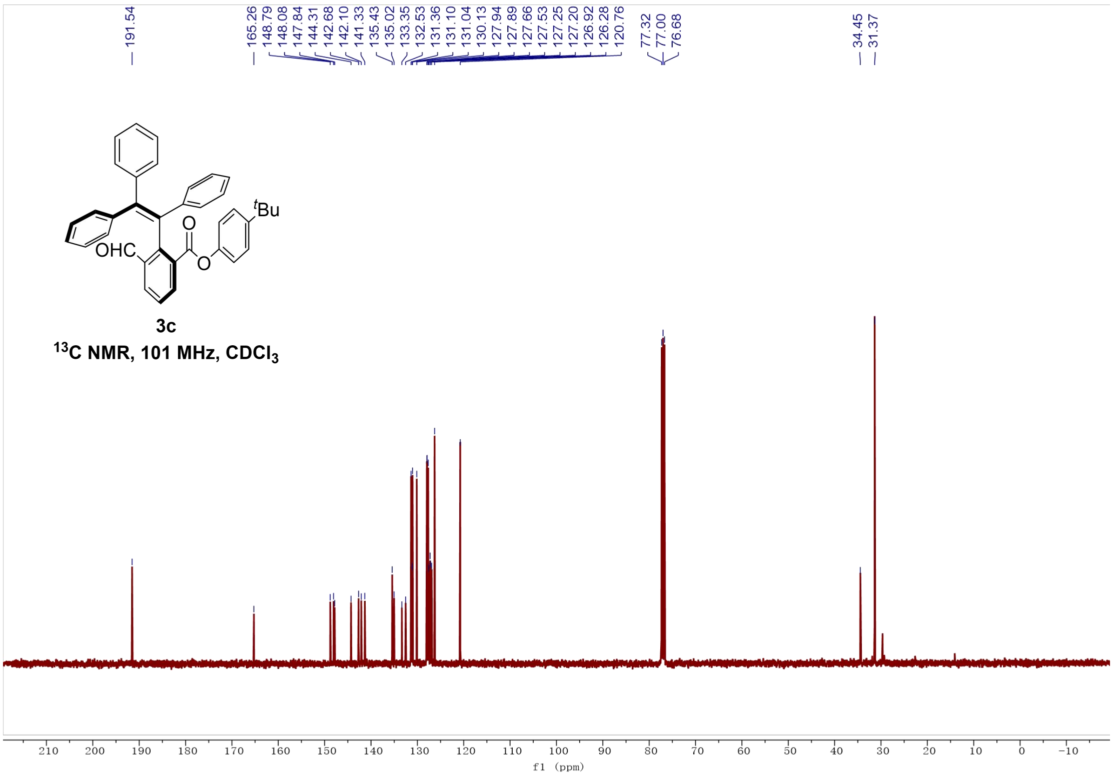
191.54
165.26
148.79
148.08
147.84
144.31
142.68
142.10
141.33
135.43
135.02
133.35
132.53
131.36
131.10
131.04
130.13
127.94
127.89
127.66
127.53
127.25
127.20
126.92
126.28
120.76
77.32
77.00
76.68
34.45
31.37
OHC
O
O
tBu
3c
13C NMR, 101 MHz, CDCl3
f1 (ppm)

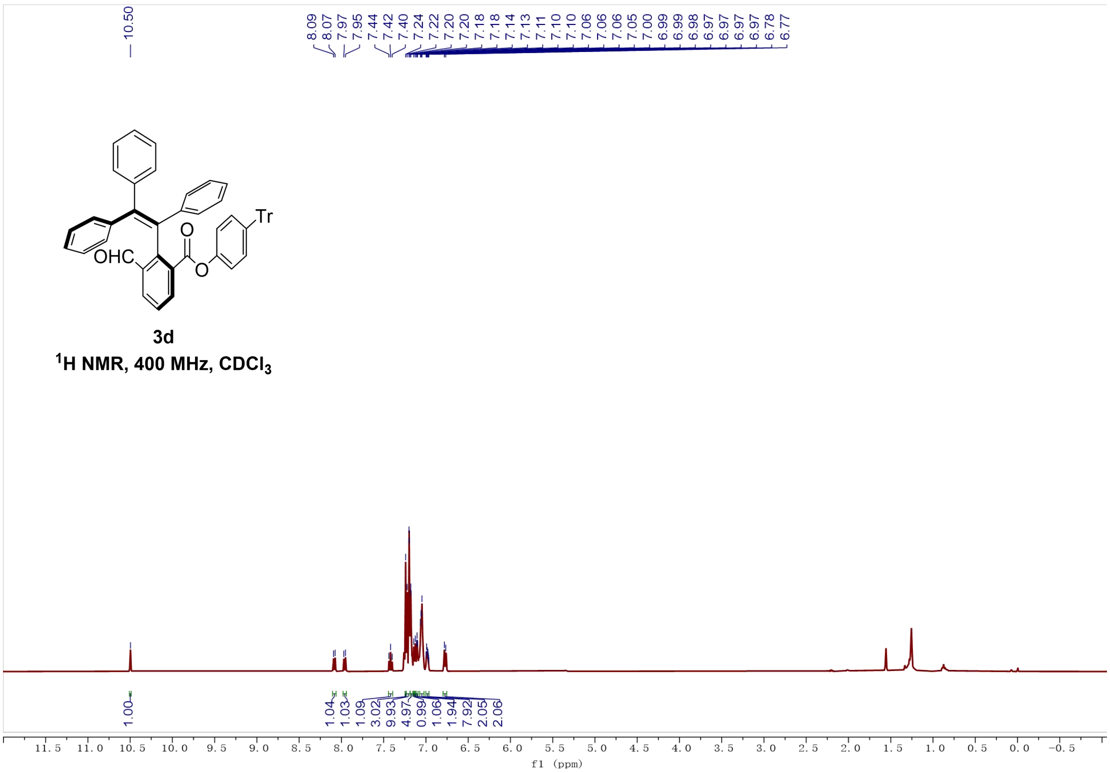
10.50
8.09 8.07 7.97 7.95 7.44 7.42 7.40 7.24 7.22 7.20 7.20 7.18 7.18 7.14 7.13 7.11 7.10 7.10 7.06 7.06 7.06 7.05 7.00 6.99 6.99 6.98 6.97 6.97 6.97 6.97 6.78 6.77
Tr
OHC
O
O
3d
1H NMR, 400 MHz, CDCl3
1.00 1.04 1.03 1.09 3.02 9.93 4.97 0.99 1.06 1.94 7.92 2.05 2.06
f1 (ppm)

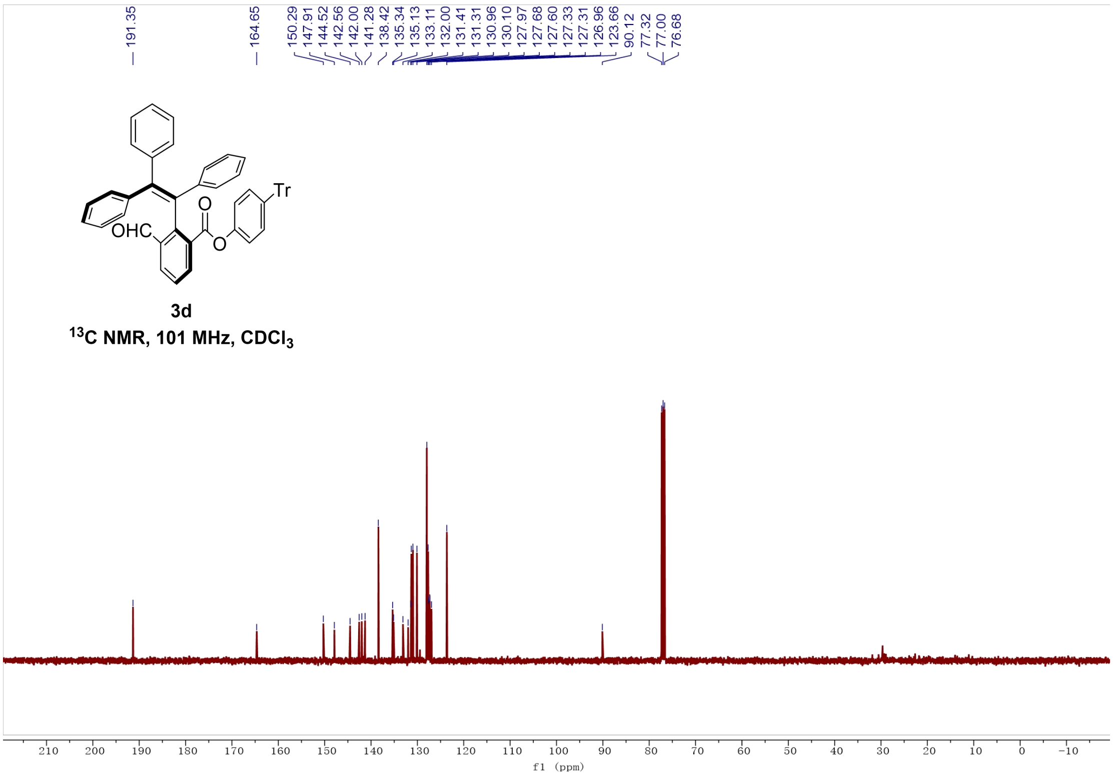
191.35
164.65
150.29 147.91 144.52 142.56 142.00 141.28 138.42 135.34 135.13 133.11 132.00 131.41 131.31 130.96 130.10 127.97 127.68 127.60 127.33 127.31 126.96 123.66 90.12 77.32 77.00 76.68
Tr
OHC
O
O
3d
13C NMR, 101 MHz, CDCl3
f1 (ppm)

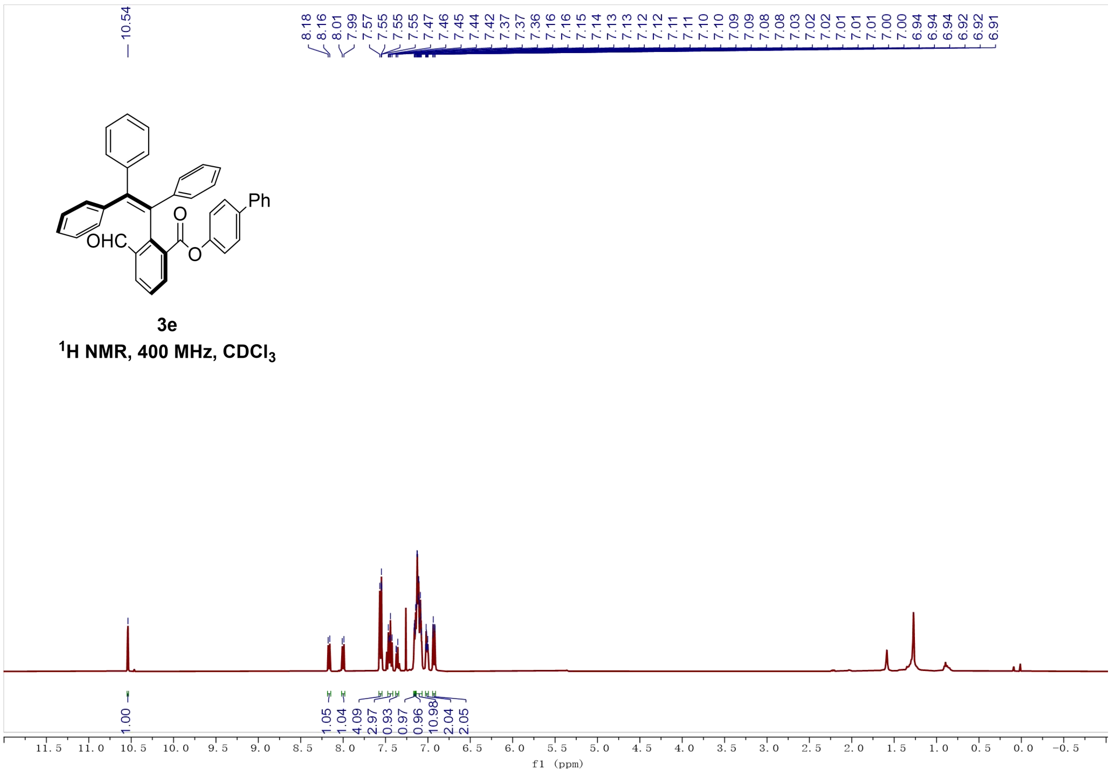
10.54
8.18
8.16
8.01
7.99
7.57
7.55
7.55
7.55
7.47
7.46
7.45
7.44
7.42
7.37
7.37
7.36
7.16
7.16
7.15
7.14
7.13
7.13
7.12
7.12
7.11
7.11
7.10
7.09
7.09
7.08
7.08
7.03
7.02
7.01
7.01
7.01
7.00
7.00
6.94
6.94
6.92
6.92
6.91
Ph
OHC
O
3e
1H NMR, 400 MHz, CDCl3
1.00
1.05
1.04
4.09
2.97
0.93
0.97
0.96
10.98
2.04
2.05
f1 (ppm)

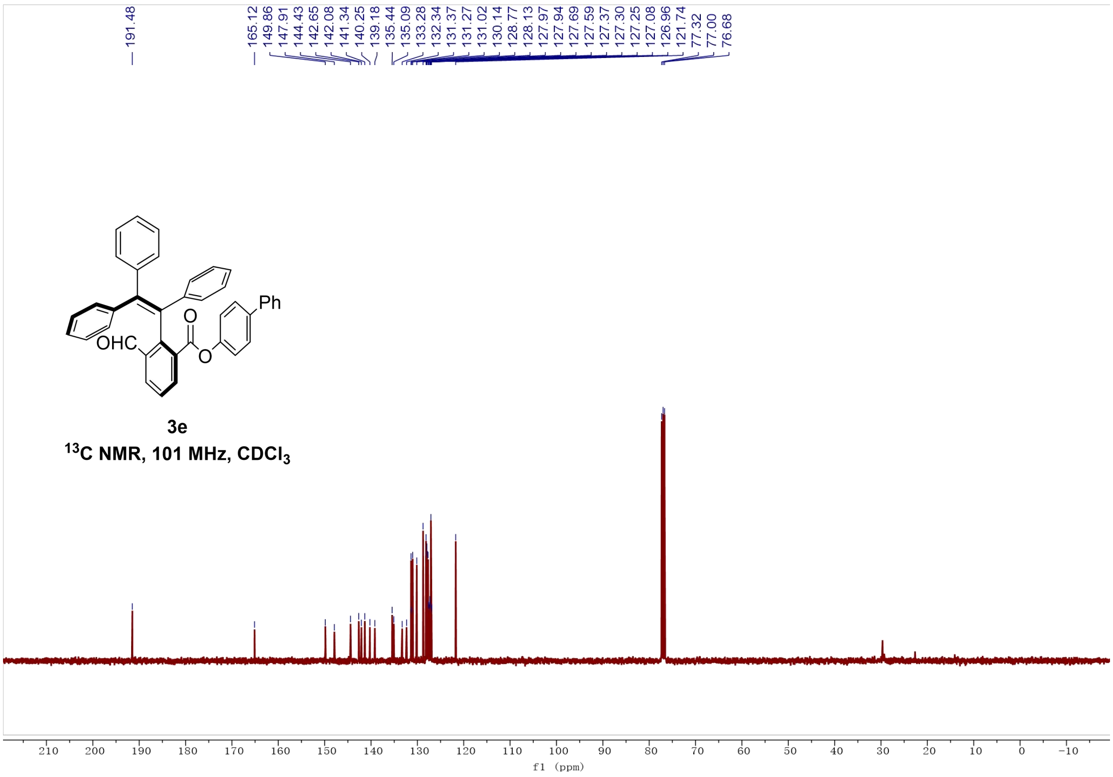
191.48
165.12
149.86
147.91
144.43
142.65
142.08
141.34
140.25
139.18
135.44
135.09
133.28
132.34
131.37
131.27
131.02
130.14
128.77
128.13
127.97
127.94
127.69
127.59
127.37
127.30
127.25
127.08
126.96
121.74
77.32
77.00
76.68
Ph
OHC
O
3e
13C NMR, 101 MHz, CDCl3
f1 (ppm)

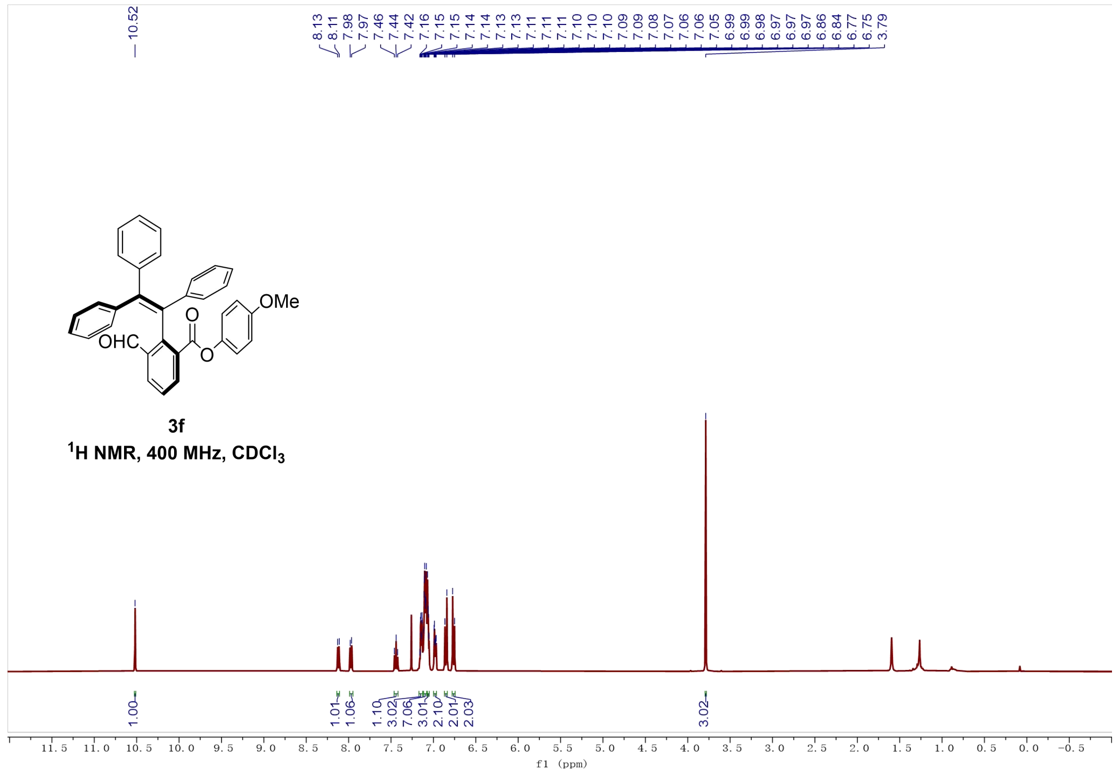
3f
1H NMR, 400 MHz, CDCl3
f1 (ppm)

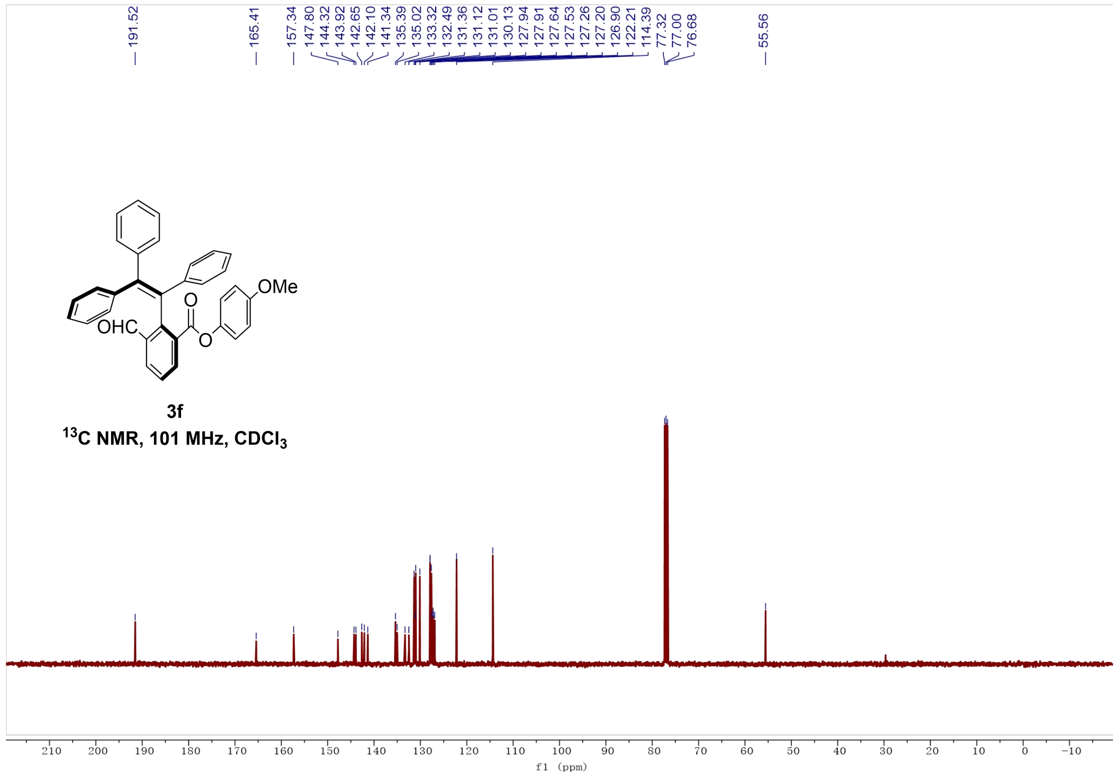
3f
13C NMR, 101 MHz, CDCl3
f1 (ppm)

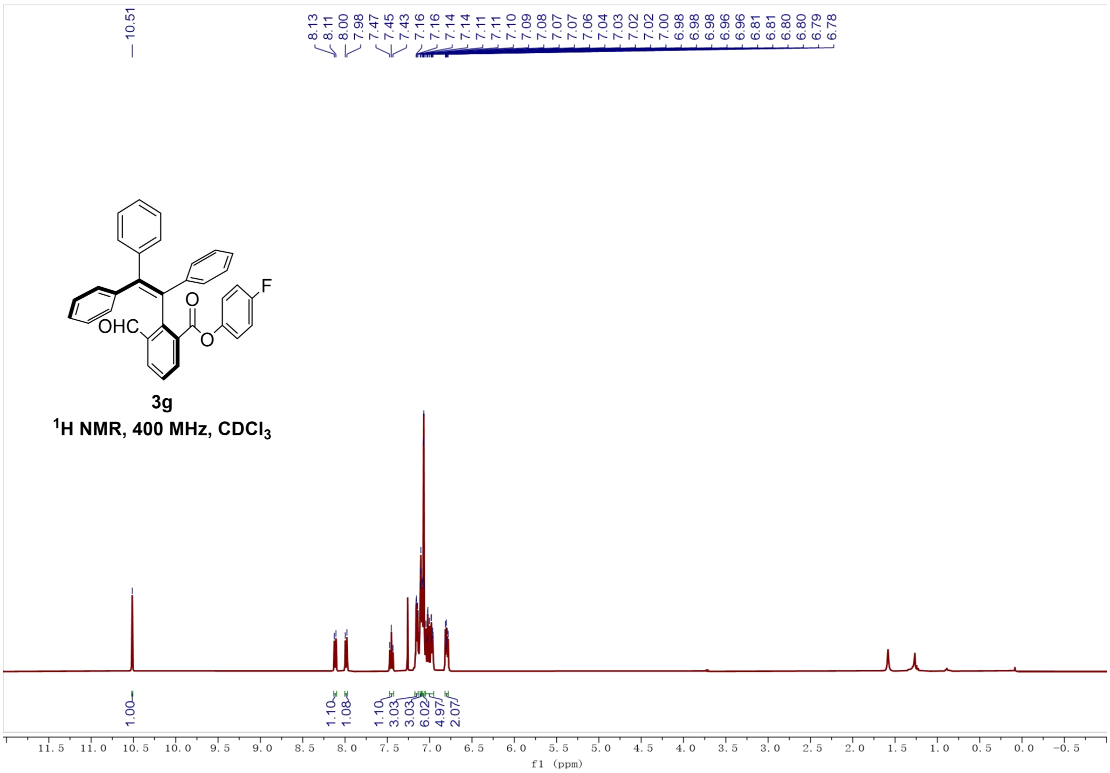
10.51
8.13 8.11 8.00 7.98 7.47 7.45 7.43 7.16 7.16 7.14 7.14 7.11 7.11 7.10 7.09 7.08 7.07 7.07 7.06 7.04 7.03 7.02 7.00 6.98 6.98 6.98 6.96 6.96 6.81 6.80 6.80 6.79 6.78
OHC
O
O
F
3g
¹H NMR, 400 MHz, CDCl₃
1.00 1.10 1.08 1.10 3.03 3.03 6.02 4.97 2.07
11.5 11.0 10.5 10.0 9.5 9.0 8.5 8.0 7.5 7.0 6.5 6.0 5.5 5.0 4.5 4.0 3.5 3.0 2.5 2.0 1.5 1.0 0.5 0.0 -0.5
f1 (ppm)

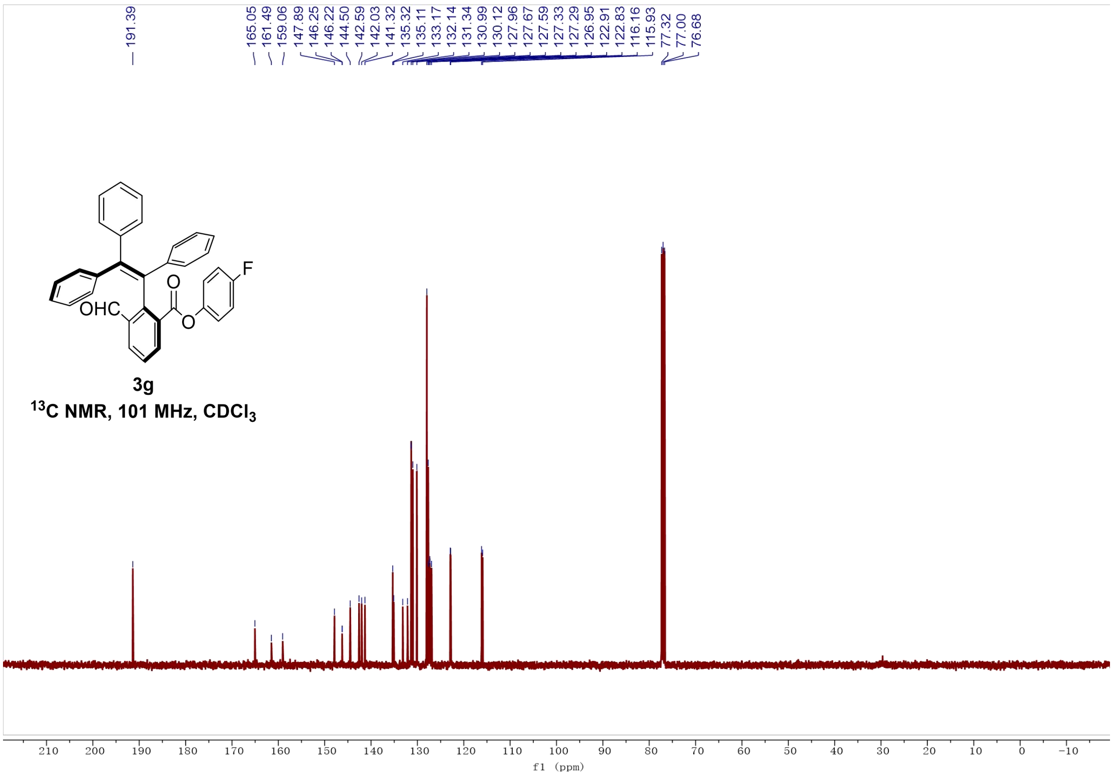
191.39
165.05 161.49 159.06 147.89 146.25 146.22 144.50 142.59 142.03 141.32 135.32 135.11 133.17 132.14 131.34 130.99 130.12 127.96 127.67 127.59 127.33 127.29 126.95 122.91 122.83 116.16 115.93 77.32 77.00 76.68
OHC
O
O
F
3g
¹³C NMR, 101 MHz, CDCl₃
210 200 190 180 170 160 150 140 130 120 110 100 90 80 70 60 50 40 30 20 10 0 -10
f1 (ppm)

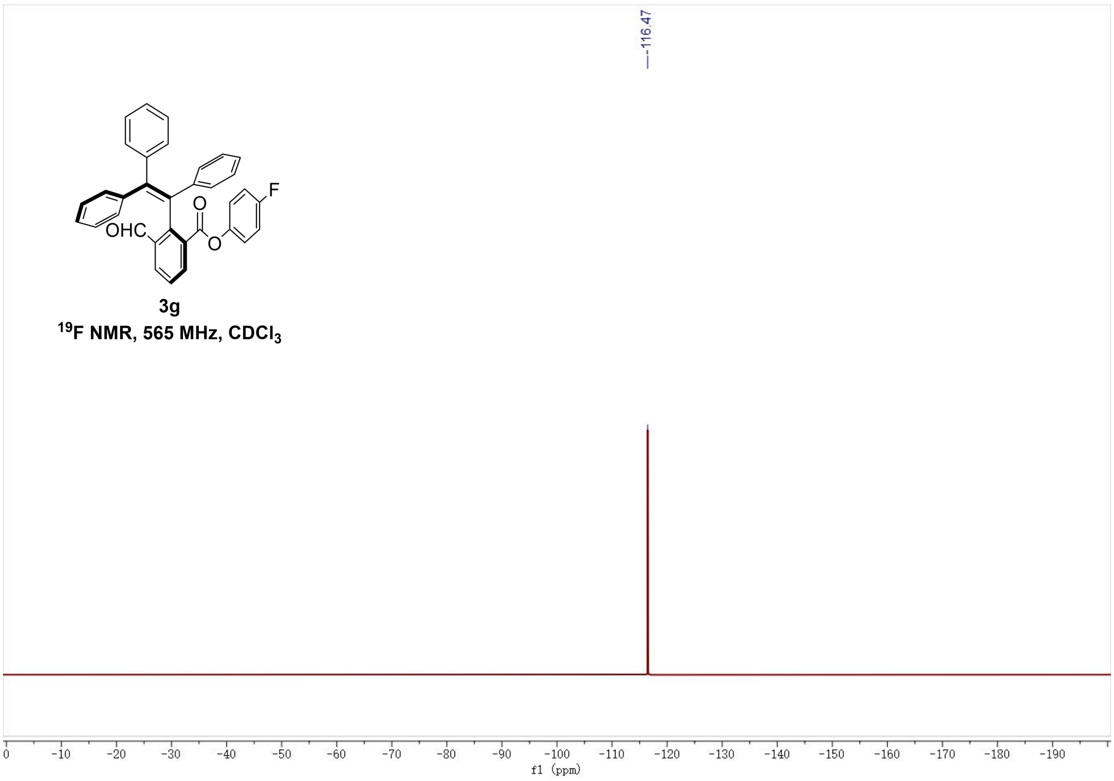
-116.47
OHC
O
F
3g
^{19}F NMR, 565 MHz, $CDCl_3$
f1 (ppm)

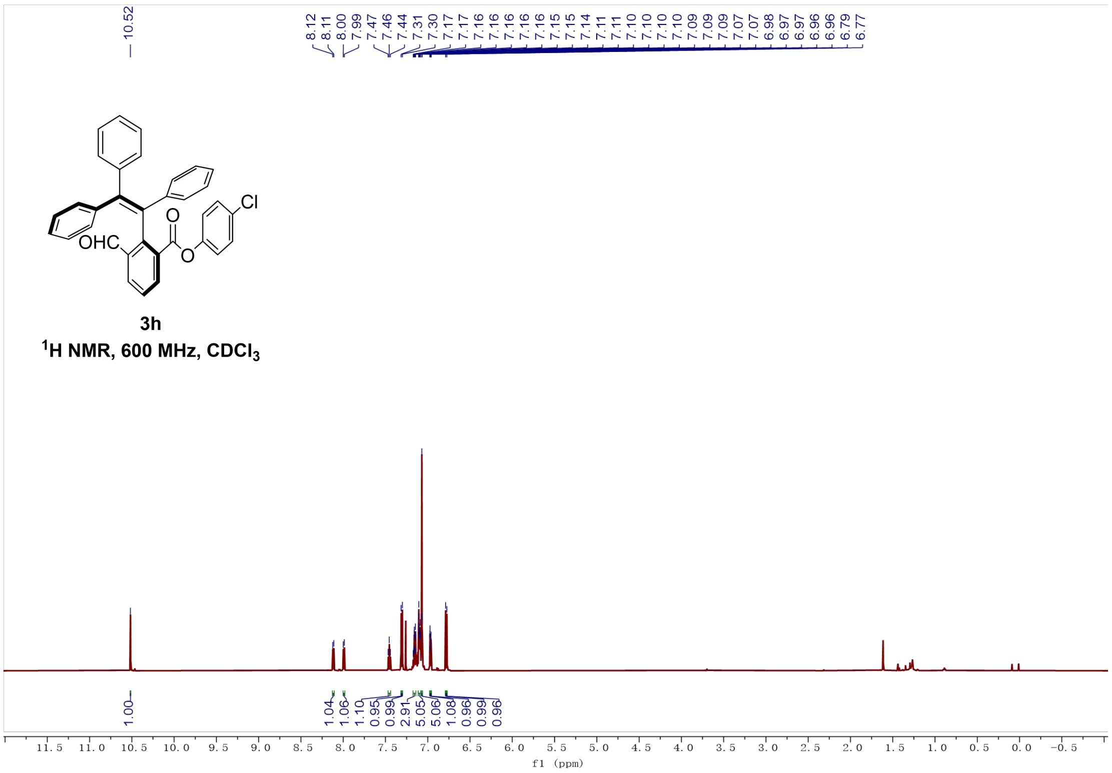
10.52
OHC
O
Cl
3h
^{1}H NMR, 600 MHz, $CDCl_3$
f1 (ppm)

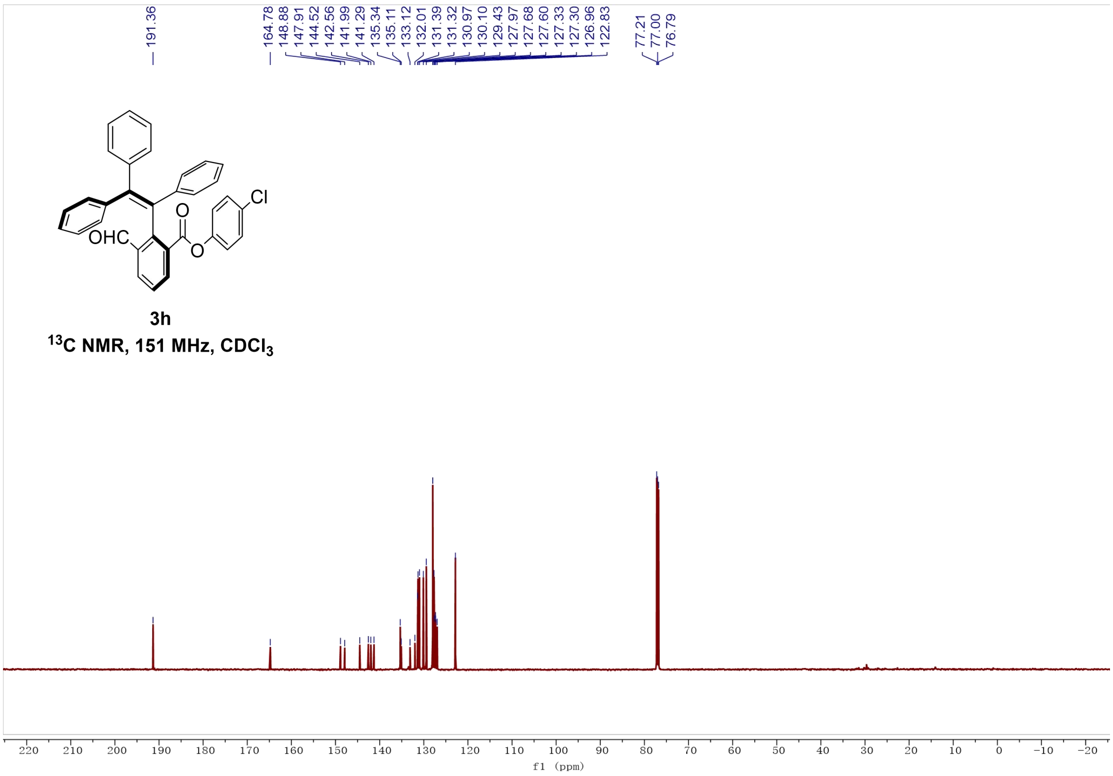
191.36
164.78
148.88
147.91
144.52
142.56
141.99
141.29
135.34
135.11
133.12
132.01
131.39
131.32
130.97
130.10
129.43
127.97
127.68
127.60
127.33
127.30
126.96
122.83
77.21
77.00
76.79
OHC
O
O
Cl
3h
^{13}C NMR, 151 MHz, $CDCl_3$
f1 (ppm)

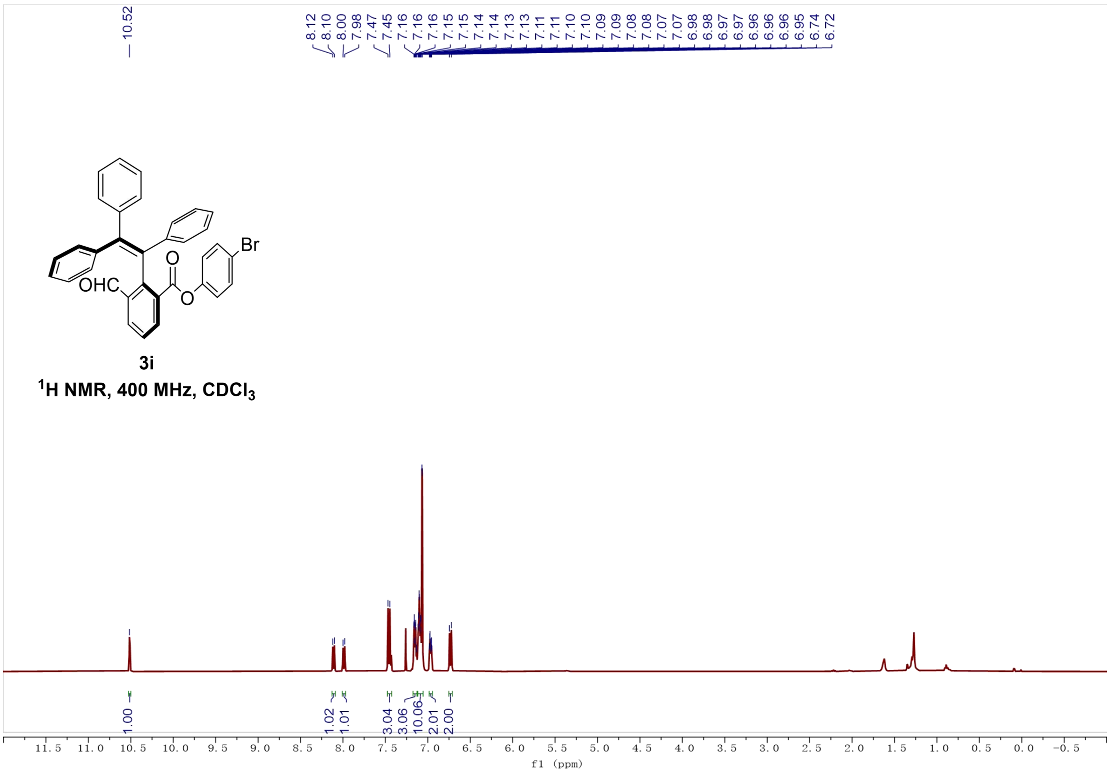
10.52
8.12
8.10
8.00
7.98
7.47
7.45
7.16
7.16
7.15
7.15
7.14
7.13
7.13
7.11
7.11
7.10
7.10
7.09
7.09
7.08
7.08
7.07
7.07
6.98
6.98
6.97
6.97
6.96
6.96
6.96
6.95
6.74
6.72
OHC
O
O
Br
3i
^{1}H NMR, 400 MHz, $CDCl_3$
1.00
1.02
1.01
3.04
3.06
10.06
2.01
2.00
f1 (ppm)

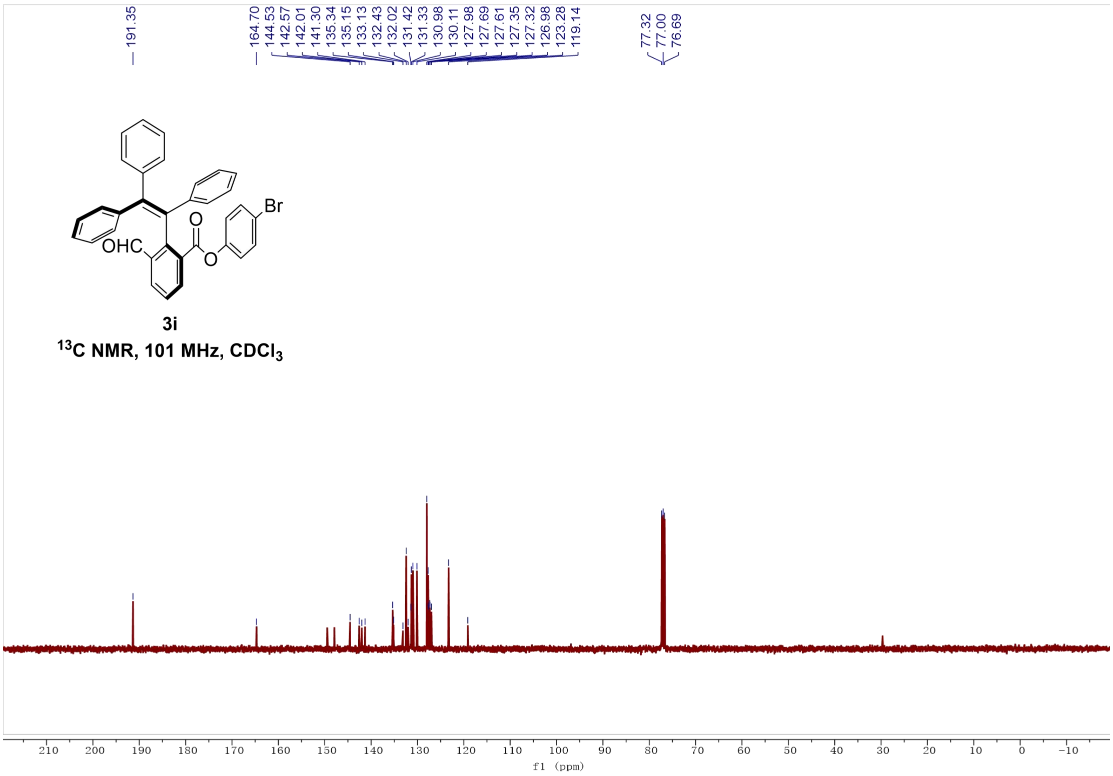

3i

^{13}C NMR, 101 MHz, CDCl$_3$

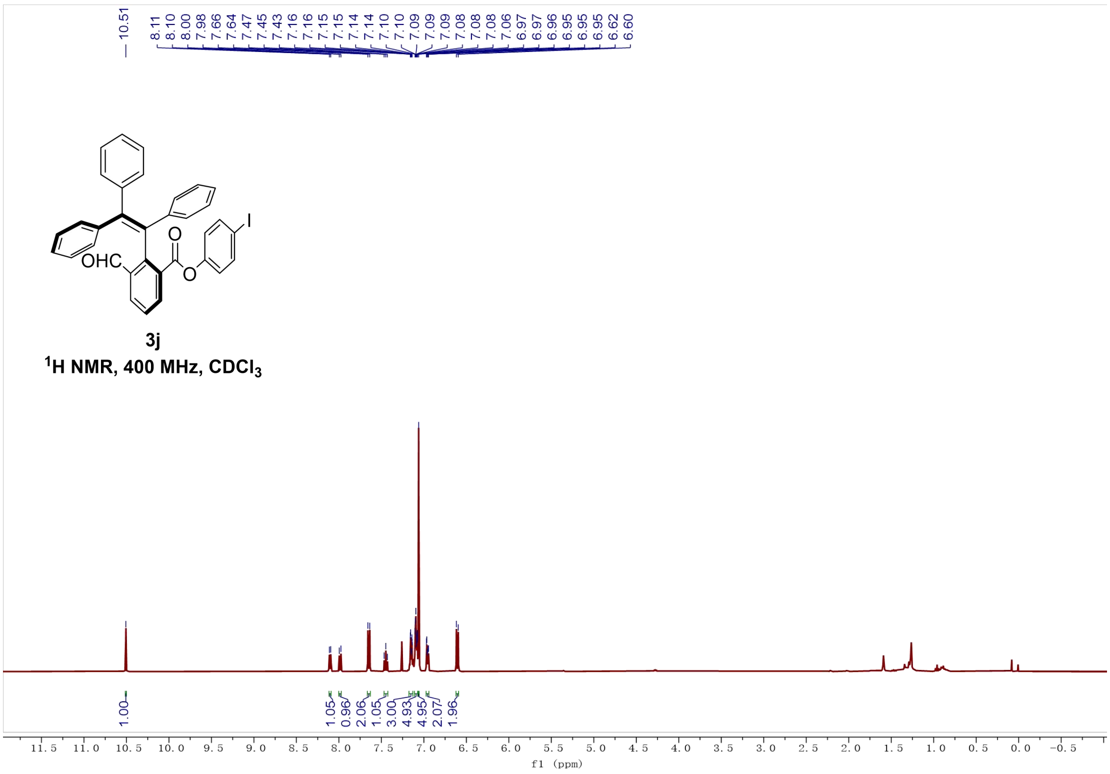

3j

^{1}H NMR, 400 MHz, CDCl$_3$

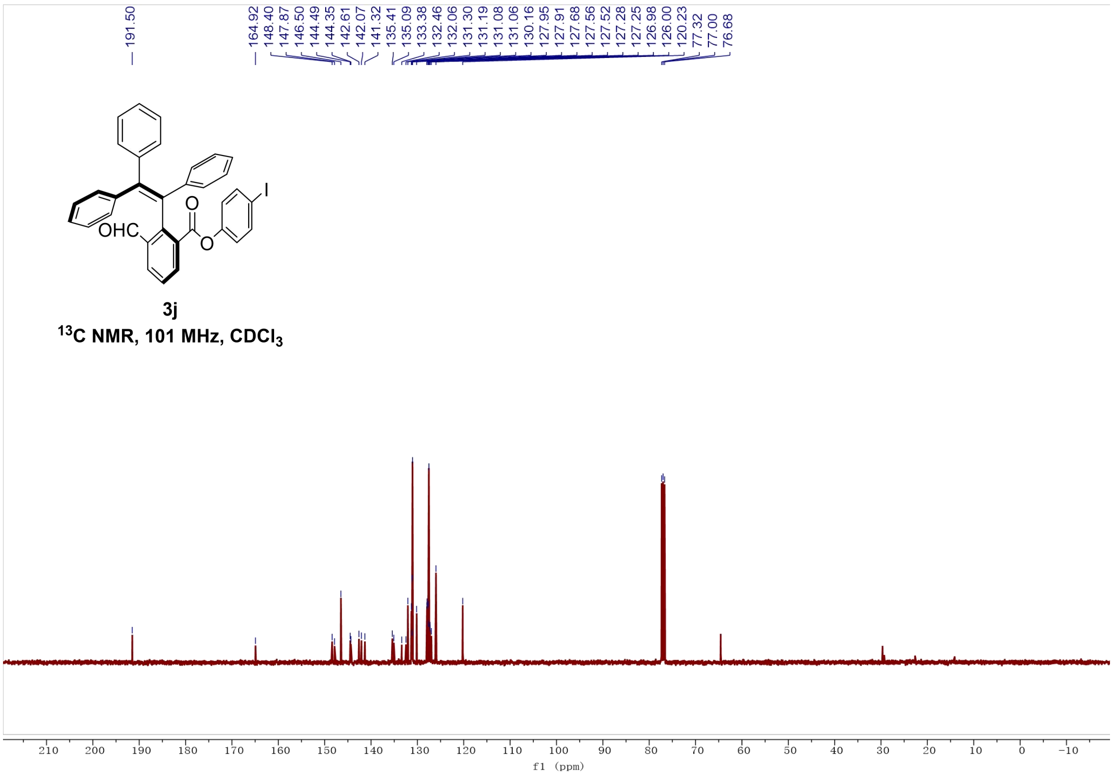

3j
^{13}C NMR, 101 MHz, $CDCl_3$

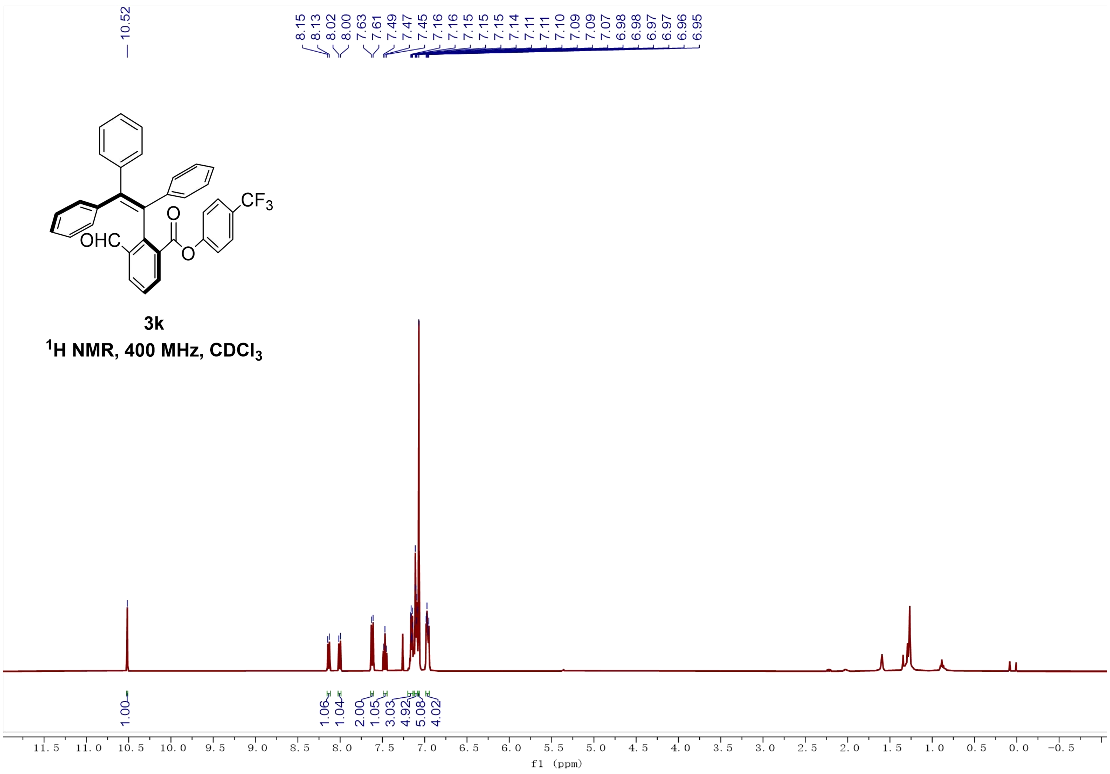

3k
^{1}H NMR, 400 MHz, $CDCl_3$

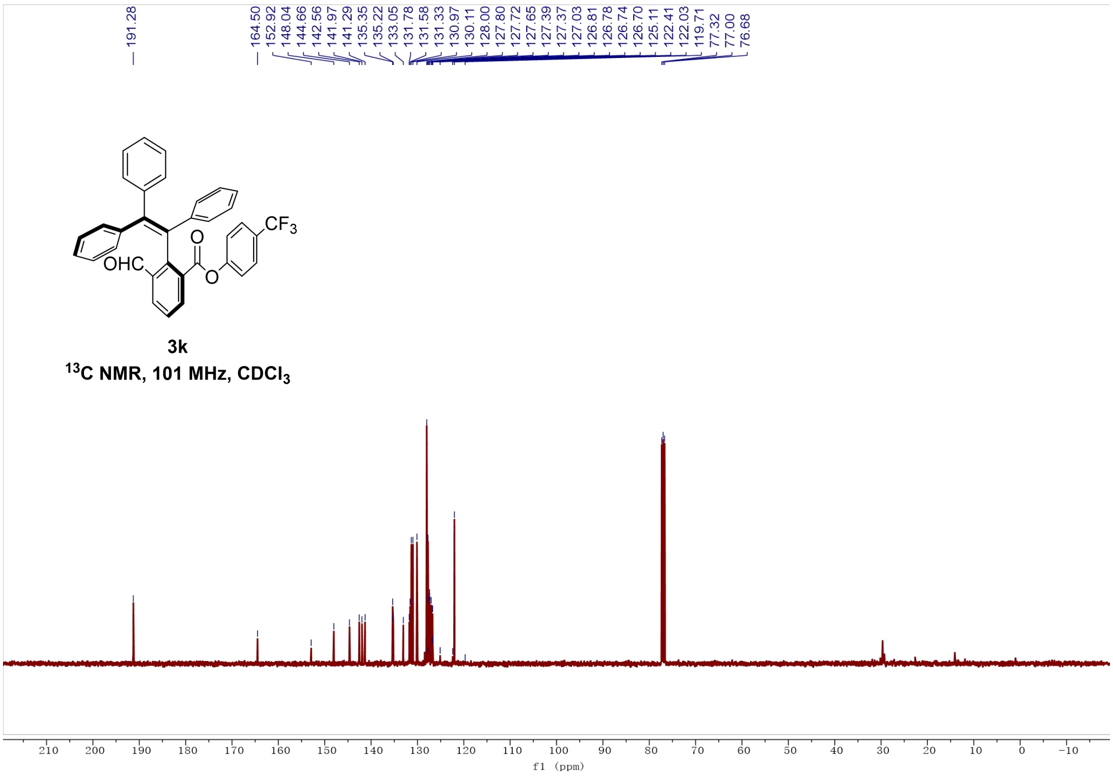
191.28
164.50
152.92
148.04
144.66
142.56
141.97
141.29
135.35
135.22
133.05
131.78
131.58
131.33
130.97
130.11
128.00
127.80
127.72
127.65
127.39
127.37
127.03
126.81
126.78
126.74
126.70
125.11
122.41
122.03
119.71
77.32
77.00
76.68
OHC
O
O
CF3
3k
13C NMR, 101 MHz, CDCl3
f1 (ppm)

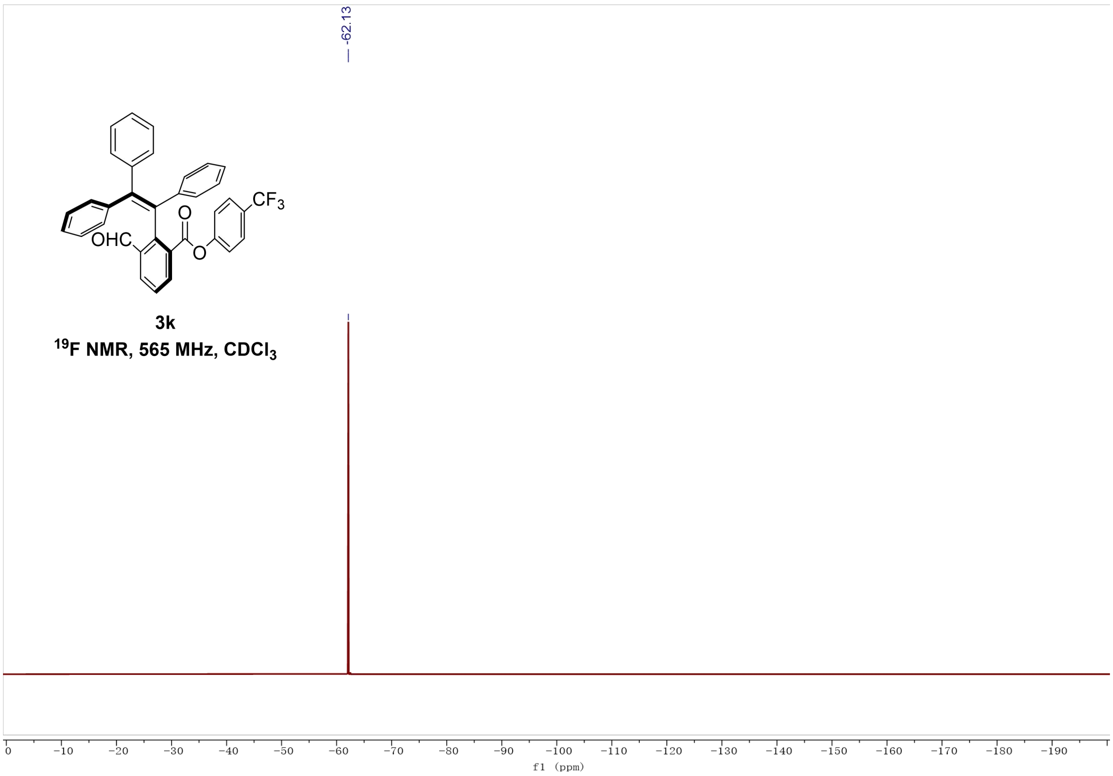
-62.13
OHC
O
O
CF3
3k
19F NMR, 565 MHz, CDCl3
f1 (ppm)

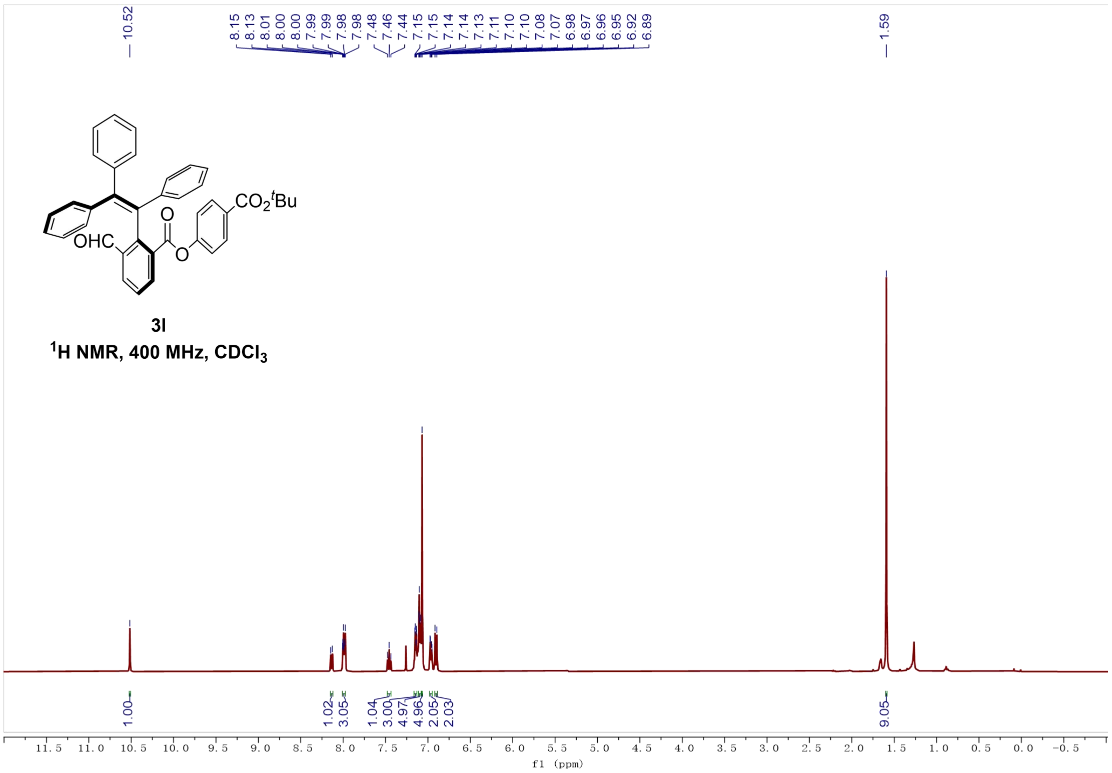

3l
^{1}H NMR, 400 MHz, CDCl$_3$

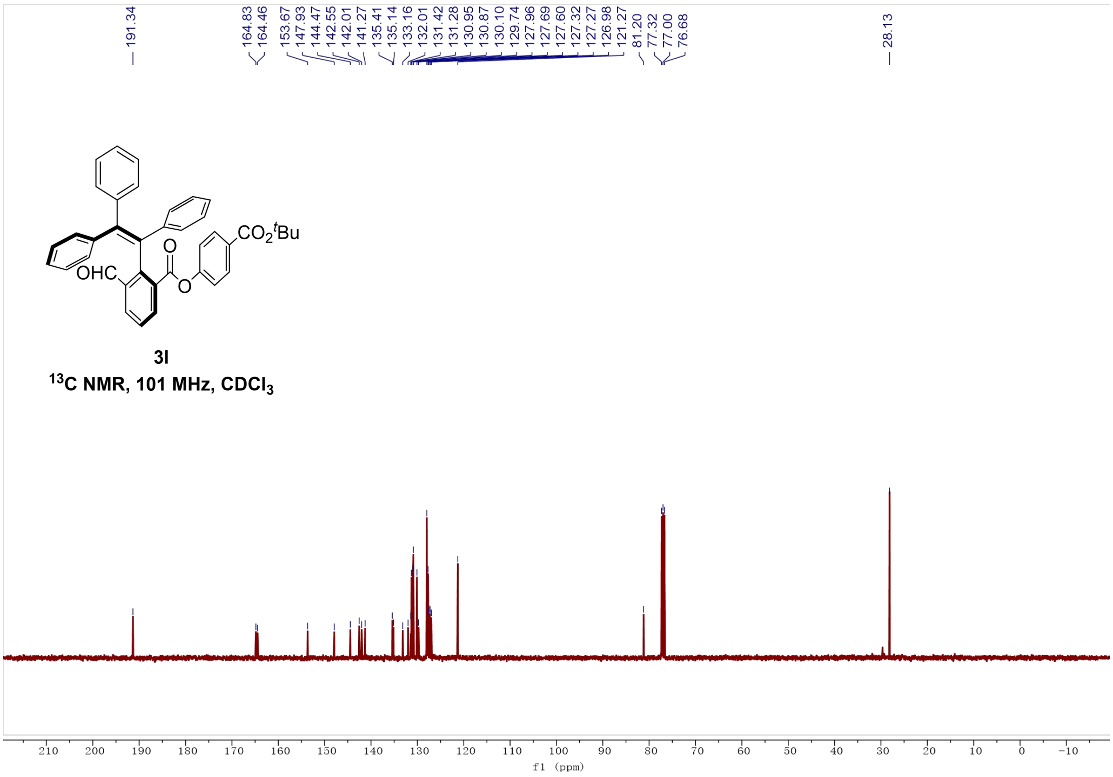

3l
^{13}C NMR, 101 MHz, CDCl$_3$

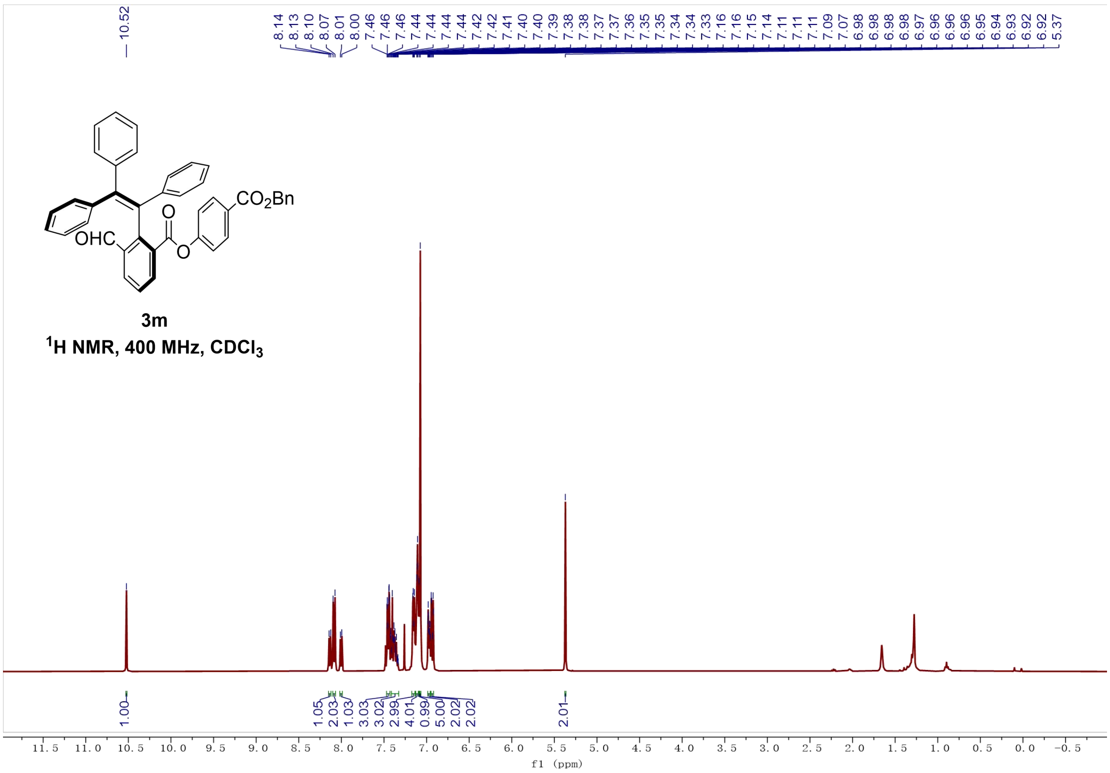

10.52
8.14
8.13
8.10
8.07
8.01
8.00
7.46
7.46
7.44
7.44
7.44
7.44
7.42
7.41
7.40
7.40
7.39
7.38
7.38
7.37
7.36
7.35
7.35
7.34
7.34
7.33
7.16
7.16
7.15
7.14
7.11
7.11
7.11
7.09
7.07
6.98
6.98
6.98
6.97
6.96
6.96
6.95
6.94
6.93
6.92
6.92
5.37
CO2Bn
O
OHC
O
3m
1H NMR, 400 MHz, CDCl3
1.00
1.05
2.03
1.03
3.03
3.02
2.99
4.01
0.99
5.00
2.02
2.02
2.01
f1 (ppm)

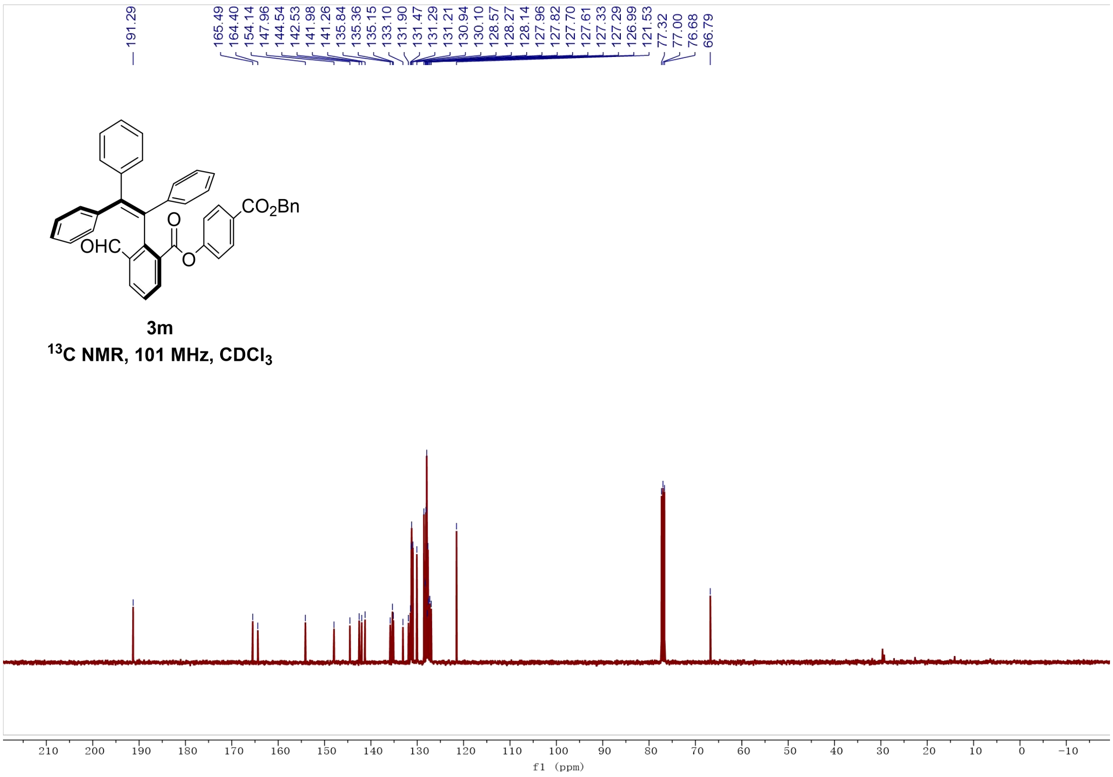

191.29
165.49
164.40
154.14
147.96
144.54
142.53
141.98
141.26
135.84
135.36
135.15
133.10
131.90
131.47
131.29
131.21
130.94
130.10
128.57
128.27
128.14
127.96
127.82
127.70
127.61
127.33
127.29
126.99
121.53
77.32
77.00
76.68
66.79
CO2Bn
O
OHC
O
3m
13C NMR, 101 MHz, CDCl3
f1 (ppm)

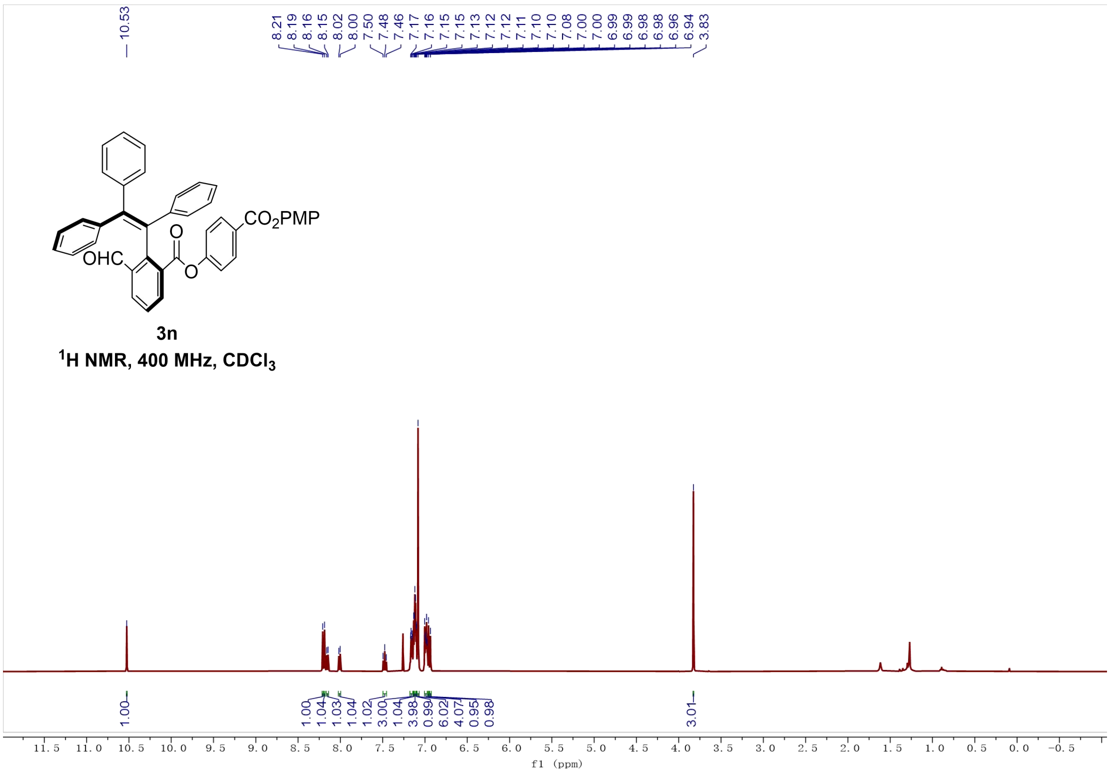
10.53
8.21 8.19 8.16 8.15 8.02 8.00 7.50 7.48 7.46 7.17 7.16 7.15 7.15 7.13 7.12 7.12 7.11 7.10 7.10 7.08 7.00 7.00 6.99 6.99 6.98 6.98 6.96 6.94 3.83
CO2PMP
O
OHC
O
3n
1H NMR, 400 MHz, CDCl3
1.00
1.00 1.04 1.03 1.04 1.02 3.00 1.04 3.98 0.99 6.02 4.07 0.95 0.98
3.01
11.5 11.0 10.5 10.0 9.5 9.0 8.5 8.0 7.5 7.0 6.5 6.0 5.5 5.0 4.5 4.0 3.5 3.0 2.5 2.0 1.5 1.0 0.5 0.0 -0.5
f1 (ppm)

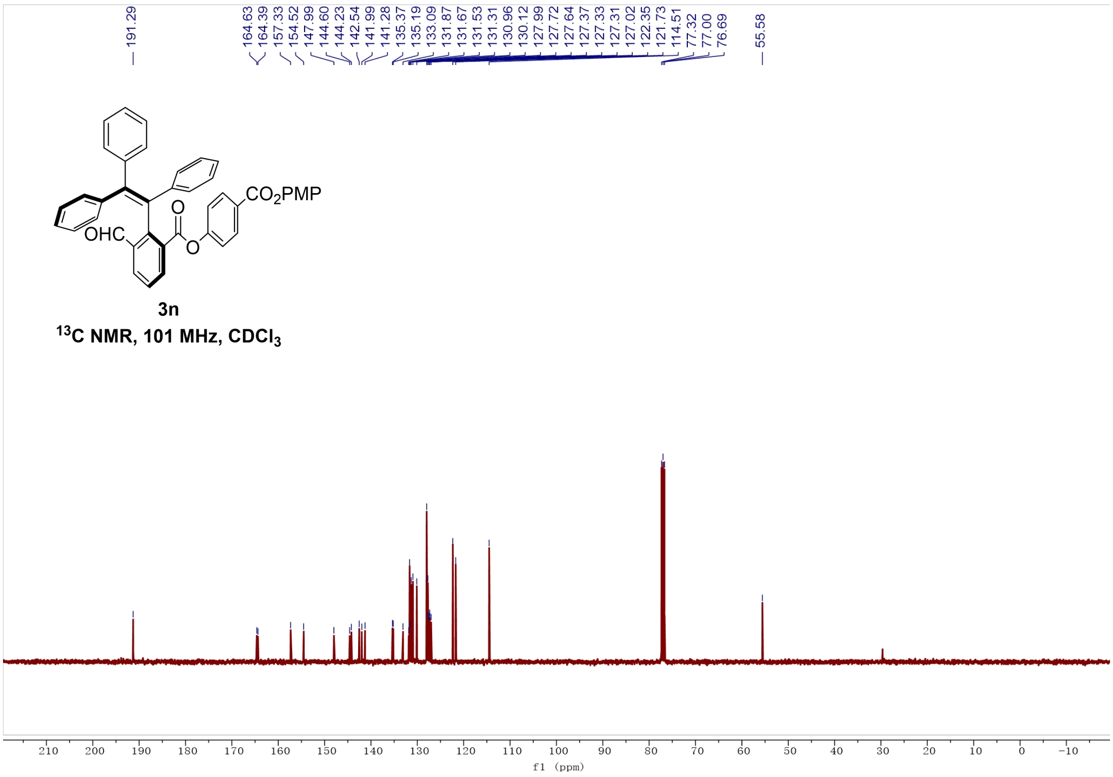
191.29
164.63 164.39 157.33 154.52 147.99 144.60 144.23 142.54 141.99 141.28 135.37 135.19 133.09 131.87 131.67 131.53 131.31 130.96 130.12 127.99 127.72 127.64 127.37 127.33 127.31 127.02 122.35 121.73 114.51 77.32 77.00 76.69
55.58
CO2PMP
O
OHC
O
3n
13C NMR, 101 MHz, CDCl3
210 200 190 180 170 160 150 140 130 120 110 100 90 80 70 60 50 40 30 20 10 0 -10
f1 (ppm)

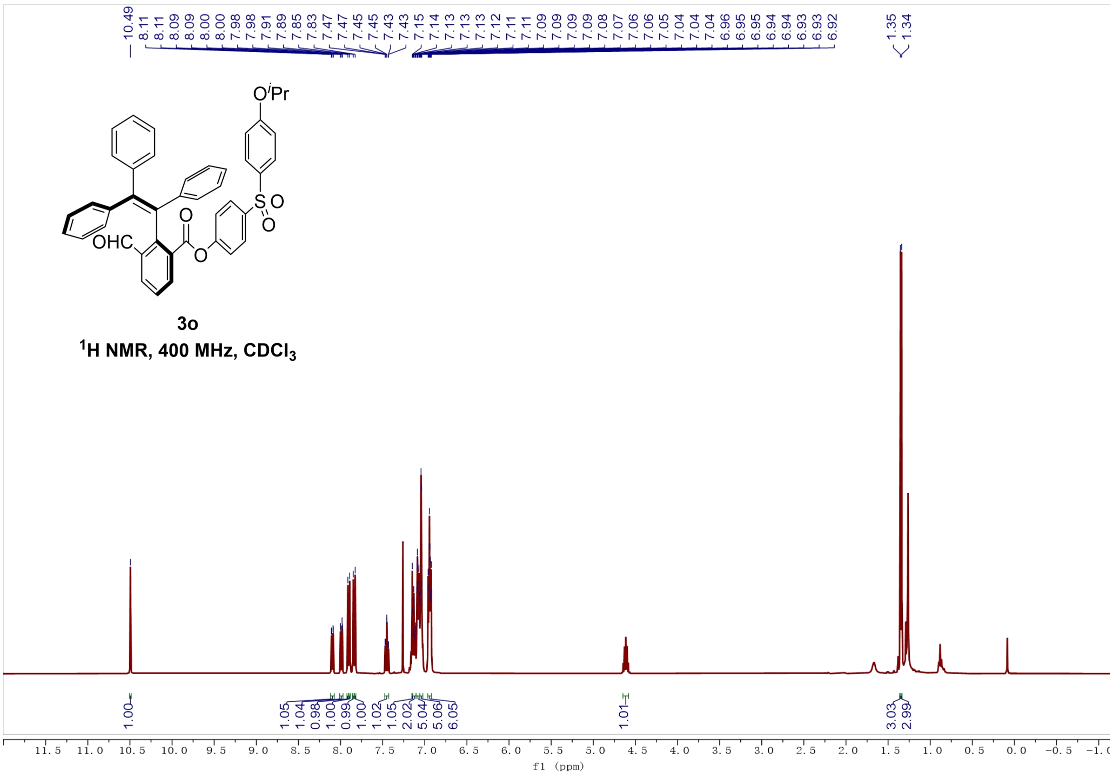
10.49
8.11
8.11
8.09
8.09
8.00
8.00
7.98
7.98
7.91
7.89
7.85
7.83
7.47
7.47
7.45
7.45
7.43
7.43
7.15
7.14
7.13
7.13
7.13
7.12
7.11
7.11
7.09
7.09
7.09
7.09
7.08
7.07
7.06
7.05
7.04
7.04
7.04
6.96
6.95
6.94
6.93
6.93
6.93
6.92
1.35
1.34
O^{i}Pr
OHC
O
O
S
O
O
3o
^{1}H NMR, 400 MHz, CDCl$_3$
1.00
1.05
1.04
0.98
1.00
0.99
1.00
1.02
1.05
2.02
5.04
5.06
6.05
1.01
3.03
2.99
f1 (ppm)

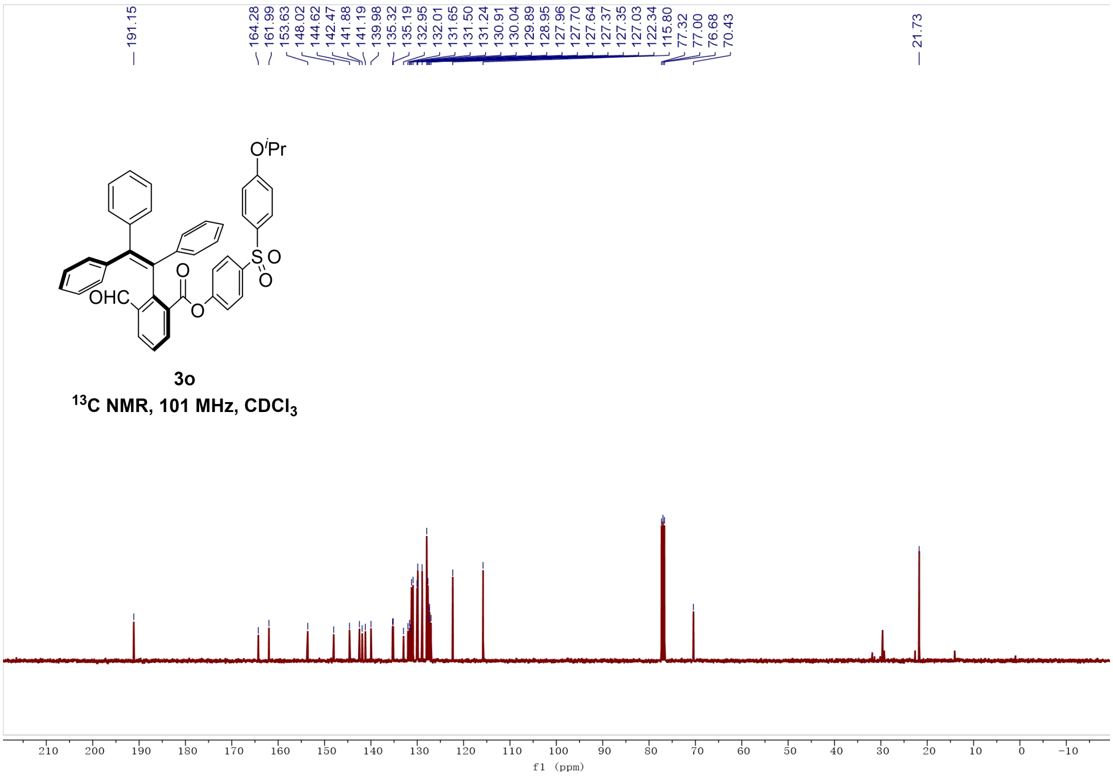
191.15
164.28
161.99
153.63
148.02
144.62
142.47
141.88
141.19
139.98
135.32
135.19
132.95
132.01
131.65
131.50
131.24
130.91
130.04
129.89
128.95
127.96
127.70
127.64
127.37
127.35
127.03
122.34
115.80
77.32
77.00
76.68
70.43
21.73
O^{i}Pr
OHC
O
O
S
O
O
3o
^{13}C NMR, 101 MHz, CDCl$_3$
f1 (ppm)

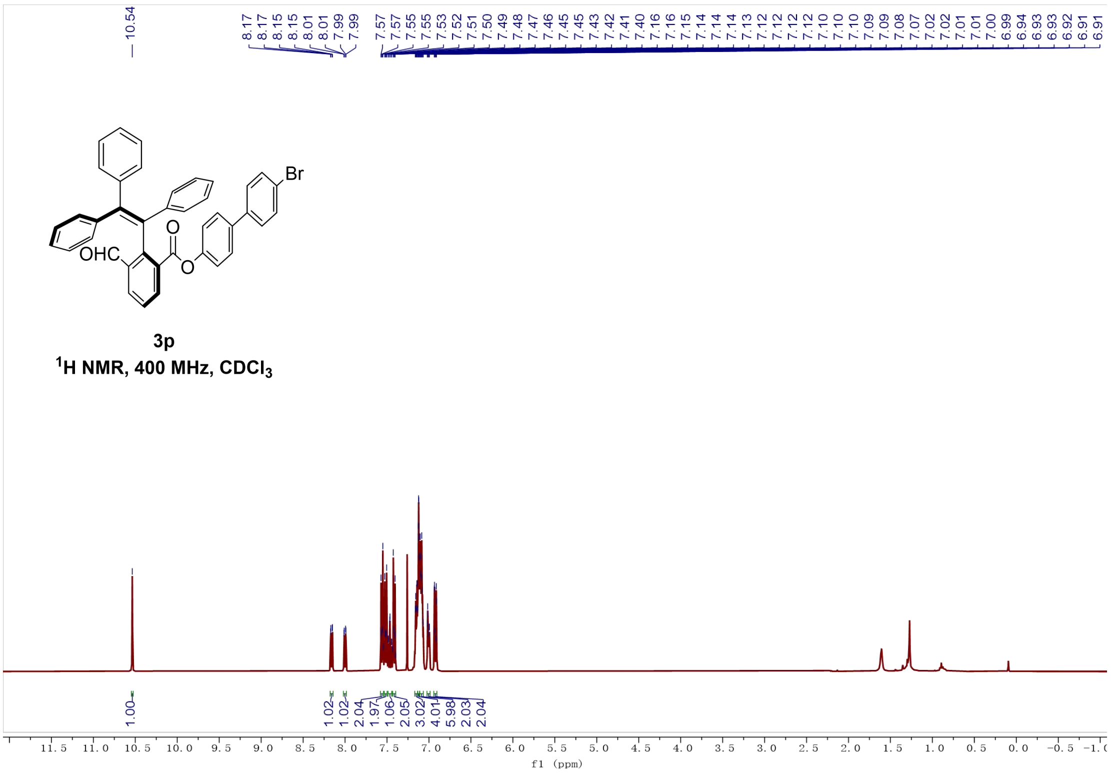

3p
^{1}H NMR, 400 MHz, $CDCl_3$

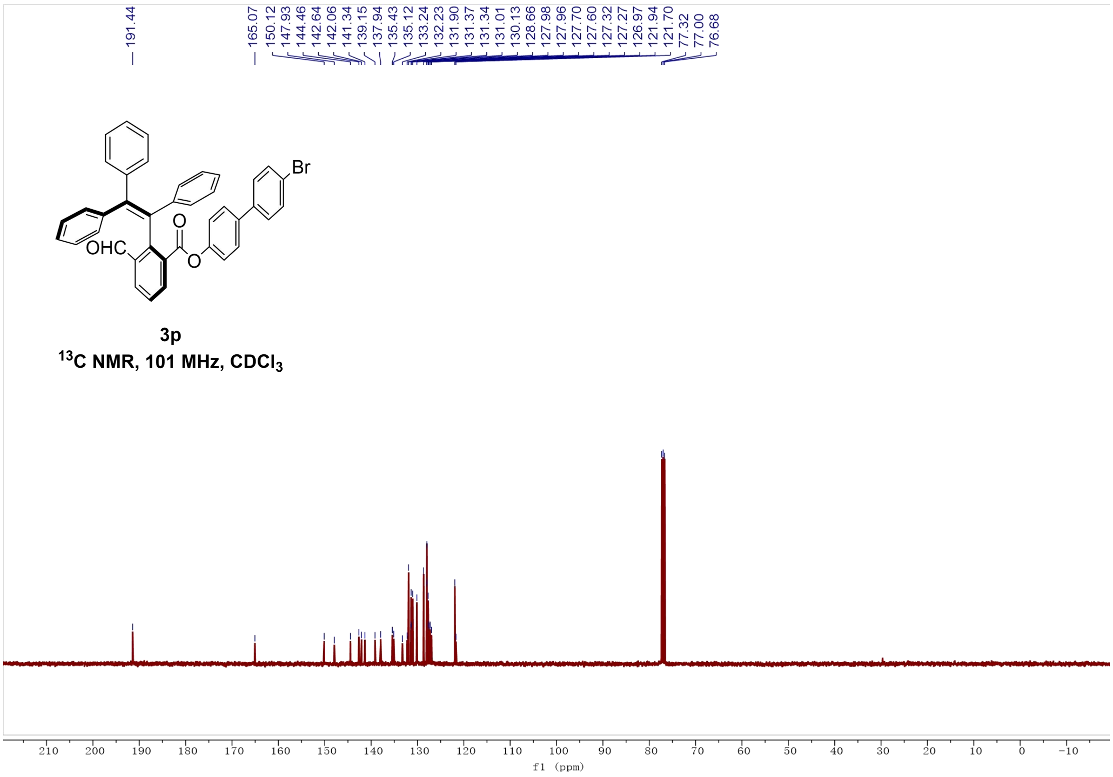

3p
^{13}C NMR, 101 MHz, $CDCl_3$

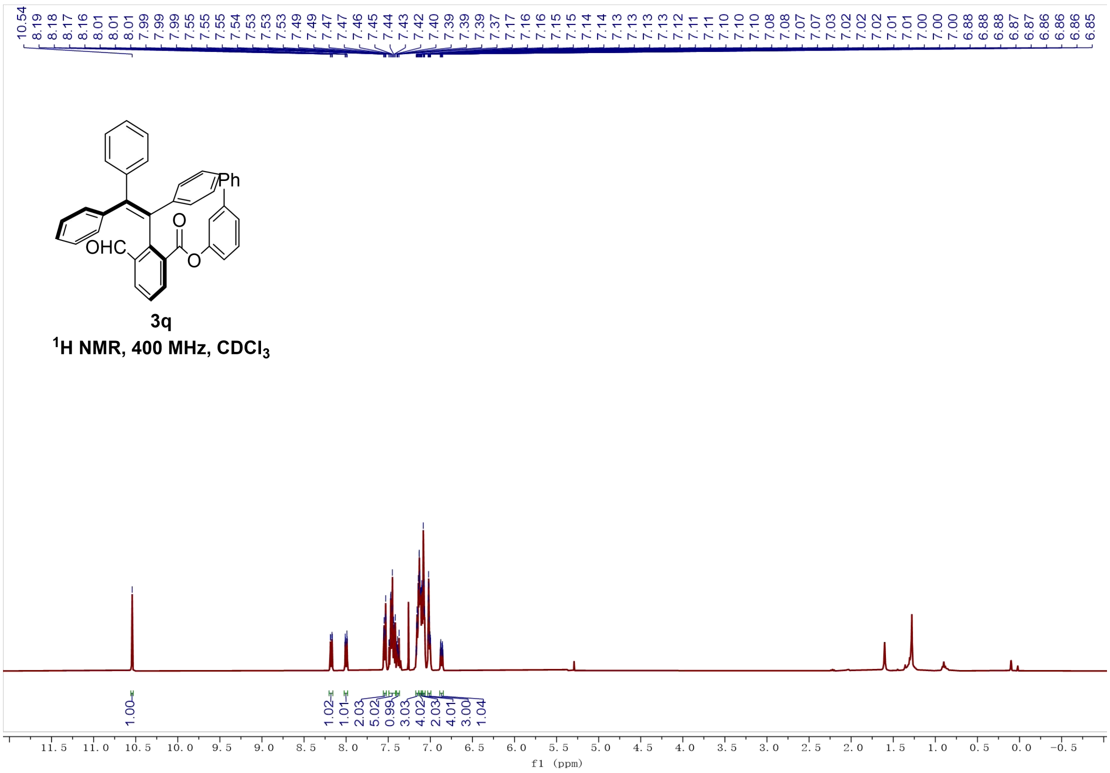
10.54 8.19 8.18 8.17 8.16 8.01 8.01 8.01 7.99 7.99 7.99 7.55 7.55 7.55 7.54 7.53 7.53 7.53 7.49 7.49 7.47 7.47 7.46 7.45 7.44 7.43 7.42 7.40 7.39 7.39 7.39 7.37 7.17 7.16 7.16 7.15 7.15 7.14 7.14 7.13 7.13 7.13 7.13 7.12 7.11 7.11 7.10 7.10 7.08 7.08 7.07 7.07 7.03 7.02 7.02 7.01 7.01 7.00 7.00 7.00 6.88 6.88 6.87 6.87 6.86 6.86 6.85
OHC
O
O
Ph
3q
^{1}H NMR, 400 MHz, CDCl$_3$
1.00 1.02 1.01 2.03 5.02 0.99 3.03 4.02 2.03 4.01 3.00 1.04
f1 (ppm)

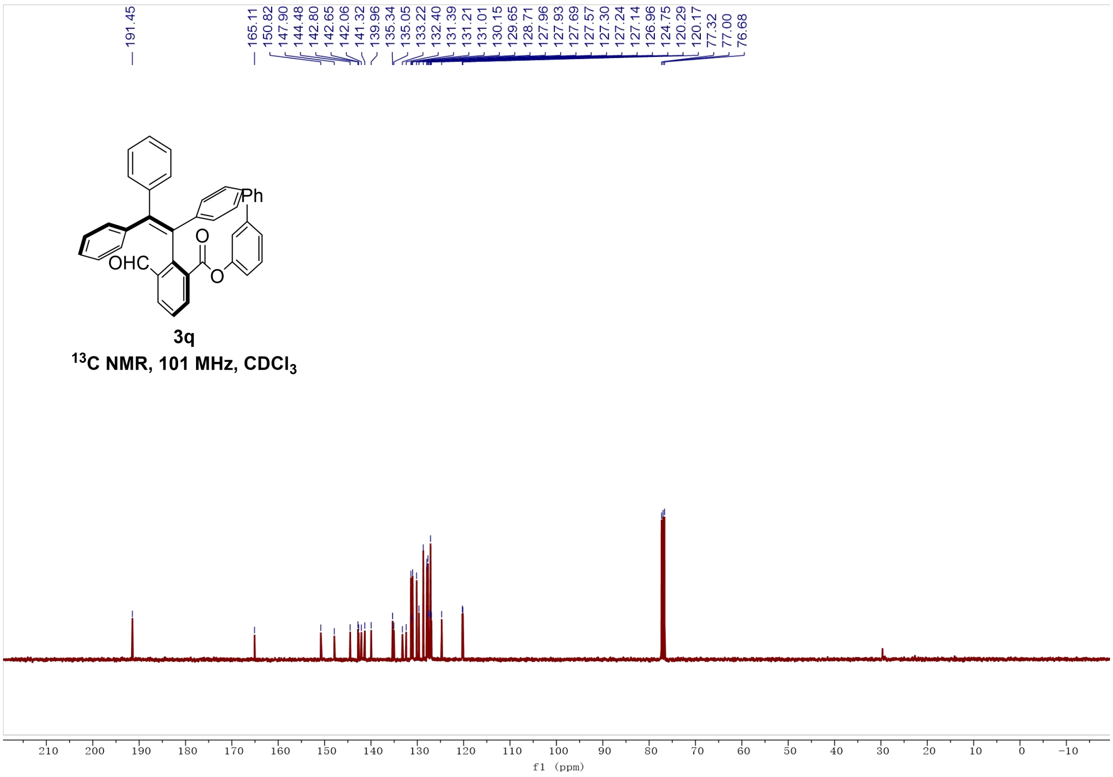
191.45
165.11 150.82 147.90 144.48 142.80 142.65 142.06 141.32 139.96 135.34 135.05 133.22 132.40 131.39 131.21 131.01 130.15 129.65 128.71 127.96 127.93 127.69 127.57 127.30 127.24 127.14 126.96 124.75 120.29 120.17 77.32 77.00 76.68
OHC
O
O
Ph
3q
^{13}C NMR, 101 MHz, CDCl$_3$
f1 (ppm)

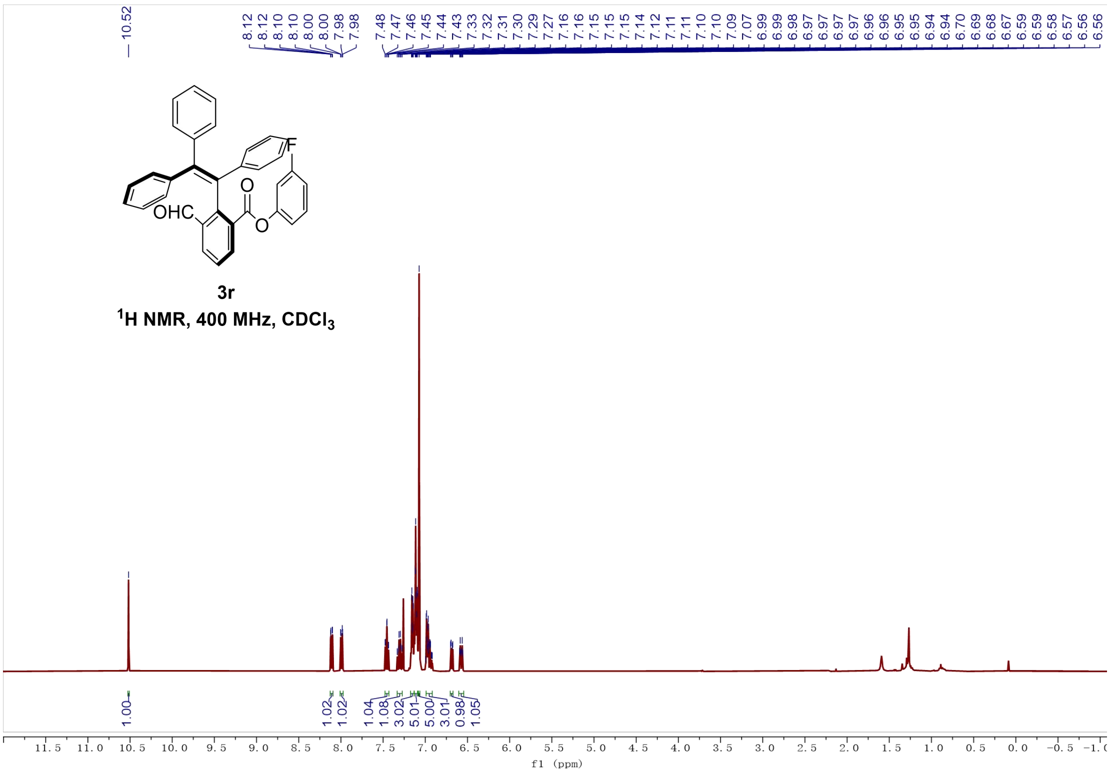

3r

^{1}H NMR, 400 MHz, CDCl$_3$

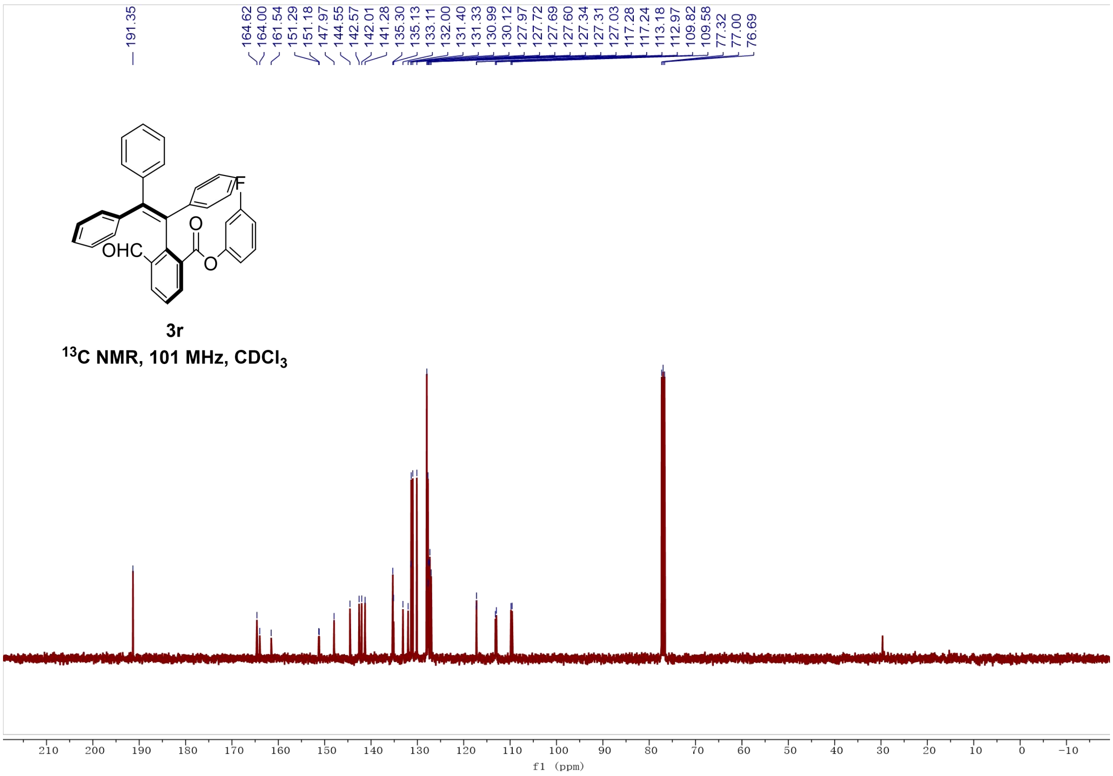

3r

^{13}C NMR, 101 MHz, CDCl$_3$

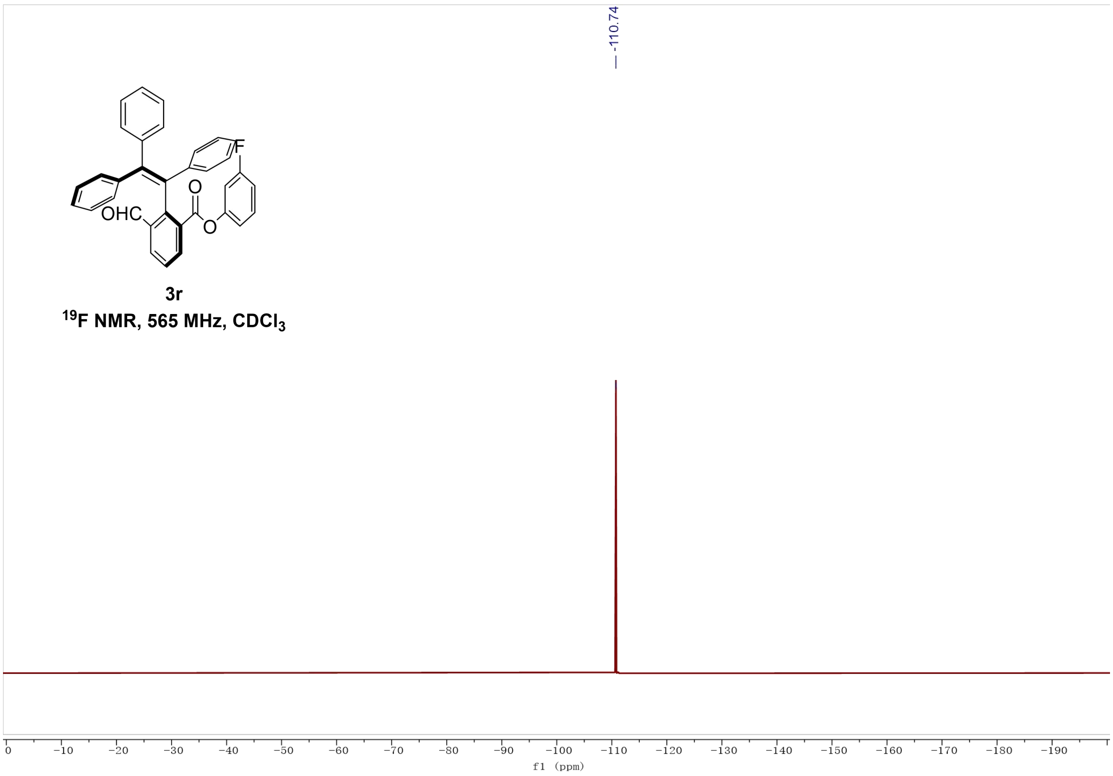
-110.74
OHC
O
F
3r
19F NMR, 565 MHz, CDCl3
0 −10 −20 −30 −40 −50 −60 −70 −80 −90 −100 −110 −120 −130 −140 −150 −160 −170 −180 −190
f1 (ppm)

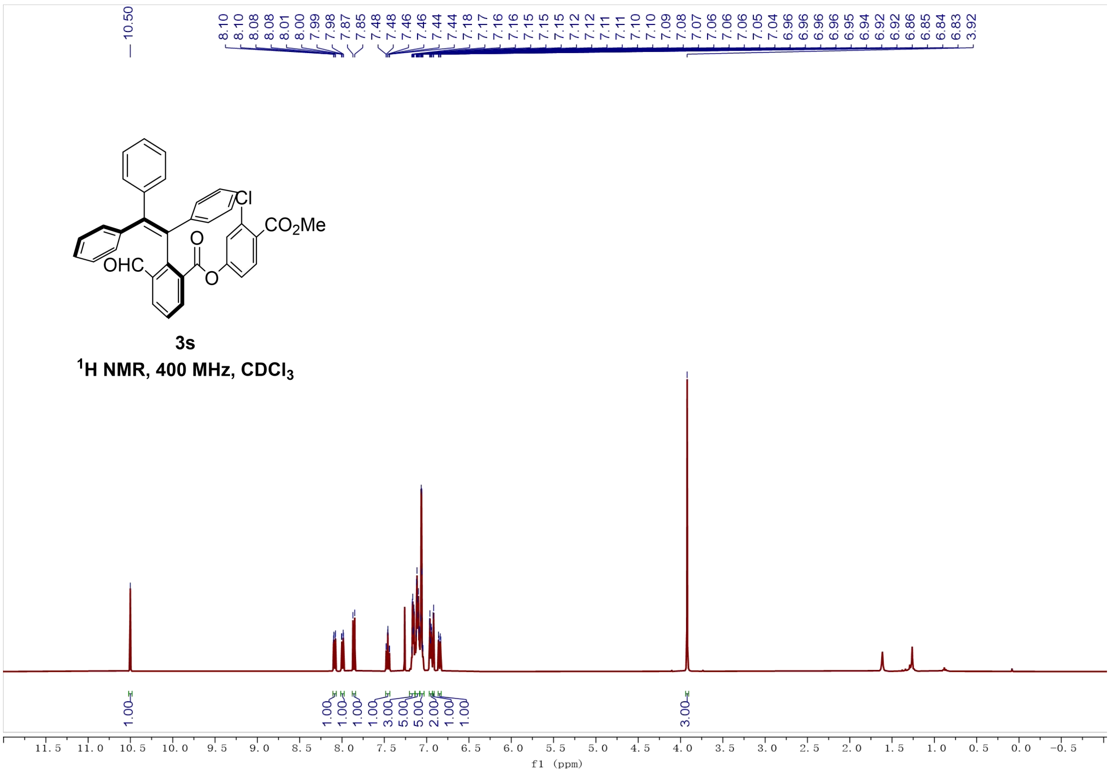
10.50
8.10 8.10 8.08 8.08 8.01 8.00 7.99 7.98 7.87 7.85 7.48 7.48 7.46 7.46 7.44 7.44 7.18 7.17 7.16 7.16 7.15 7.15 7.15 7.12 7.12 7.11 7.11 7.10 7.10 7.09 7.08 7.07 7.06 7.06 7.05 7.04 6.96 6.96 6.96 6.95 6.94 6.92 6.92 6.86 6.85 6.84 6.83 3.92
Cl
CO2Me
OHC
O
3s
1H NMR, 400 MHz, CDCl3
1.00
1.00 1.00 1.00 1.00 3.00 5.00 5.00 2.00 1.00 1.00
3.00
11.5 11.0 10.5 10.0 9.5 9.0 8.5 8.0 7.5 7.0 6.5 6.0 5.5 5.0 4.5 4.0 3.5 3.0 2.5 2.0 1.5 1.0 0.5 0.0 −0.5
f1 (ppm)

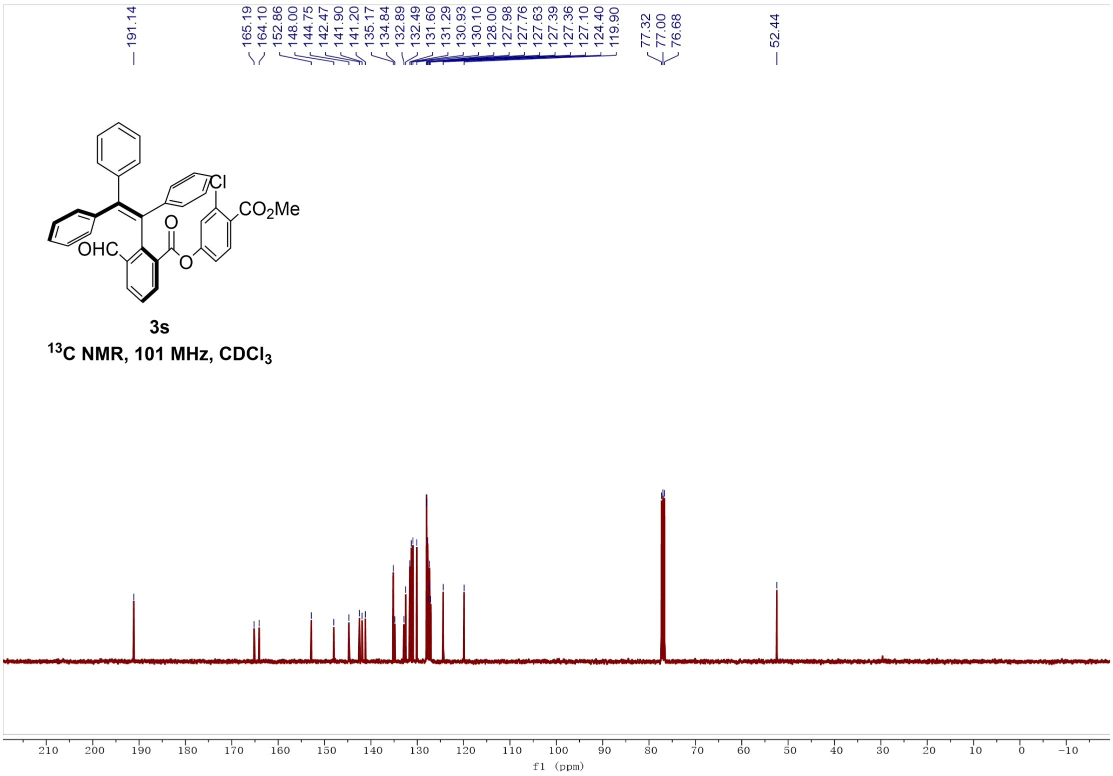
191.14
165.19
164.10
152.86
148.00
144.75
142.47
141.90
141.20
135.17
134.84
132.89
132.49
131.60
131.29
130.93
130.10
128.00
127.98
127.76
127.63
127.39
127.36
127.10
124.40
119.90
77.32
77.00
76.68
52.44
Cl
CO_2Me
O
OHC
O
3s
^{13}C NMR, 101 MHz, $CDCl_3$
f1 (ppm)

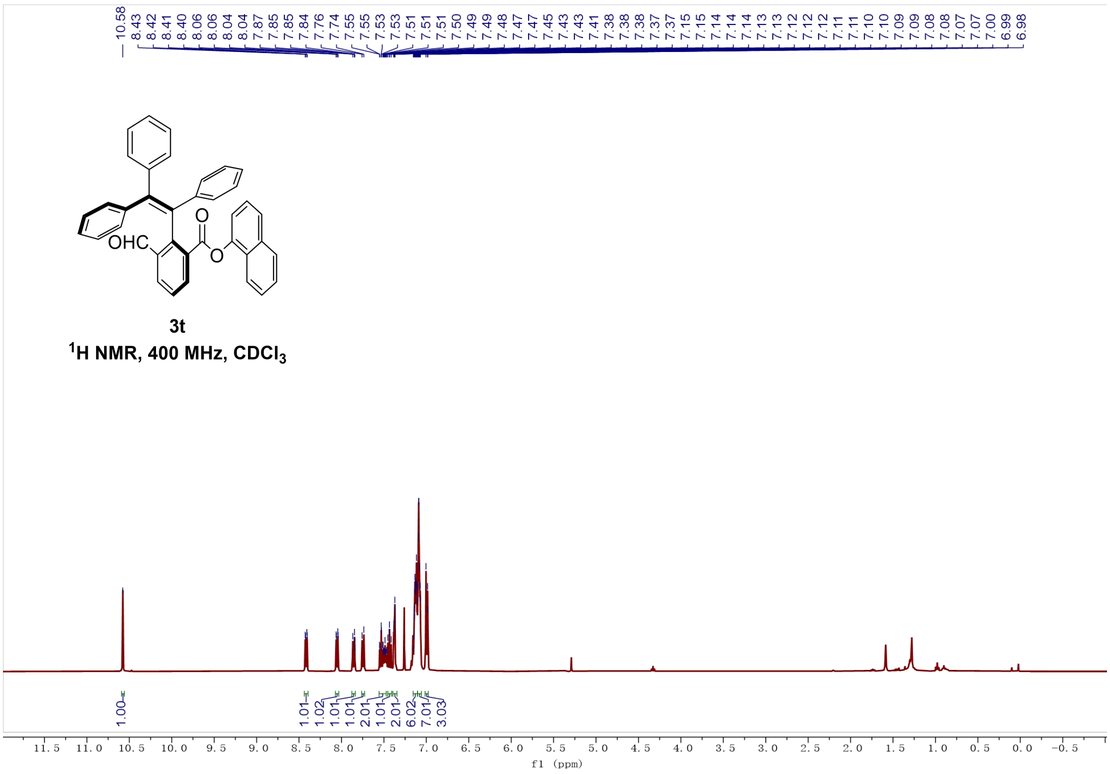
10.58
8.43
8.42
8.41
8.40
8.06
8.06
8.04
8.04
7.87
7.85
7.85
7.84
7.76
7.74
7.55
7.55
7.53
7.53
7.51
7.51
7.50
7.49
7.49
7.48
7.47
7.47
7.45
7.43
7.43
7.41
7.38
7.38
7.38
7.37
7.15
7.15
7.14
7.14
7.13
7.13
7.12
7.12
7.11
7.11
7.10
7.10
7.09
7.09
7.08
7.08
7.07
7.07
7.00
6.99
6.98
O
OHC
O
3t
^1H NMR, 400 MHz, $CDCl_3$
1.00
1.01
1.02
1.01
1.01
2.01
1.01
2.01
6.02
7.01
3.03
f1 (ppm)

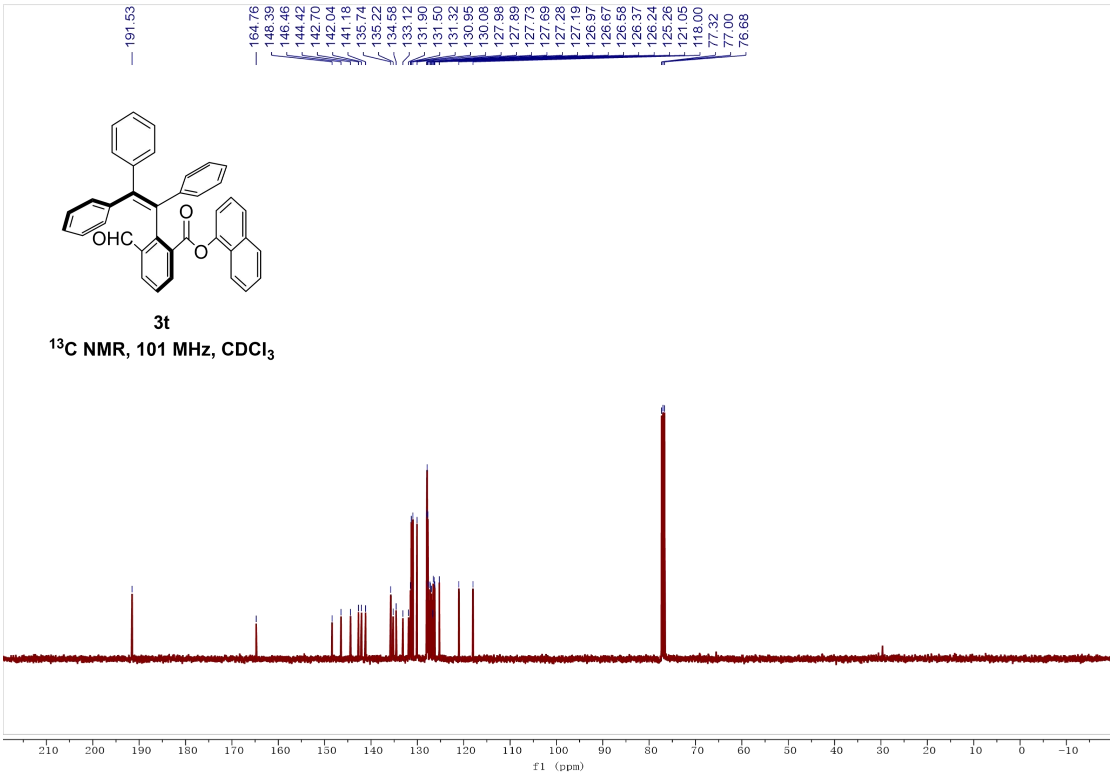
191.53
164.76
148.39
146.46
144.42
142.70
142.04
141.18
135.74
135.22
134.58
133.12
131.90
131.50
131.32
130.95
130.08
127.98
127.89
127.73
127.69
127.28
127.19
126.97
126.67
126.58
126.37
126.24
125.26
121.05
118.00
77.32
77.00
76.68
OHC
O
O
3t
13C NMR, 101 MHz, CDCl3
f1 (ppm)

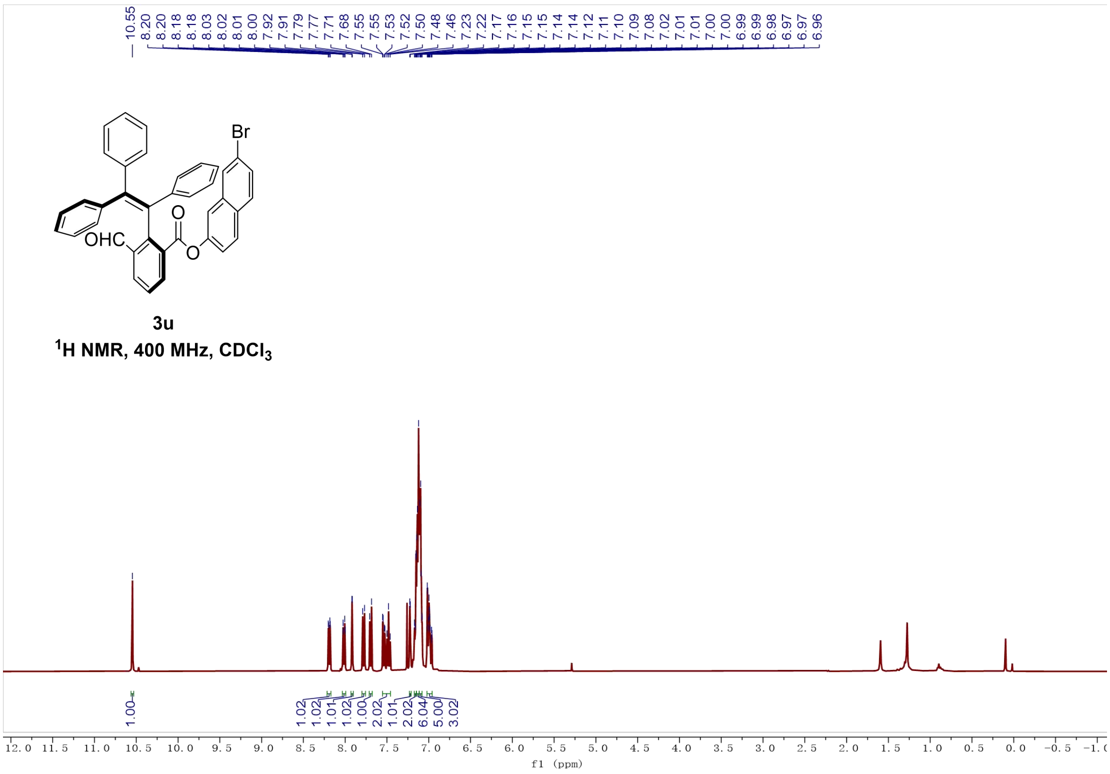
10.55
8.20
8.20
8.18
8.03
8.02
8.01
8.00
7.92
7.91
7.79
7.77
7.71
7.68
7.55
7.53
7.52
7.50
7.48
7.46
7.23
7.22
7.17
7.16
7.15
7.14
7.12
7.11
7.10
7.09
7.08
7.02
7.01
7.01
7.00
7.00
6.99
6.99
6.98
6.97
6.97
6.96
Br
OHC
O
O
3u
1H NMR, 400 MHz, CDCl3
1.00
1.02
1.02
1.01
1.02
1.00
2.02
1.01
2.02
6.04
5.00
3.02
f1 (ppm)

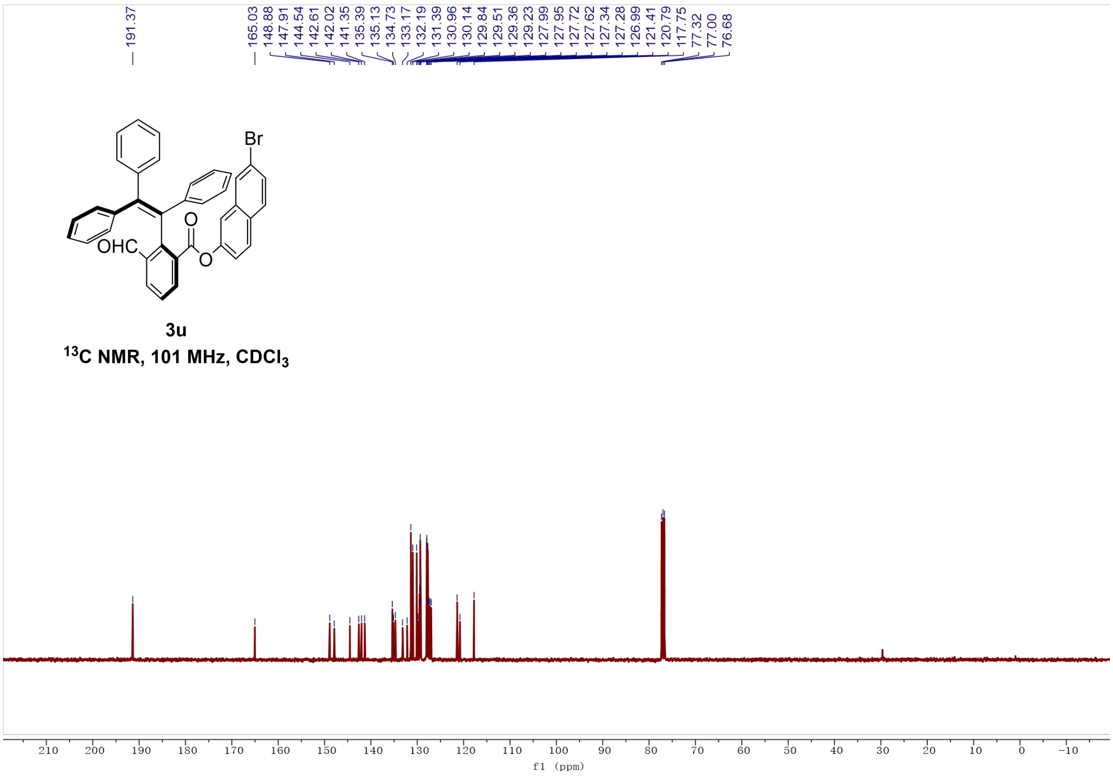
191.37
165.03 148.88 147.91 144.54 142.61 142.02 141.35 135.39 135.13 134.73 133.17 132.19 131.39 130.96 130.14 129.84 129.51 129.36 129.23 127.99 127.95 127.72 127.62 127.34 127.28 126.99 121.41 120.79 117.75 77.32 77.00 76.68
Br
O
OHC
O
3u
13C NMR, 101 MHz, CDCl3
f1 (ppm)

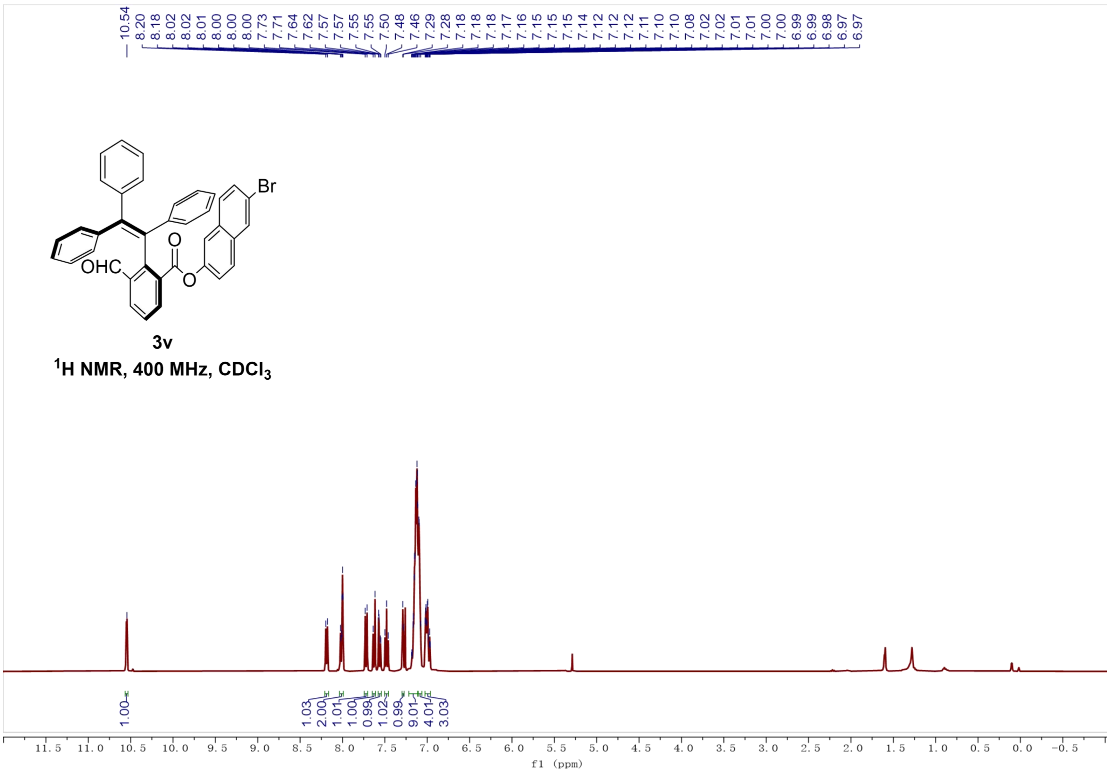
10.54
8.20 8.18 8.02 8.02 8.01 8.00 8.00 7.73 7.71 7.64 7.62 7.57 7.57 7.55 7.50 7.48 7.46 7.29 7.28 7.18 7.18 7.17 7.16 7.15 7.15 7.14 7.12 7.12 7.11 7.10 7.10 7.08 7.02 7.02 7.01 7.01 7.00 7.00 6.99 6.99 6.98 6.97 6.97
Br
O
OHC
O
3v
1H NMR, 400 MHz, CDCl3
1.00
1.03 2.00 1.01 1.00 0.99 1.02 0.99 9.01 4.01 3.03
f1 (ppm)

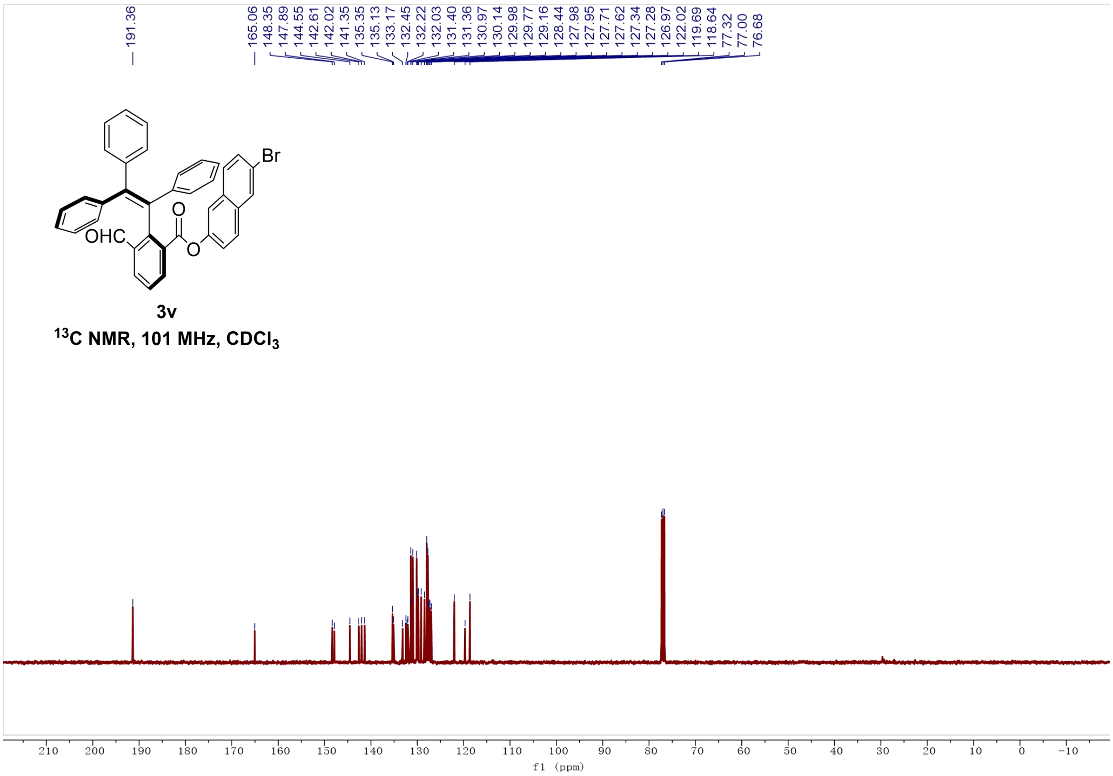

3v

^{13}C NMR, 101 MHz, $CDCl_3$

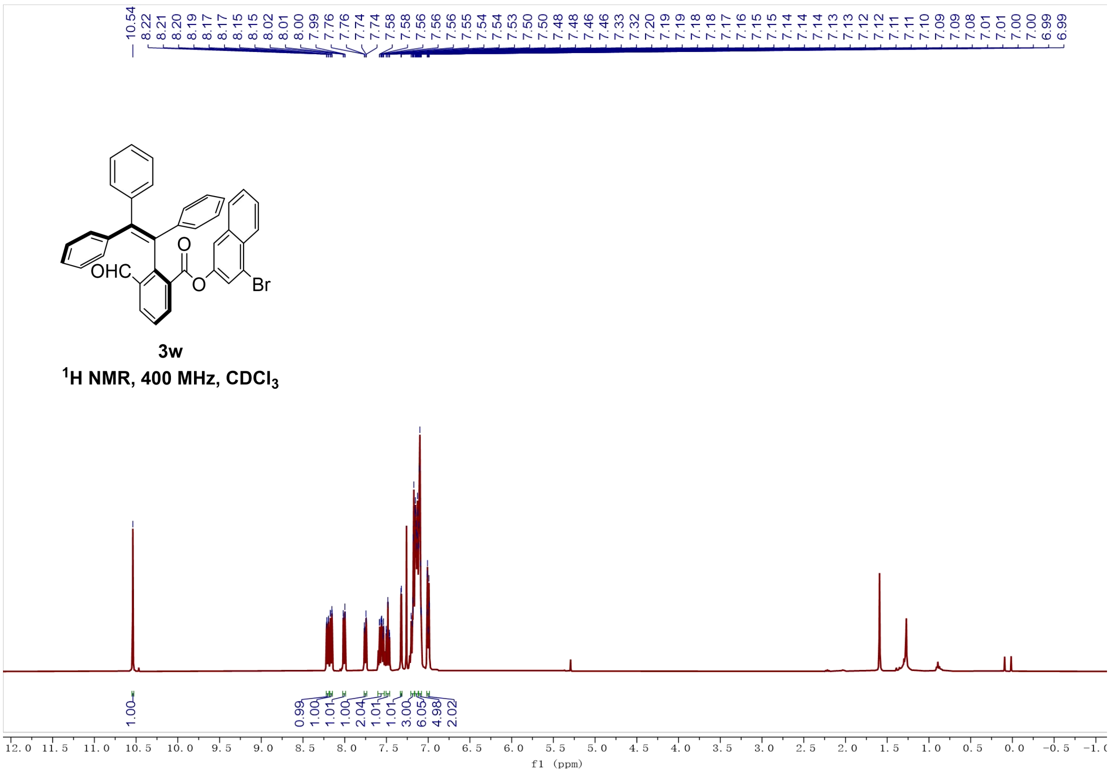

3w

^{1}H NMR, 400 MHz, $CDCl_3$

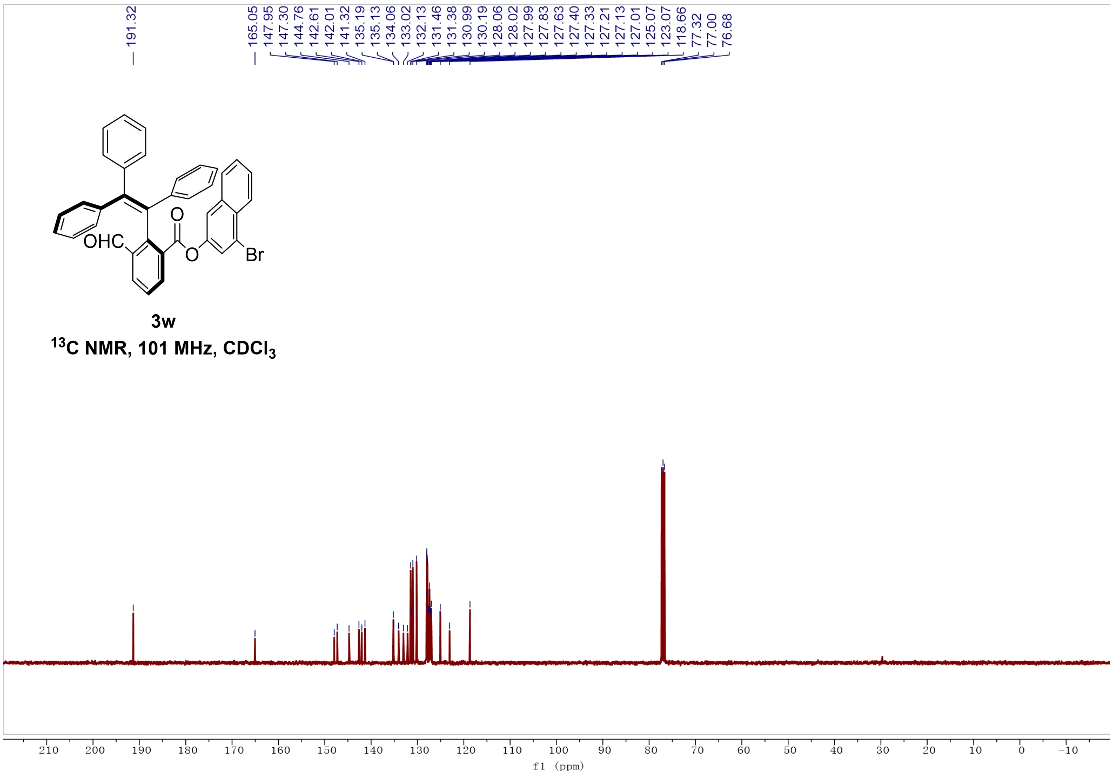
191.32
165.05
147.95
147.30
144.76
142.61
142.01
141.32
135.19
135.13
134.06
133.02
132.13
131.46
131.38
130.99
130.19
128.06
128.02
127.99
127.83
127.63
127.40
127.33
127.21
127.13
127.01
125.07
123.07
118.66
77.32
77.00
76.68
O
OHC
O
Br
3w
^{13}C NMR, 101 MHz, $CDCl_3$
210 200 190 180 170 160 150 140 130 120 110 100 90 80 70 60 50 40 30 20 10 0 −10
f1 (ppm)

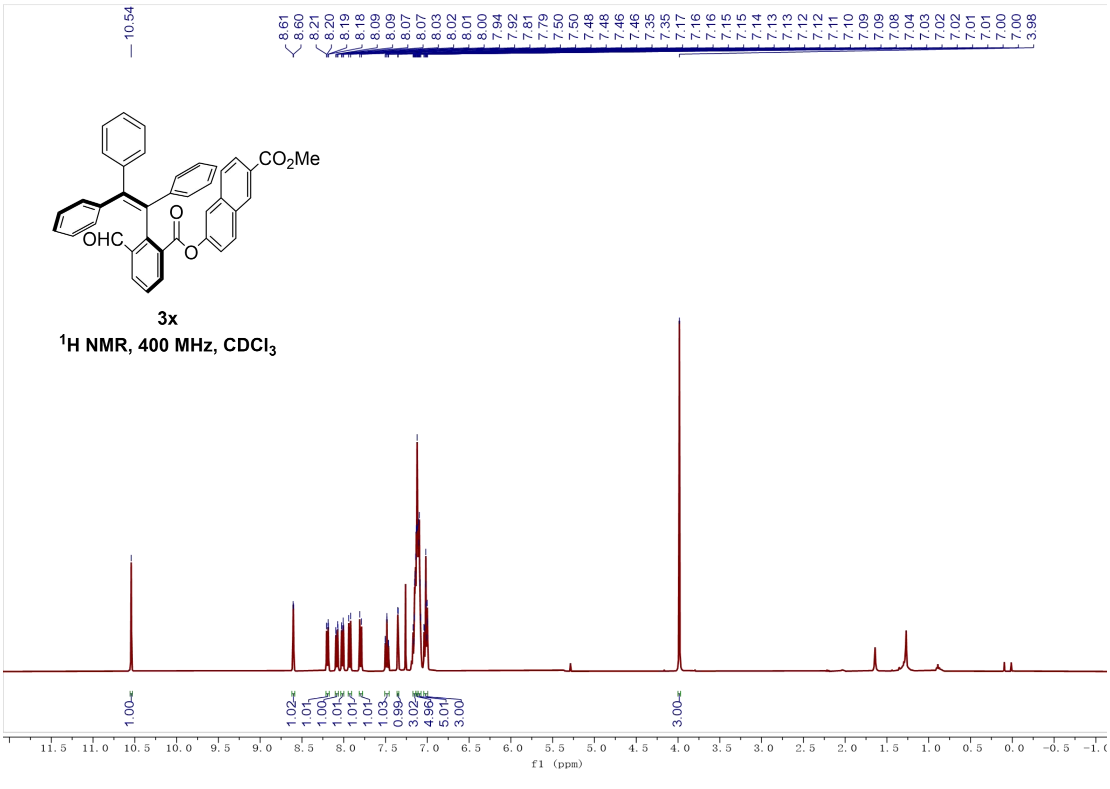
10.54
8.61
8.60
8.21
8.20
8.19
8.18
8.09
8.09
8.07
8.07
8.03
8.02
8.01
8.00
7.94
7.92
7.81
7.79
7.50
7.50
7.48
7.46
7.35
7.35
7.17
7.16
7.16
7.15
7.14
7.13
7.13
7.12
7.12
7.11
7.10
7.09
7.08
7.04
7.03
7.02
7.02
7.01
7.00
7.00
3.98
CO_2Me
O
OHC
O
3x
^{1}H NMR, 400 MHz, $CDCl_3$
1.00
1.02
1.01
1.00
1.01
1.01
1.01
1.03
0.99
3.02
4.96
5.01
3.00
3.00
11.5 11.0 10.5 10.0 9.5 9.0 8.5 8.0 7.5 7.0 6.5 6.0 5.5 5.0 4.5 4.0 3.5 3.0 2.5 2.0 1.5 1.0 0.5 0.0 −0.5 −1.0
f1 (ppm)

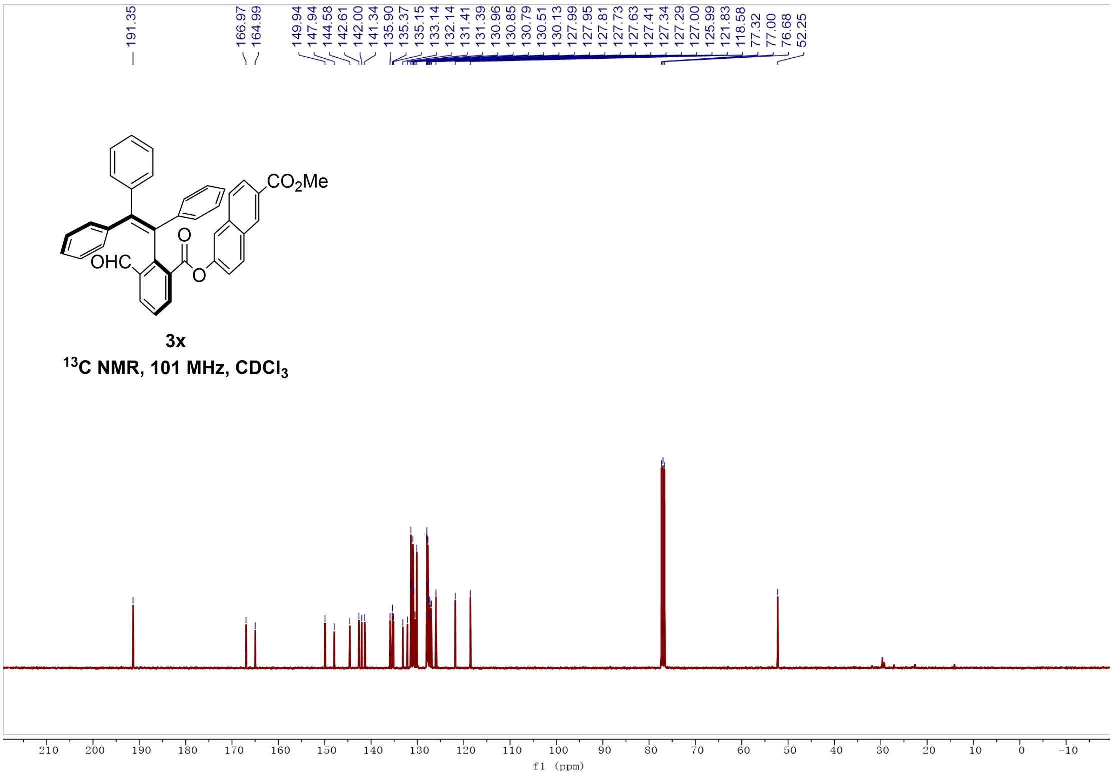

3x

^{13}C NMR, 101 MHz, CDCl$_3$

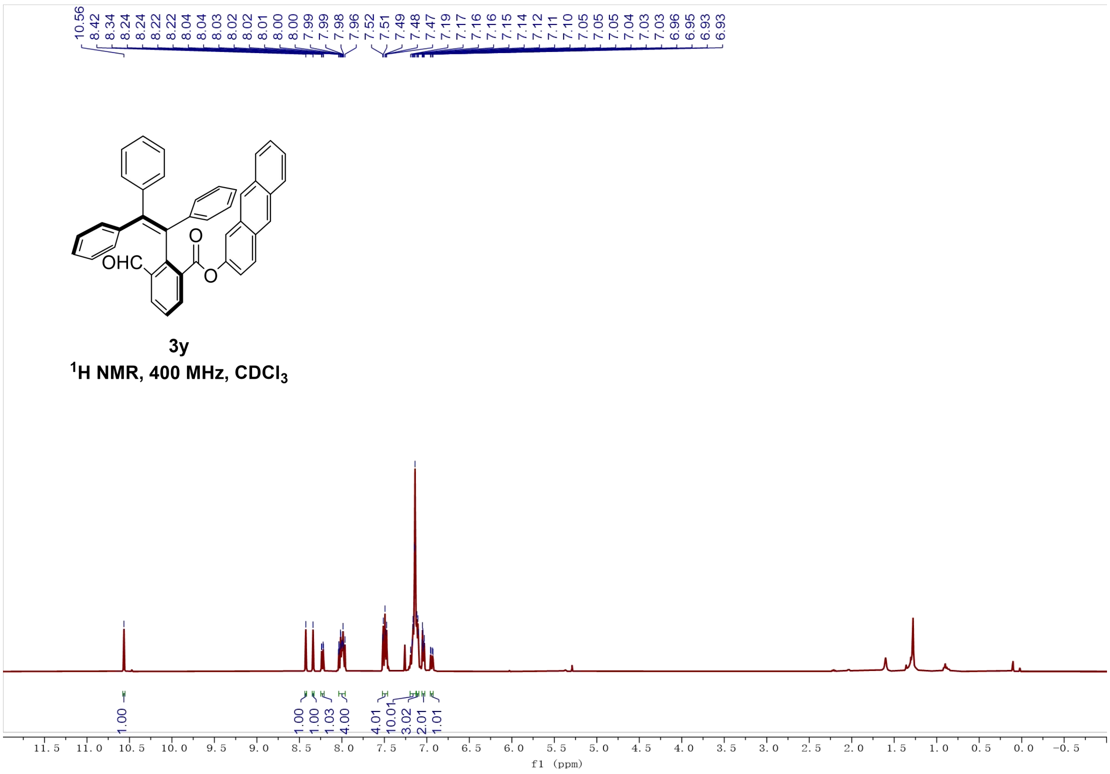

3y

^{1}H NMR, 400 MHz, CDCl$_3$

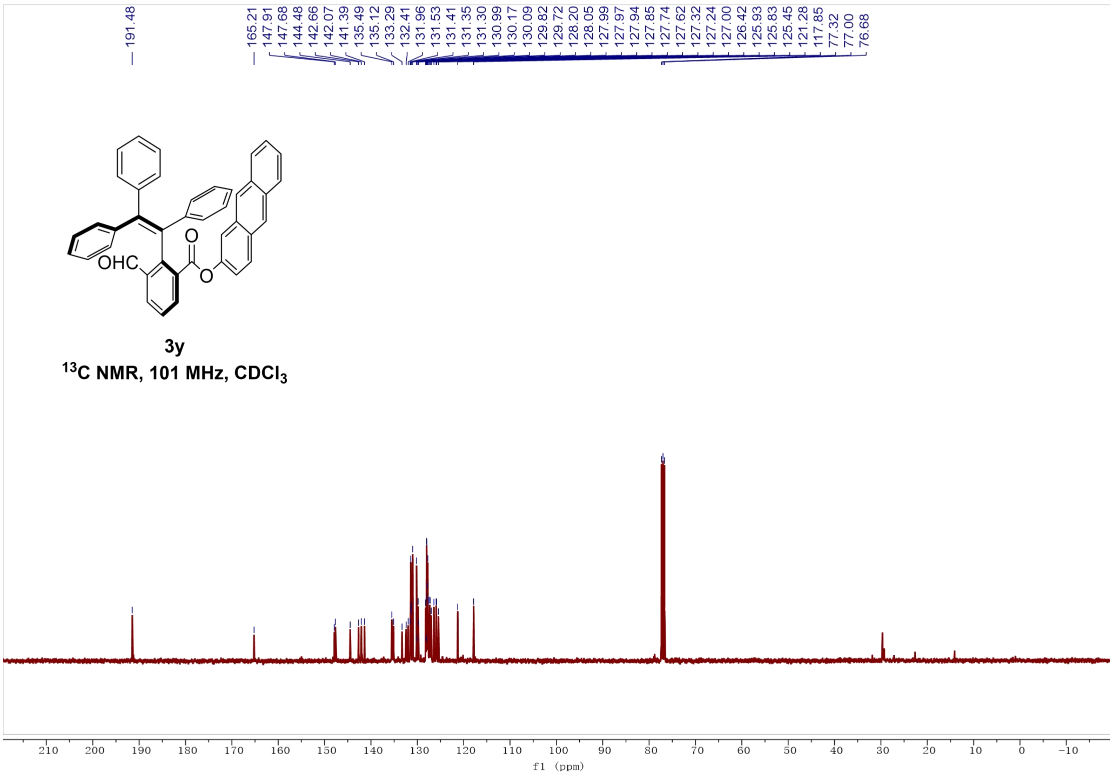
191.48
165.21 147.91 147.68 144.48 142.66 142.07 141.39 135.49 135.12 133.29 132.41 131.96 131.53 131.41 131.35 131.30 130.99 130.17 130.09 129.82 129.72 128.20 128.05 127.99 127.97 127.94 127.85 127.74 127.62 127.32 127.24 127.00 126.42 125.93 125.83 125.45 121.28 117.85 77.32 77.00 76.68
OHC
O
O
3y
^{13}C NMR, 101 MHz, CDCl$_3$
210 200 190 180 170 160 150 140 130 120 110 100 90 80 70 60 50 40 30 20 10 0 -10
f1 (ppm)

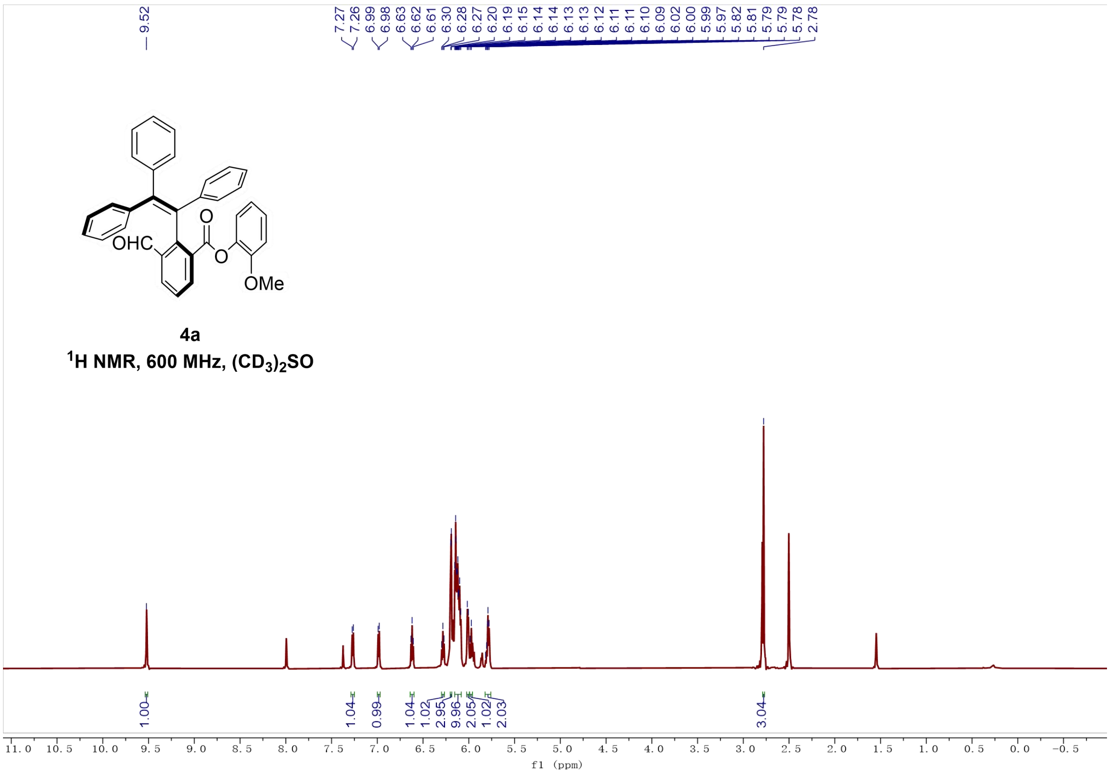
9.52
7.27 7.26 6.99 6.98 6.63 6.62 6.61 6.30 6.28 6.27 6.20 6.19 6.15 6.14 6.14 6.13 6.13 6.12 6.11 6.11 6.10 6.09 6.02 6.00 5.99 5.97 5.82 5.81 5.79 5.78 2.78
OHC
O
O
OMe
4a
^{1}H NMR, 600 MHz, (CD$_3$)$_2$SO
1.00 1.04 0.99 1.04 1.02 2.95 9.96 2.05 1.02 2.03 3.04
11.0 10.5 10.0 9.5 9.0 8.5 8.0 7.5 7.0 6.5 6.0 5.5 5.0 4.5 4.0 3.5 3.0 2.5 2.0 1.5 1.0 0.5 0.0 -0.5
f1 (ppm)

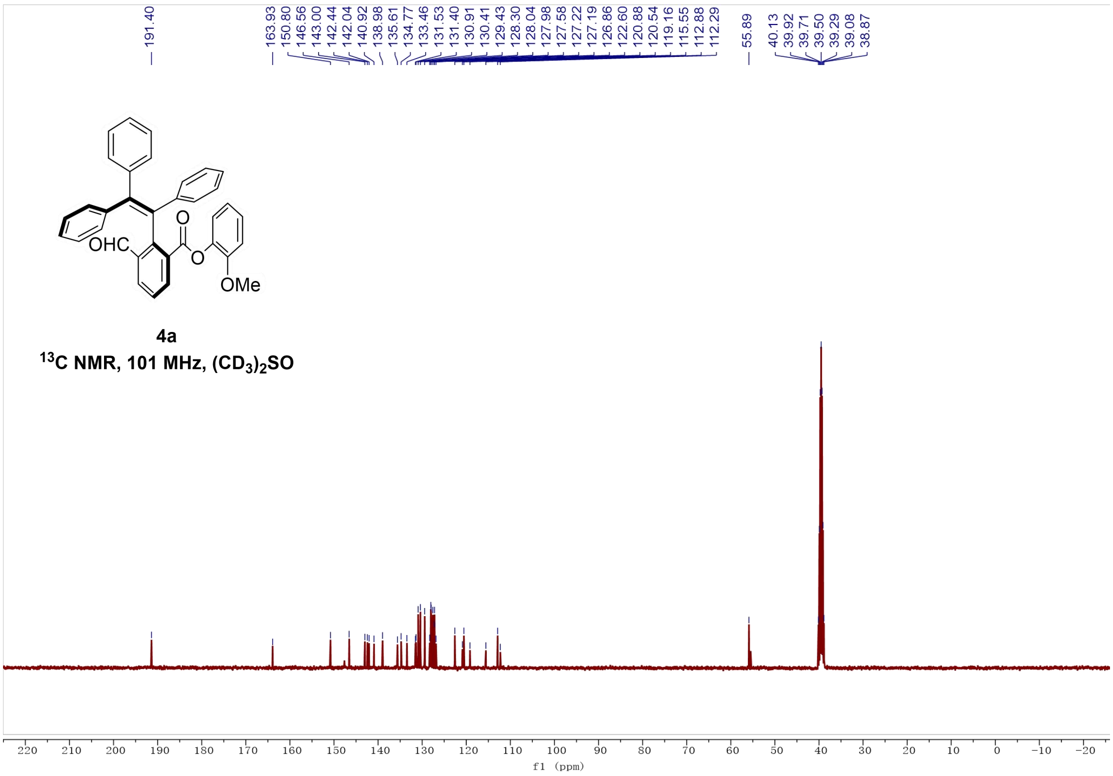

4a

^{13}C NMR, 101 MHz, $(CD_3)_2SO$

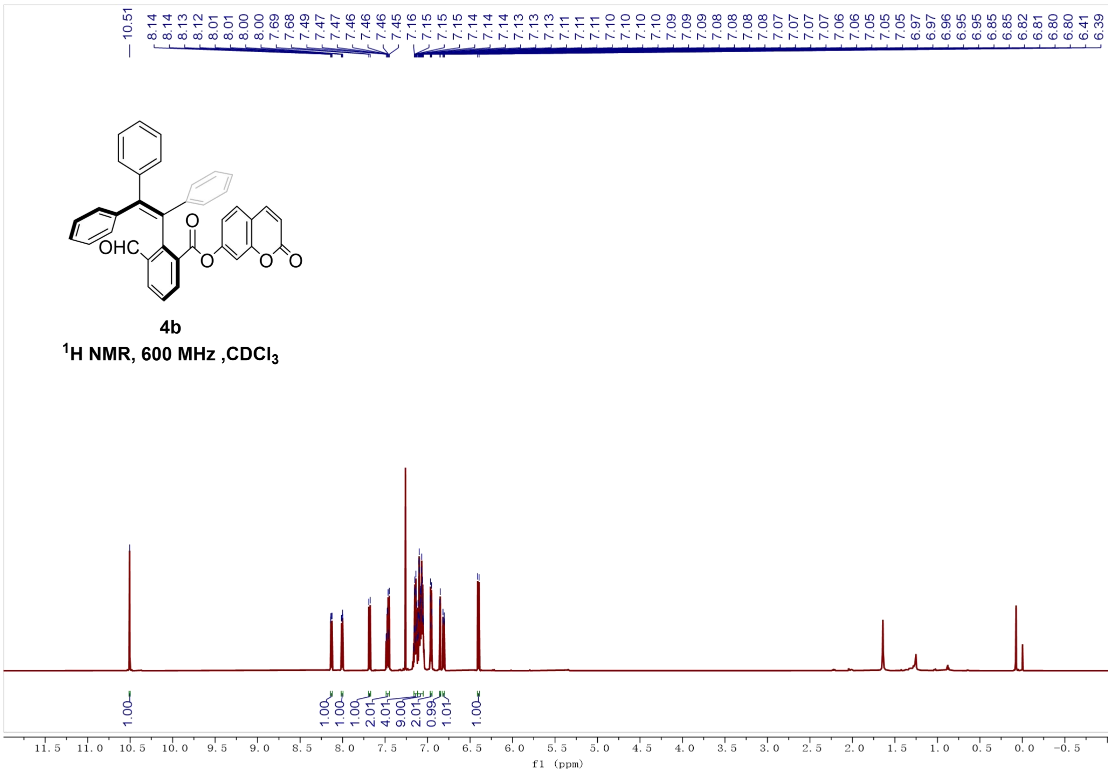

4b

^{1}H NMR, 600 MHz ,$CDCl_3$

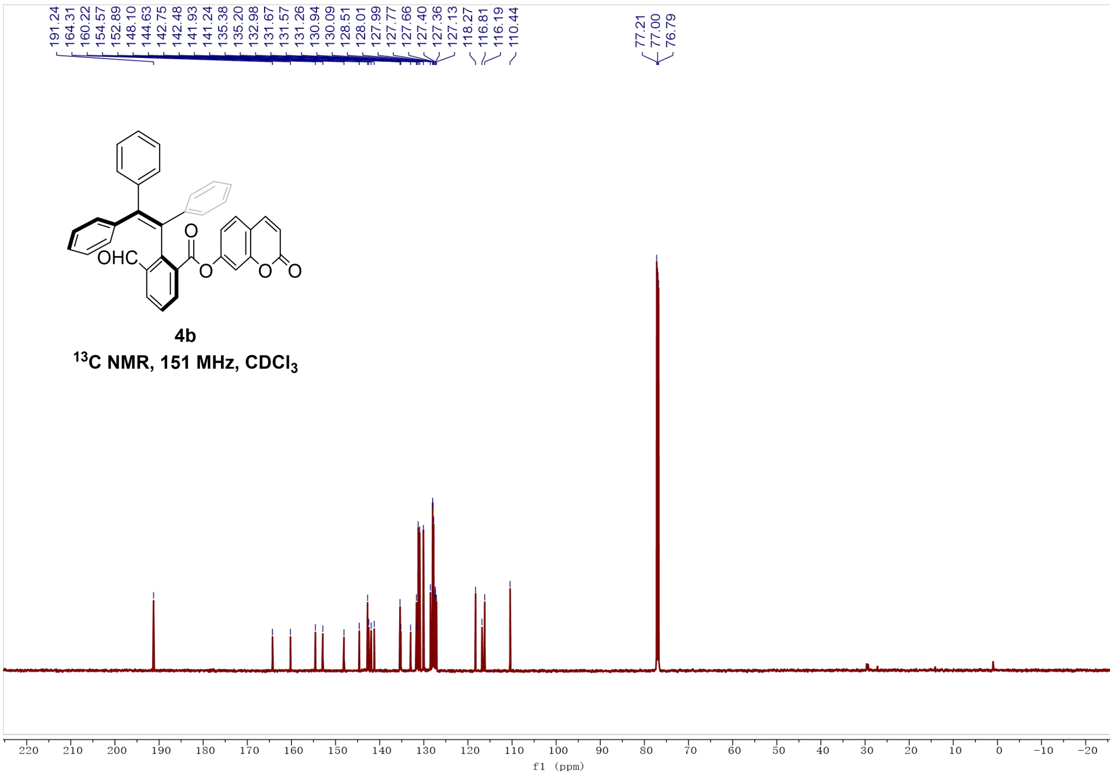
191.24 164.31 160.22 154.57 152.89 148.10 144.63 142.75 142.48 141.93 141.24 135.38 135.20 132.98 131.67 131.57 131.26 130.94 130.09 128.51 128.01 127.99 127.77 127.66 127.40 127.36 127.13 118.27 116.81 116.19 110.44
77.21 77.00 76.79
OHC
O
4b
13C NMR, 151 MHz, CDCl3
f1 (ppm)

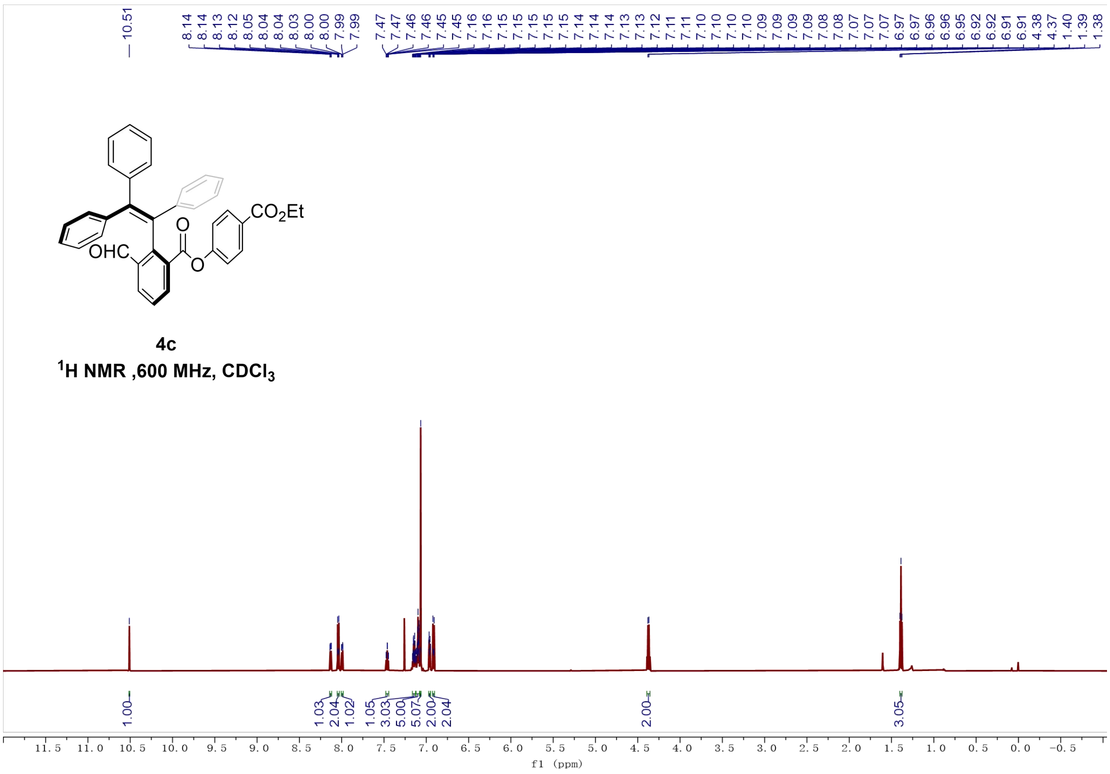
10.51
8.14 8.14 8.13 8.12 8.05 8.04 8.04 8.03 8.00 8.00 7.99 7.99 7.47 7.47 7.46 7.45 7.45 7.16 7.16 7.15 7.15 7.15 7.15 7.14 7.14 7.13 7.13 7.12 7.11 7.11 7.10 7.10 7.10 7.09 7.09 7.09 7.08 7.08 7.07 7.07 7.07 6.97 6.97 6.96 6.96 6.95 6.92 6.92 6.91 6.91 4.38 4.37 1.40 1.39 1.38
CO2Et
OHC
O
4c
1H NMR ,600 MHz, CDCl3
1.00 1.03 2.04 1.02 1.05 3.03 5.00 5.07 2.00 2.04 2.00 3.05
f1 (ppm)

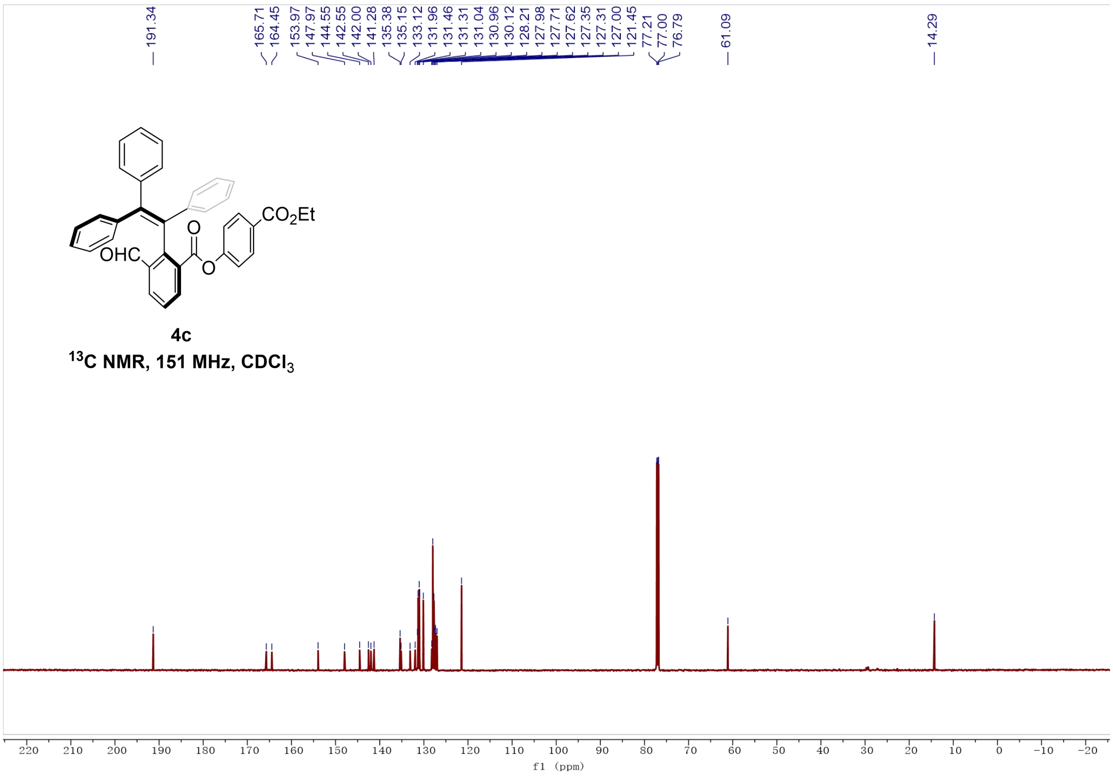

4c
^{13}C NMR, 151 MHz, $CDCl_3$

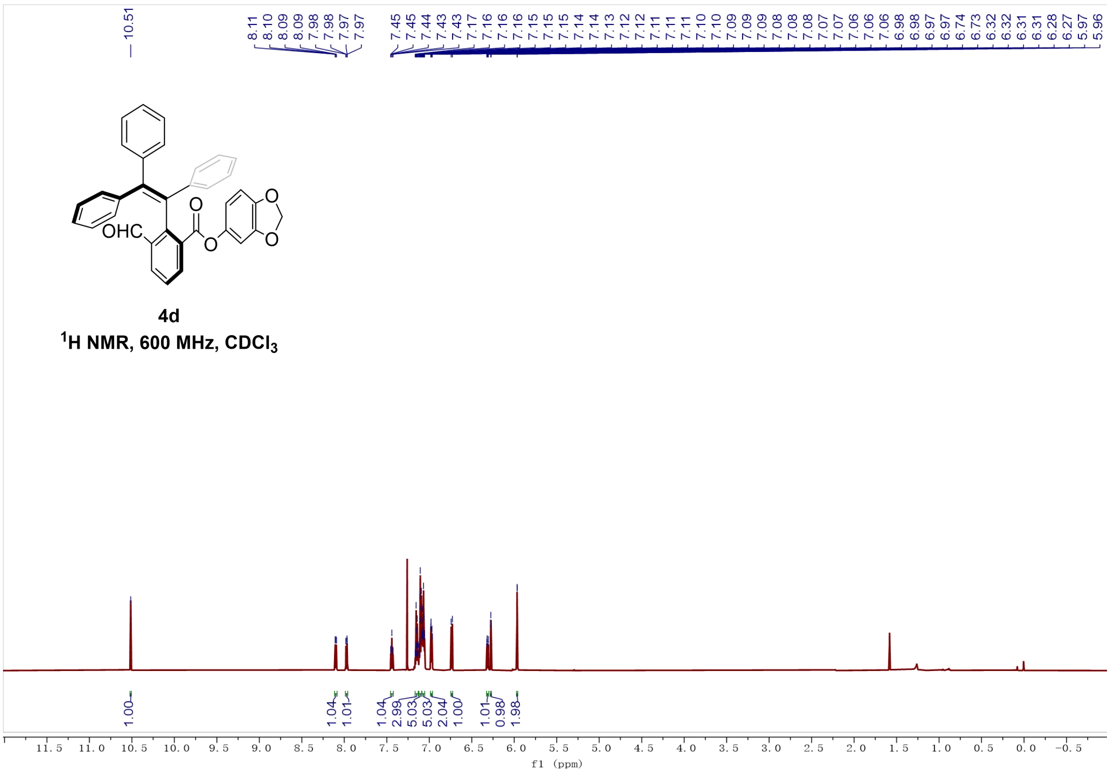

4d
^{1}H NMR, 600 MHz, $CDCl_3$

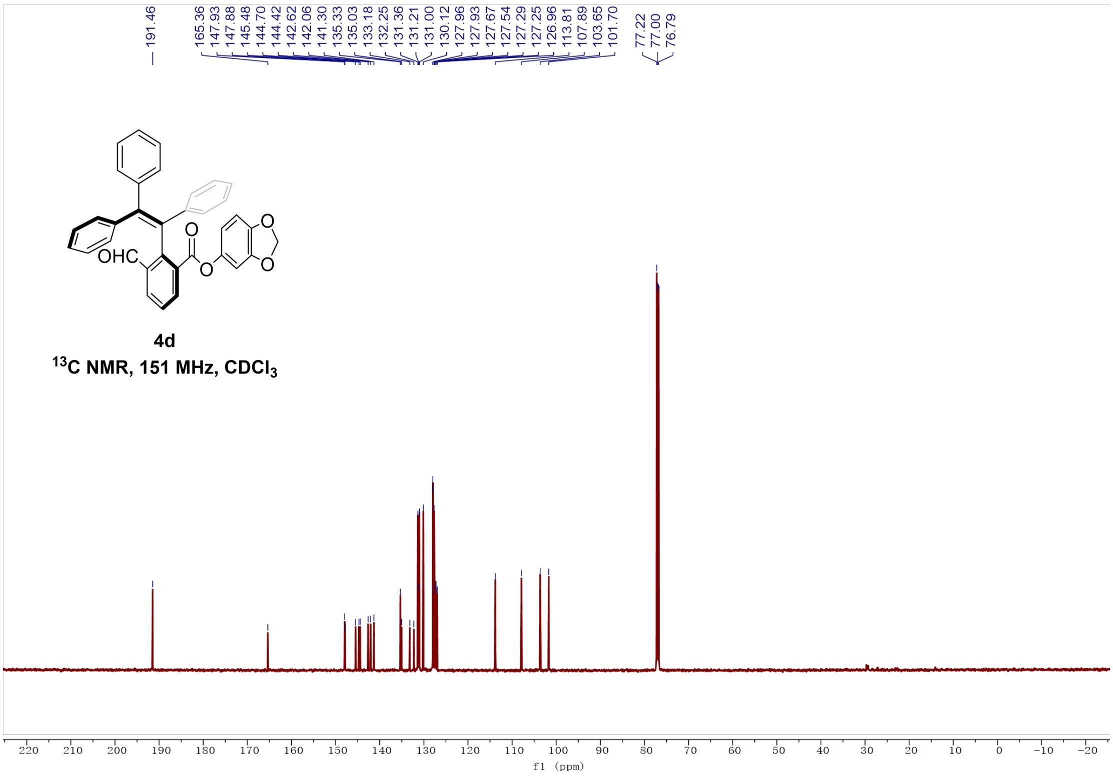

4d
^{13}C NMR, 151 MHz, $CDCl_3$

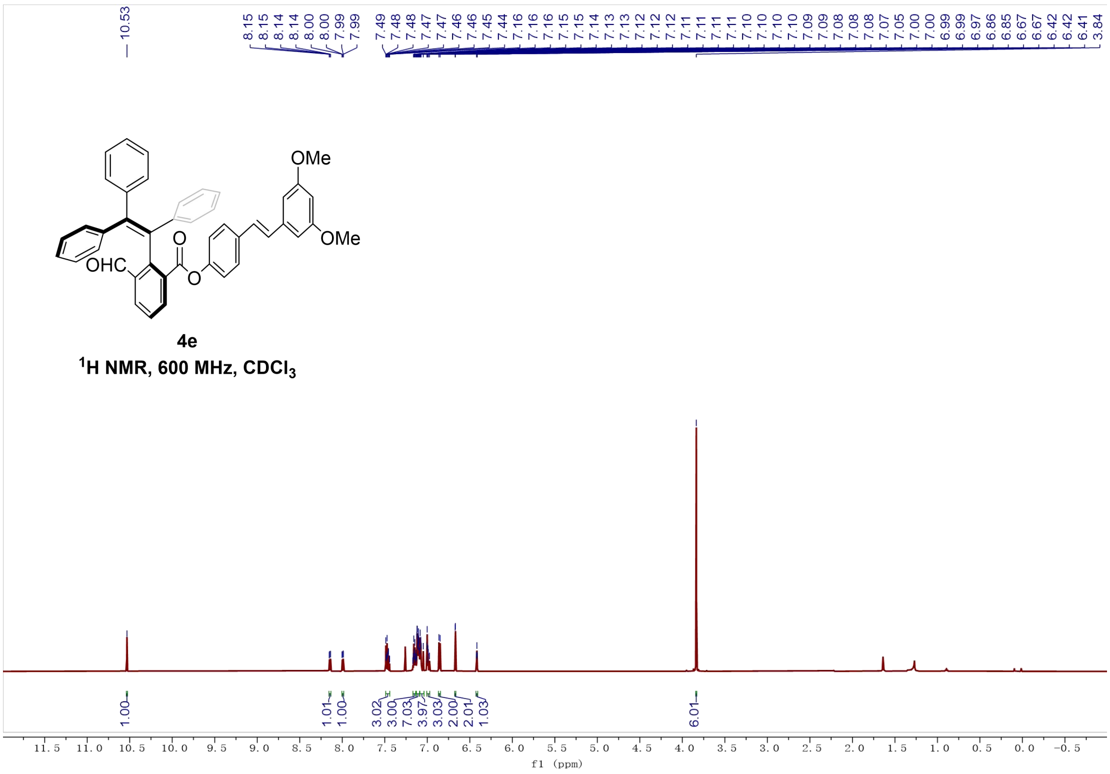

4e
^{1}H NMR, 600 MHz, $CDCl_3$

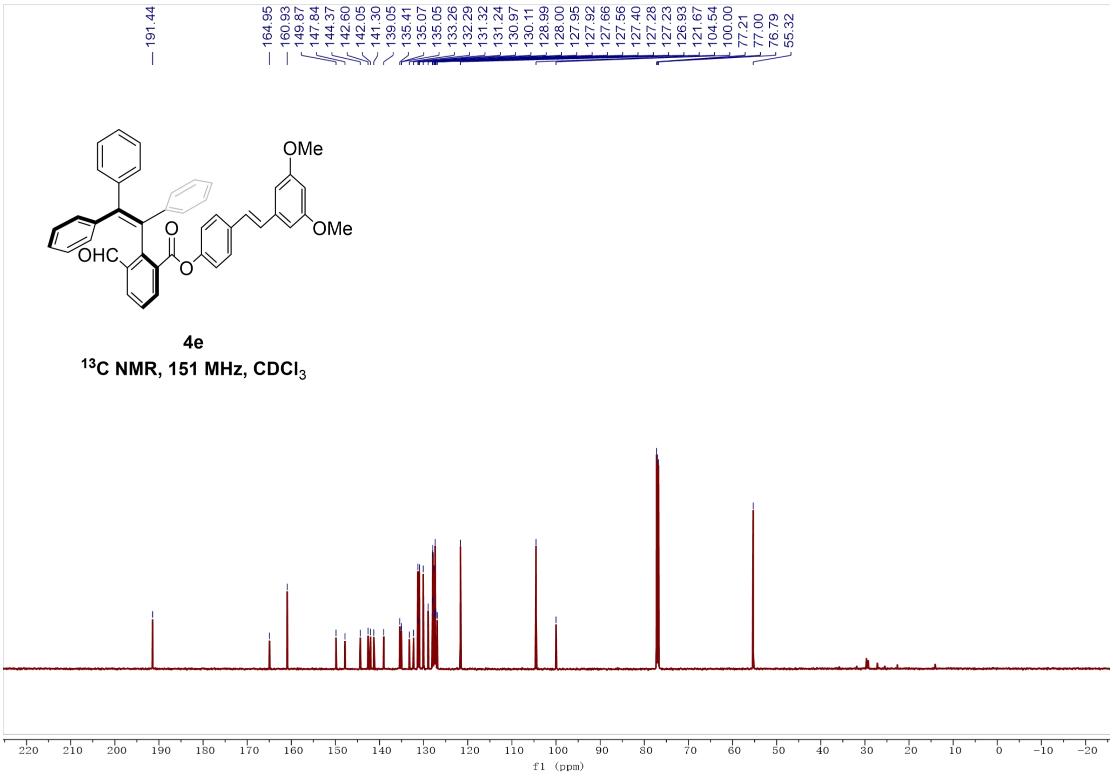

4e

^{13}C NMR, 151 MHz, $CDCl_3$

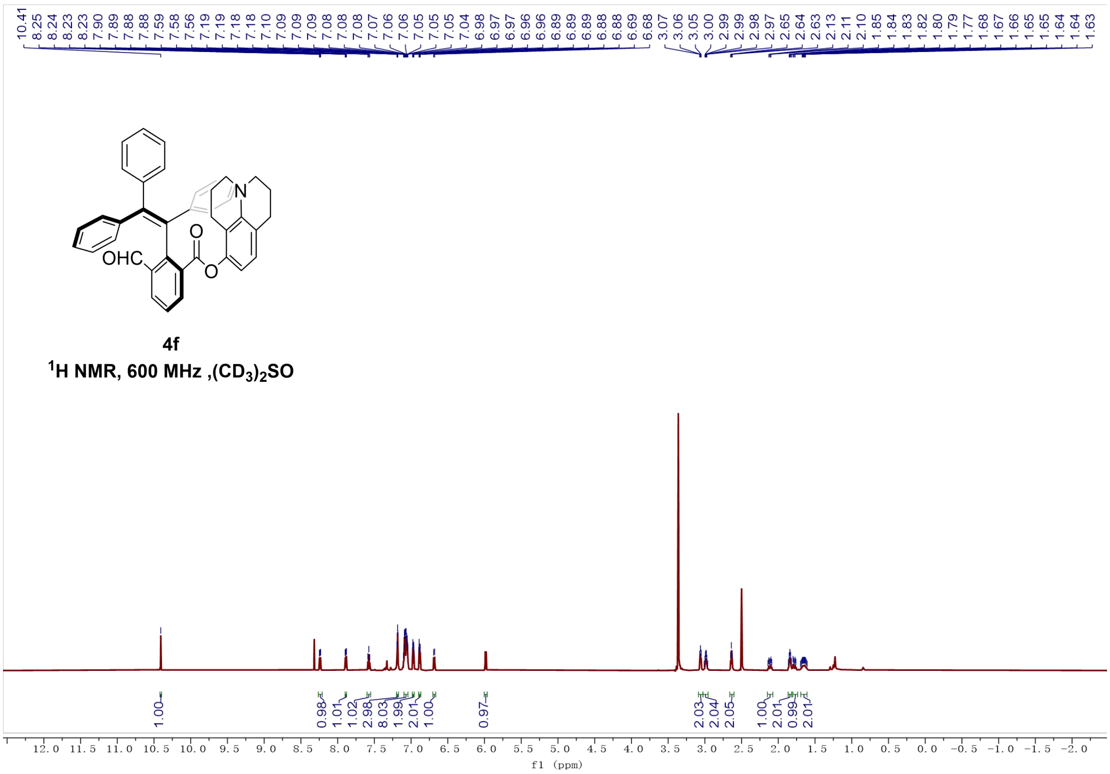

4f

^{1}H NMR, 600 MHz ,$(CD_3)_2SO$

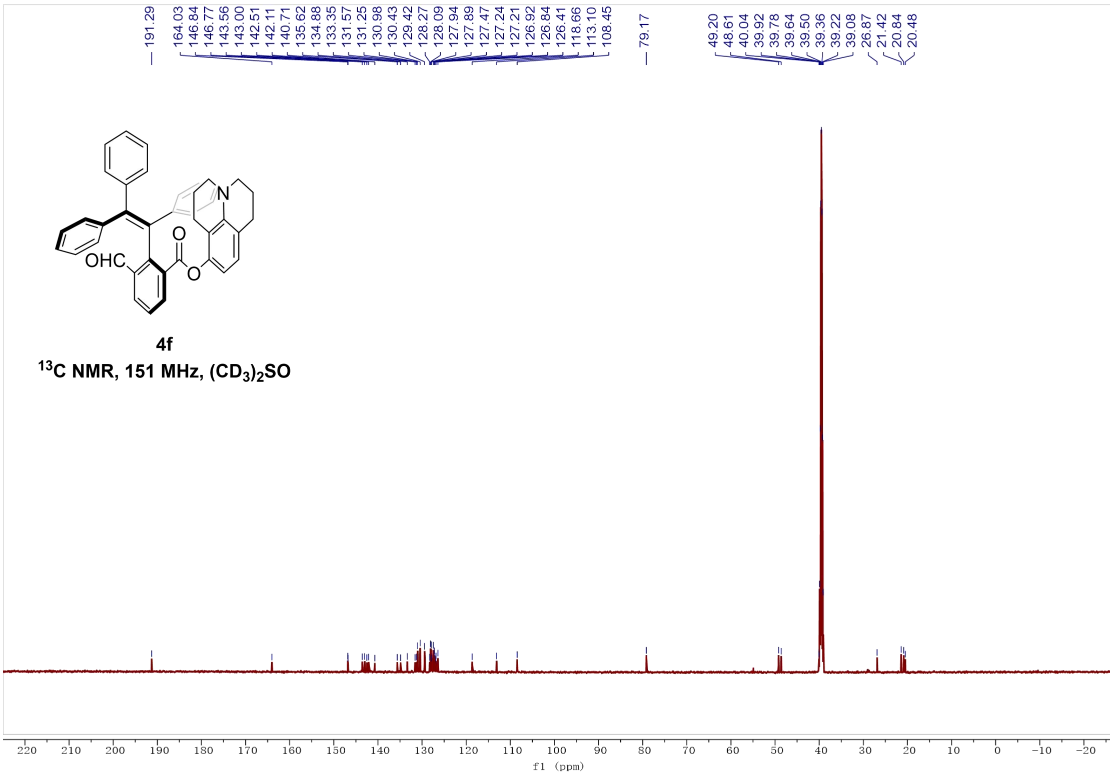
191.29
164.03 146.84 146.77 143.56 143.00 142.51 142.11 140.71 135.62 134.88 133.35 131.57 131.25 130.98 130.43 129.42 128.27 128.09 127.94 127.89 127.47 127.24 127.21 126.92 126.84 126.41 118.66 113.10 108.45
79.17
49.20 48.61 40.04 39.92 39.78 39.64 39.50 39.36 39.22 39.08 26.87 21.42 20.84 20.48
OHC
O
O
N
4f
^{13}C NMR, 151 MHz, $(CD_3)_2SO$
f1 (ppm)

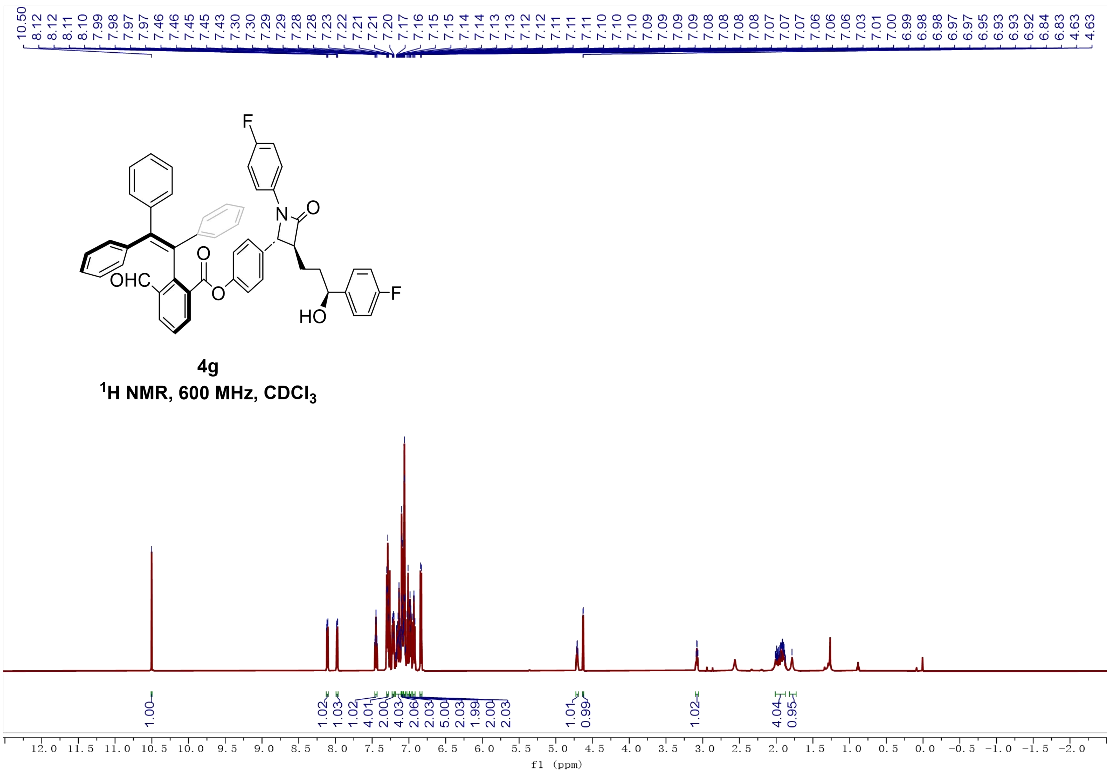
F
N
O
O
OHC
O
HO
F
4g
^{1}H NMR, 600 MHz, $CDCl_3$
f1 (ppm)

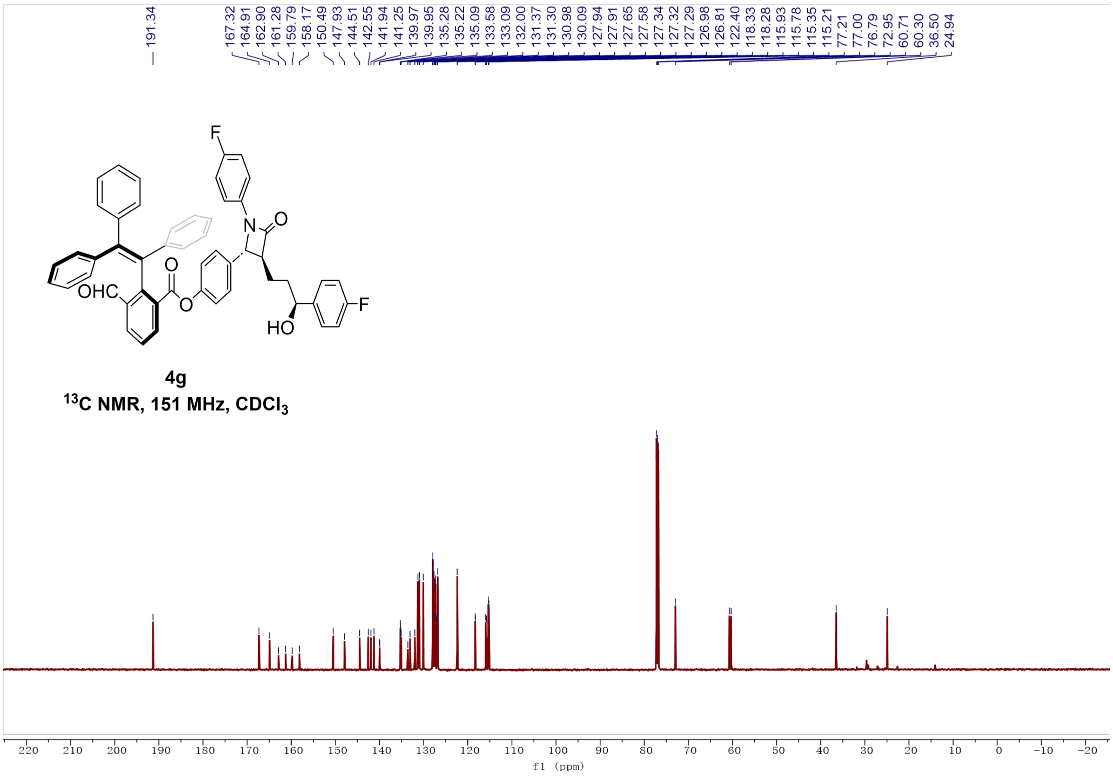
191.34
167.32
164.91
162.90
161.28
159.79
158.17
150.49
147.93
144.51
142.55
141.94
141.25
139.97
139.95
135.28
135.22
135.09
133.58
133.09
132.00
131.37
131.30
130.98
130.09
127.94
127.91
127.65
127.58
127.34
127.32
127.29
126.98
126.81
122.40
118.33
118.28
115.93
115.78
115.35
115.21
77.21
77.00
76.79
72.95
60.71
60.30
36.50
24.94
F
N
O
OHC
O
O
HO
F
4g
^{13}C NMR, 151 MHz, CDCl$_3$
f1 (ppm)

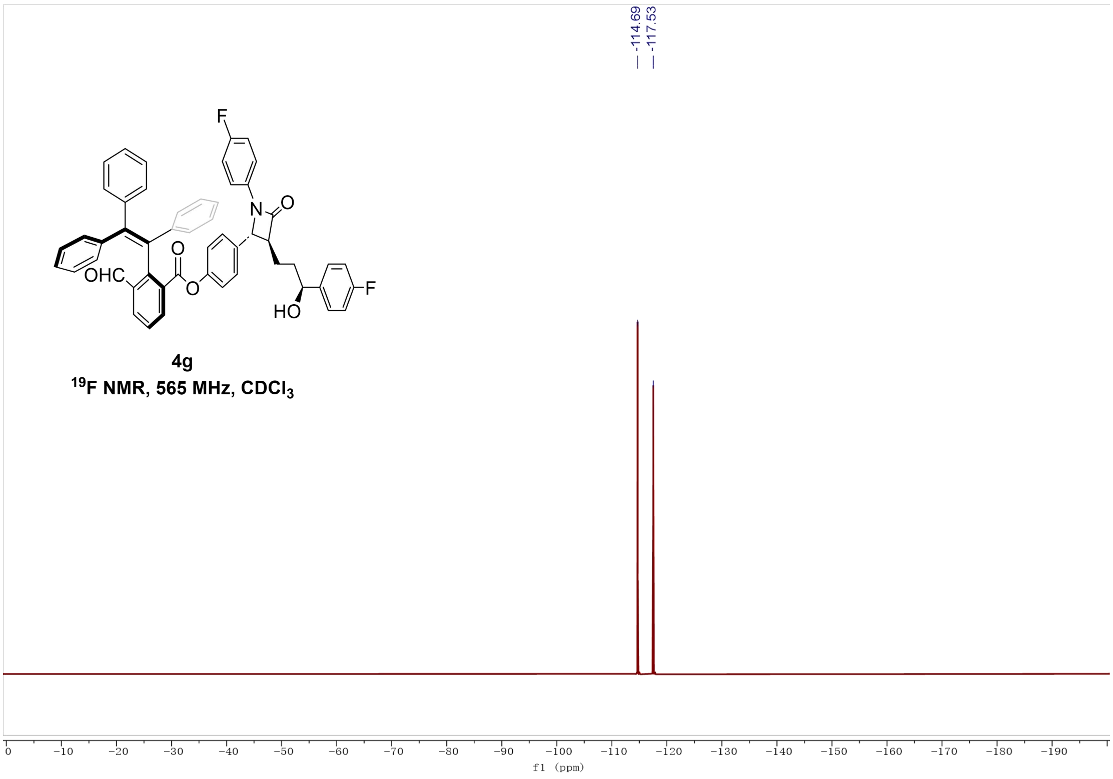
-114.69
-117.53
F
N
O
OHC
O
O
HO
F
4g
^{19}F NMR, 565 MHz, CDCl$_3$
f1 (ppm)

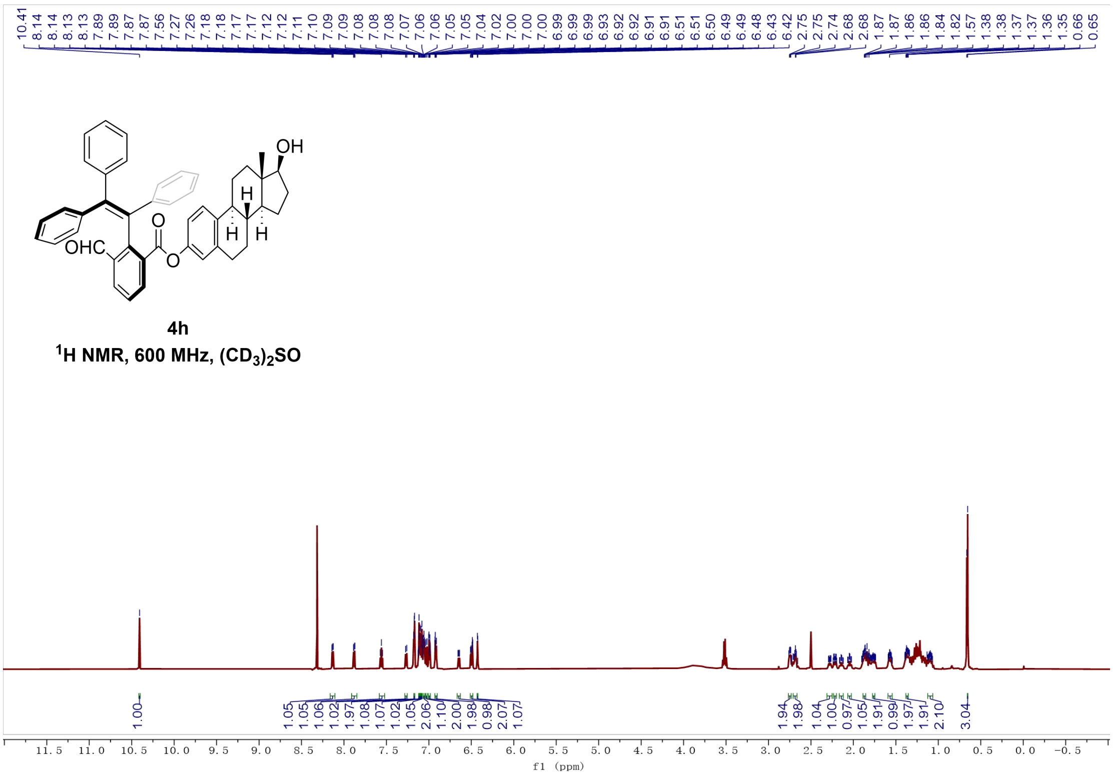
10.41 8.14 8.14 8.13 8.13 7.89 7.89 7.87 7.87 7.56 7.27 7.26 7.18 7.18 7.17 7.17 7.12 7.12 7.11 7.10 7.09 7.09 7.08 7.08 7.08 7.07 7.06 7.06 7.05 7.05 7.04 7.02 7.00 7.00 7.00 6.99 6.99 6.99 6.93 6.92 6.92 6.91 6.91 6.51 6.51 6.50 6.49 6.49 6.48 6.43 6.42 2.75 2.75 2.74 2.68 2.68 1.87 1.87 1.86 1.86 1.84 1.82 1.57 1.38 1.38 1.37 1.37 1.36 1.35 0.66 0.65
OH
H
H
H
O
OHC
O
4h
^{1}H NMR, 600 MHz, $(CD_3)_2SO$
1.00 1.05 1.05 1.06 1.02 1.97 1.08 1.07 1.02 1.05 2.06 1.10 2.00 1.98 0.98 2.07 1.07 1.94 1.98 1.04 1.00 0.97 1.05 1.91 0.99 1.97 1.91 2.10 3.04
11.5 11.0 10.5 10.0 9.5 9.0 8.5 8.0 7.5 7.0 6.5 6.0 5.5 5.0 4.5 4.0 3.5 3.0 2.5 2.0 1.5 1.0 0.5 0.0 -0.5
f1 (ppm)

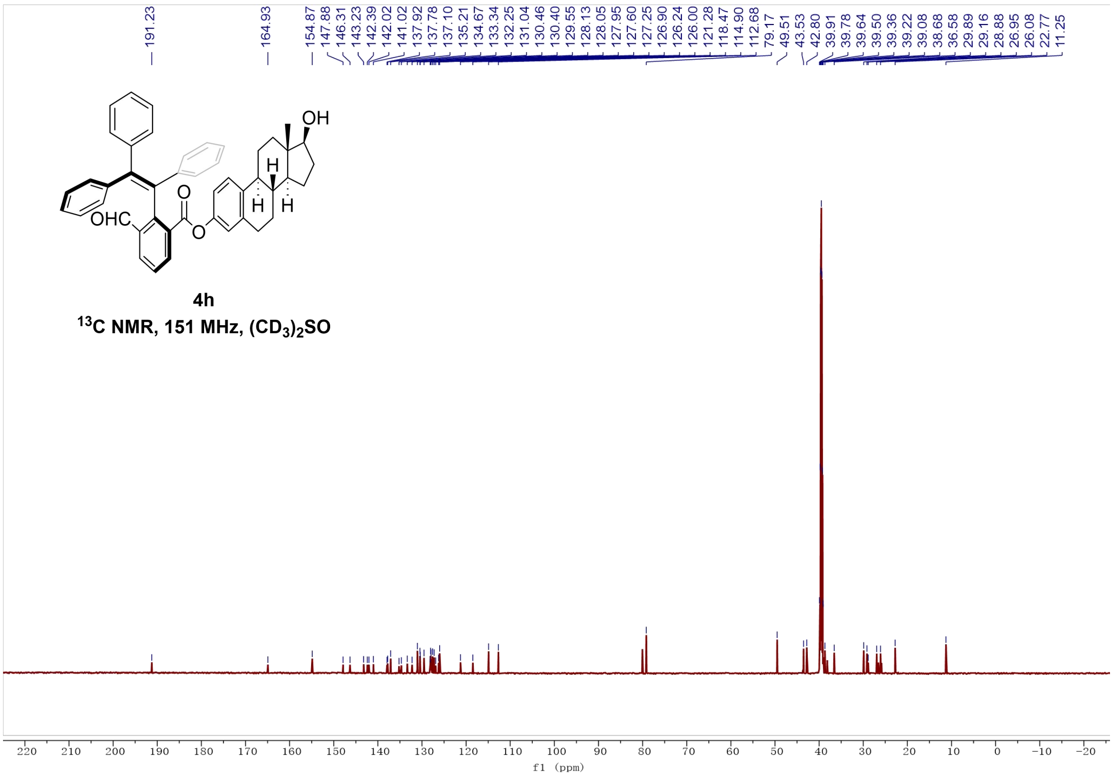
191.23 164.93 154.87 147.88 146.31 143.23 142.39 142.02 141.02 137.92 137.78 137.10 135.21 134.67 133.34 132.25 131.04 130.46 130.40 129.55 128.13 128.05 127.95 127.60 127.25 126.90 126.24 126.00 121.28 118.47 114.90 112.68 79.17 49.51 43.53 42.80 39.91 39.78 39.64 39.50 39.36 39.22 39.08 38.68 36.58 29.89 29.16 28.88 26.95 26.08 22.77 11.25
OH
H
H
H
O
OHC
O
4h
^{13}C NMR, 151 MHz, $(CD_3)_2SO$
220 210 200 190 180 170 160 150 140 130 120 110 100 90 80 70 60 50 40 30 20 10 0 -10 -20
f1 (ppm)

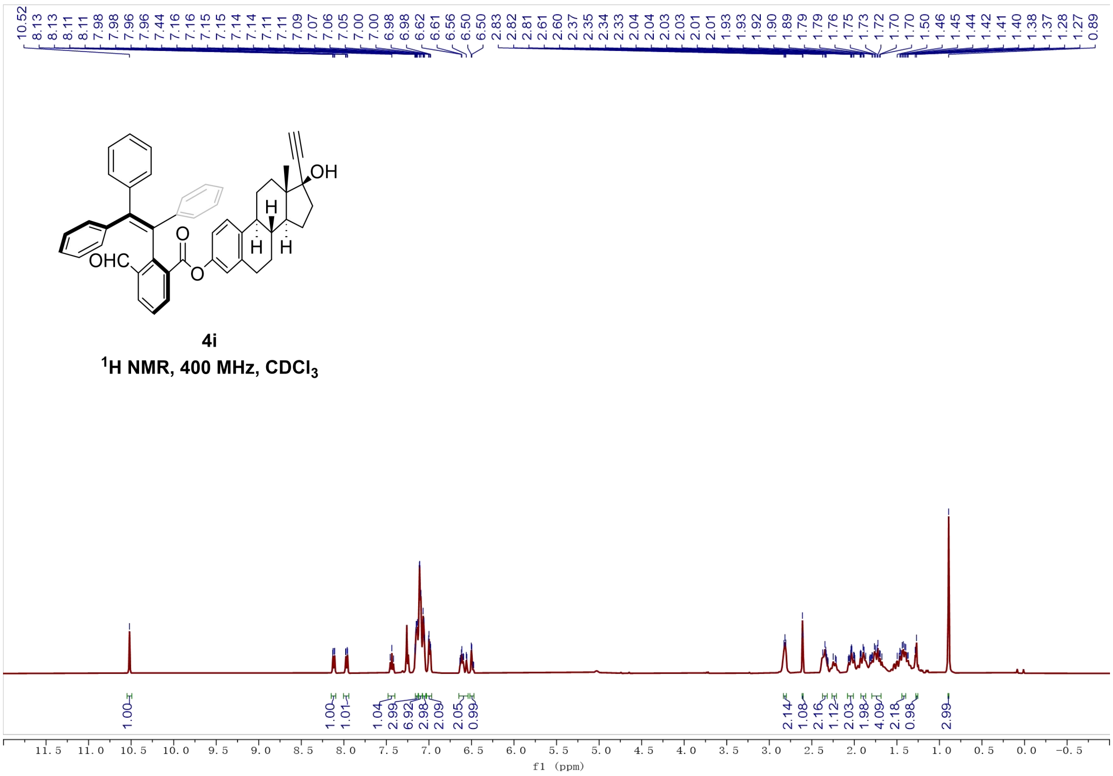

4i
^{1}H NMR, 400 MHz, $CDCl_3$

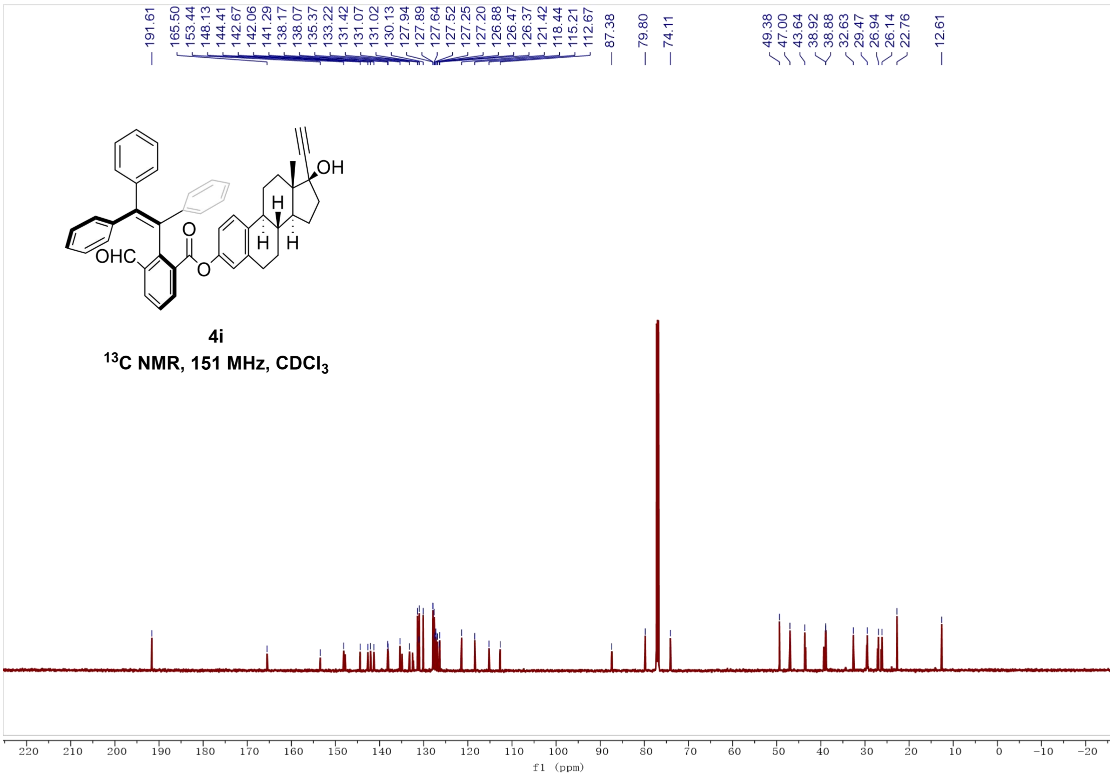

4i
^{13}C NMR, 151 MHz, $CDCl_3$

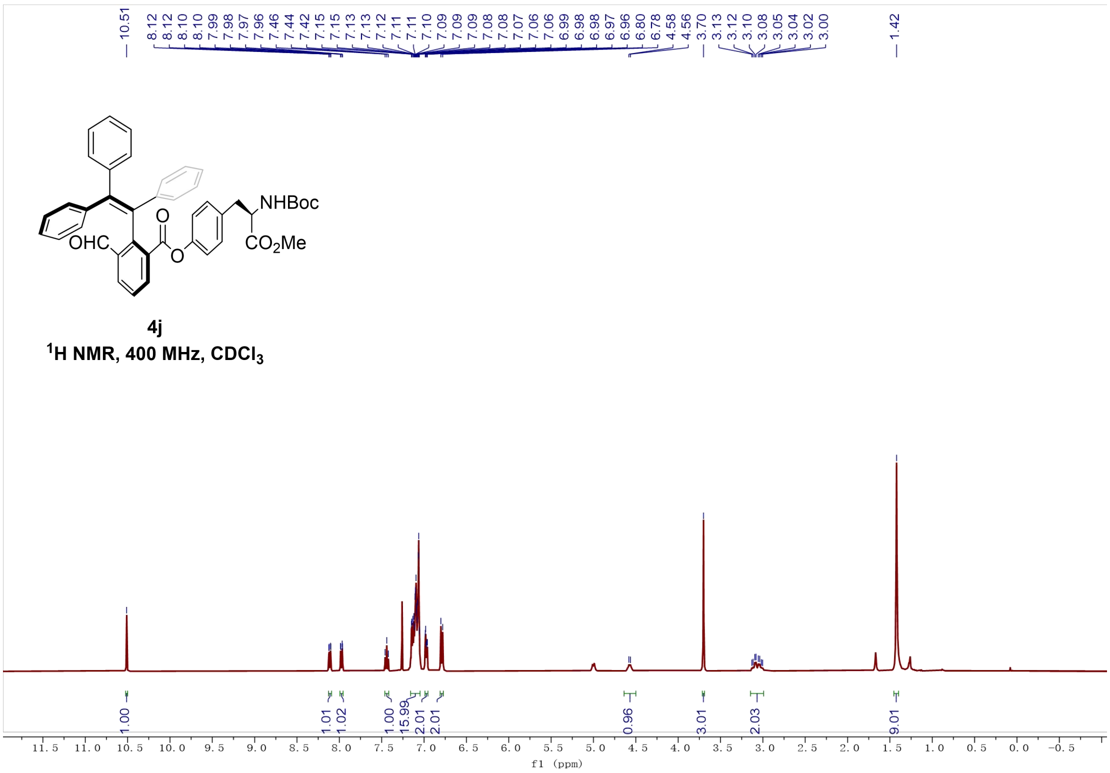
NHBoc
OHC
O
O
CO2Me
4j
1H NMR, 400 MHz, CDCl3

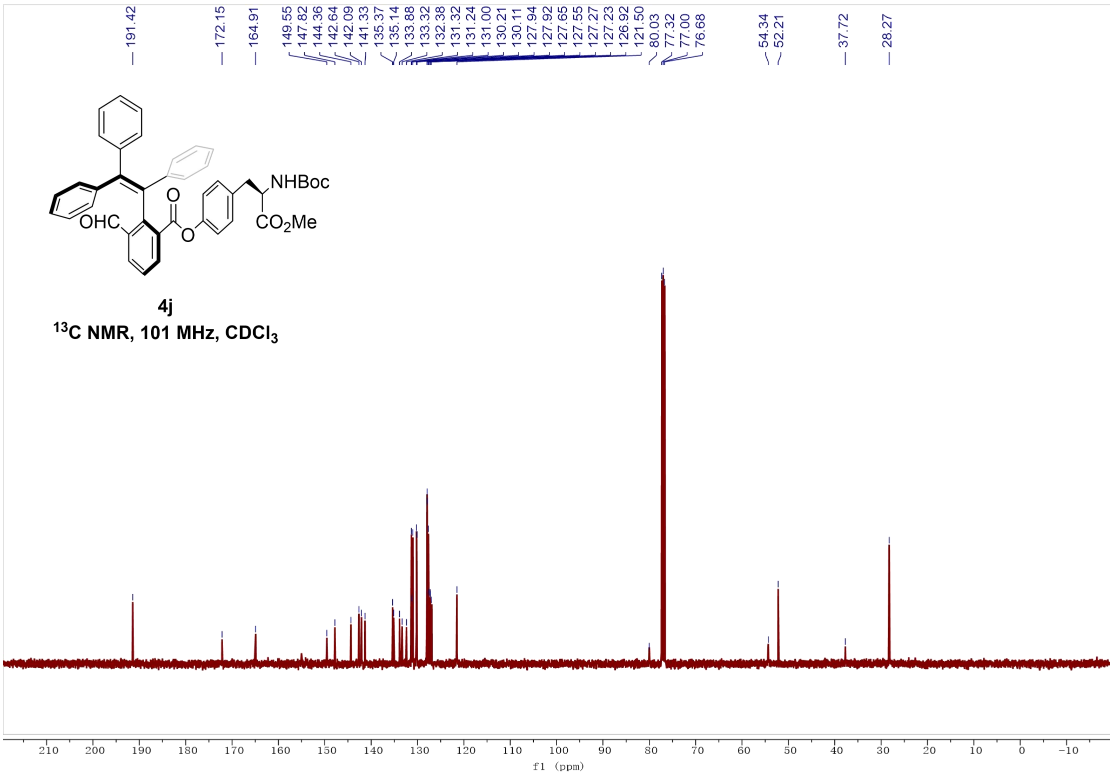
NHBoc
OHC
O
O
CO2Me
4j
13C NMR, 101 MHz, CDCl3

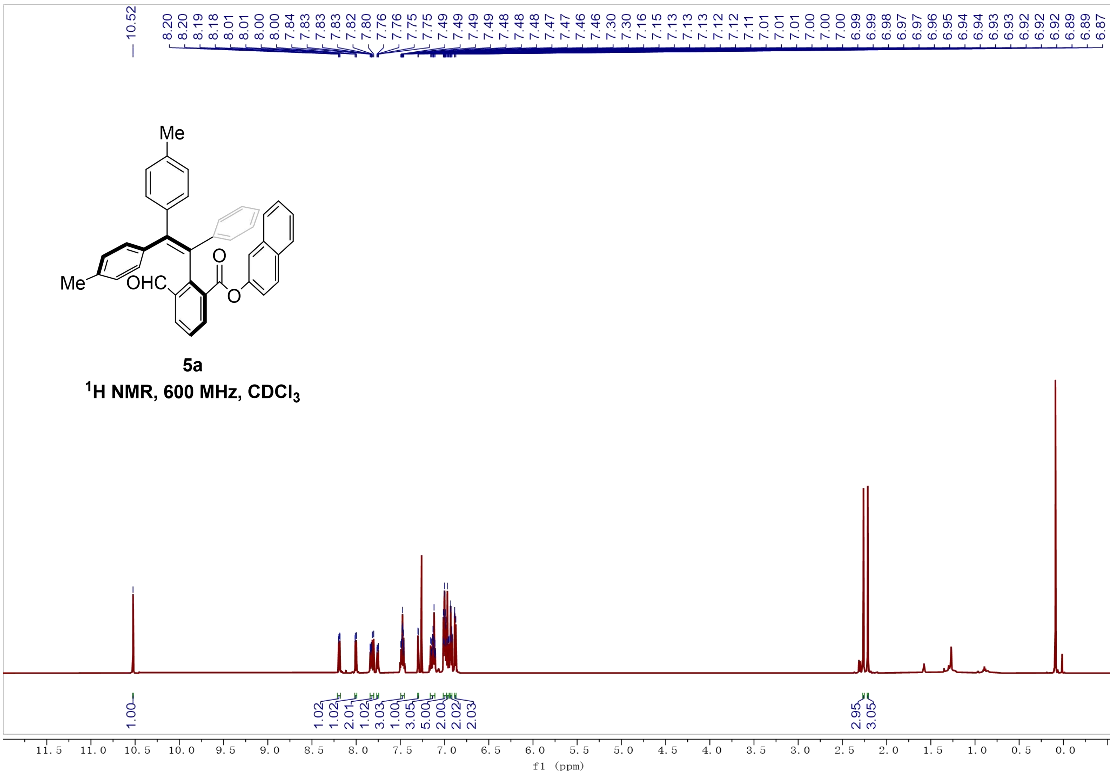
Me
Me
OHC
O
O
5a
1H NMR, 600 MHz, CDCl3
10.52
8.20 8.20 8.19 8.18 8.01 8.01 8.00 8.00 7.84 7.83 7.83 7.82 7.80 7.76 7.76 7.75 7.49 7.49 7.49 7.48 7.48 7.47 7.46 7.30 7.30 7.16 7.15 7.13 7.13 7.12 7.12 7.11 7.01 7.01 7.00 7.00 6.99 6.99 6.98 6.97 6.97 6.96 6.95 6.94 6.93 6.93 6.92 6.92 6.92 6.89 6.89 6.87
1.00 1.02 1.02 2.01 1.02 3.03 1.00 3.05 5.00 2.00 2.02 2.03 2.95 3.05
f1 (ppm)

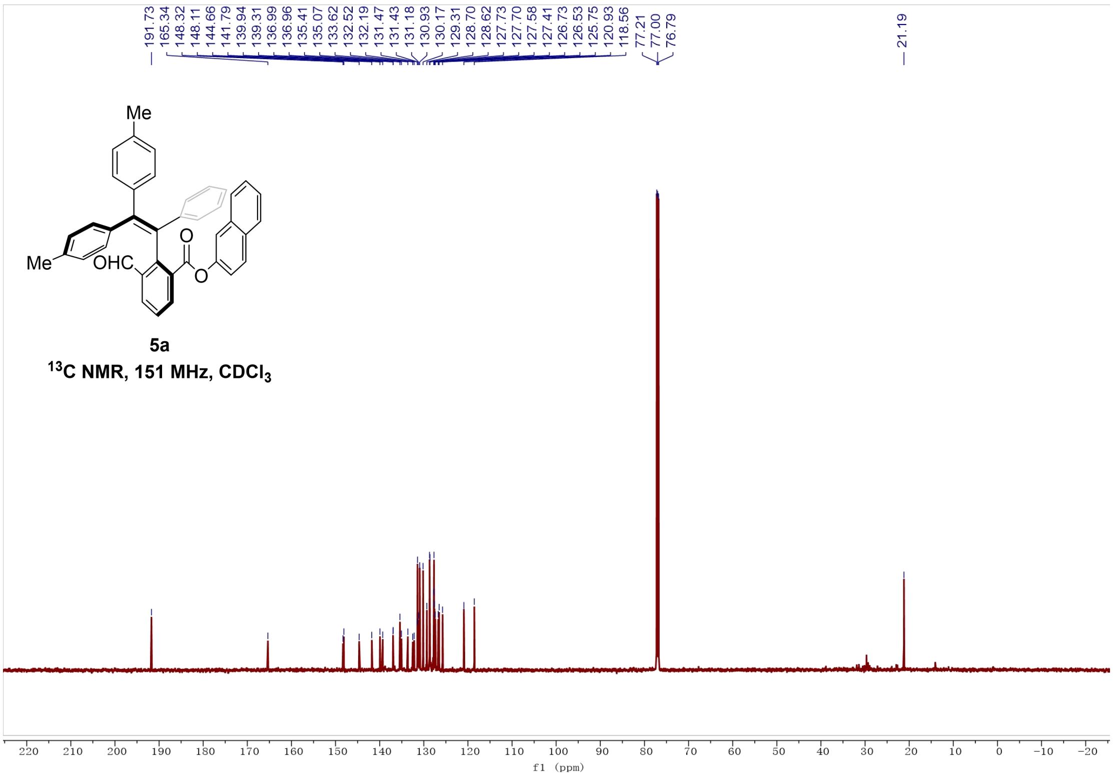
Me
Me
OHC
O
O
5a
13C NMR, 151 MHz, CDCl3
191.73 165.34 148.32 148.11 144.66 141.79 139.94 139.31 136.99 136.96 135.41 135.07 133.62 132.52 132.19 131.47 131.43 131.18 130.93 130.17 129.31 128.70 128.62 127.73 127.70 127.58 127.41 126.73 126.53 125.75 120.93 118.56 77.21 77.00 76.79 21.19
f1 (ppm)

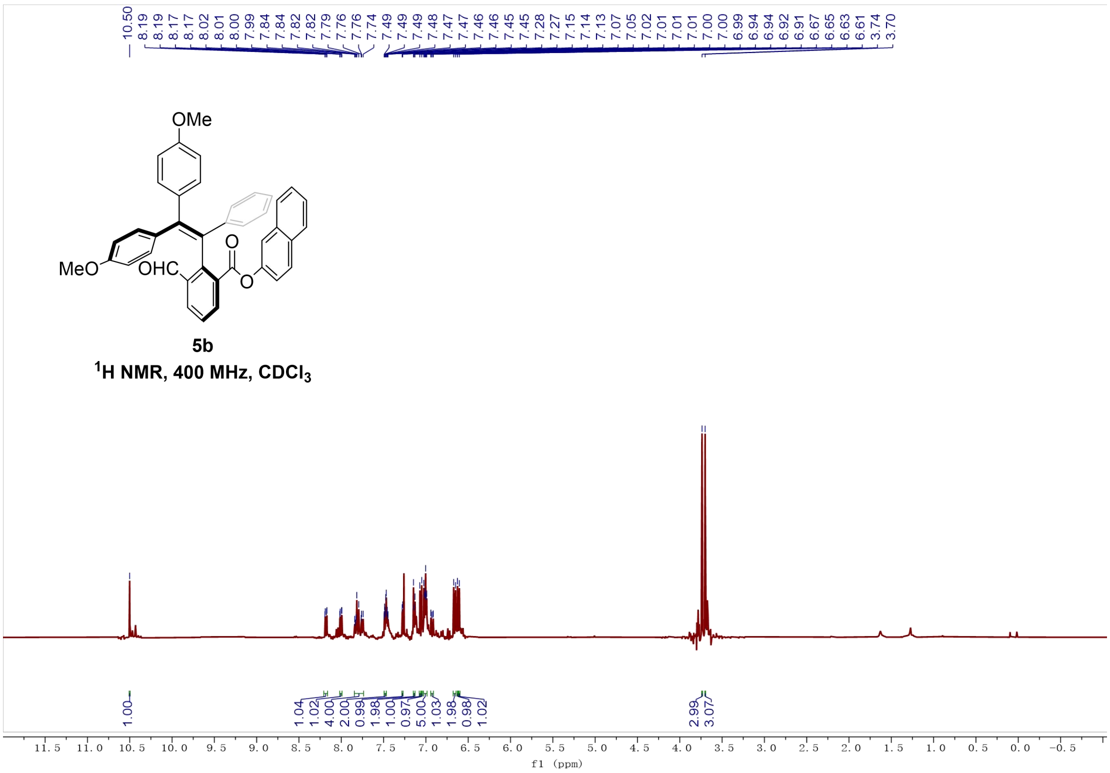
10.50 8.19 8.19 8.17 8.17 8.02 8.01 8.00 7.99 7.84 7.84 7.82 7.82 7.79 7.76 7.76 7.74 7.49 7.49 7.48 7.47 7.47 7.46 7.45 7.45 7.28 7.27 7.15 7.14 7.13 7.07 7.05 7.02 7.01 7.01 7.01 7.00 6.99 6.94 6.94 6.92 6.91 6.67 6.65 6.63 6.61 3.74 3.70
OMe
MeO
OHC
O
O
5b
^{1}H NMR, 400 MHz, $CDCl_3$
1.00 1.04 1.02 4.00 2.00 0.99 1.98 1.00 0.97 5.00 1.03 1.98 0.98 1.02 2.99 3.07
f1 (ppm)

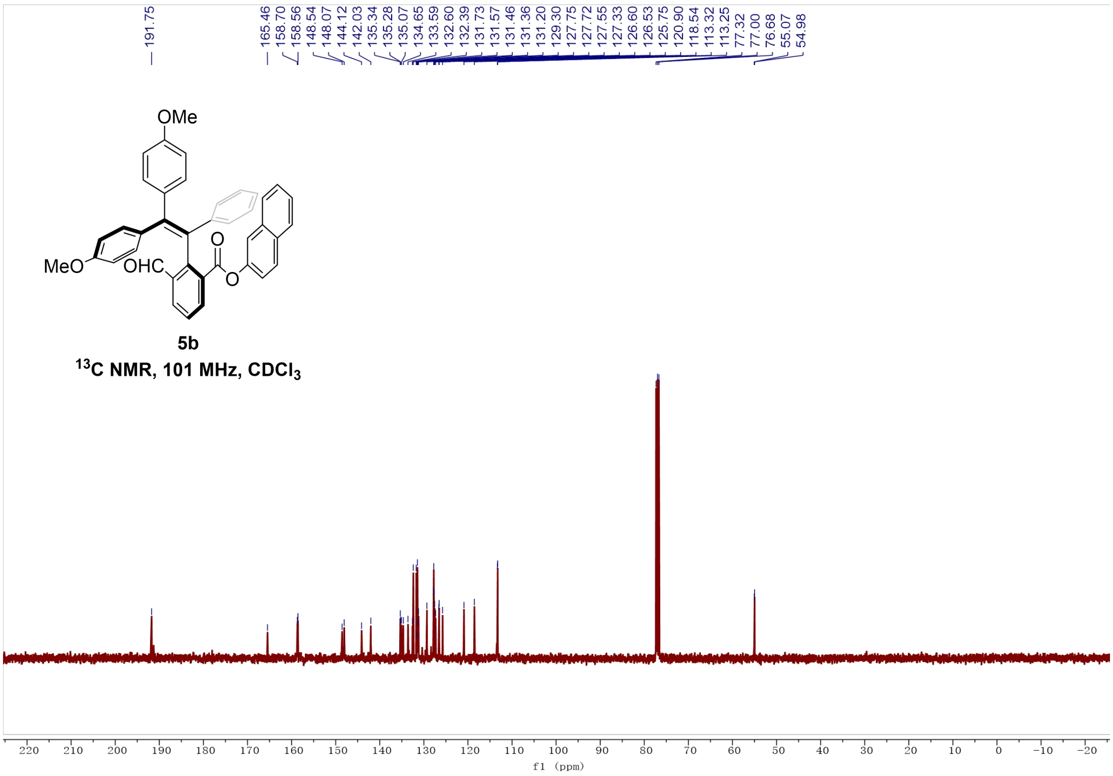
191.75 165.46 158.70 158.56 148.54 148.07 144.12 142.03 135.34 135.28 135.07 134.65 133.59 132.60 132.39 131.73 131.57 131.46 131.36 131.20 129.30 127.75 127.72 127.55 127.33 126.60 126.53 125.75 120.90 118.54 113.32 113.25 77.32 77.00 76.68 55.07 54.98
OMe
MeO
OHC
O
O
5b
^{13}C NMR, 101 MHz, $CDCl_3$
f1 (ppm)

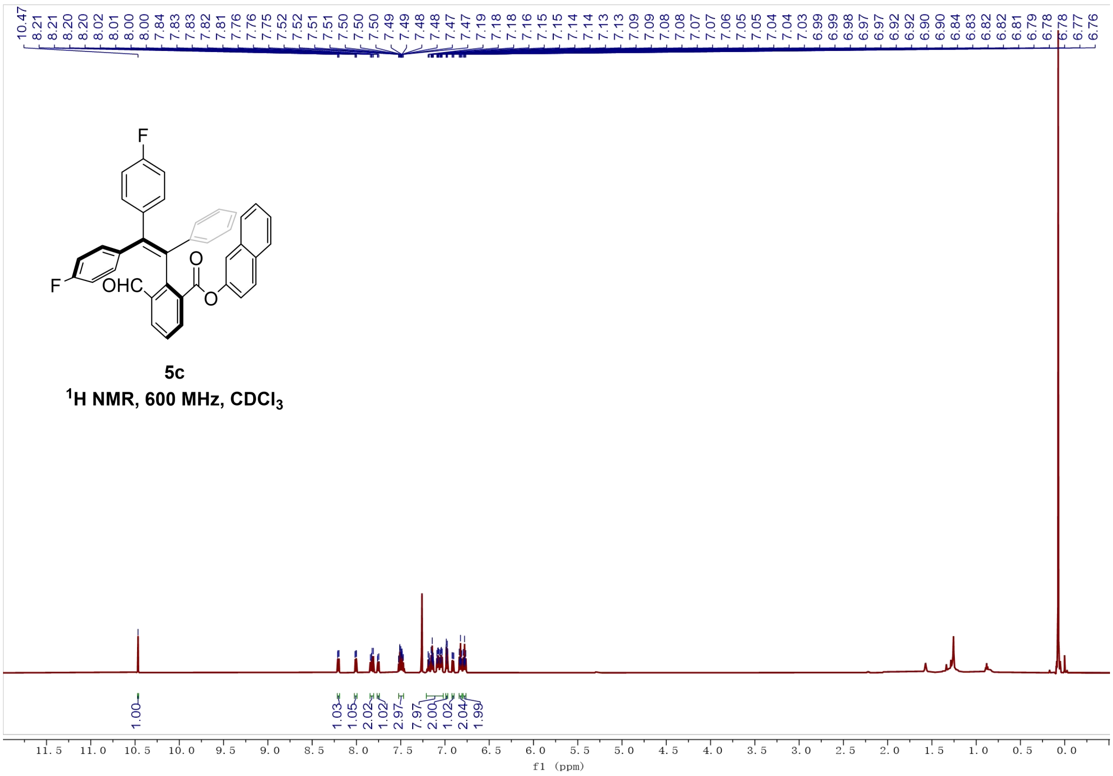
10.47 8.21 8.21 8.20 8.20 8.02 8.01 8.00 8.00 7.84 7.83 7.83 7.82 7.81 7.76 7.76 7.75 7.52 7.52 7.51 7.50 7.50 7.50 7.49 7.49 7.48 7.48 7.47 7.47 7.19 7.18 7.18 7.16 7.15 7.15 7.14 7.14 7.13 7.13 7.09 7.08 7.08 7.07 7.07 7.06 7.05 7.05 7.04 7.04 7.03 6.99 6.99 6.98 6.97 6.97 6.92 6.92 6.90 6.90 6.84 6.83 6.82 6.82 6.81 6.79 6.78 6.78 6.77 6.76
F
F
OHC
O
O
5c
1H NMR, 600 MHz, CDCl3
1.00
1.03
1.05
2.02
1.02
2.97
7.97
2.00
1.02
2.04
1.99
f1 (ppm)

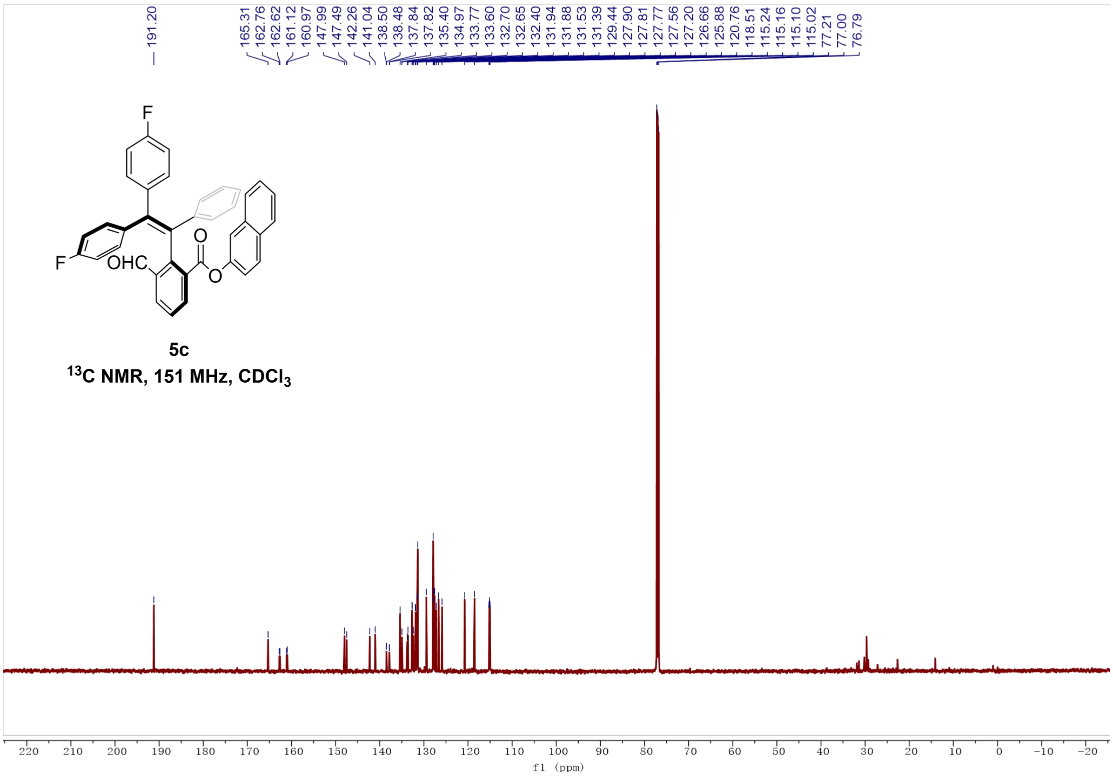
191.20
165.31 162.76 162.62 161.12 160.97 147.99 147.49 142.26 141.04 138.50 138.48 137.84 137.82 135.40 134.97 133.77 133.60 132.70 132.65 132.40 131.94 131.88 131.53 131.39 129.44 127.90 127.81 127.77 127.56 127.20 126.66 125.88 120.76 118.51 115.24 115.16 115.10 115.02 77.21 77.00 76.79
F
F
OHC
O
O
5c
13C NMR, 151 MHz, CDCl3
f1 (ppm)

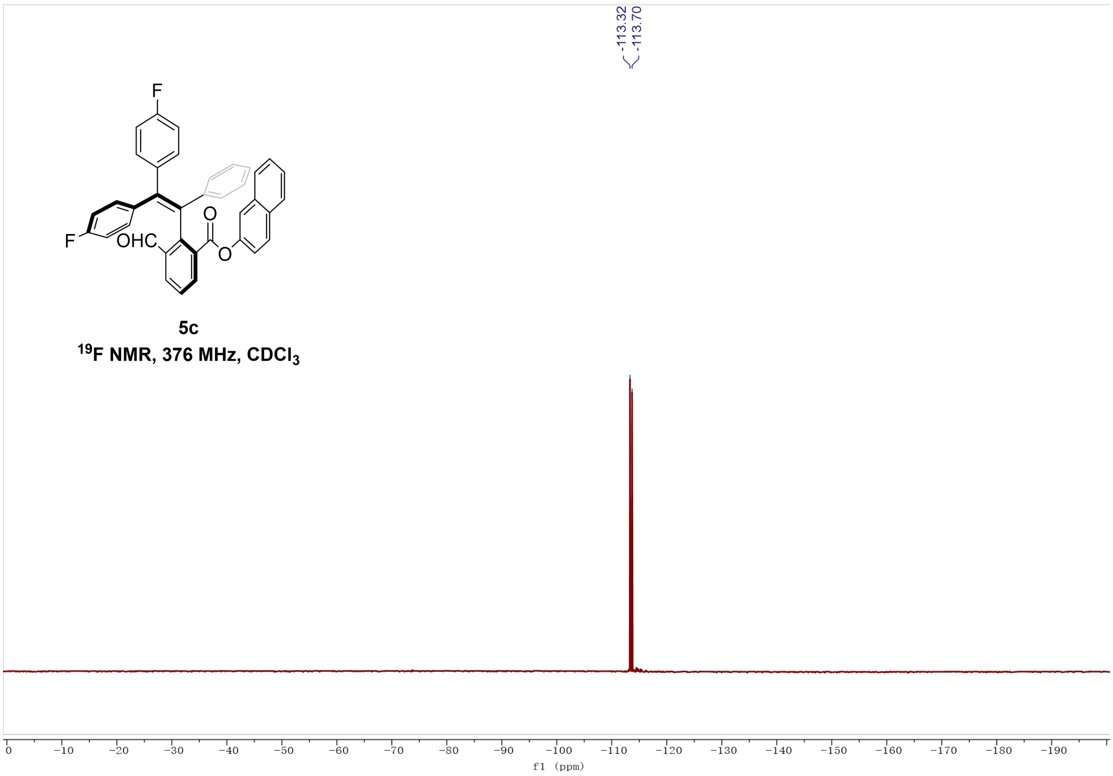
-113.32
-113.70
F
F
OHC
O
O
5c
19F NMR, 376 MHz, CDCl3
f1 (ppm)

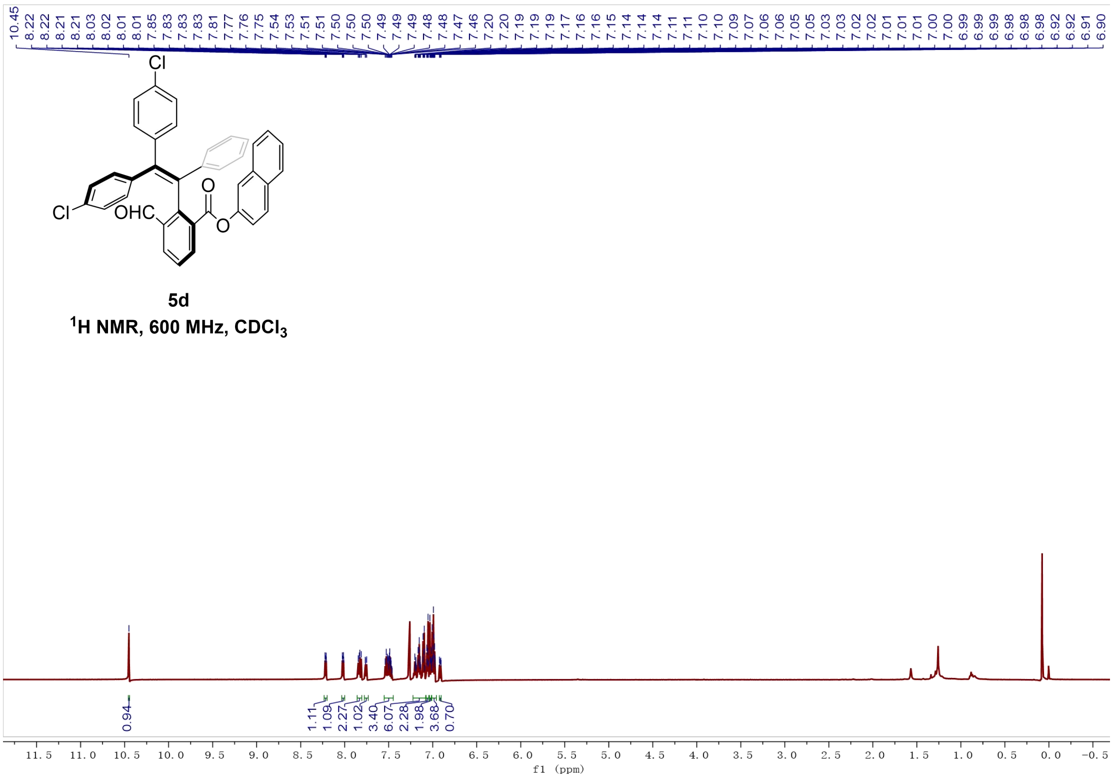
10.45 8.22 8.22 8.21 8.03 8.02 8.01 7.85 7.83 7.83 7.81 7.77 7.76 7.75 7.54 7.53 7.51 7.50 7.50 7.49 7.49 7.48 7.48 7.47 7.46 7.20 7.20 7.19 7.19 7.17 7.16 7.15 7.14 7.14 7.11 7.10 7.10 7.09 7.07 7.06 7.05 7.05 7.03 7.02 7.02 7.01 7.01 7.00 7.00 6.99 6.99 6.98 6.98 6.92 6.92 6.91 6.90
Cl
Cl
OHC
O
O
5d
1H NMR, 600 MHz, CDCl3
0.94
1.11
1.09
2.27
1.02
3.40
6.07
2.28
1.98
3.68
0.70
f1 (ppm)

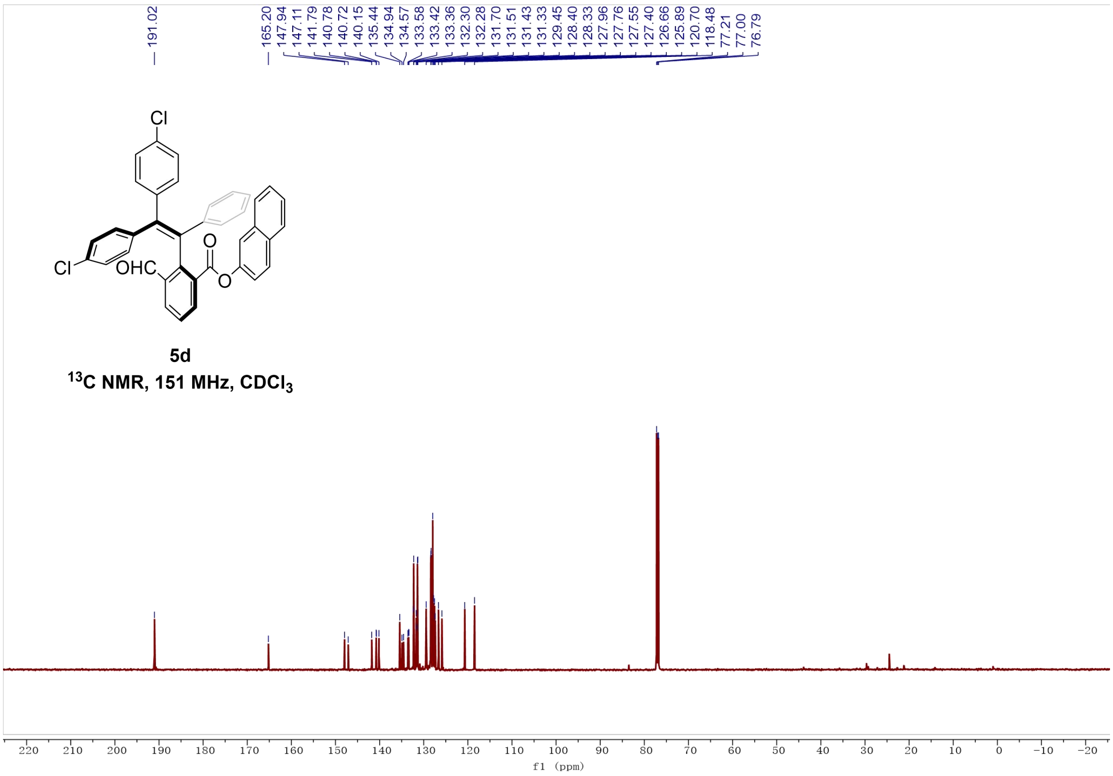

191.02
165.20
147.94
147.11
141.79
140.78
140.72
140.15
135.44
134.94
134.57
133.58
133.42
133.36
132.30
132.28
131.70
131.51
131.43
131.33
129.45
128.40
128.33
127.96
127.76
127.55
127.40
126.66
125.89
120.70
118.48
77.21
77.00
76.79
Cl
Cl
OHC
O
O
5d
^{13}C NMR, 151 MHz, CDCl$_3$
f1 (ppm)

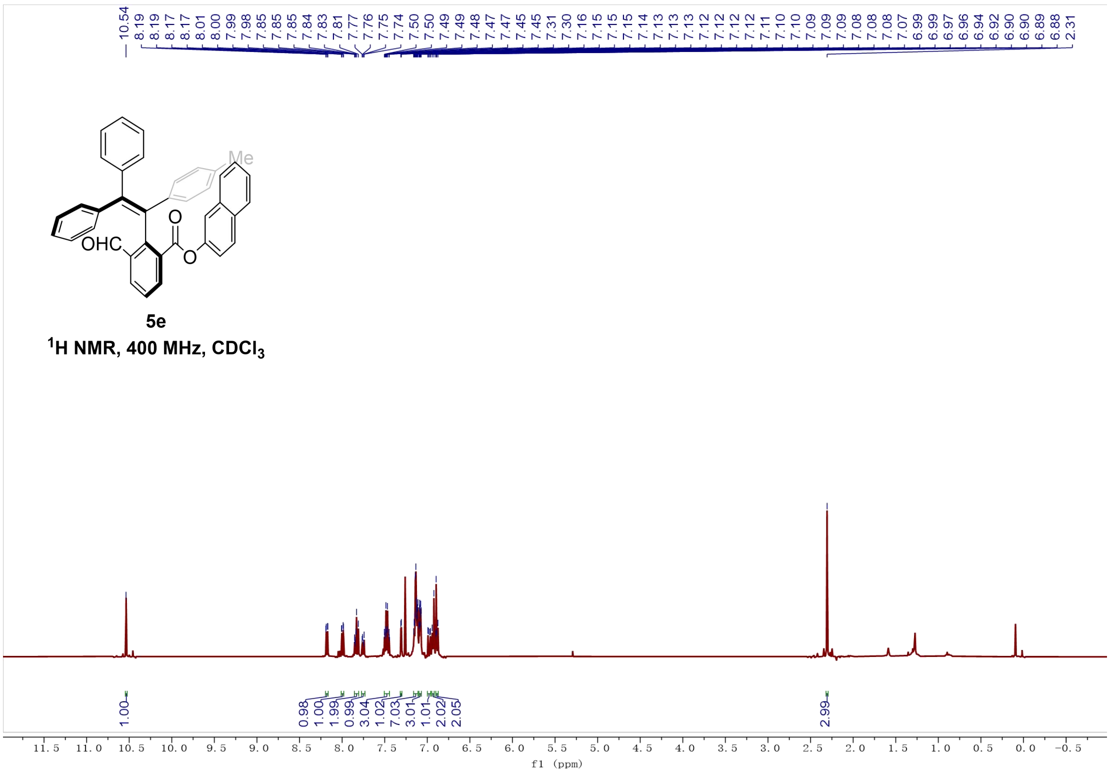

10.54
8.19
8.19
8.17
8.17
8.01
8.00
7.99
7.98
7.85
7.85
7.84
7.83
7.81
7.77
7.76
7.75
7.74
7.50
7.50
7.49
7.48
7.47
7.47
7.45
7.45
7.31
7.30
7.16
7.15
7.15
7.14
7.13
7.13
7.12
7.12
7.12
7.11
7.10
7.09
7.09
7.08
7.08
7.08
7.07
6.99
6.97
6.96
6.94
6.92
6.90
6.90
6.89
6.88
2.31
Me
OHC
O
O
5e
^{1}H NMR, 400 MHz, CDCl$_3$
1.00
0.98
1.00
1.99
0.99
3.04
1.02
7.03
3.01
1.01
2.02
2.05
2.99
f1 (ppm)

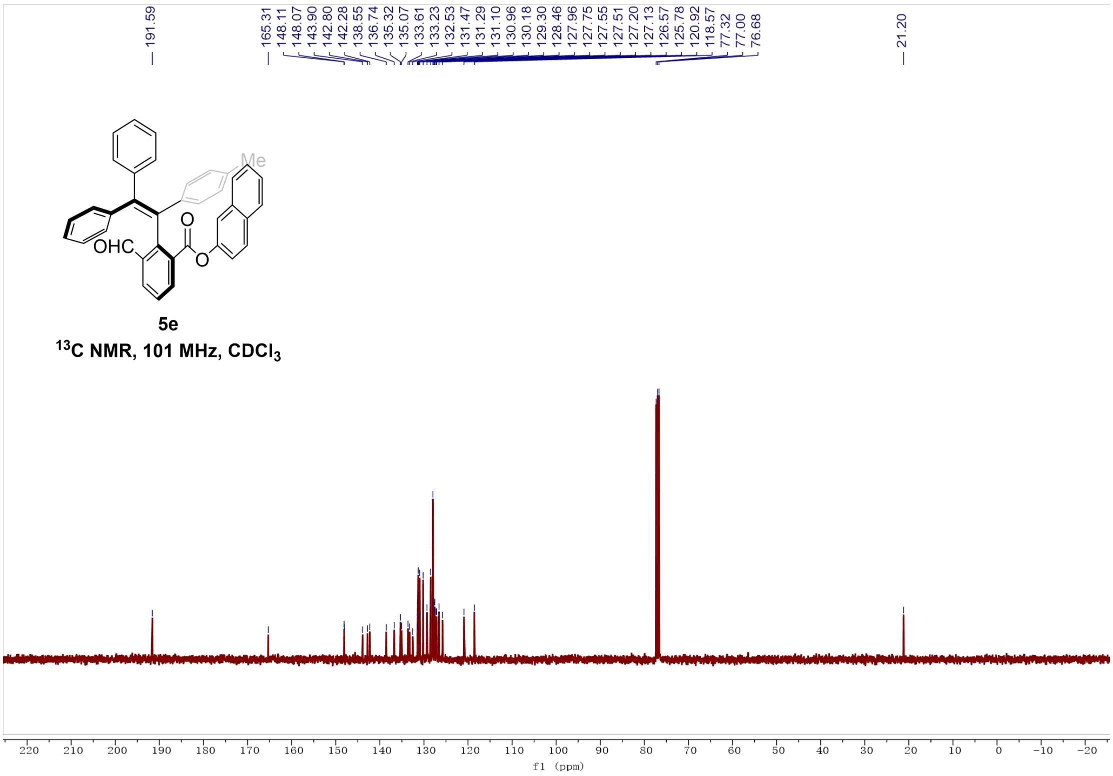

5e

^{13}C NMR, 101 MHz, CDCl$_3$

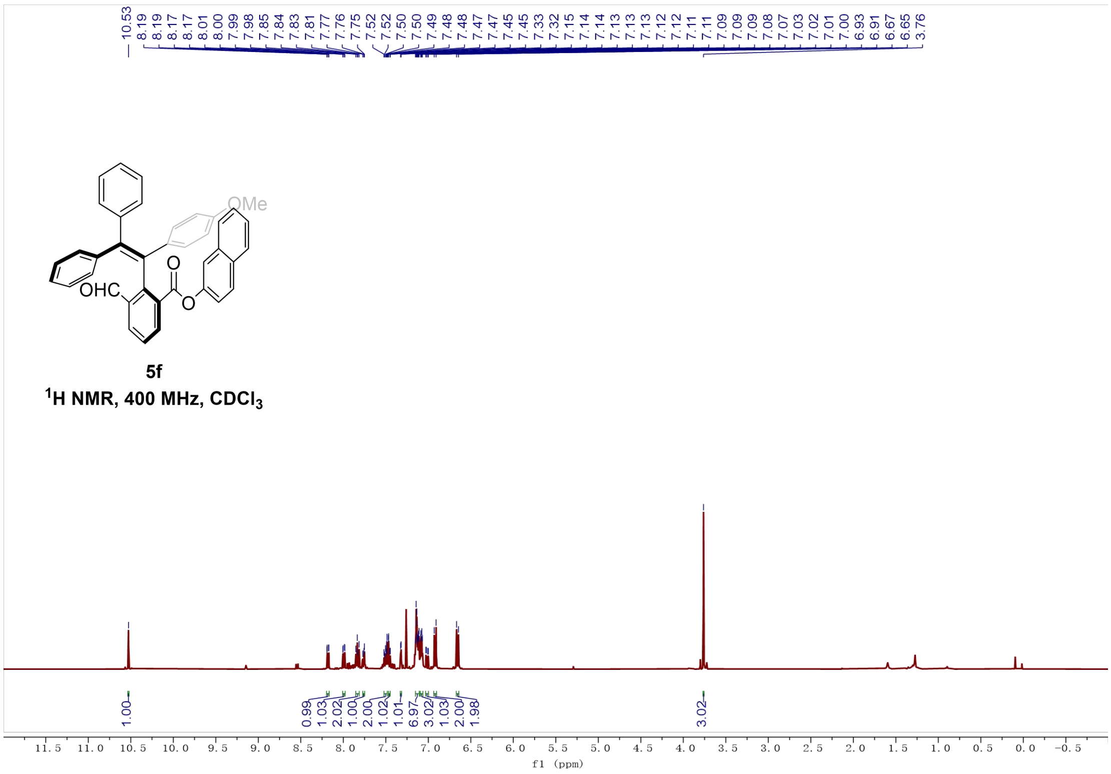

5f

^{1}H NMR, 400 MHz, CDCl$_3$

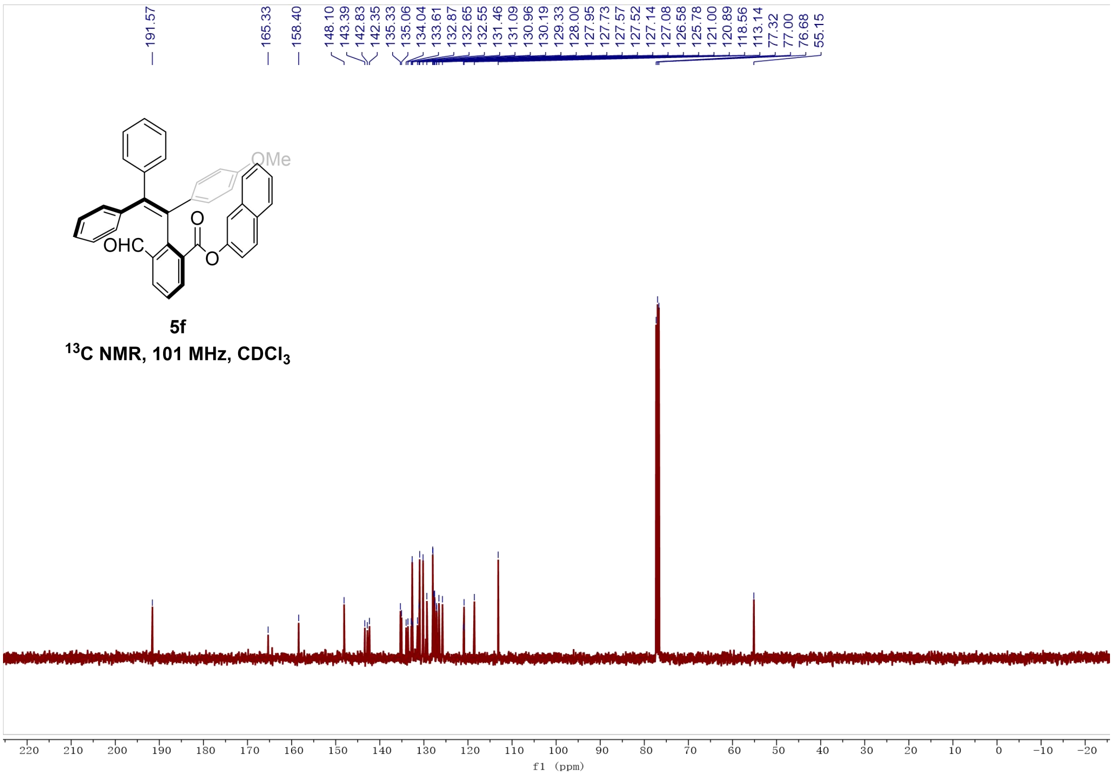

5f

^{13}C NMR, 101 MHz, CDCl$_3$

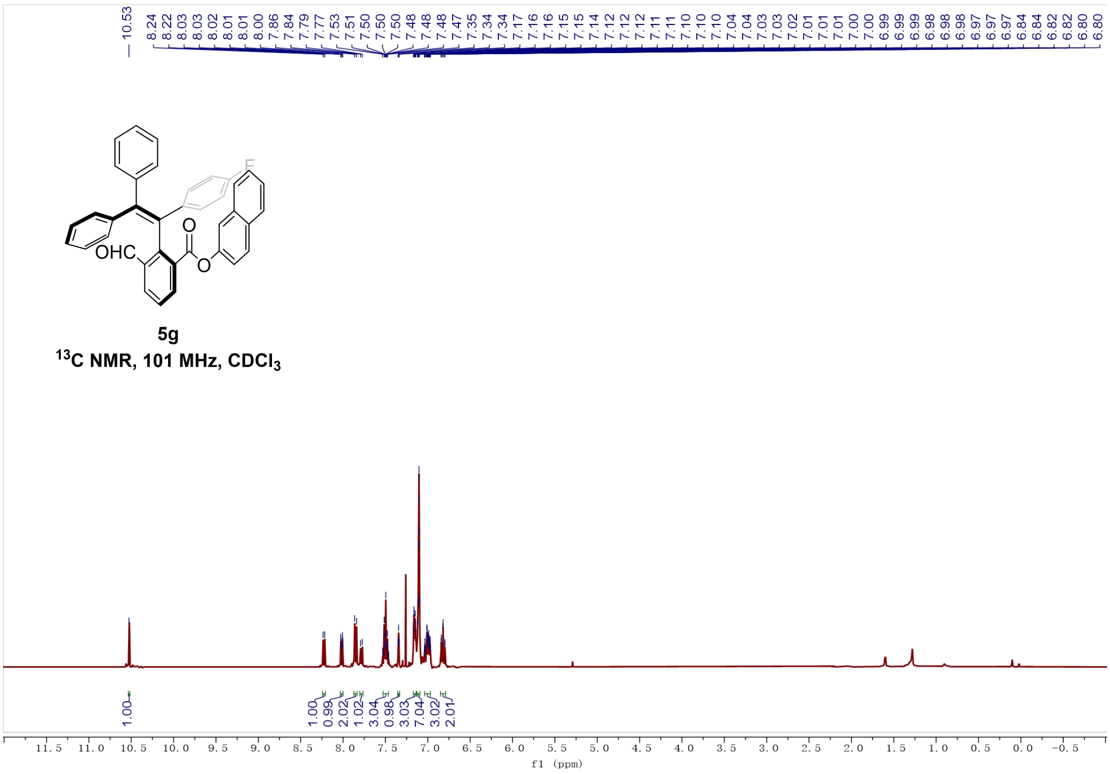

5g

^{13}C NMR, 101 MHz, CDCl$_3$

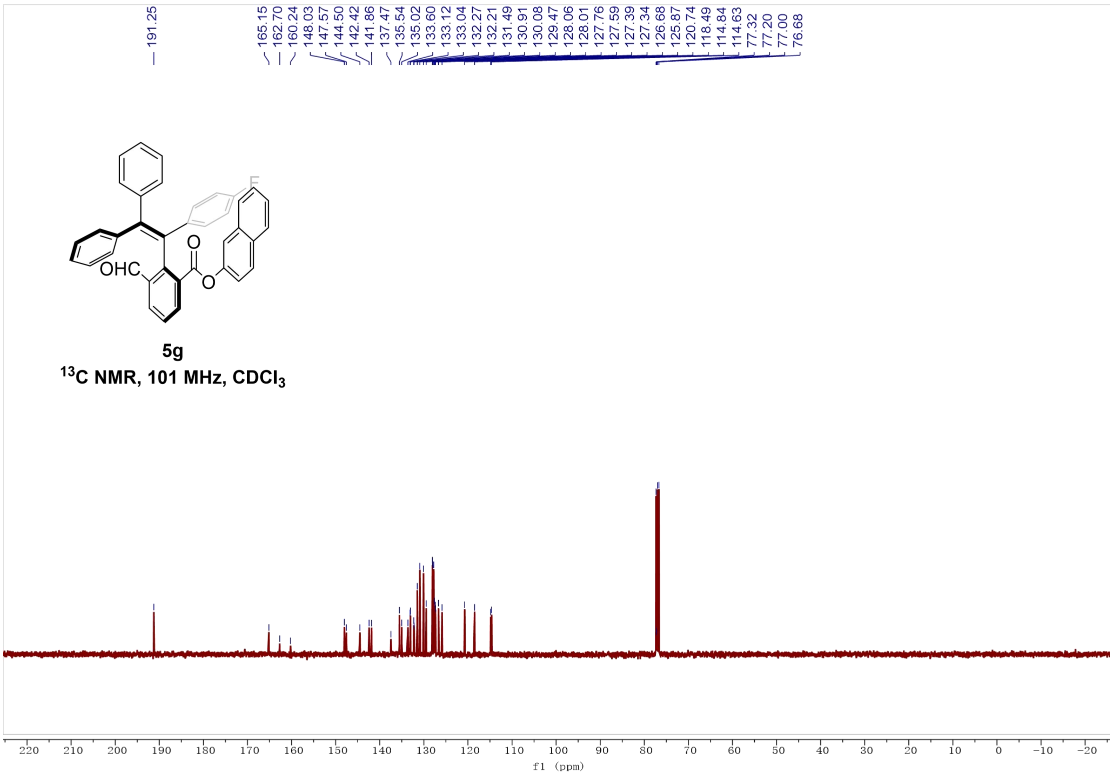
191.25
165.15
162.70
160.24
148.03
147.57
144.50
142.42
141.86
137.47
135.54
135.02
133.60
133.12
133.04
132.27
132.21
131.49
130.91
130.08
129.47
128.06
128.01
127.76
127.59
127.39
127.34
126.68
125.87
120.74
118.49
114.84
114.63
77.32
77.20
77.00
76.68
OHC
O
O
F
5g
^{13}C NMR, 101 MHz, CDCl$_3$
f1 (ppm)

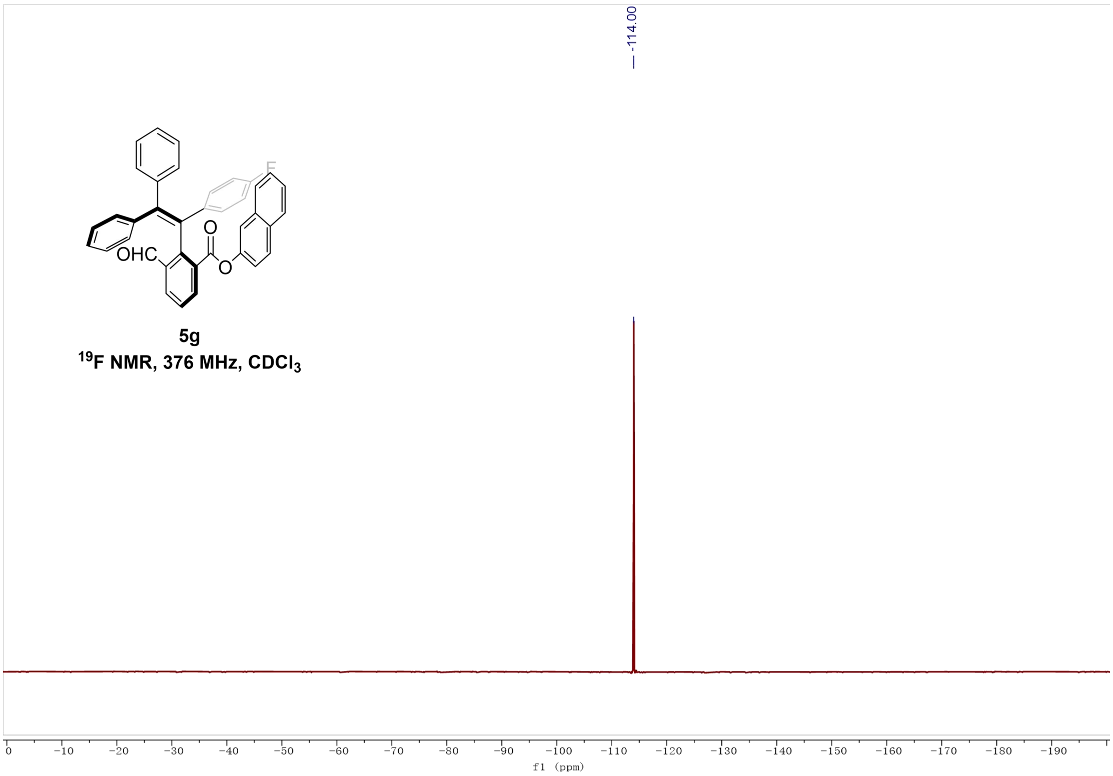
-114.00
OHC
O
O
F
5g
^{19}F NMR, 376 MHz, CDCl$_3$
f1 (ppm)

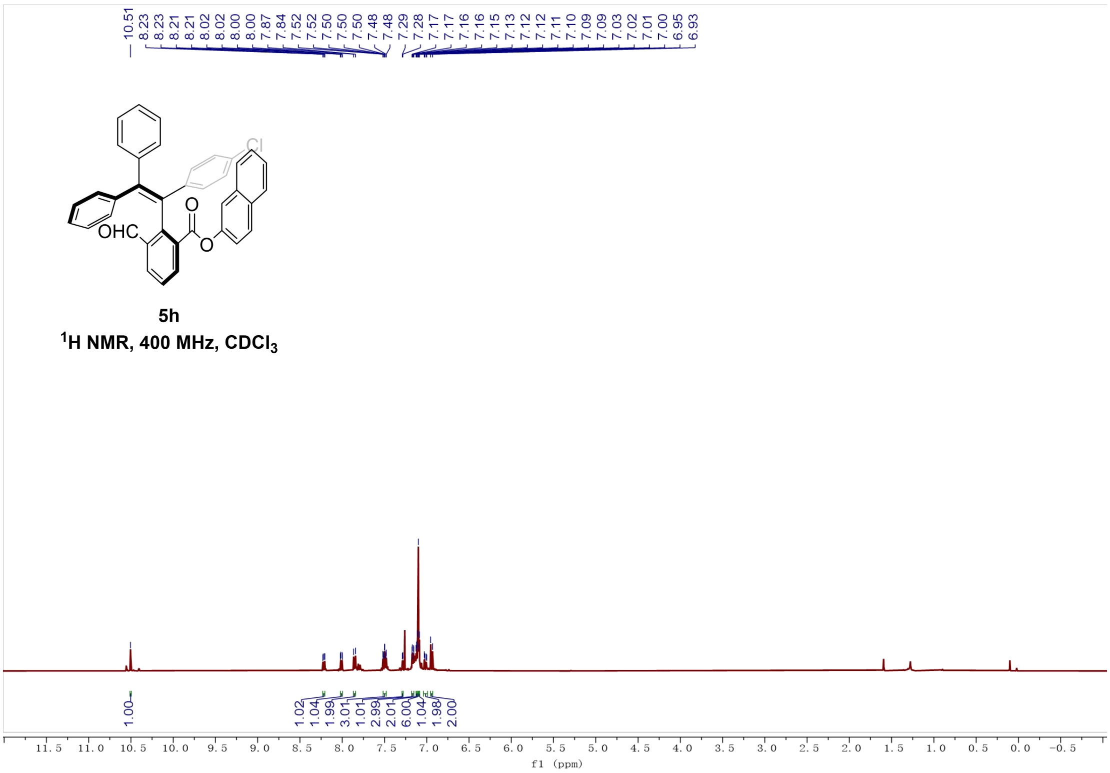
10.51 8.23 8.23 8.21 8.02 8.02 8.00 7.87 7.84 7.52 7.52 7.50 7.50 7.48 7.29 7.28 7.17 7.16 7.15 7.13 7.12 7.12 7.11 7.10 7.09 7.03 7.02 7.01 7.00 6.95 6.93
OHC
O
O
Cl
5h
^{1}H NMR, 400 MHz, $CDCl_3$
1.00 1.02 1.04 1.99 3.01 1.01 2.99 2.01 6.00 1.04 1.98 2.00
f1 (ppm)

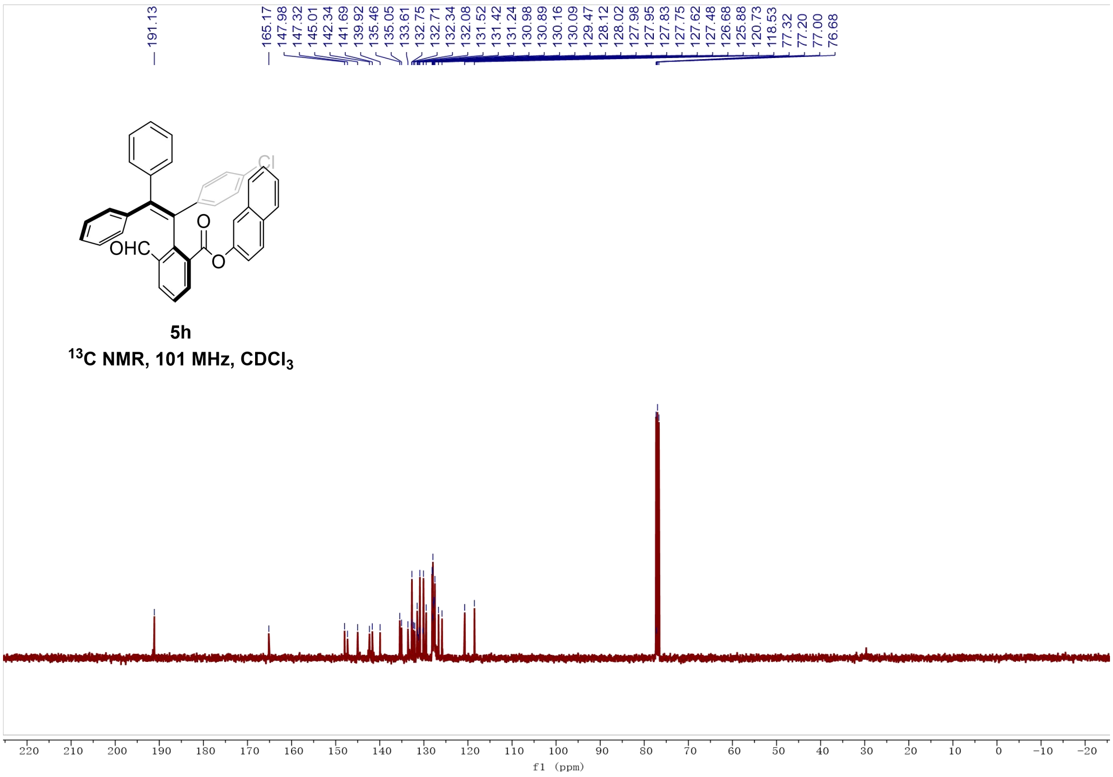
191.13
165.17 147.98 147.32 145.01 142.34 141.69 139.92 135.46 135.05 133.61 132.75 132.71 132.34 132.08 131.52 131.42 131.24 130.98 130.89 130.16 130.09 129.47 128.12 128.02 127.98 127.95 127.83 127.75 127.62 127.48 126.68 125.88 120.73 118.53 77.32 77.20 77.00 76.68
OHC
O
O
Cl
5h
^{13}C NMR, 101 MHz, $CDCl_3$
f1 (ppm)

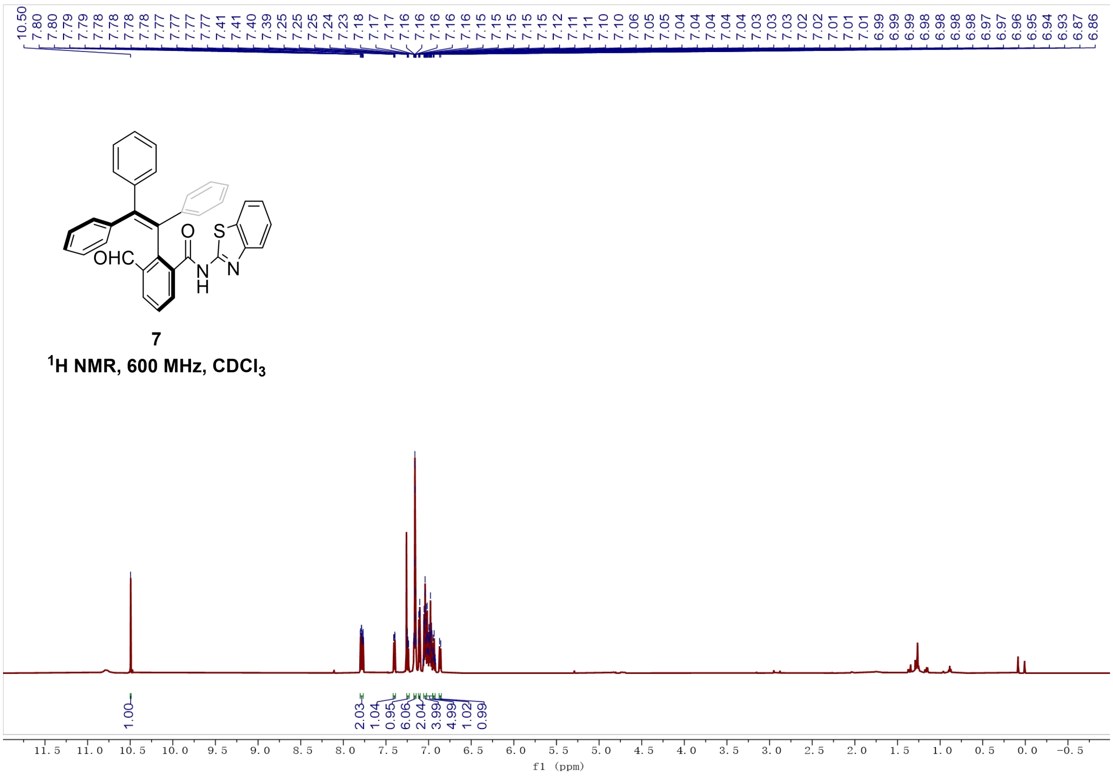

7

^{1}H NMR, 600 MHz, $CDCl_3$

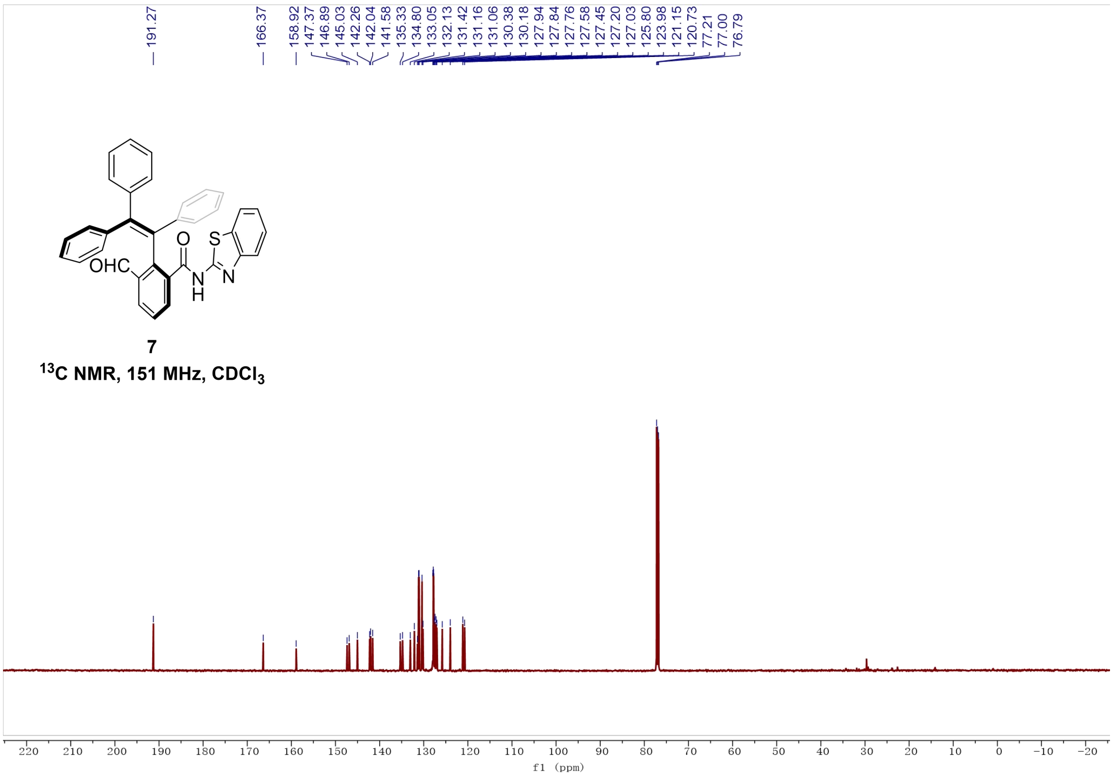

7

^{13}C NMR, 151 MHz, $CDCl_3$

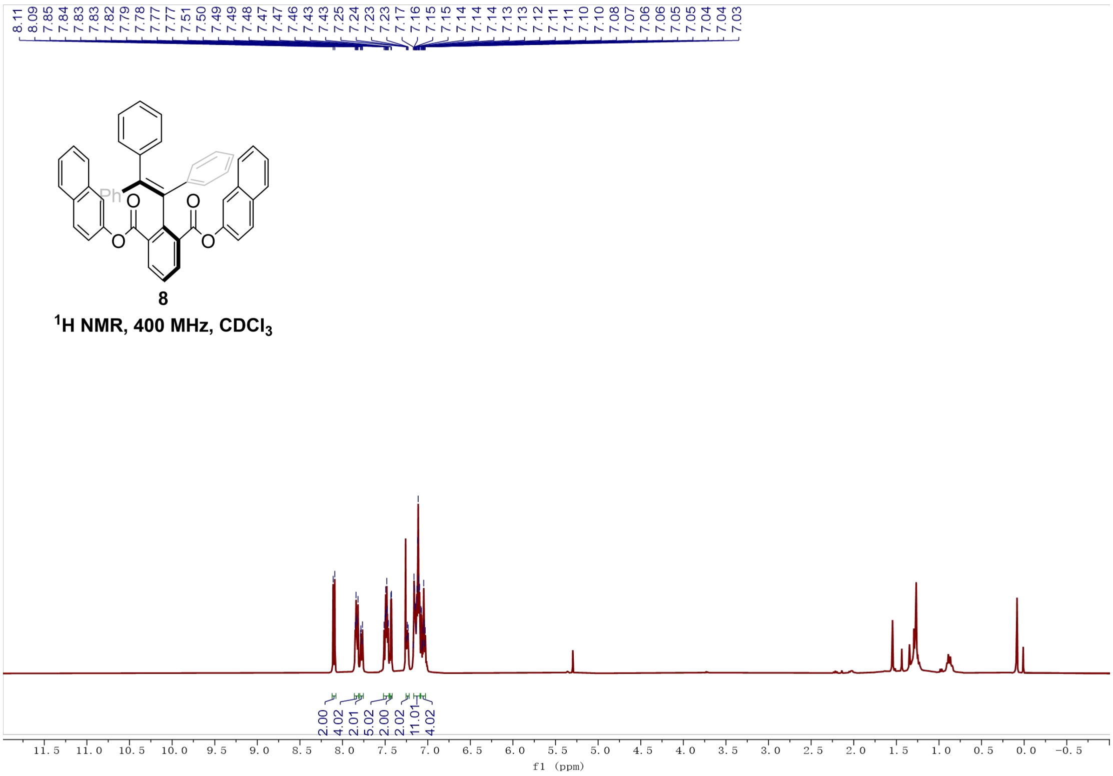
8.11 8.09 7.85 7.84 7.83 7.83 7.82 7.79 7.78 7.77 7.77 7.51 7.50 7.49 7.49 7.48 7.47 7.47 7.46 7.43 7.43 7.25 7.24 7.23 7.23 7.17 7.16 7.15 7.14 7.14 7.14 7.13 7.13 7.12 7.11 7.11 7.10 7.10 7.08 7.07 7.06 7.06 7.05 7.05 7.04 7.04 7.03
Ph
O
O
O
O
8
^{1}H NMR, 400 MHz, CDCl$_3$
2.00 4.02 2.01 5.02 2.00 2.02 11.01 4.02
f1 (ppm)

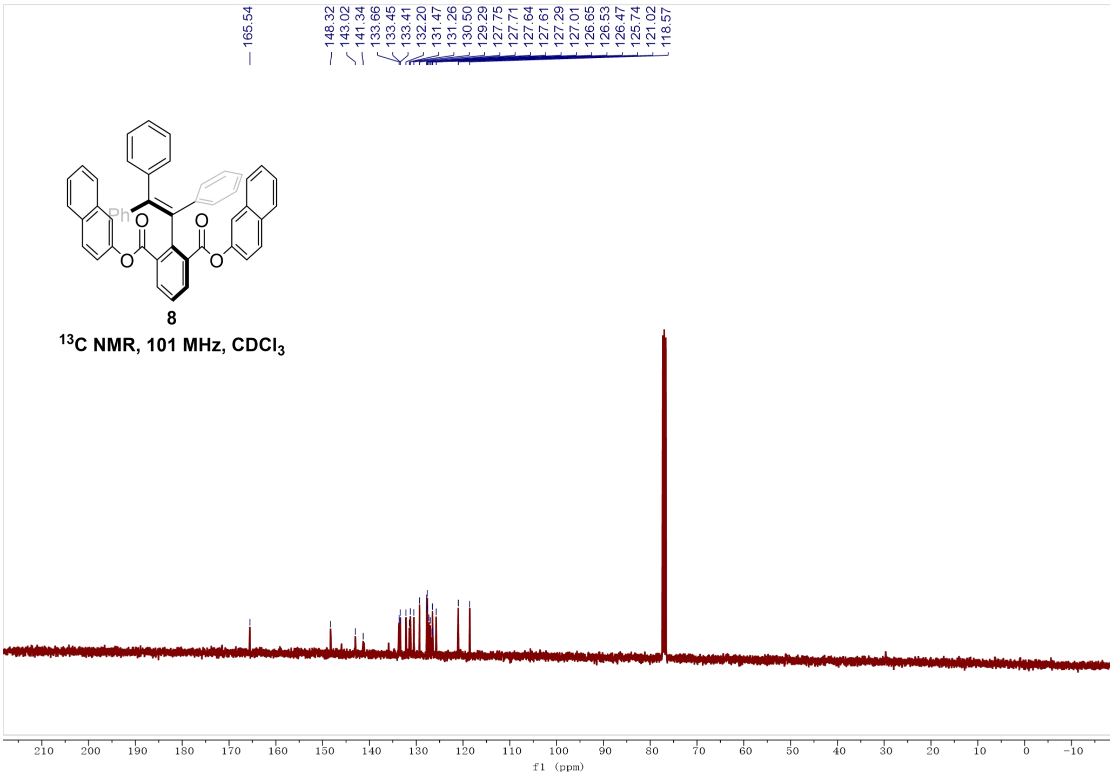
165.54
148.32 143.02 141.34 133.66 133.45 133.41 132.20 131.47 131.26 130.50 129.29 127.75 127.71 127.64 127.61 127.29 127.01 126.65 126.53 126.47 125.74 121.02 118.57
Ph
O
O
O
O
8
^{13}C NMR, 101 MHz, CDCl$_3$
f1 (ppm)

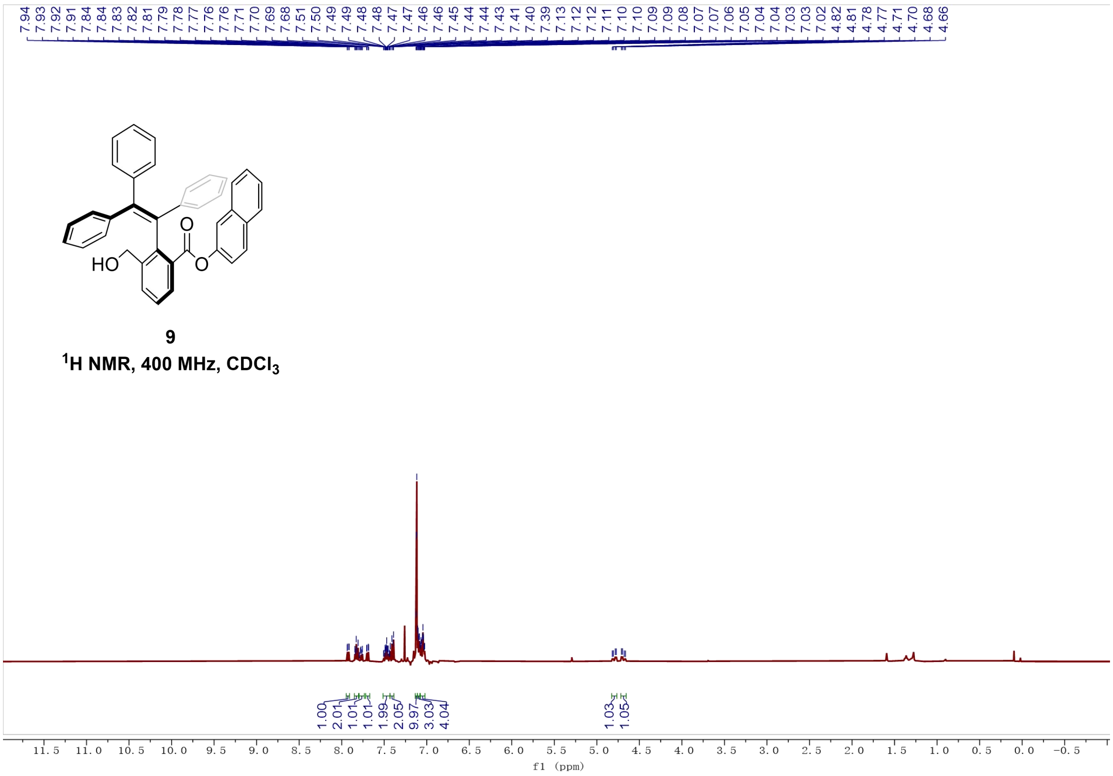
7.94 7.93 7.92 7.91 7.84 7.84 7.83 7.82 7.81 7.79 7.78 7.77 7.76 7.76 7.71 7.70 7.69 7.68 7.51 7.50 7.49 7.49 7.48 7.48 7.47 7.47 7.46 7.46 7.45 7.44 7.44 7.43 7.41 7.40 7.39 7.13 7.12 7.12 7.11 7.10 7.10 7.09 7.09 7.08 7.07 7.07 7.06 7.05 7.04 7.04 7.03 7.03 7.02 4.82 4.81 4.78 4.77 4.71 4.70 4.68 4.66
HO
O
O
9
1H NMR, 400 MHz, CDCl3
1.00 2.01 1.01 1.01 1.99 2.05 9.97 3.03 4.04 1.03 1.05
f1 (ppm)

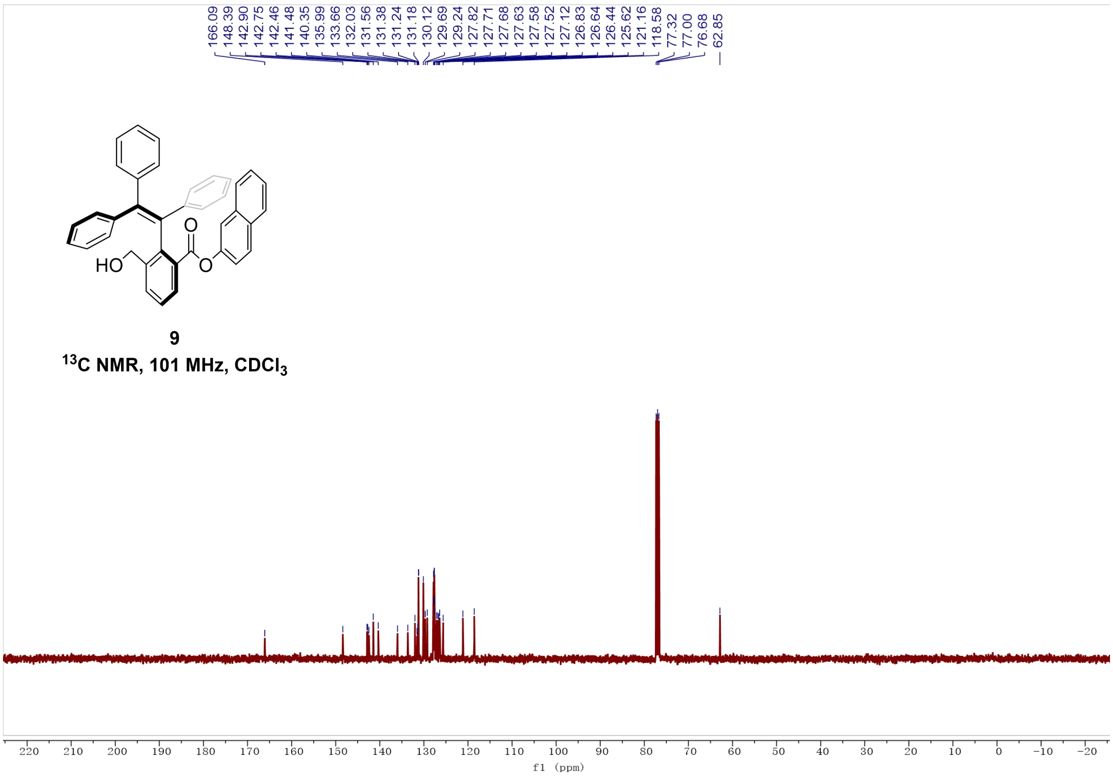
166.09 148.39 142.90 142.75 142.46 141.48 140.35 135.99 133.66 132.03 131.56 131.38 131.24 131.18 130.12 129.69 129.24 127.82 127.71 127.68 127.63 127.58 127.52 127.12 126.83 126.64 126.44 125.62 121.16 118.58 77.32 77.00 76.68 62.85
HO
O
O
9
13C NMR, 101 MHz, CDCl3
f1 (ppm)

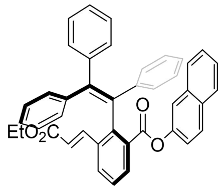

10

^{1}H NMR, 600 MHz, CDCl3

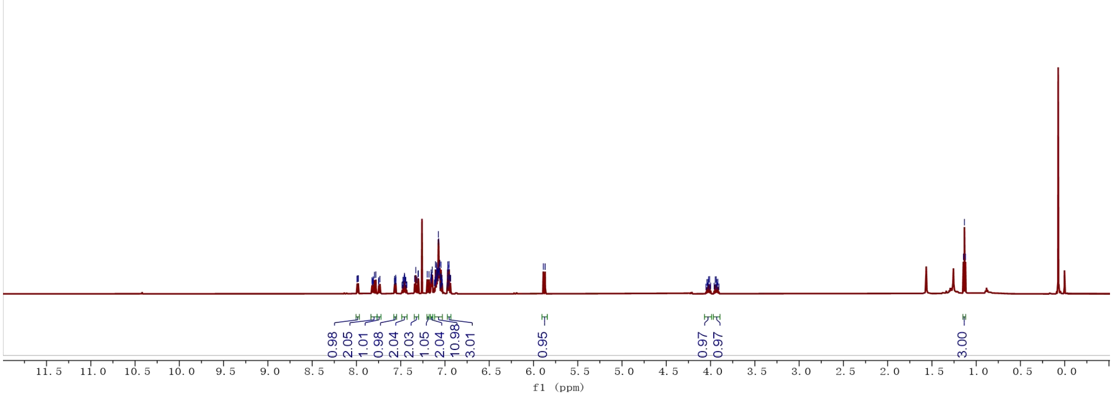

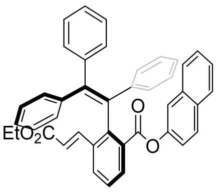

10

^{13}C NMR, 151 MHz, CDCl3

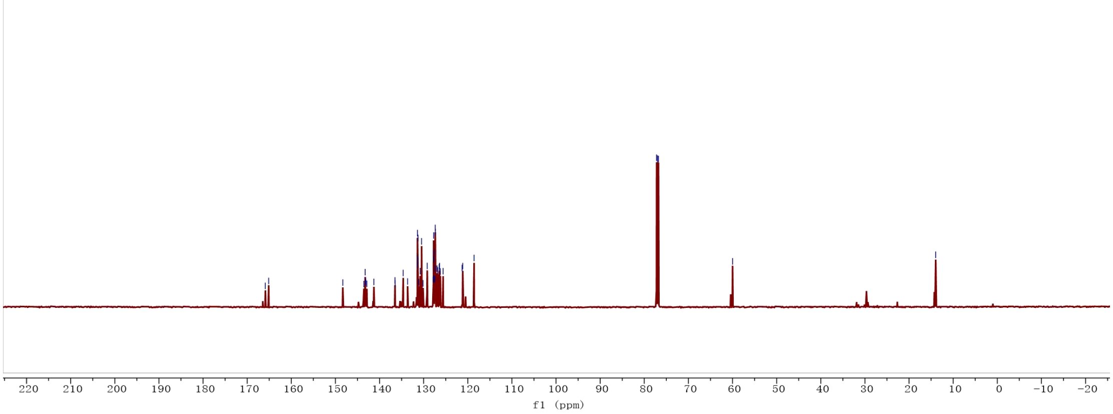

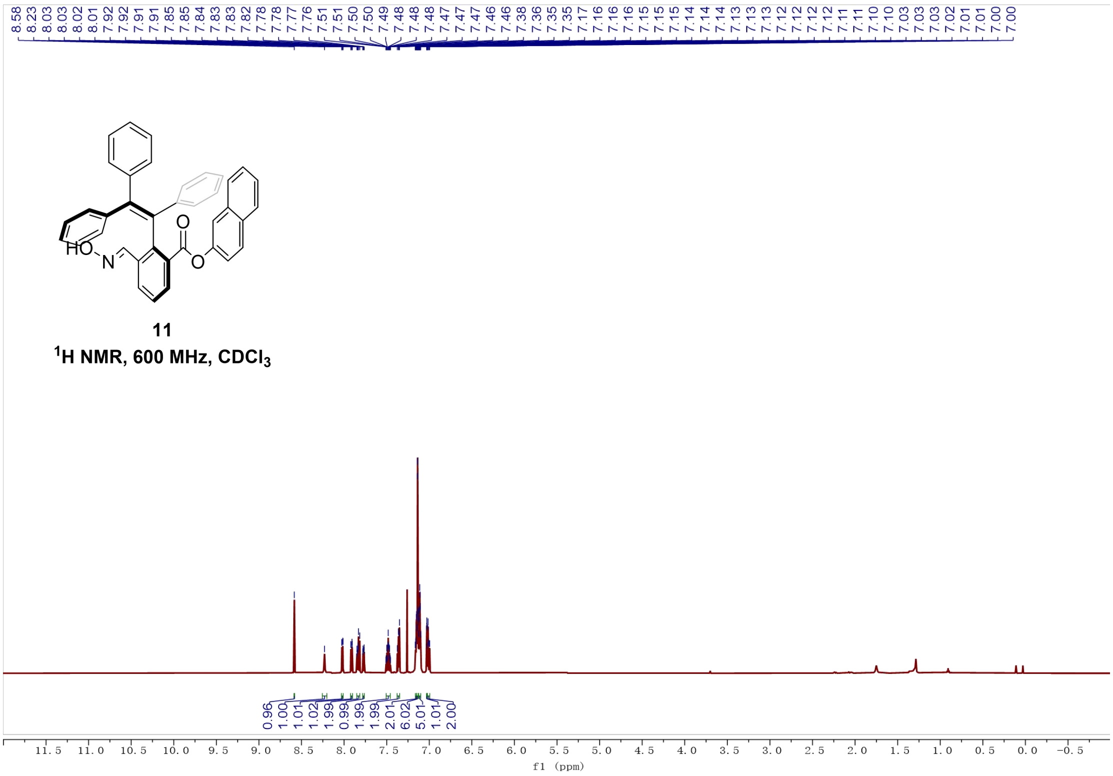
8.58 8.23 8.03 8.03 8.02 8.01 7.92 7.92 7.91 7.91 7.85 7.85 7.84 7.83 7.83 7.82 7.78 7.78 7.77 7.76 7.51 7.51 7.50 7.50 7.49 7.48 7.48 7.48 7.47 7.47 7.46 7.38 7.36 7.35 7.35 7.17 7.16 7.16 7.16 7.15 7.15 7.15 7.14 7.14 7.13 7.13 7.13 7.12 7.12 7.12 7.12 7.11 7.11 7.10 7.10 7.03 7.03 7.03 7.02 7.01 7.01 7.00 7.00
HO
N
O
O
11
1H NMR, 600 MHz, CDCl3
0.96 1.00 1.01 1.02 1.99 0.99 1.99 1.99 2.01 6.02 5.01 1.01 2.00
11.5 11.0 10.5 10.0 9.5 9.0 8.5 8.0 7.5 7.0 6.5 6.0 5.5 5.0 4.5 4.0 3.5 3.0 2.5 2.0 1.5 1.0 0.5 0.0 -0.5
f1 (ppm)

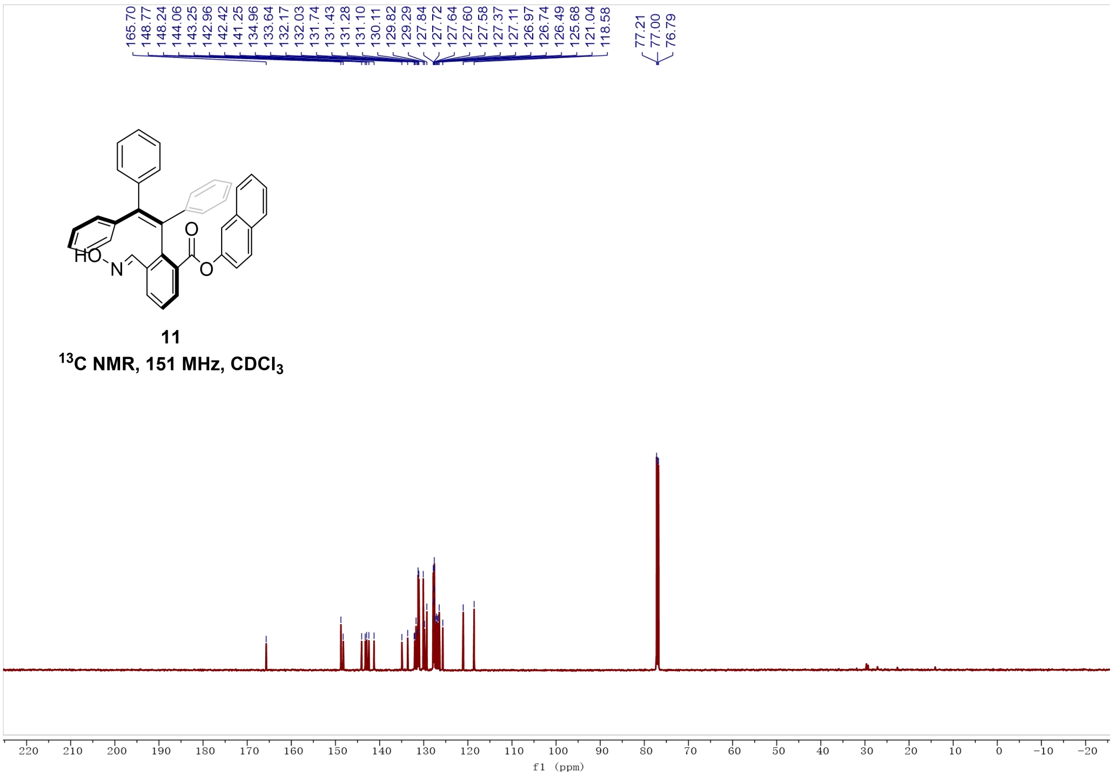
165.70 148.77 148.24 144.06 143.25 142.96 142.42 141.25 134.96 133.64 132.17 132.03 131.74 131.43 131.28 131.10 130.11 129.82 129.29 127.84 127.72 127.64 127.60 127.58 127.37 127.11 126.97 126.74 126.49 125.68 121.04 118.58
77.21 77.00 76.79
HO
N
O
O
11
13C NMR, 151 MHz, CDCl3
220 210 200 190 180 170 160 150 140 130 120 110 100 90 80 70 60 50 40 30 20 10 0 -10 -20
f1 (ppm)

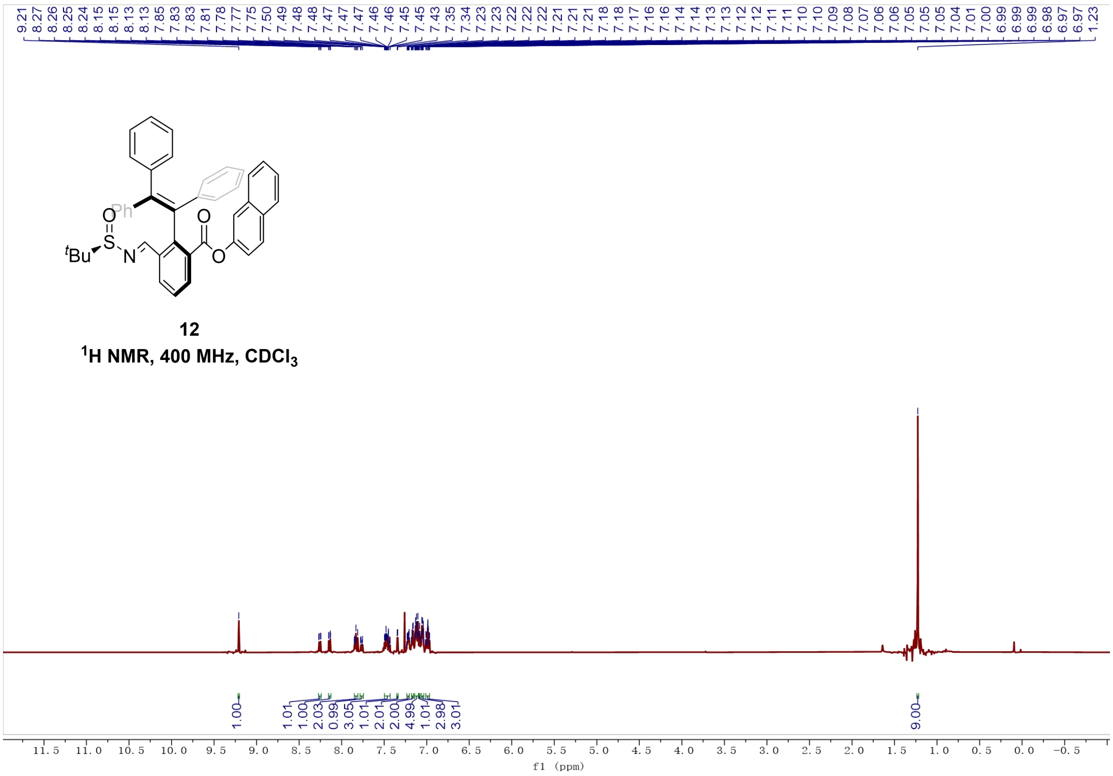
9.21 8.27 8.26 8.25 8.24 8.15 8.15 8.13 8.13 7.85 7.83 7.83 7.81 7.78 7.77 7.75 7.50 7.49 7.48 7.48 7.47 7.47 7.46 7.45 7.45 7.43 7.35 7.34 7.23 7.23 7.22 7.22 7.22 7.21 7.21 7.21 7.18 7.18 7.17 7.16 7.16 7.14 7.14 7.13 7.13 7.12 7.12 7.11 7.11 7.10 7.10 7.09 7.08 7.07 7.06 7.06 7.05 7.05 7.05 7.04 7.01 7.00 6.99 6.99 6.99 6.98 6.97 6.97 1.23
O
Ph
S
tBu
N
O
O
12
1H NMR, 400 MHz, CDCl3
1.00 1.01 1.00 2.03 0.99 3.05 1.01 2.01 2.00 4.99 1.01 2.98 3.01 9.00
11.5 11.0 10.5 10.0 9.5 9.0 8.5 8.0 7.5 7.0 6.5 6.0 5.5 5.0 4.5 4.0 3.5 3.0 2.5 2.0 1.5 1.0 0.5 0.0 -0.5
f1 (ppm)

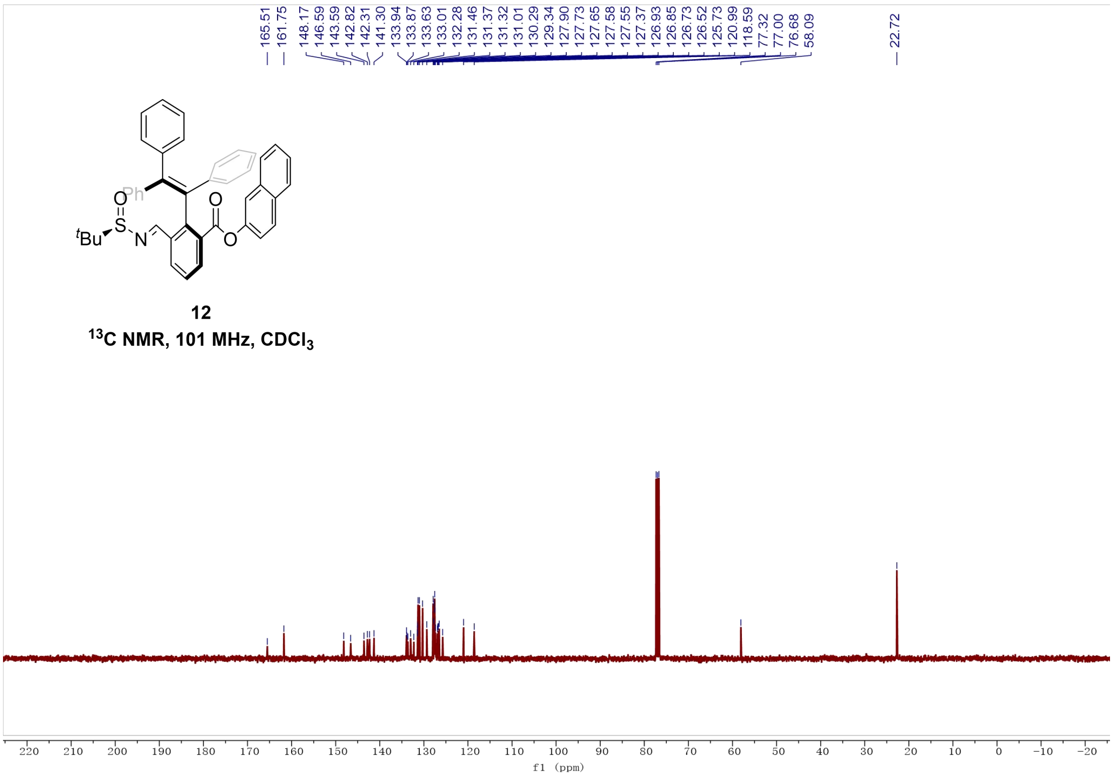
165.51 161.75 148.17 146.59 143.59 142.82 142.31 141.30 133.94 133.87 133.63 133.01 132.28 131.46 131.37 131.32 131.01 130.29 129.34 127.90 127.73 127.65 127.58 127.55 127.37 126.93 126.85 126.73 126.52 125.73 120.99 118.59 77.32 77.00 76.68 58.09
22.72
O
Ph
S
tBu
N
O
O
12
13C NMR, 101 MHz, CDCl3
220 210 200 190 180 170 160 150 140 130 120 110 100 90 80 70 60 50 40 30 20 10 0 -10 -20
f1 (ppm)

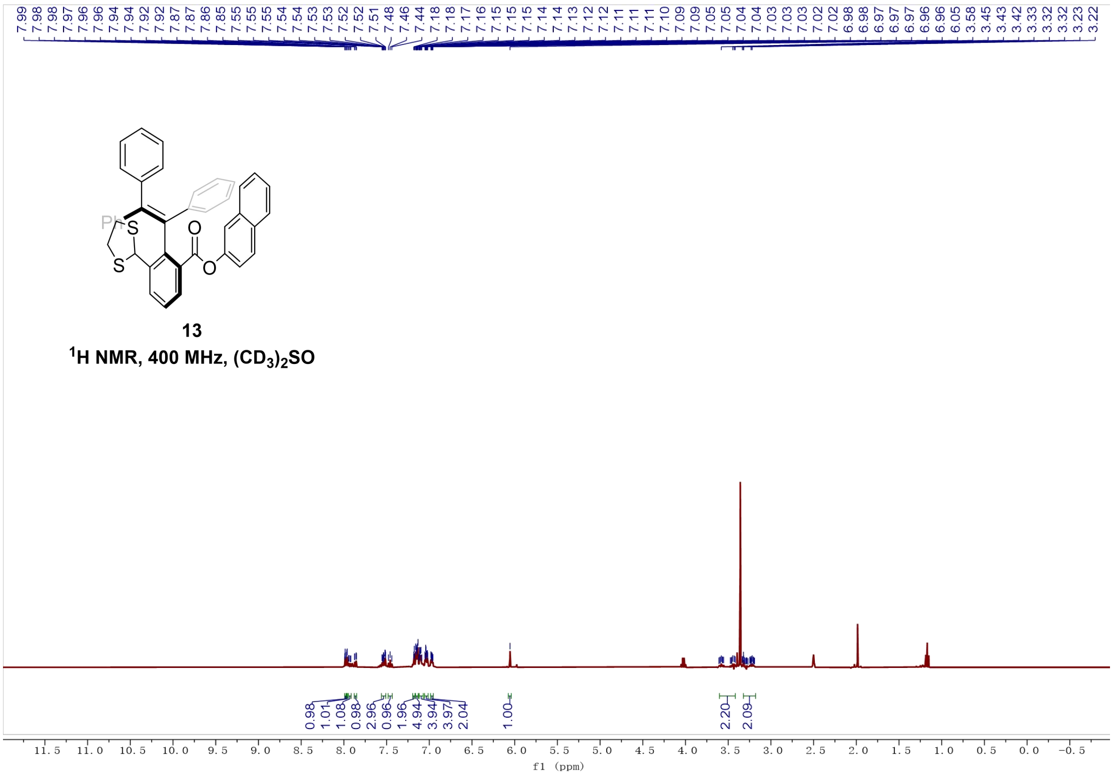
7.99 7.98 7.98 7.97 7.96 7.96 7.94 7.94 7.92 7.92 7.87 7.87 7.86 7.85 7.55 7.55 7.55 7.54 7.54 7.53 7.53 7.52 7.52 7.51 7.48 7.46 7.44 7.18 7.18 7.17 7.16 7.15 7.15 7.14 7.13 7.13 7.12 7.12 7.11 7.11 7.10 7.09 7.09 7.05 7.05 7.04 7.04 7.03 7.03 7.02 7.02 6.98 6.98 6.97 6.97 6.96 6.96 6.05 3.58 3.45 3.43 3.42 3.33 3.32 3.32 3.23 3.22
Ph
S
S
O
O
13
1H NMR, 400 MHz, (CD3)2SO
0.98 1.01 1.08 0.98 2.96 0.96 1.96 4.94 3.94 3.97 2.04 1.00 2.20 2.09
11.5 11.0 10.5 10.0 9.5 9.0 8.5 8.0 7.5 7.0 6.5 6.0 5.5 5.0 4.5 4.0 3.5 3.0 2.5 2.0 1.5 1.0 0.5 0.0 -0.5
f1 (ppm)

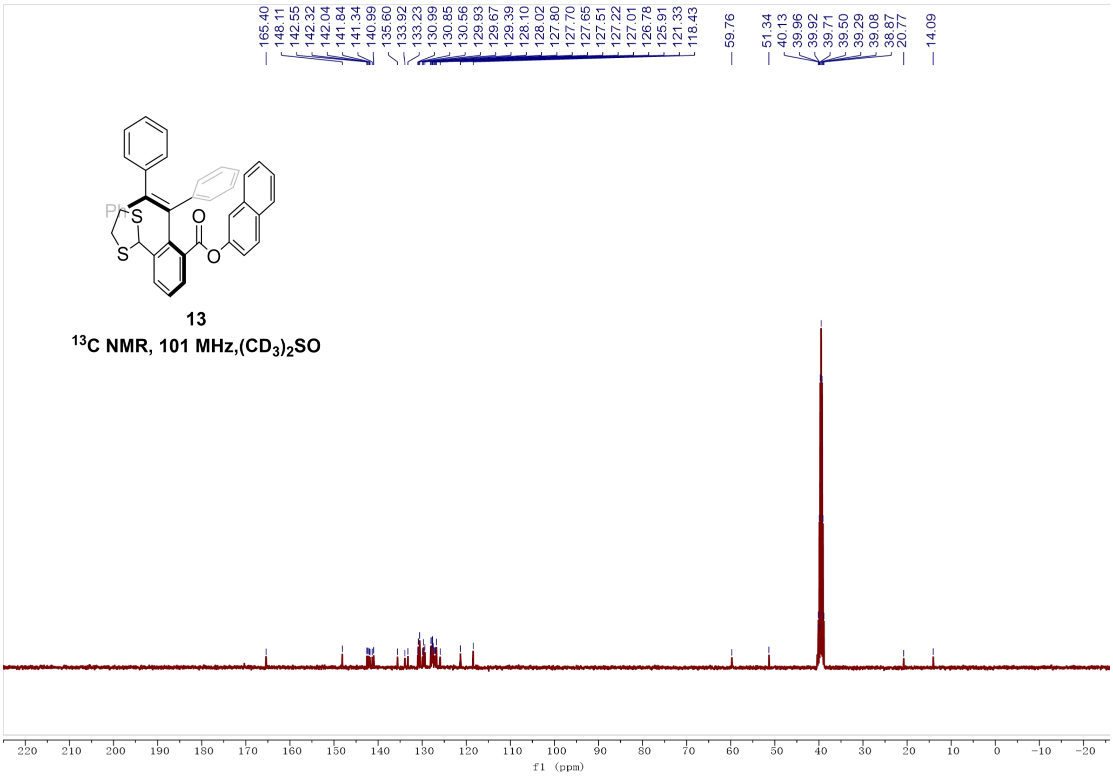
165.40 148.11 142.55 142.32 142.04 141.84 141.34 140.99 135.60 133.92 133.23 130.99 130.85 130.56 129.93 129.67 129.39 128.10 128.02 127.80 127.70 127.65 127.51 127.22 127.01 126.78 125.91 121.33 118.43 59.76 51.34 40.13 39.96 39.92 39.71 39.50 39.29 39.08 38.87 20.77 14.09
Ph
S
S
O
O
13
13C NMR, 101 MHz,(CD3)2SO
220 210 200 190 180 170 160 150 140 130 120 110 100 90 80 70 60 50 40 30 20 10 0 -10 -20
f1 (ppm)

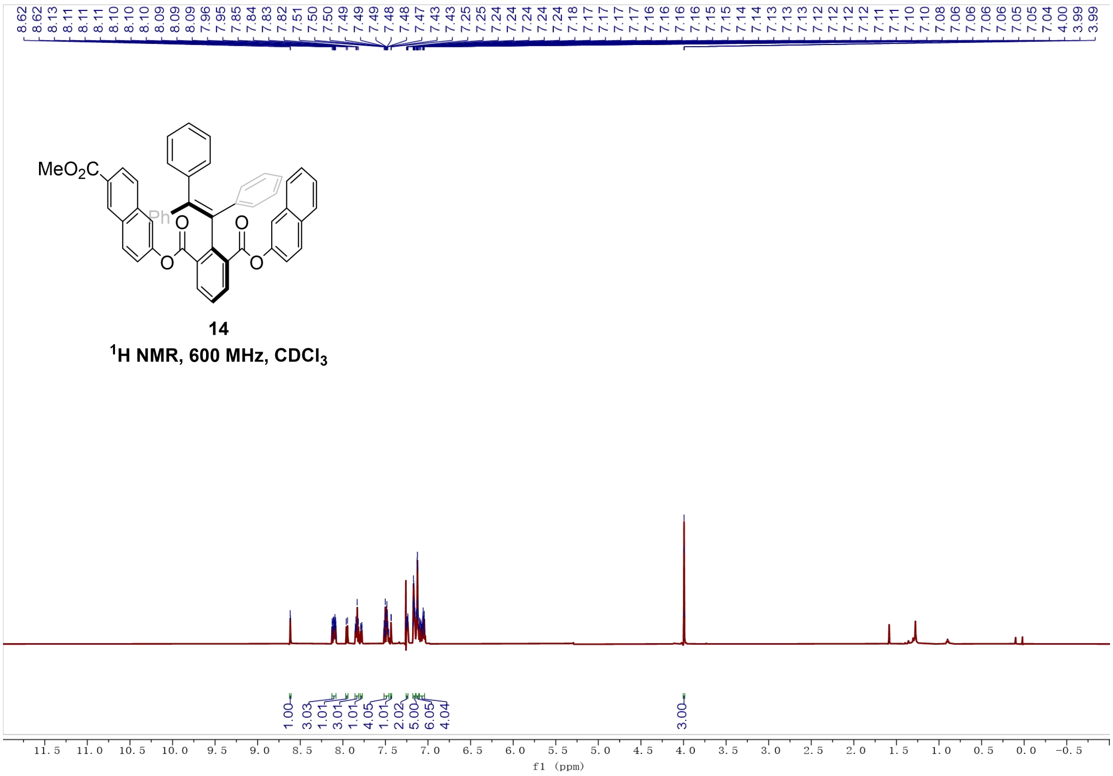

14

^1H NMR, 600 MHz, $CDCl_3$

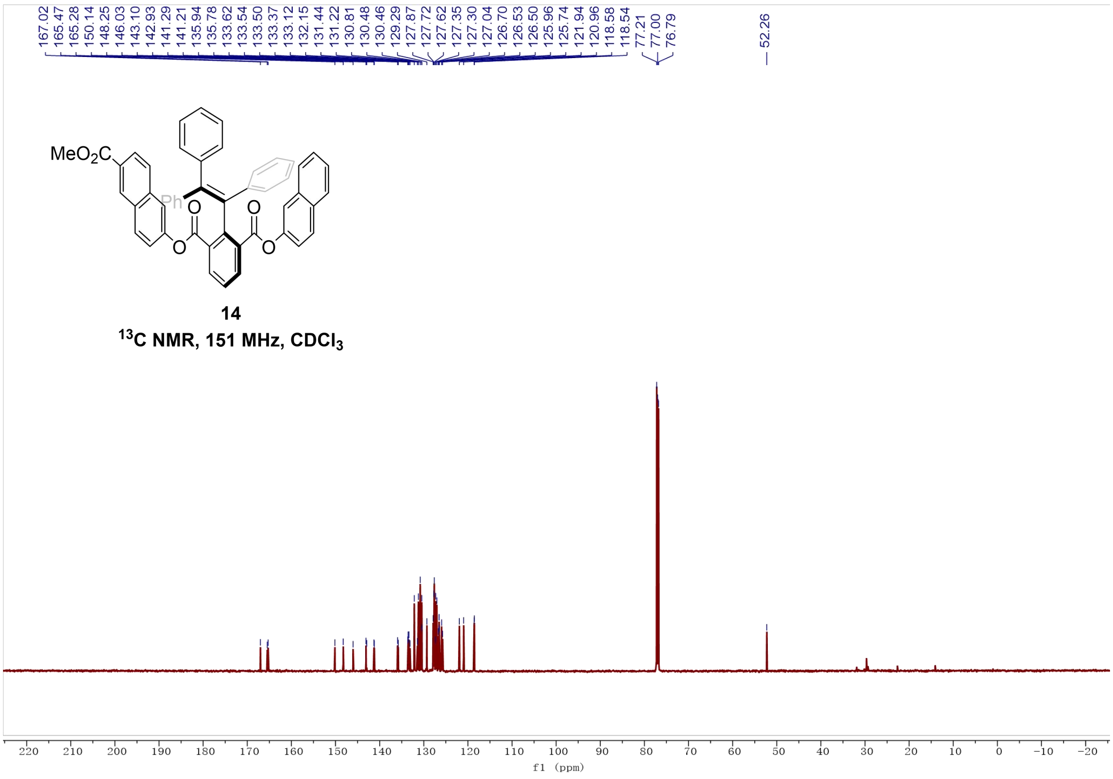

14

^{13}C NMR, 151 MHz, $CDCl_3$

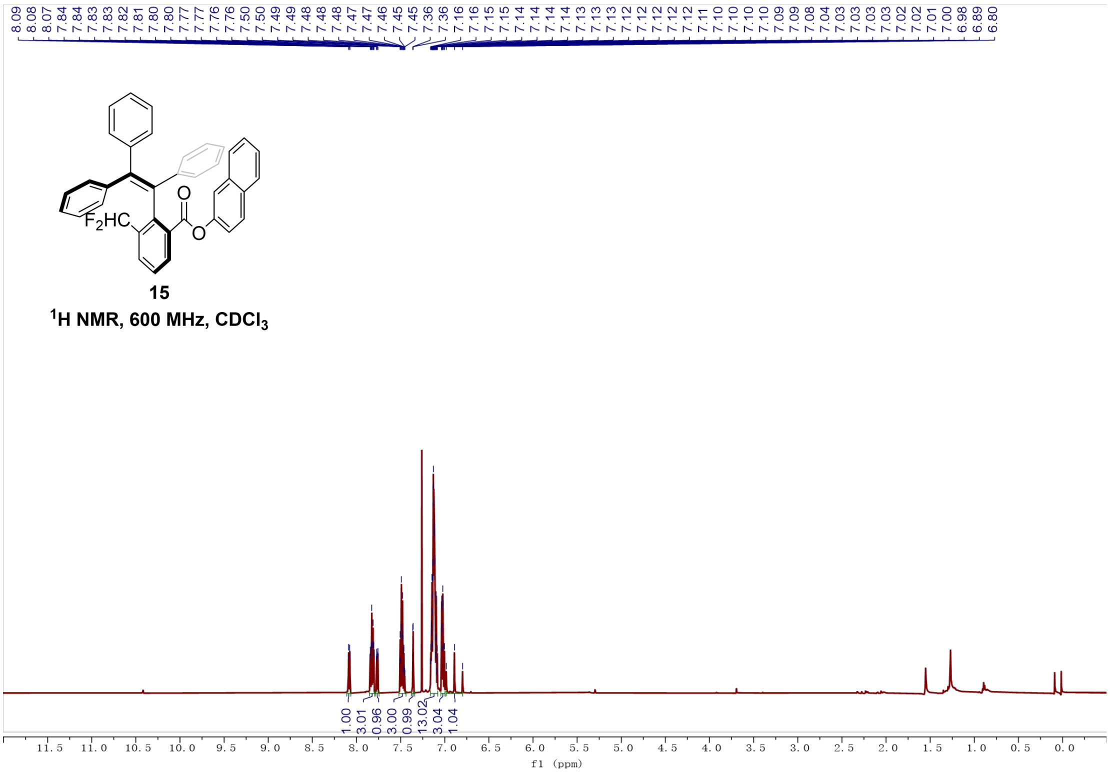

15

^{1}H NMR, 600 MHz, $CDCl_3$

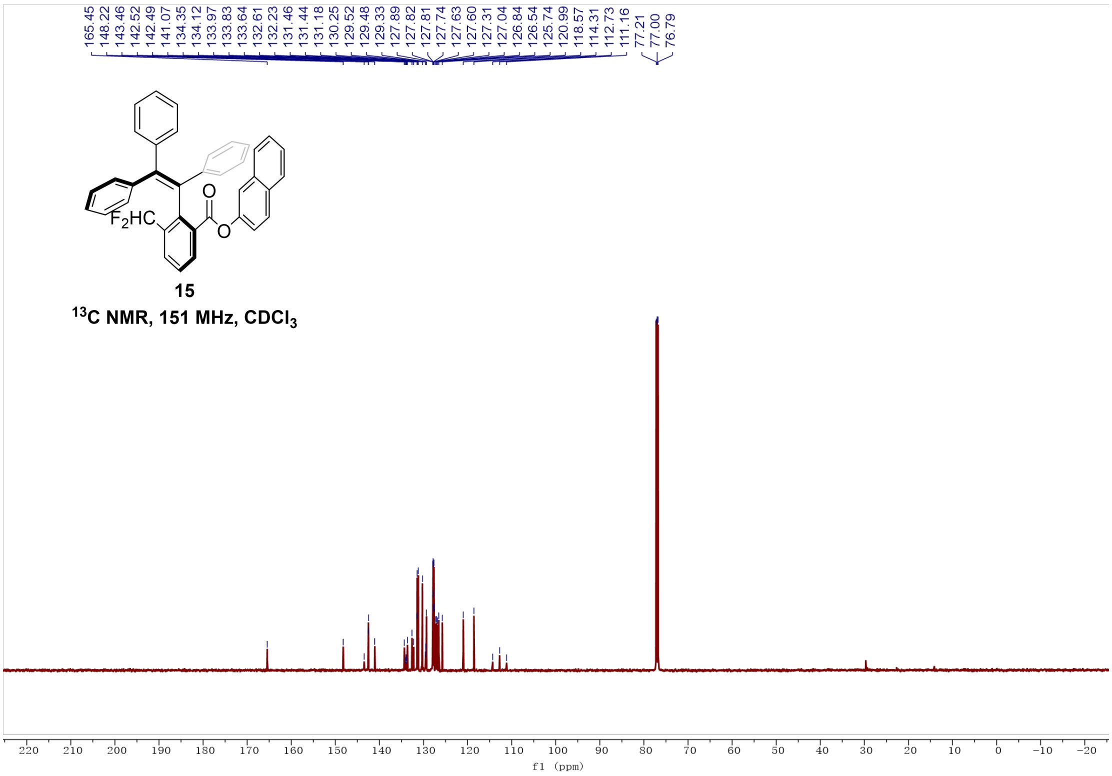

15

^{13}C NMR, 151 MHz, $CDCl_3$

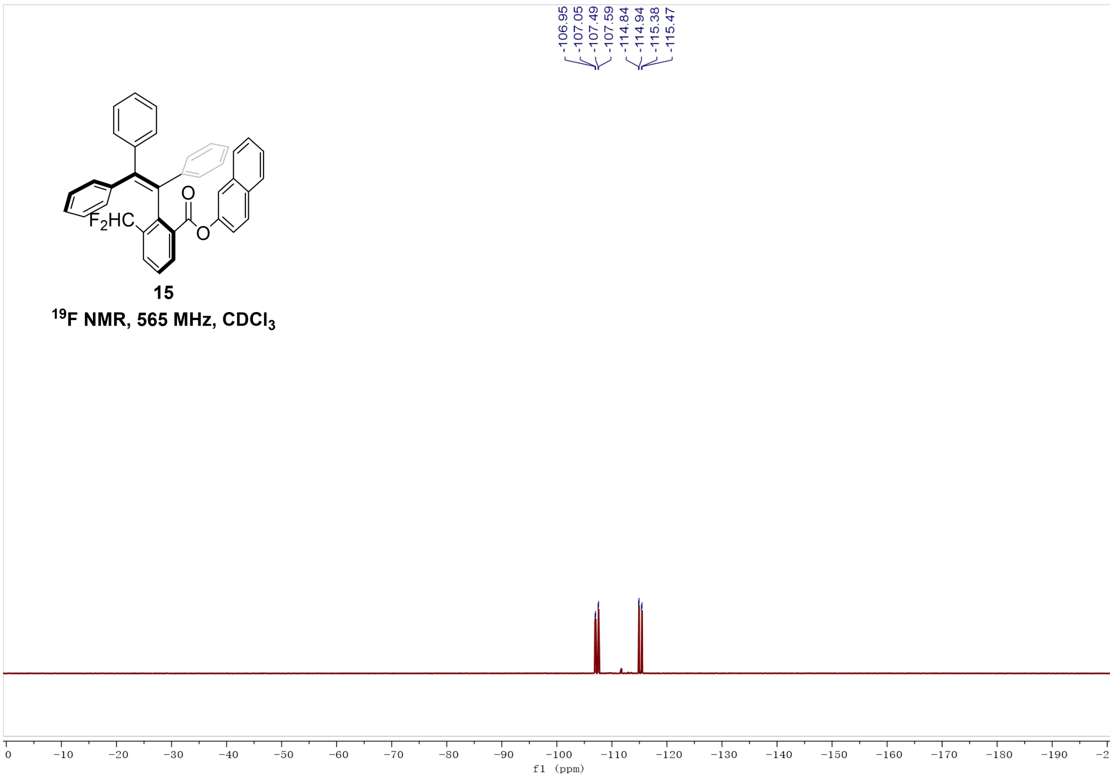

15

^{19}F NMR, 565 MHz, CDCl$_3$

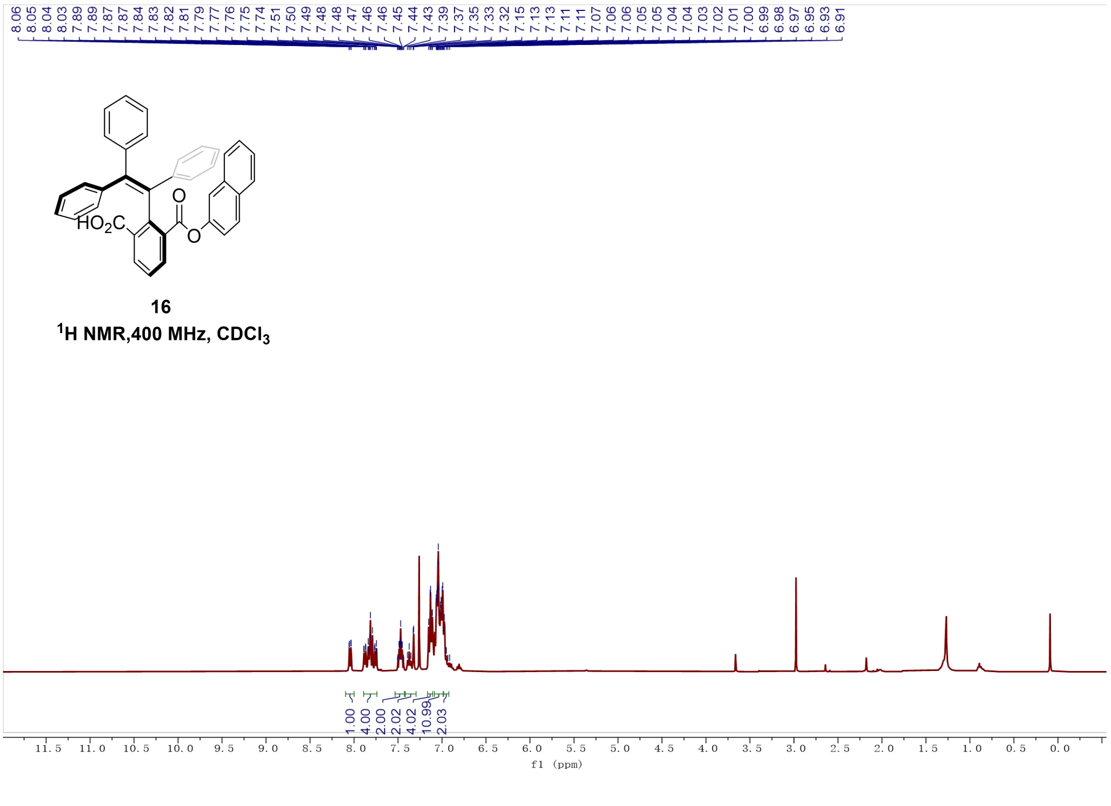

16

^{1}H NMR,400 MHz, CDCl$_3$

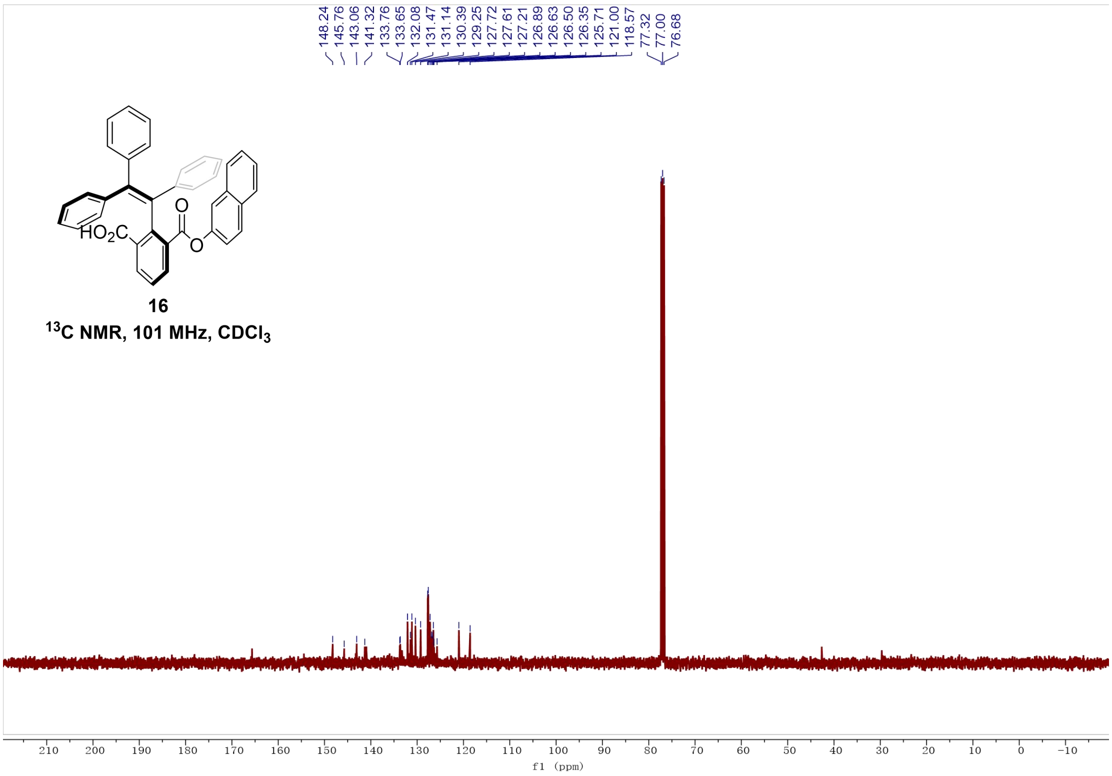
148.24
145.76
143.06
141.32
133.76
133.65
132.08
131.47
131.14
130.39
129.25
127.72
127.61
127.21
126.89
126.63
126.50
126.35
125.71
121.00
118.57
77.32
77.00
76.68
HO2C
O
O
16
13C NMR, 101 MHz, CDCl3
210
200
190
180
170
160
150
140
130
120
110
100
90
80
70
60
50
40
30
20
10
0
-10
f1 (ppm)

8. Copies of HPLC Spectra

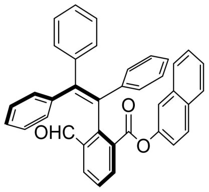

3a

HPLC conditions: Chiralpak OD, 20% ^{i}PrOH/Hx eluent, 0.6 mL/min, 254 nm

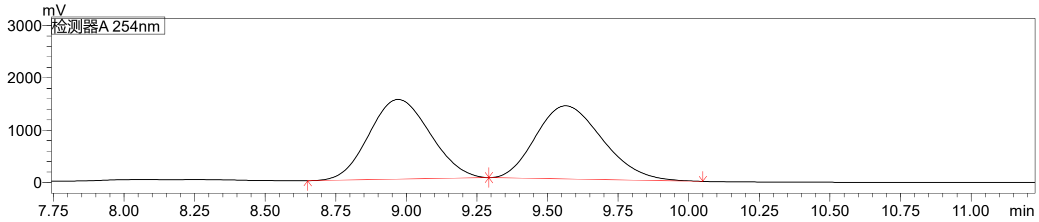

Peak#	Ret. Time	Height	Area%
1	8.970	1523471	49.911
2	9.563	1398257	50.089

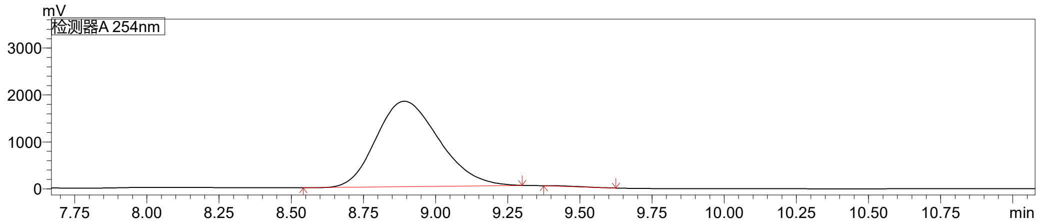

Peak#	Ret. Time	Height	Area%
1	8.892	1821293	99.916
2	9.379	862	0.084

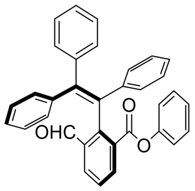

3b

HPLC conditions: Chiralpak IA, 20% ^{i}PrOH/Hx eluent, 1.0 mL/min, 254 nm

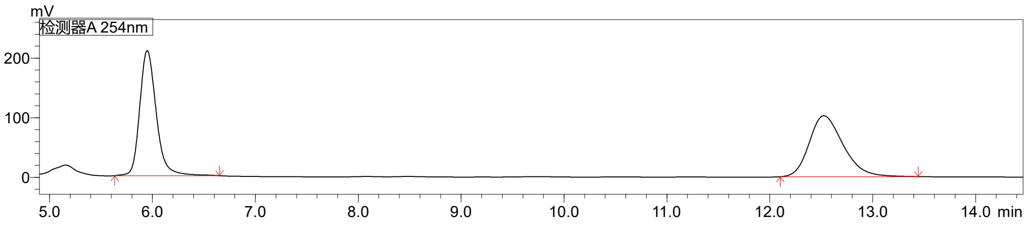

Peak#	Ret. Time	Height	Area%
1	5.949	209756	49.973
2	12.525	102278	50.027

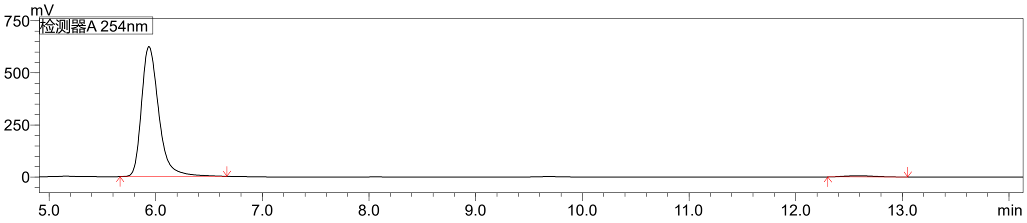

Peak#	Ret. Time	Height	Area%
1	5.937	623291	98.408
2	12.586	5657	1.592

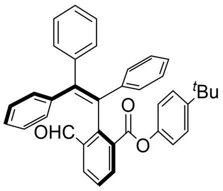

3c

HPLC conditions: Chiralpak IA, 20% ^{i}PrOH/Hx eluent, 1.0 mL/min, 254 nm

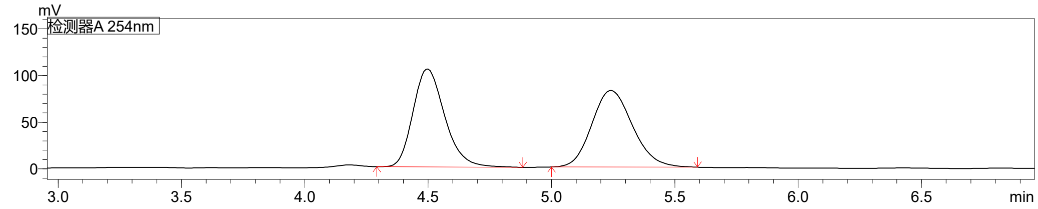

Peak#	Ret. Time	Height	Area%
1	4.497	104855	49.872
2	5.241	82155	50.128

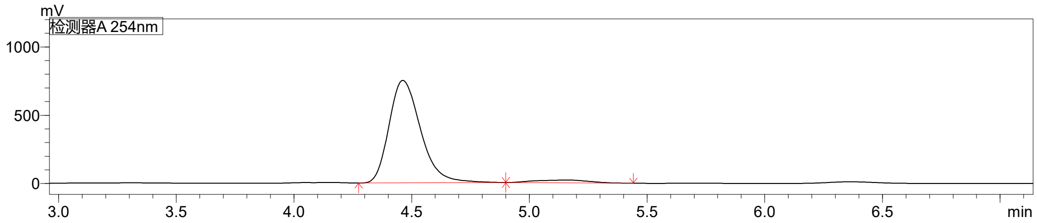

Peak#	Ret. Time	Height	Area%
1	4.463	749576	95.569
2	5.158	20778	4.431

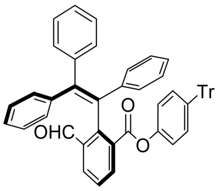

3d

HPLC conditions: Chiralpak OD, 20% ^{i}PrOH/Hx eluent, 0.6 mL/min, 254 nm

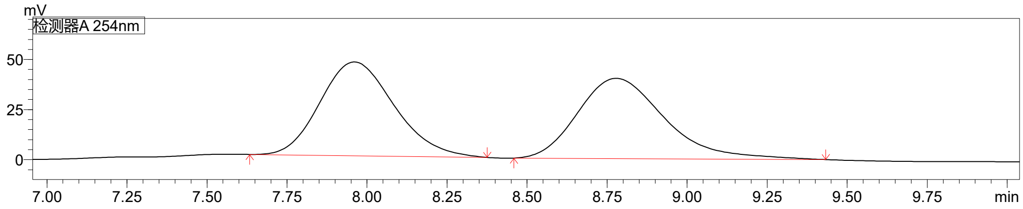

Peak#	Ret. Time	Height	Area%
1	7.960	46832	49.975
2	8.778	40092	50.025

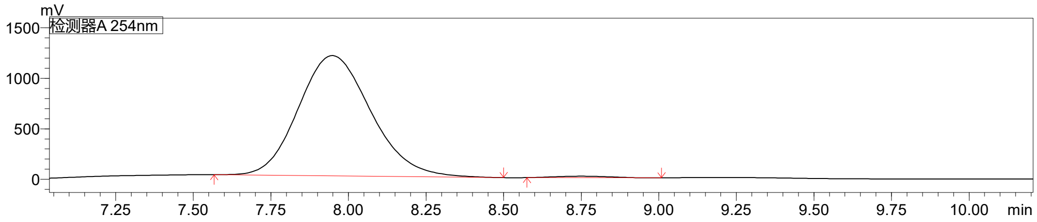

Peak#	Ret. Time	Height	Area%
1	7.948	1191062	99.051
2	8.750	15028	0.949

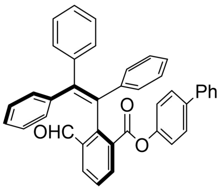

3e

HPLC conditions: Chiralpak IA, 20% ^{i}PrOH/Hx eluent, 1.0 mL/min, 254 nm

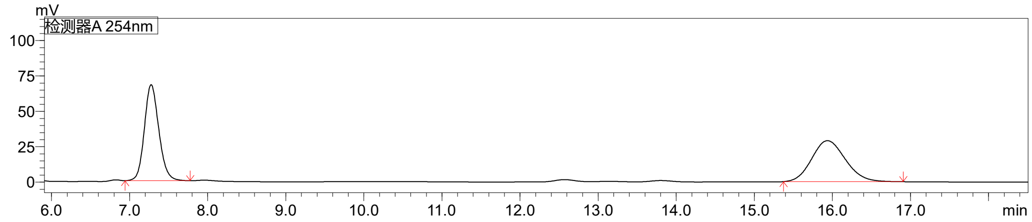

Peak#	Ret. Time	Height	Area%
1	7.275	67922	50.132
2	15.937	28950	49.868

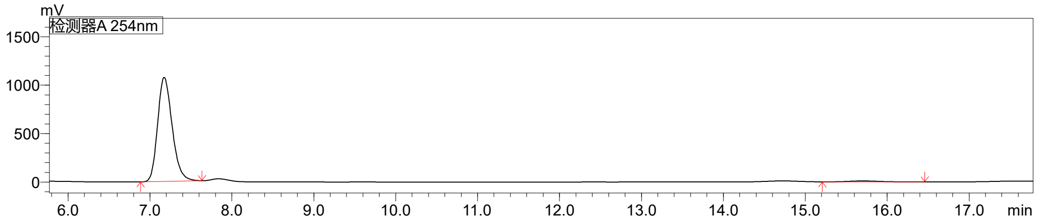

Peak#	Ret. Time	Height	Area%
1	7.173	1075569	98.140
2	15.703	9437	1.860

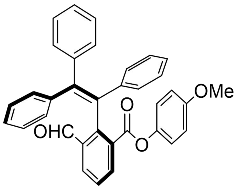

3f

HPLC conditions: Chiralpak OD, 20% ^{i}PrOH/Hx eluent, 0.6 mL/min, 254 nm

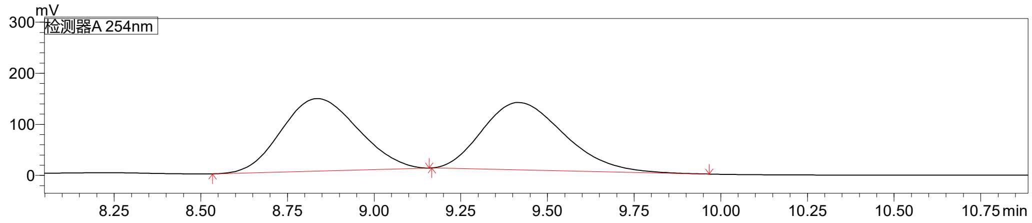

Peak#	Ret. Time	Height	Area%
1	8.836	141982	50.172
2	9.416	132018	49.828

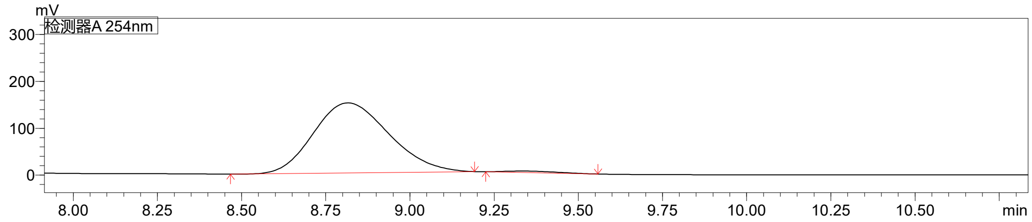

Peak#	Ret. Time	Height	Area%
1	8.816	149281	98.612
2	9.332	3035	1.388

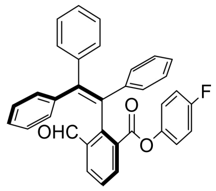

3g

HPLC conditions: Chiralpak IA, 20% ^{i}PrOH/Hx eluent, 1.0 mL/min, 254 nm

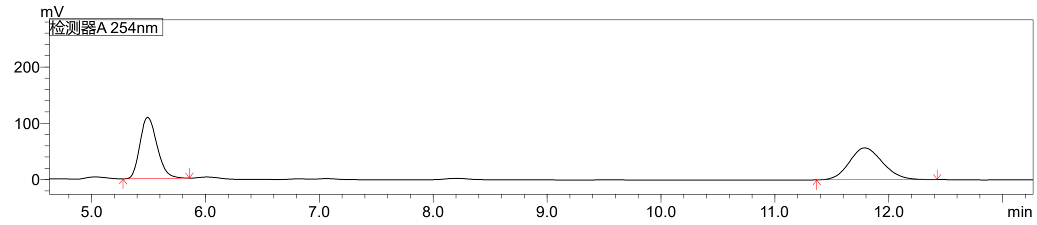

Peak#	Ret. Time	Height	Area%
1	5.492	108902	49.818
2	11.790	56617	50.182

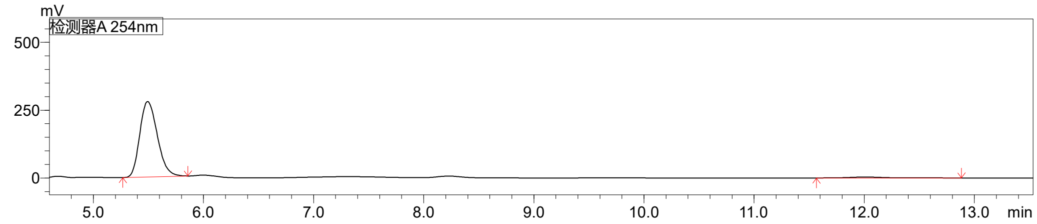

Peak#	Ret. Time	Height	Area%
1	5.493	278527	97.835
2	12.008	3204	2.165

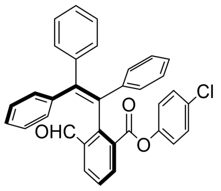

3h

HPLC conditions: Chiralpak OD, 20% ⁱPrOH/Hx eluent, 0.8 mL/min, 254 nm

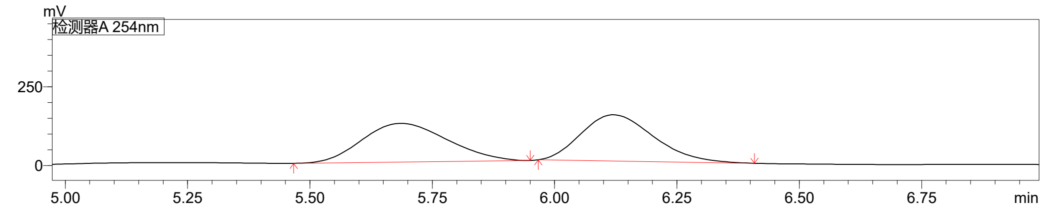

Peak#	Ret. Time	Height	Area%
1	5.686	122775	49.654
2	6.120	146709	50.346

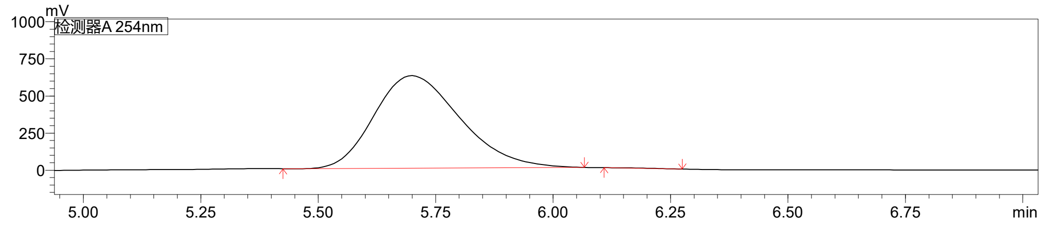

Peak#	Ret. Time	Height	Area%
1	5.699	623661	99.915
2	6.113	239	0.085

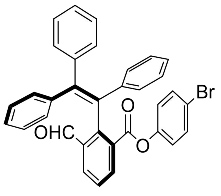

3i

HPLC conditions: Chiralpak IA, 20% ^{i}PrOH/Hx eluent, 1.0 mL/min, 254 nm

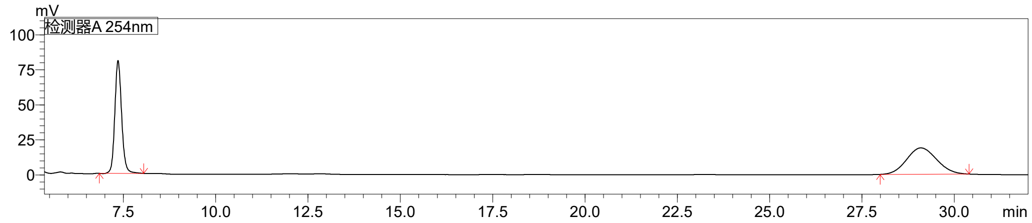

Peak#	Ret. Time	Height	Area%
1	7.354	80550	49.251
2	29.097	18823	50.749

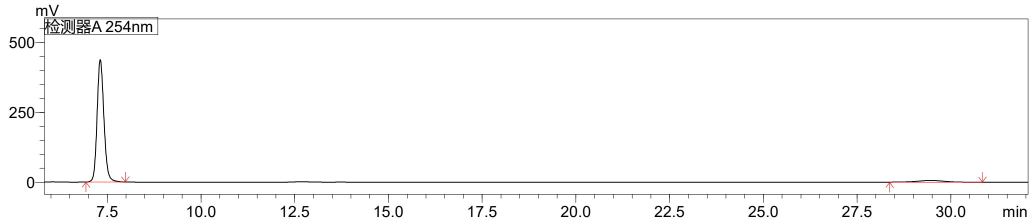

Peak#	Ret. Time	Height	Area%
1	7.314	438057	94.735
2	29.463	6243	5.265

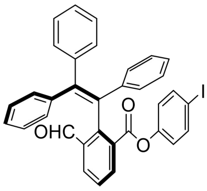

3j

HPLC conditions: Chiralpak OD, 20% ^{i}PrOH/Hx eluent, 0.6 mL/min, 254 nm

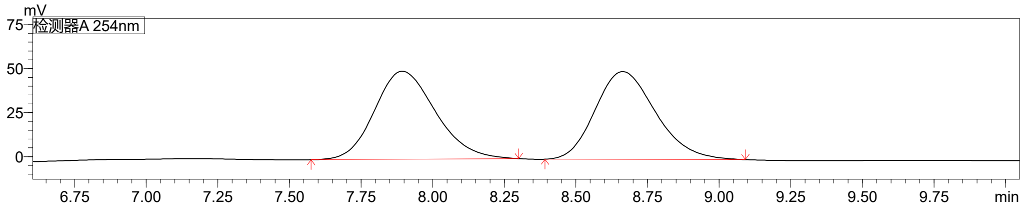

Peak#	Ret. Time	Height	Area%
1	7.894	50016	50.260
2	8.663	49889	49.740

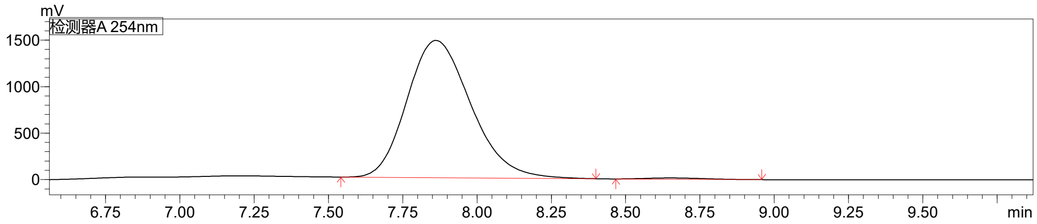

Peak#	Ret. Time	Height	Area%
1	7.862	1476252	99.186
2	8.651	13853	0.814

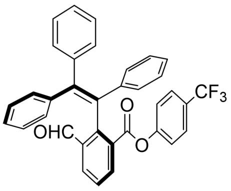

3k

HPLC conditions: Chiralpak IA, 20% ^{i}PrOH/Hx eluent, 1.0 mL/min, 254 nm

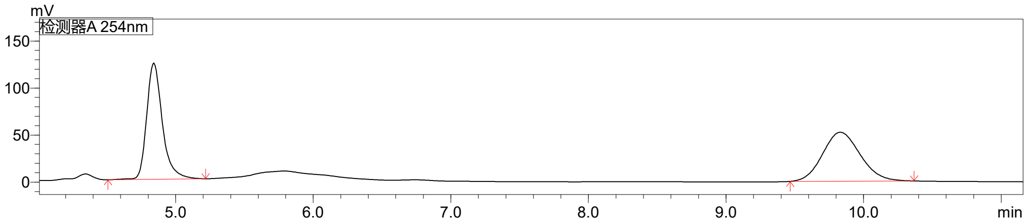

Peak#	Ret. Time	Height	Area%
1	4.840	123681	49.882
2	9.831	52000	50.118

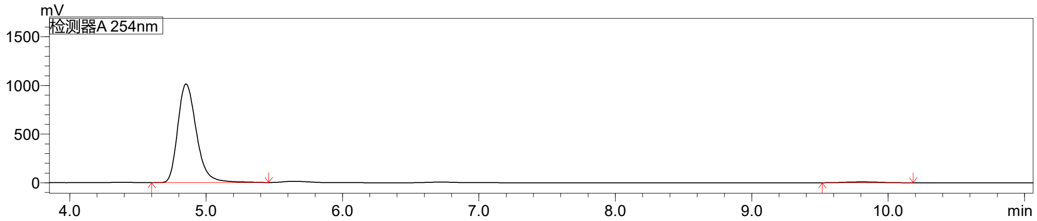

Peak#	Ret. Time	Height	Area%
1	4.853	1014301	98.524
2	9.806	8256	1.476

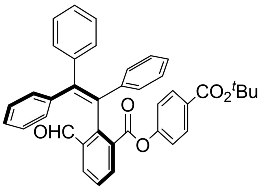

3l

HPLC conditions: Chiralpak IA, 20% ^{i}PrOH/Hx eluent, 1.0 mL/min, 254 nm

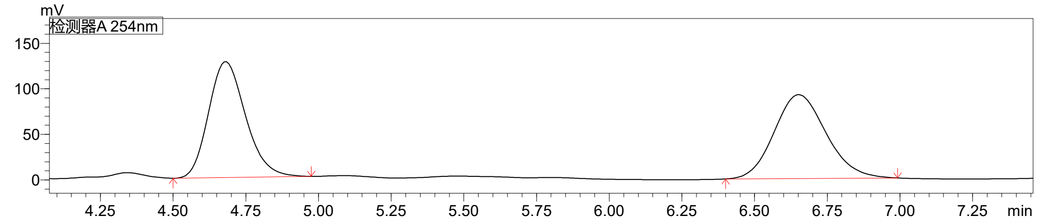

Peak#	Ret. Time	Height	Area%
1	4.680	126976	49.573
2	6.651	92054	50.427

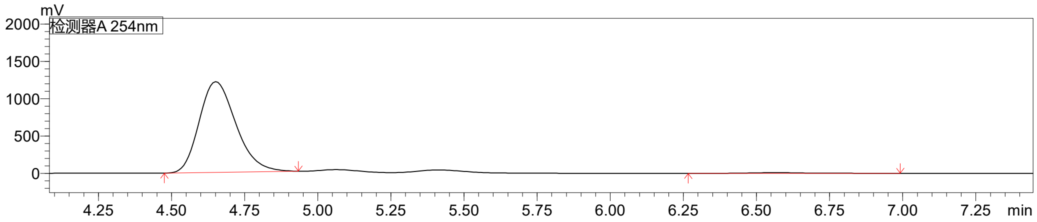

Peak#	Ret. Time	Height	Area%
1	4.651	1215491	98.997
2	6.562	8717	1.003

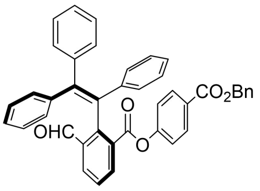

3m

HPLC conditions: Chiralpak IA, 20% ^{i}PrOH/Hx eluent, 1.0 mL/min, 254 nm

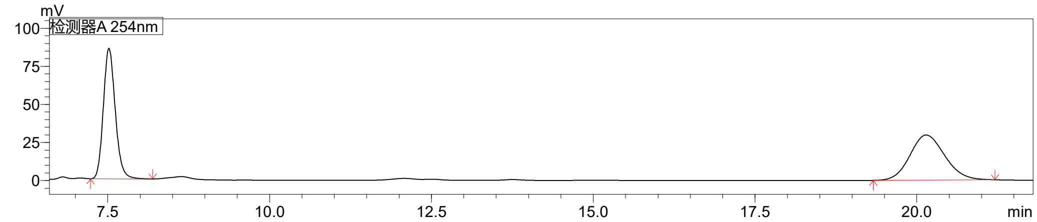

Peak#	Ret. Time	Height	Area%
1	7.517	85710	49.752
2	20.145	29614	50.248

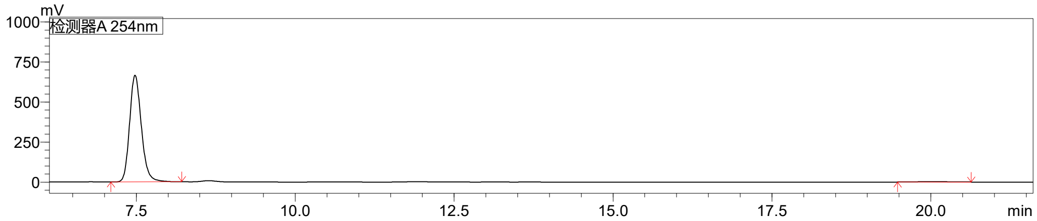

Peak#	Ret. Time	Height	Area%
1	7.480	665902	99.037
2	20.019	2474	0.963

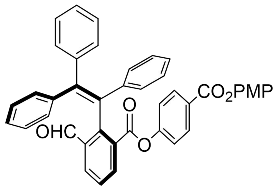

3n

HPLC conditions: Chiralpak OD 20% ^{i}PrOH/Hx eluent, 0.8 mL/min, 254 nm

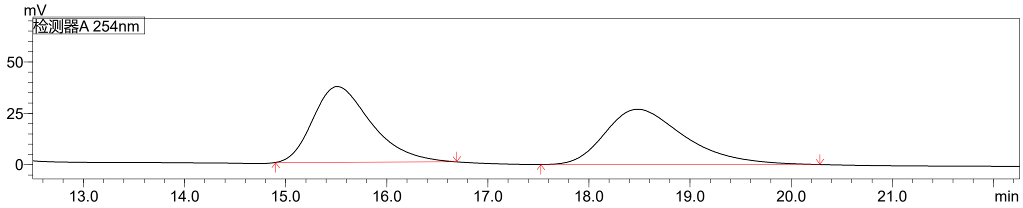

Peak#	Ret. Time	Height	Area%
1	15.511	36815	50.051
2	18.481	26870	49.949

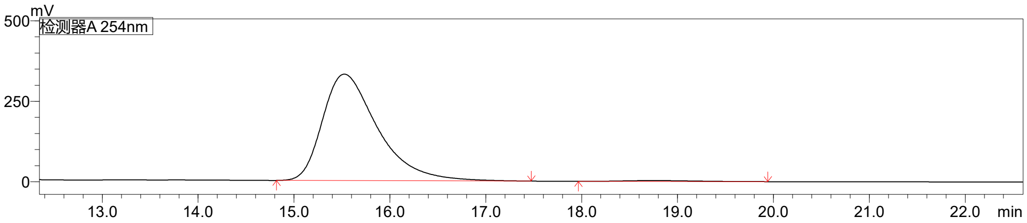

Peak#	Ret. Time	Height	Area%
1	15.524	330261	98.879
2	18.746	2888	1.121

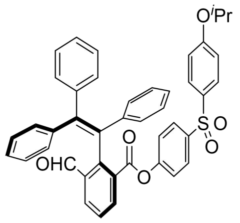

3o

HPLC conditions: Chiralpak IA 20% ^{i}PrOH/Hx eluent, 1.0 mL/min, 254 nm

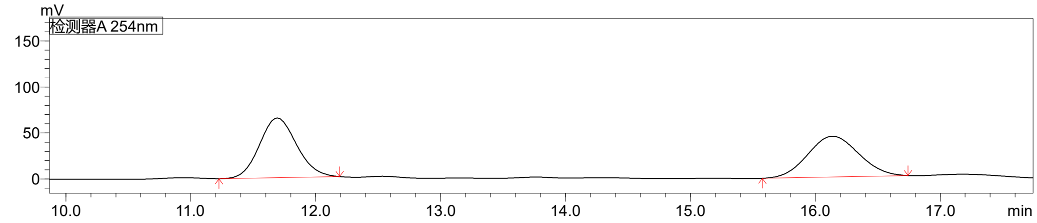

Peak#	Ret. Time	Height	Area%
1	11.692	64790	51.571
2	16.138	44382	48.429

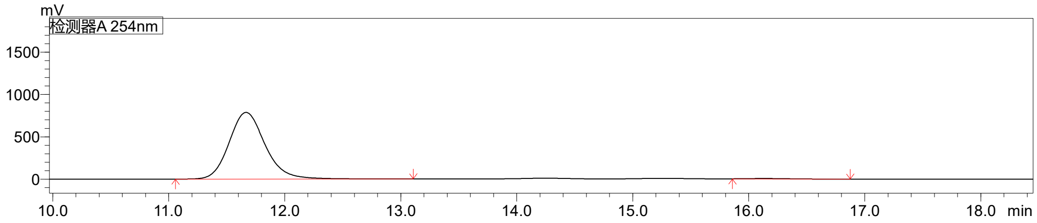

Peak#	Ret. Time	Height	Area%
1	11.665	788318	99.451
2	16.126	4515	0.549

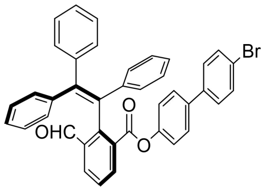

3p

HPLC conditions: Chiralpak OD 20% ^{i}PrOH/Hx eluent, 0.6 mL/min, 254 nm

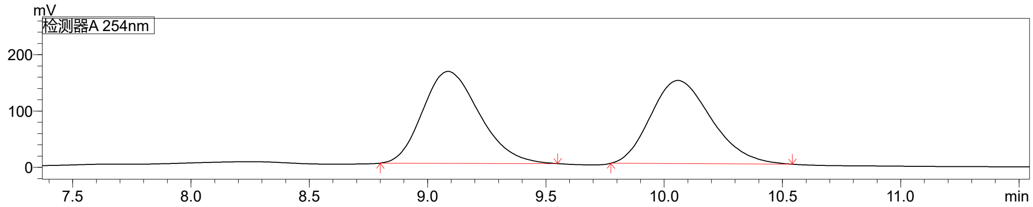

Peak#	Ret. Time	Height	Area%
1	9.087	163234	50.754
2	10.058	147891	49.246

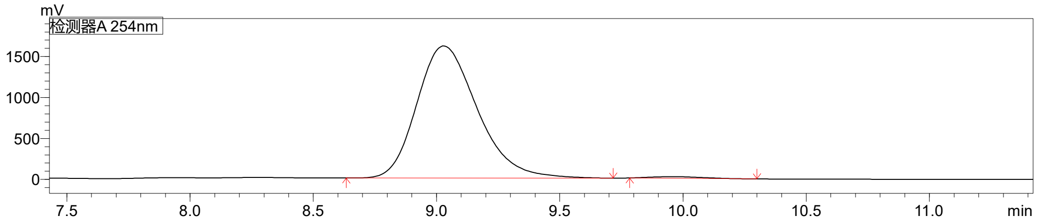

Peak#	Ret. Time	Height	Area%
1	9.029	1612500	98.966
2	9.954	19850	1.034

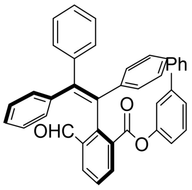

3q

HPLC conditions: Chiralpak IA 20% ^{i}PrOH/Hx eluent, 1.0 mL/min, 254 nm

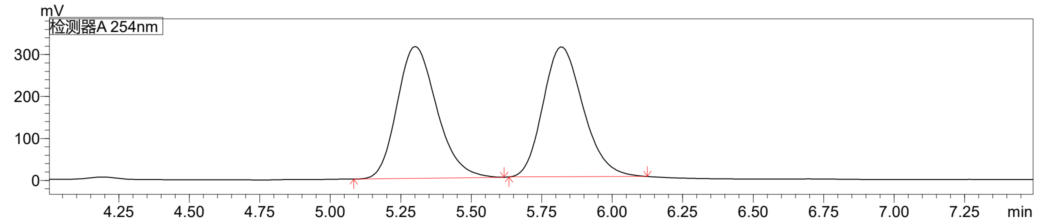

Peak#	Ret. Time	Height	Area%
1	5.301	314660	50.048
2	5.820	309871	49.952

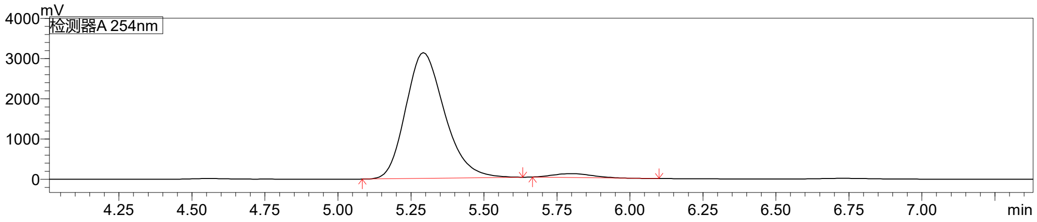

Peak#	Ret. Time	Height	Area%
1	5.292	3131027	97.114
2	5.798	95465	2.886

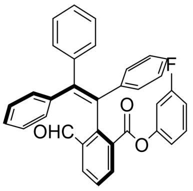

3r

HPLC conditions: Chiralpak IA 20% ^{i}PrOH/Hx eluent, 1.0 mL/min, 254 nm

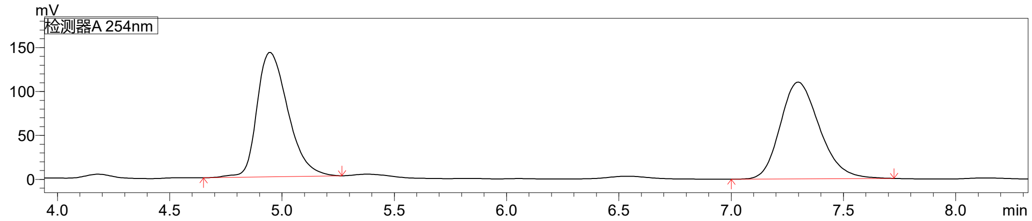

Peak#	Ret. Time	Height	Area%
1	4.946	141401	50.346
2	7.298	110188	49.654

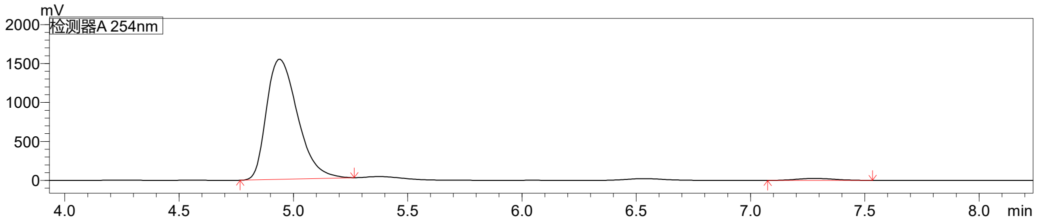

Peak#	Ret. Time	Height	Area%
1	4.939	1543002	98.004
2	7.279	25626	1.996

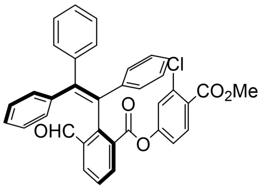

3s

HPLC conditions: Chiralpak IA 20% ⁱPrOH/Hx eluent, 1.0 mL/min, 254 nm

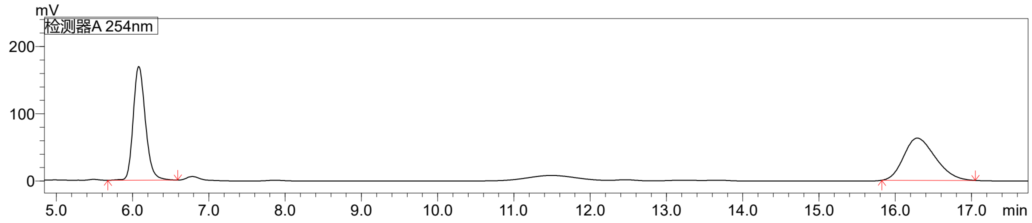

Peak#	Ret. Time	Height	Area%
1	6.080	169100	50.994
2	16.288	62661	49.006

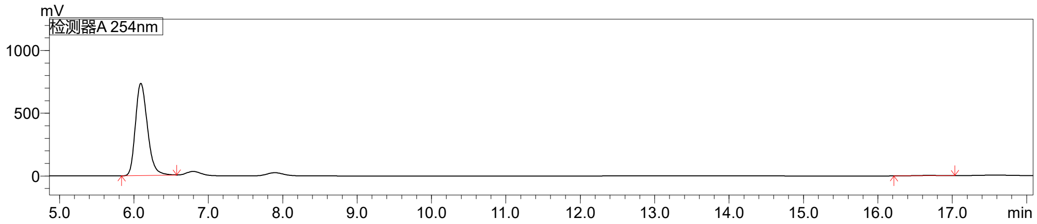

Peak#	Ret. Time	Height	Area%
1	6.092	734890	99.163
2	16.686	2937	0.837

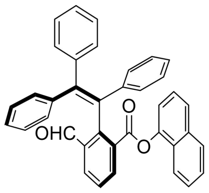

3t

HPLC conditions: Chiralpak OD 5% ^{i}PrOH/Hx eluent, 0.8 mL/min, 254 nm

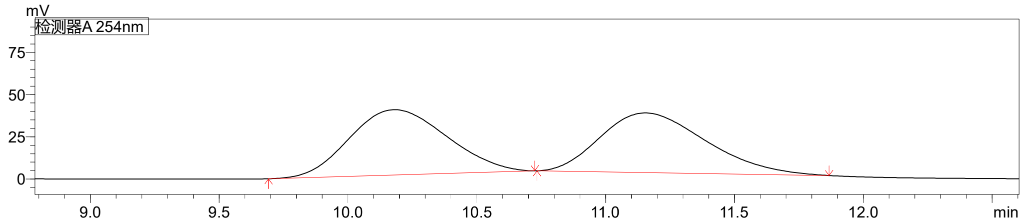

Peak#	Ret. Time	Height	Area%
1	10.181	38696	50.323
2	11.153	35290	49.677

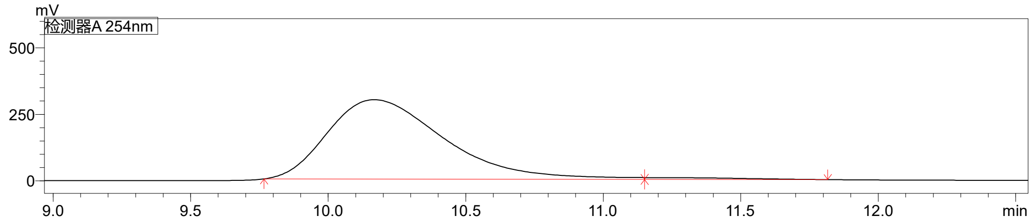

Peak#	Ret. Time	Height	Area%
1	10.168	298416	98.256
2	11.163	6781	1.744

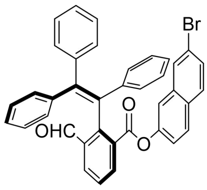

3u

HPLC conditions: Chiralpak OD 20% ^iPrOH/Hx eluent, 0.6 mL/min, 254 nm

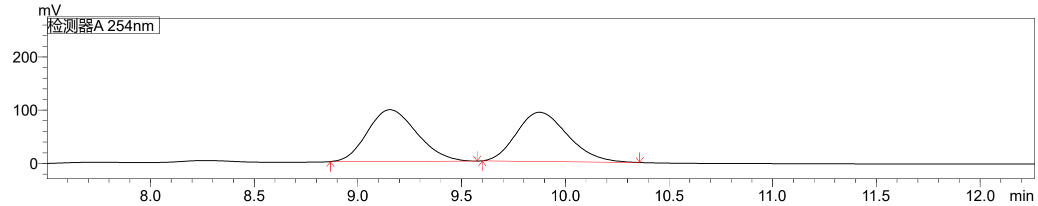

Peak#	Ret. Time	Height	Area%
1	9.154	97030	50.365
2	9.876	92467	49.635

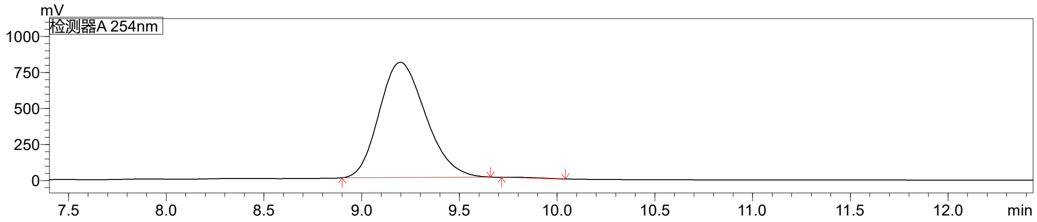

Peak#	Ret. Time	Height	Area%
1	9.197	800170	99.785
2	9.763	1761	0.215

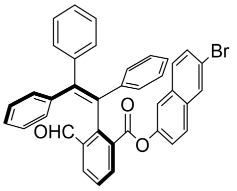

3v

HPLC conditions: Chiralpak OD 20% ^{i}PrOH/Hx eluent, 0.6 mL/min, 254 nm

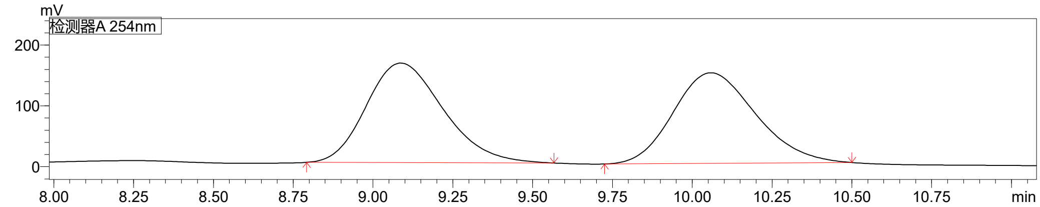

Peak#	Ret. Time	Height	Area%
1	9.087	163634	50.652
2	10.058	148923	49.348

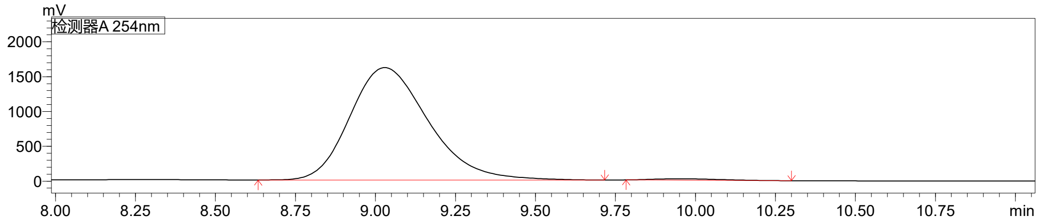

Peak#	Ret. Time	Height	Area%
1	9.029	1612500	98.966
2	9.954	19850	1.034

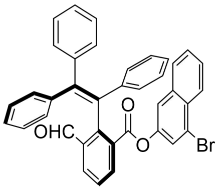

3w

HPLC conditions: Chiralpak OD 20% ^{i}PrOH/Hx eluent, 0.6 mL/min, 254 nm

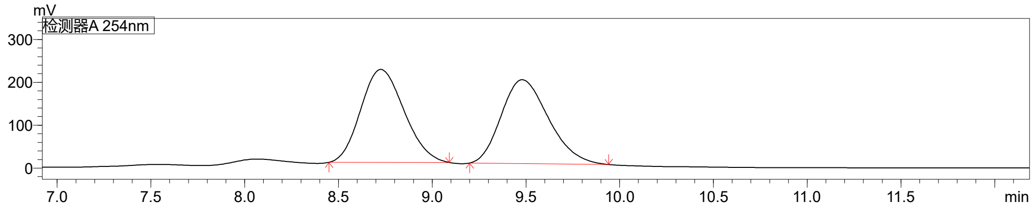

Peak#	Ret. Time	Height	Area%
1	8.726	216911	50.381
2	9.481	195466	49.619

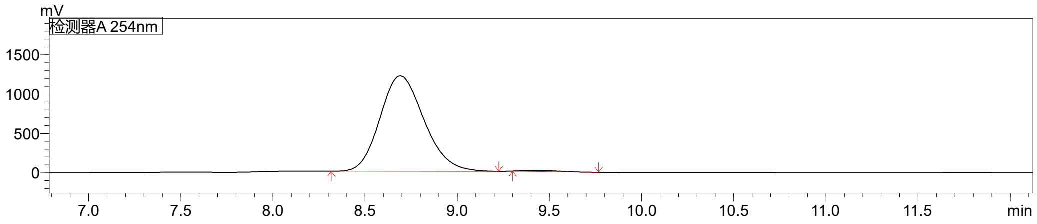

Peak#	Ret. Time	Height	Area%
1	8.690	1214709	99.196
2	9.423	12769	0.804

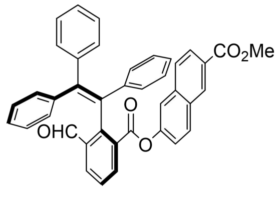

3x

HPLC conditions: Chiralpak OD 20% ^{i}PrOH/Hx eluent, 0.6 mL/min, 254 nm

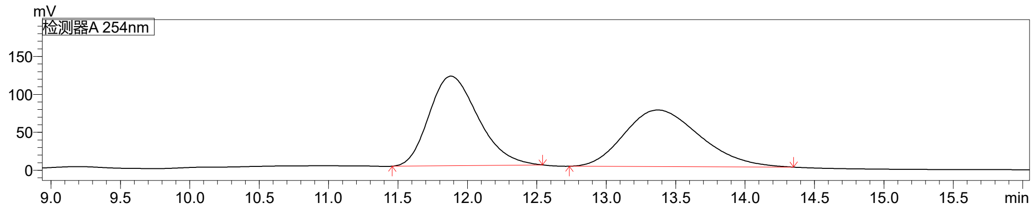

Peak#	Ret. Time	Height	Area%
1	11.880	118039	50.598
2	13.371	74670	49.402

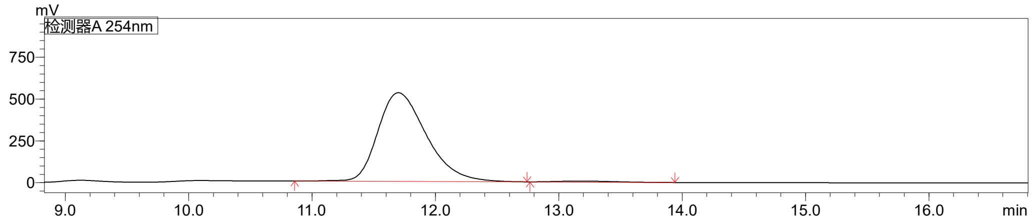

Peak#	Ret. Time	Height	Area%
1	11.699	530161	98.783
2	13.172	5717	1.217

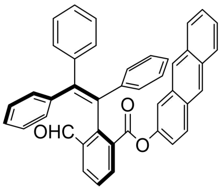

3y

HPLC conditions: Chiralpak OD 20% ^{i}PrOH/Hx eluent, 0.6 mL/min, 254 nm

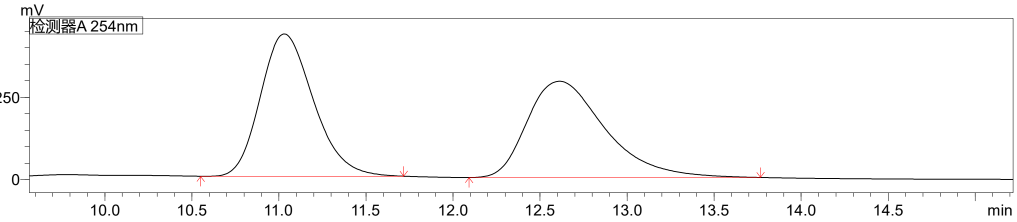

Peak#	Ret. Time	Height	Area%
1	11.031	431668	50.147
2	12.611	292177	49.853

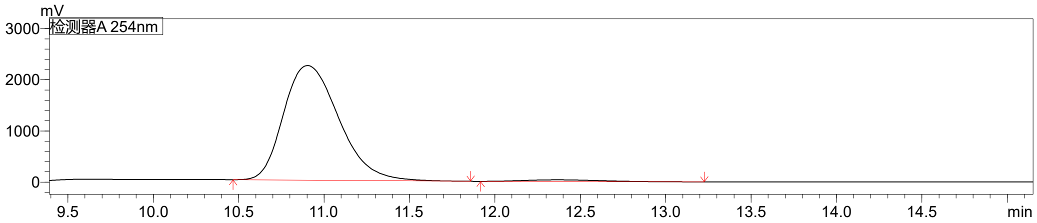

Peak#	Ret. Time	Height	Area%
1	10.903	2241007	98.017
2	12.364	32942	1.983

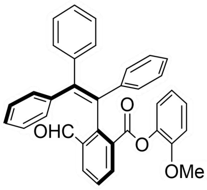

4a

HPLC conditions: Chiralpak OD 20% ^{i}PrOH/Hx eluent, 0.8 mL/min, 254 nm

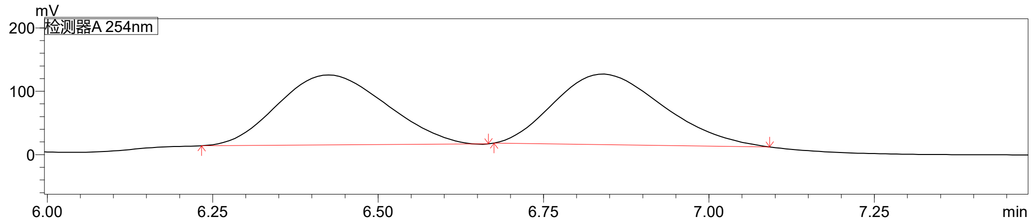

Peak#	Ret. Time	Height	Area%
1	6.424	110525	49.535
2	6.839	111289	50.465

数据文件名:ZYZ-97-2S-OD8020-254-0.8.lcd
样品名:ZYZ-97-2S-OD8020-254-0.8

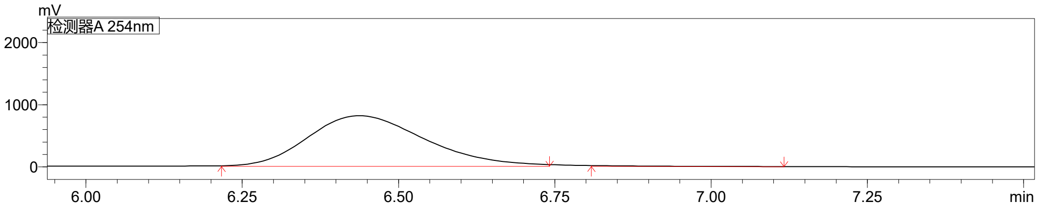

Peak#	Ret. Time	Height	Area%
1	6.437	816525	98.890
2	6.821	14035	1.110

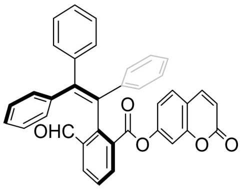

4b

HPLC conditions: Chiralpak OD 20% ^{i}PrOH/Hx eluent, 0.8 mL/min, 254 nm

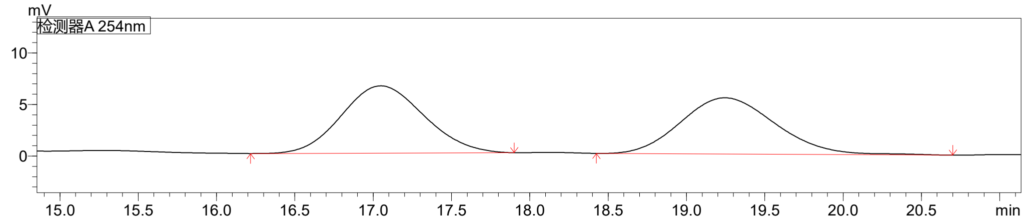

Peak#	Ret. Time	Height	Area%
1	17.047	6527	51.048
2	19.246	5452	48.952

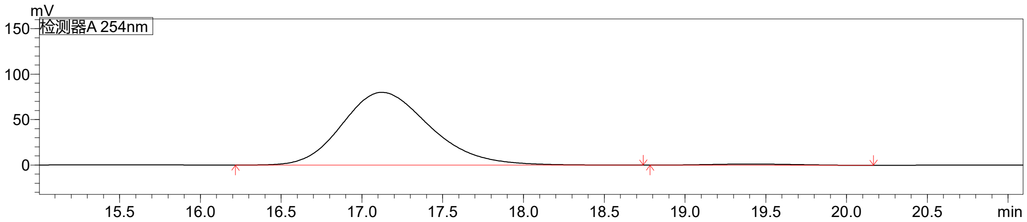

Peak#	Ret. Time	Height	Area%
1	17.124	80069	98.338
2	19.404	1344	1.662

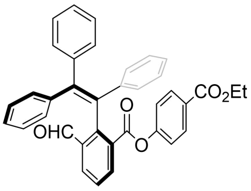

4c

HPLC conditions: Chiralpak OD 20% ^{i}PrOH/Hx eluent, 0.8 mL/min, 254 nm

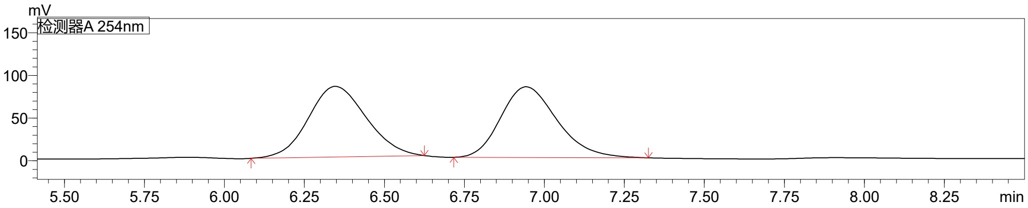

Peak#	Ret. Time	Height	Area%
1	6.347	82743	50.697
2	6.943	82891	49.303

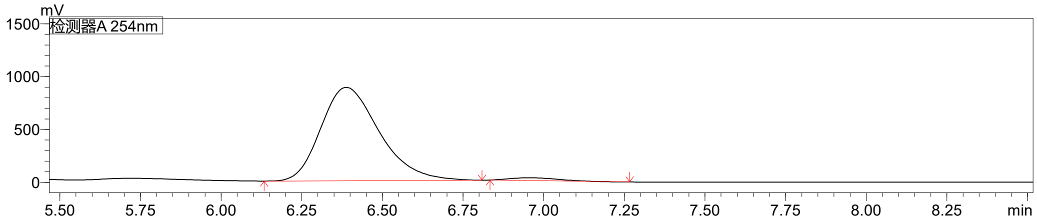

Peak#	Ret. Time	Height	Area%
1	6.388	883745	97.685
2	6.956	26635	2.315

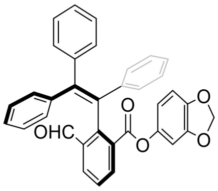

4d

HPLC conditions: Chiralpak OD 10% ^{i}PrOH/Hx eluent, 0.8 mL/min, 254 nm

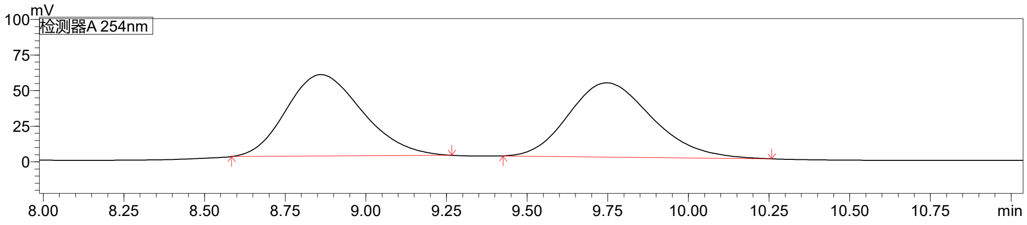

Peak#	Ret. Time	Height	Area%
1	8.860	57189	50.017
2	9.746	52100	49.983

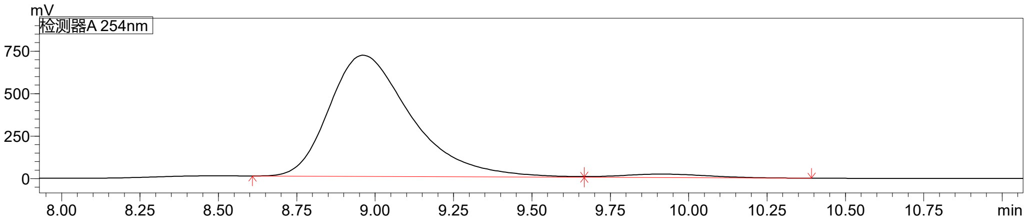

Peak#	Ret. Time	Height	Area%
1	8.961	713292	97.056
2	9.915	20768	2.944

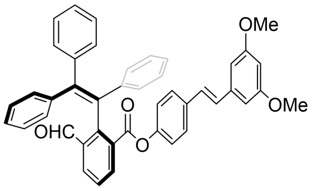

4e

HPLC conditions: Chiralpak OD 20% ^{i}PrOH/Hx eluent, 0.8 mL/min, 254 nm

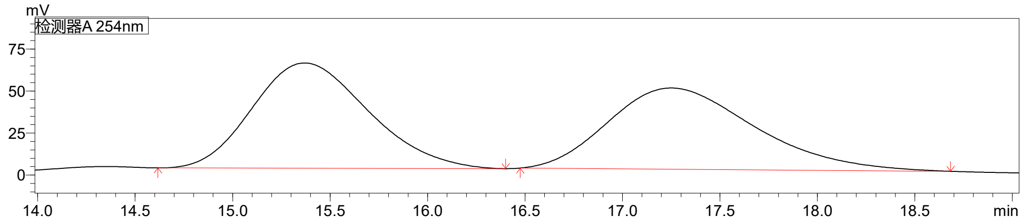

Peak#	Ret. Time	Height	Area%
1	15.369	62664	50.677
2	17.250	48340	49.323

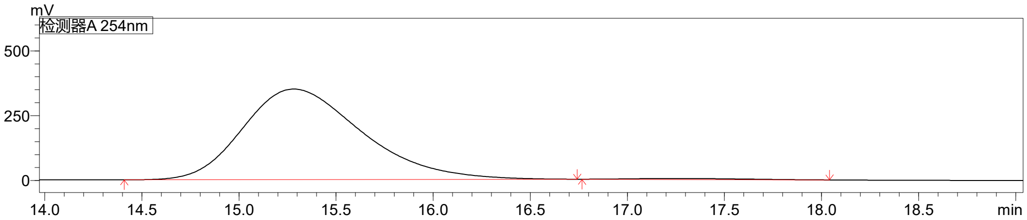

Peak#	Ret. Time	Height	Area%
1	15.282	349127	98.960
2	17.258	3867	1.040

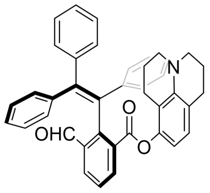

4f

HPLC conditions: Chiralpak IA 20% ^{i}PrOH/Hx eluent, 1.0 mL/min, 254 nm

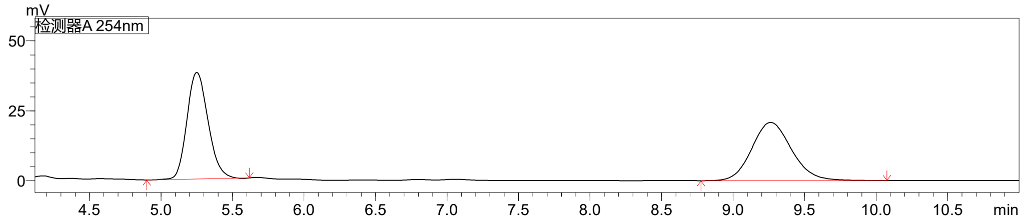

Peak#	Ret. Time	Height	Area%
1	5.250	38119	49.286
2	9.262	20791	50.714

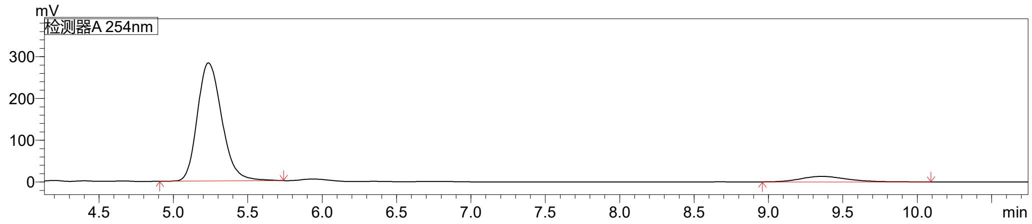

Peak#	Ret. Time	Height	Area%
1	5.235	282927	91.885
2	9.358	13183	8.115

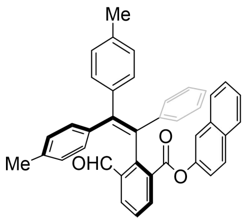

5a

HPLC conditions: Chiralpak IA 20% ^{i}PrOH/Hx eluent, 0.8 mL/min, 254 nm

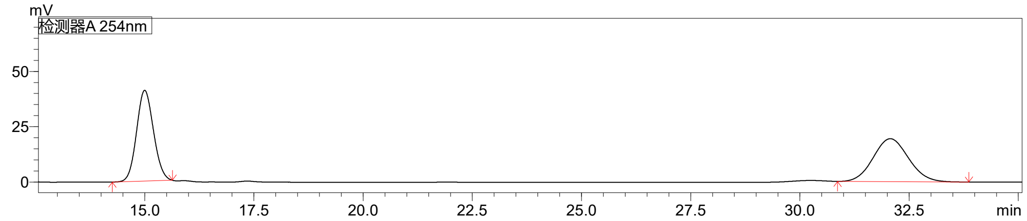

Peak#	Ret. Time	Height	Area%
1	14.998	41019	50.208
2	32.068	19420	49.792

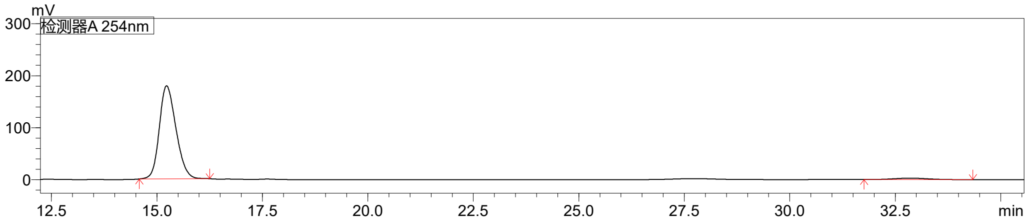

Peak#	Ret. Time	Height	Area%
1	15.228	179168	96.959
2	32.850	2783	3.041

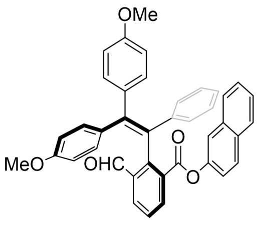

5b

HPLC conditions: Chiralpak IC 20% ^{i}PrOH/Hx eluent, 0.8 mL/min, 254 nm

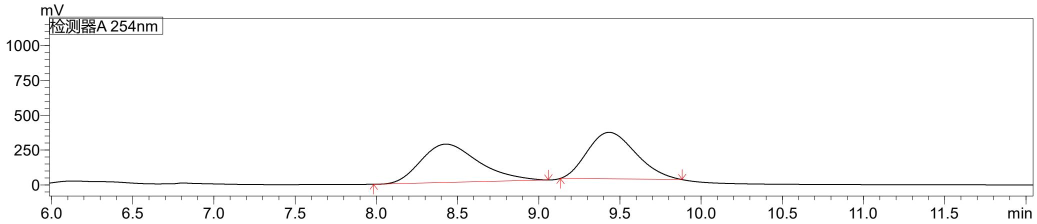

Peak#	Ret. Time	Height	Area%
1	8.429	275616	49.552
2	9.433	334151	50.448

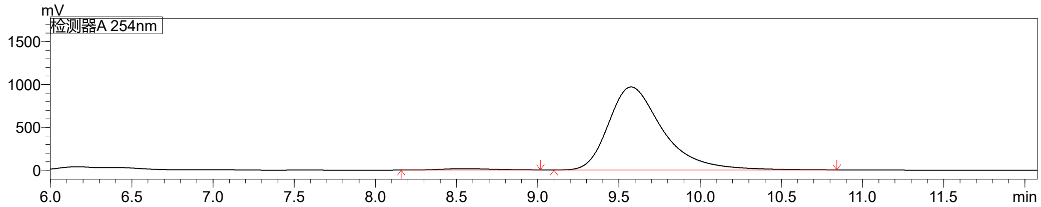

Peak#	Ret. Time	Height	Area%
1	8.546	15780	1.538
2	9.575	967534	98.462

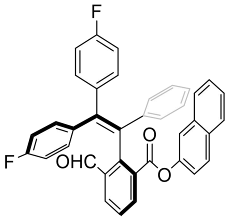

5c

HPLC conditions: Chiralpak IA 20% ^{i}PrOH/Hx eluent, 0.8 mL/min, 254 nm

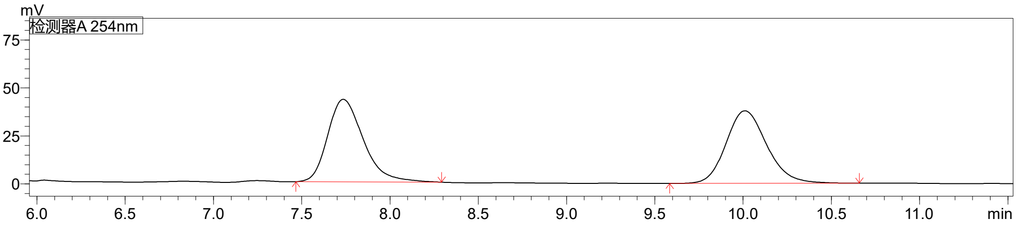

Peak#	Ret. Time	Height	Area%
1	7.735	43048	49.862
2	10.010	37840	50.138

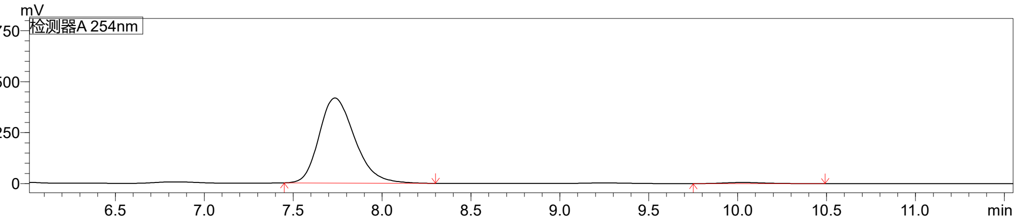

Peak#	Ret. Time	Height	Area%
1	7.734	47936	98.494
2	10.026	5678	1.506

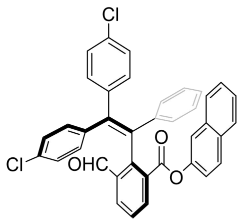

5d

HPLC conditions: Chiralpak IA 20% ^{i}PrOH/Hx eluent, 1.0 mL/min, 254 nm

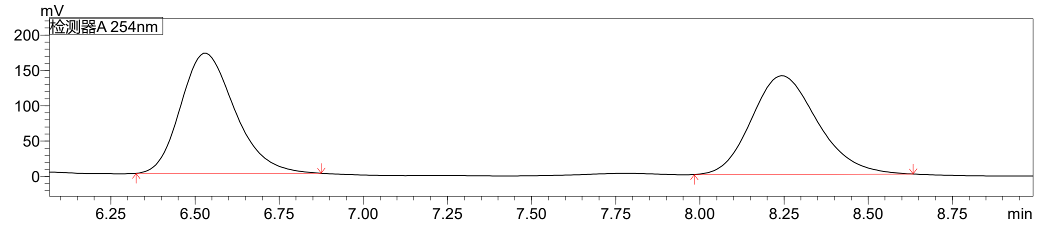

Peak#	Ret. Time	Height	Area%
1	6.530	169709	49.901
2	8.243	139465	50.099

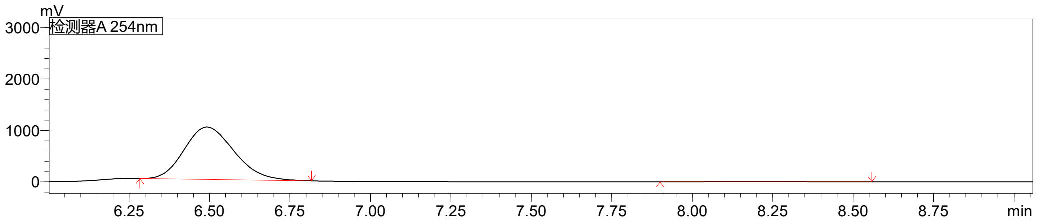

Peak#	Ret. Time	Height	Area%
1	6.492	1019907	98.531
2	8.191	11717	1.469

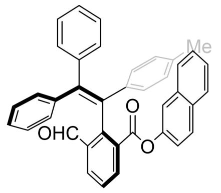

5e

HPLC conditions: Chiralpak OD 3% ^{i}PrOH/Hx eluent, 0.8 mL/min, 254 nm

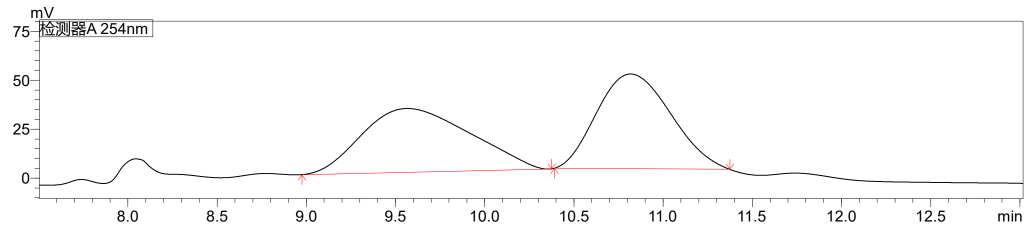

Peak#	Ret. Time	Height	Area%
1	9.567	32563	49.535
2	10.818	48386	50.465

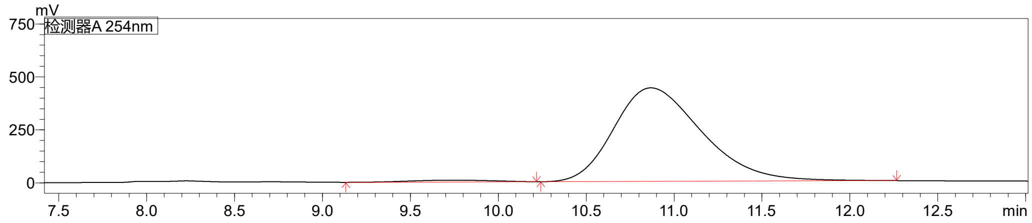

Peak#	Ret. Time	Height	Area%
1	9.743	8632	1.776
2	10.868	441461	98.224

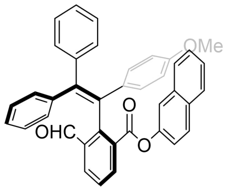

5f

HPLC conditions: Chiralpak OD 5% ^{i}PrOH/Hx eluent, 0.8 mL/min, 254 nm

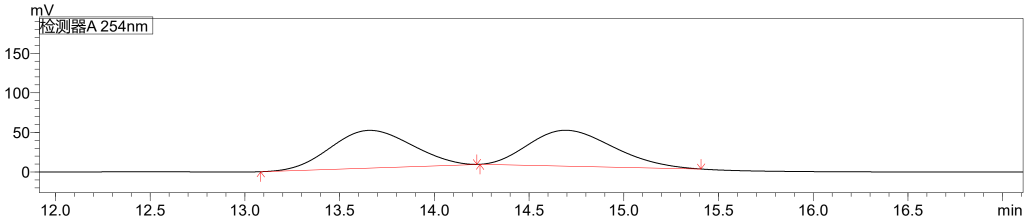

Peak#	Ret. Time	Height	Area%
1	13.660	47555	50.103
2	14.693	45278	49.897

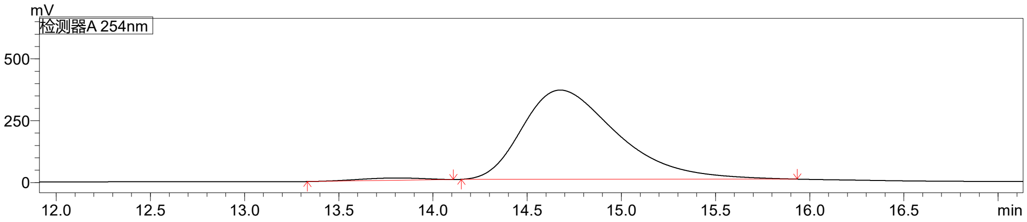

Peak#	Ret. Time	Height	Area%
1	13.810	9388	1.742
2	14.676	360748	98.258

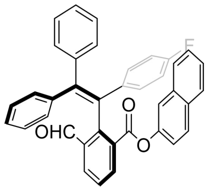

5g

HPLC conditions: Chiralpak OD 20% ^{i}PrOH/Hx eluent, 0.8 mL/min, 254 nm

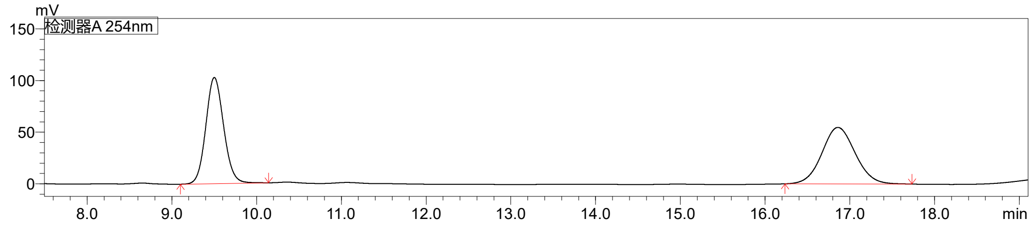

Peak#	Ret. Time	Height	Area%
1	9.501	102722	50.087
2	16.858	54722	49.913

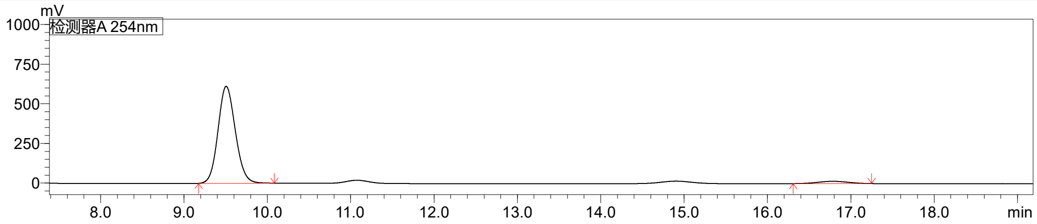

Peak#	Ret. Time	Height	Area%
1	9.506	611801	96.040
2	16.785	14429	3.960

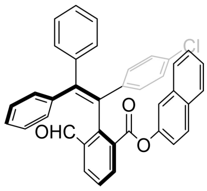

5h

HPLC conditions: Chiralpak IA 20% ^{i}PrOH/Hx eluent, 0.8 mL/min, 254 nm

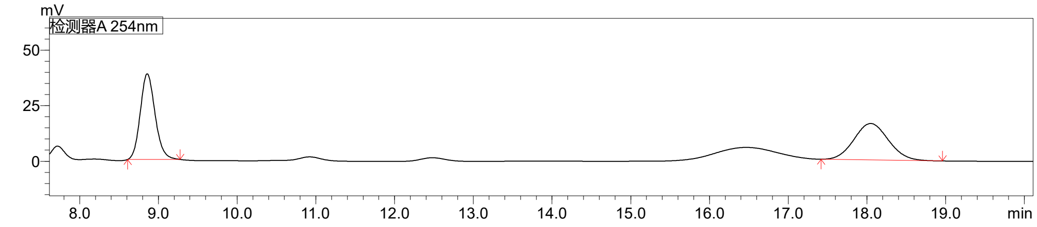

Peak#	Ret. Time	Height	Area%
1	8.858	38528	50.132
2	18.046	16406	49.868

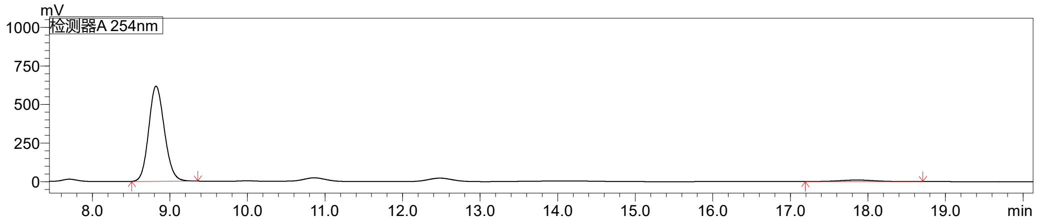

Peak#	Ret. Time	Height	Area%
1	8.821	616739	96.598
2	17.860	9685	3.402

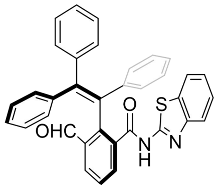

7

HPLC conditions: Chiralpak AD 20% ^{i}PrOH/Hx eluent, 0.8 mL/min, 254 nm

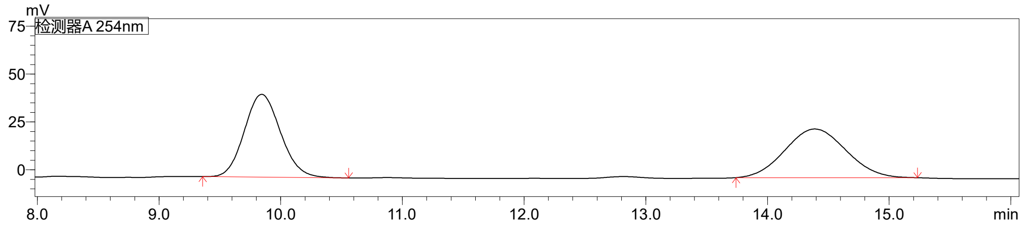

Peak#	Ret. Time	Height	Area%
1	9.843	43262	50.223
2	14.389	25414	49.777

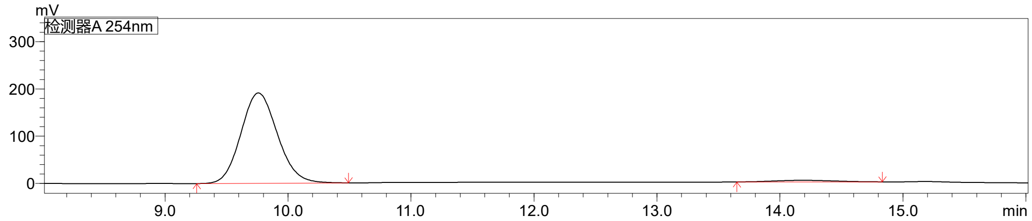

Peak#	Ret. Time	Height	Area%
1	9.758	191159	97.121
2	14.188	3418	2.879

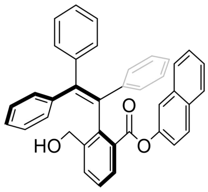

9

HPLC conditions: Chiralpak IA 20% ^{i}PrOH/Hx eluent, 0.8 mL/min, 254 nm

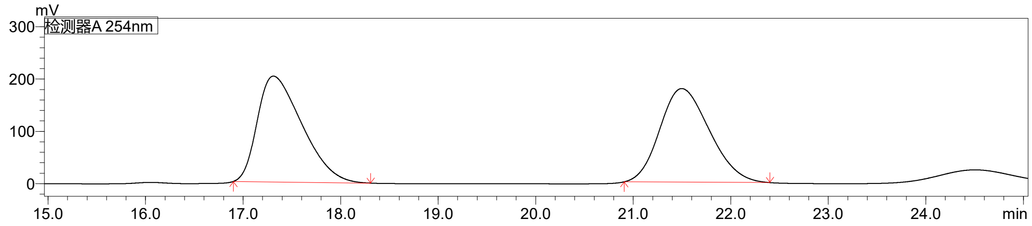

Peak#	Ret. Time	Height	Area%
1	17.311	202423	50.396
2	21.497	178956	49.604

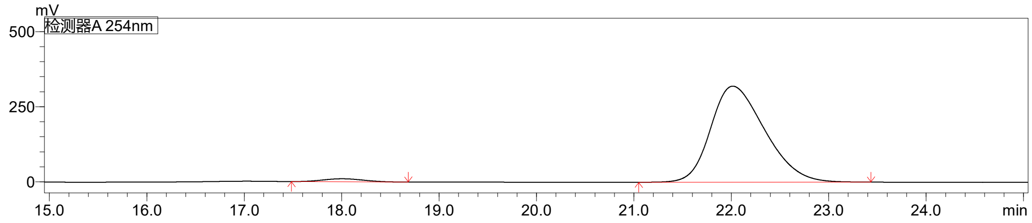

Peak#	Ret. Time	Height	Area%
1	18.004	10113	2.330
2	22.015	319623	97.670

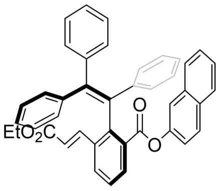

10

HPLC conditions: Chiralpak AD 20% ^{i}PrOH/Hx eluent, 0.8 mL/min, 254 nm

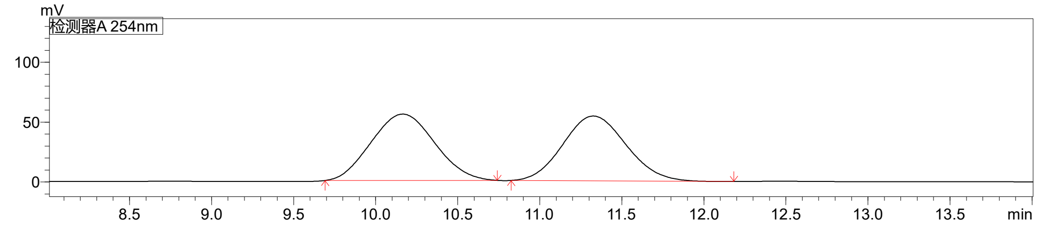

Peak#	Ret. Time	Height	Area%
1	10.164	55462	50.745
2	11.326	54181	49.255

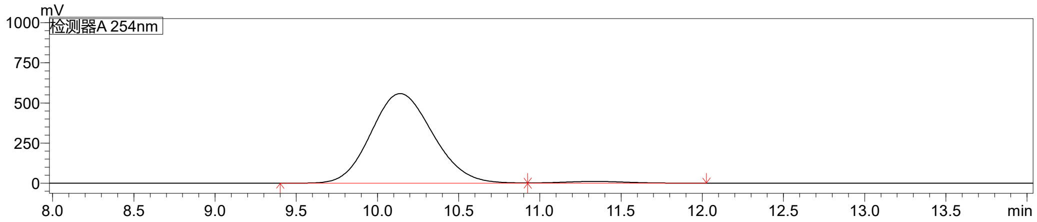

Peak#	Ret. Time	Height	Area%
1	10.139	558610	98.045
2	11.335	10955	1.955

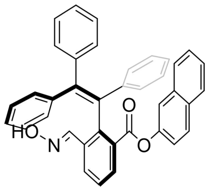

11

HPLC conditions: Chiralpak AS 20% ^{i}PrOH/Hx eluent, 0.8 mL/min, 254 nm

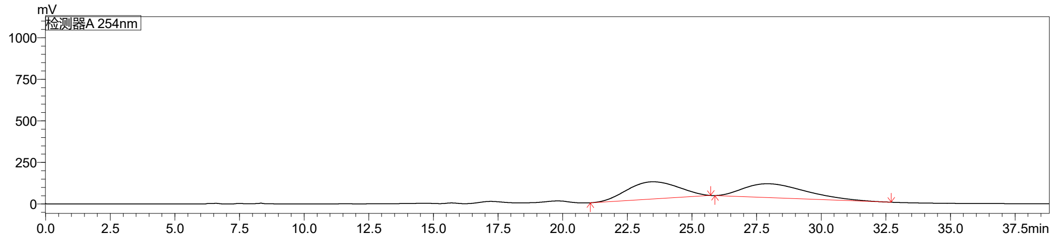

Peak#	Ret. Time	Height	Area%
1	23.505	103348	50.311
2	27.924	83062	49.689

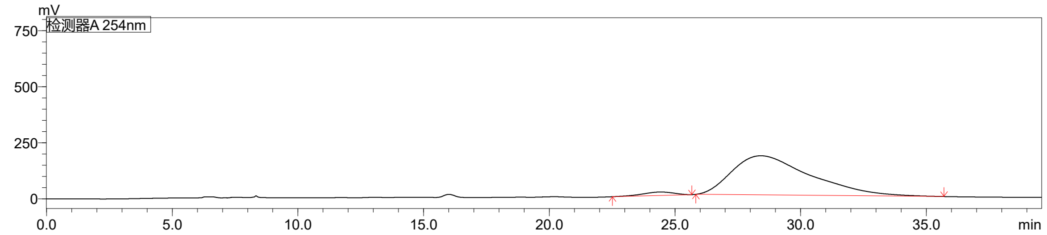

Peak#	Ret. Time	Height	Area%
1	24.431	15270	3.335
2	28.415	174719	96.665

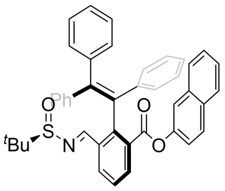

12

HPLC conditions: Chiralpak IA 20% ^{i}PrOH/Hx eluent, 0.8 mL/min, 254 nm

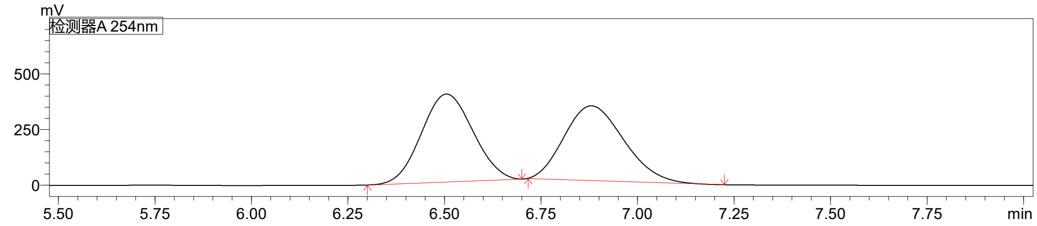

Peak#	Ret. Time	Height	Area%
1	6.505	395434	50.445
2	6.880	336081	49.555

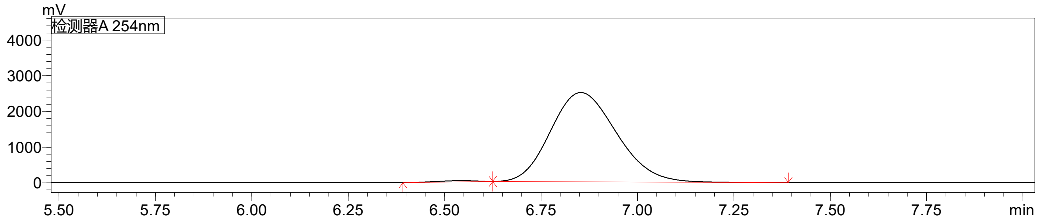

Peak#	Ret. Time	Height	Area%
1	6.543	32052	0.766
2	6.853	2501487	99.234

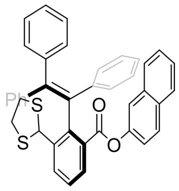

13

HPLC conditions: Chiralpak IA 30% ^{i}PrOH/Hx eluent, 0.8 mL/min, 254 nm

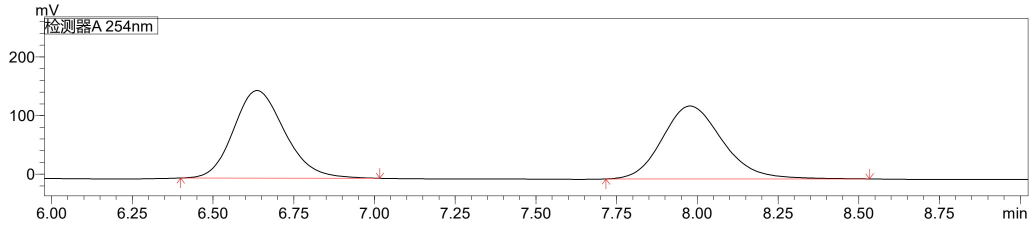

Peak#	Ret. Time	Height	Area%
1	6.636	149445	50.521
2	7.977	124474	49.479

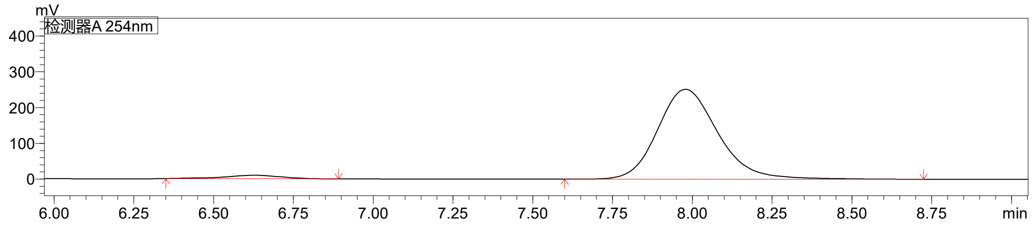

Peak#	Ret. Time	Height	Area%
1	6.627	9644	3.518
2	7.978	251444	96.482

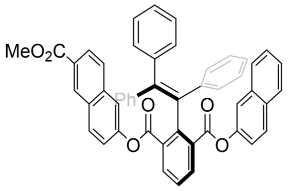

14

HPLC conditions: Chiralpak OD 10% ^{i}PrOH/Hx eluent, 0.8 mL/min, 254 nm

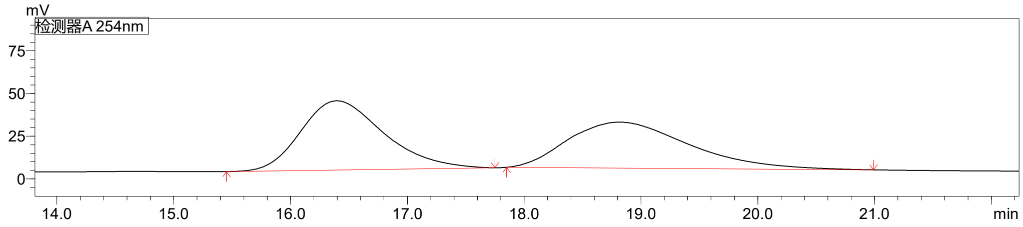

Peak#	Ret. Time	Height	Area%
1	16.397	40528	50.845
2	18.815	26895	49.155

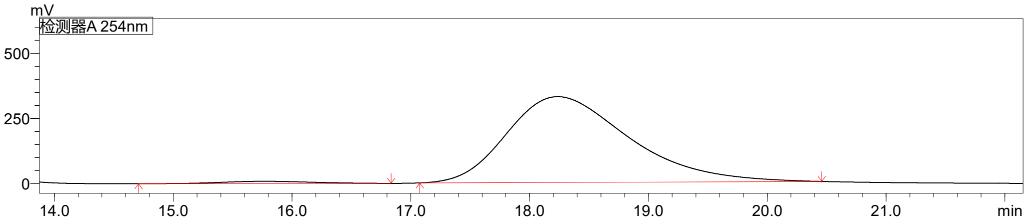

Peak#	Ret. Time	Height	Area%
1	15.766	8395	1.715
2	18.237	333067	98.285

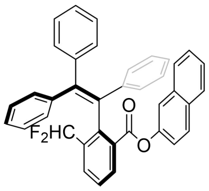

15

HPLC conditions: Chiralpak IA 10% ^{i}PrOH/Hx eluent, 0.8 mL/min, 254 nm

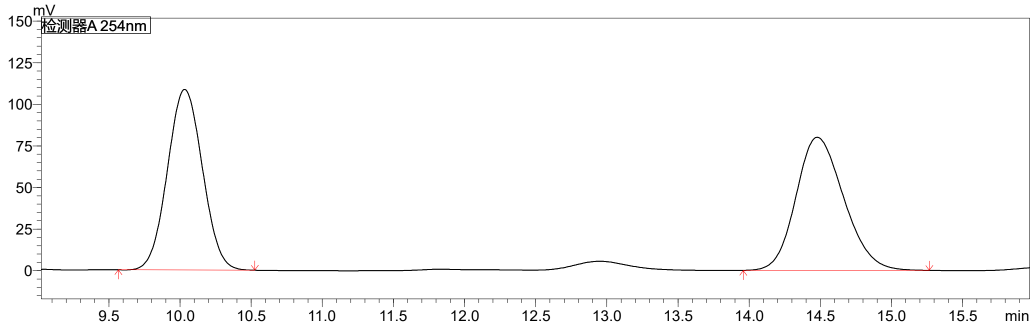

Peak#	Ret. Time	Height	Area%
1	10.032	108545	49.867
2	14.479	80005	50.133

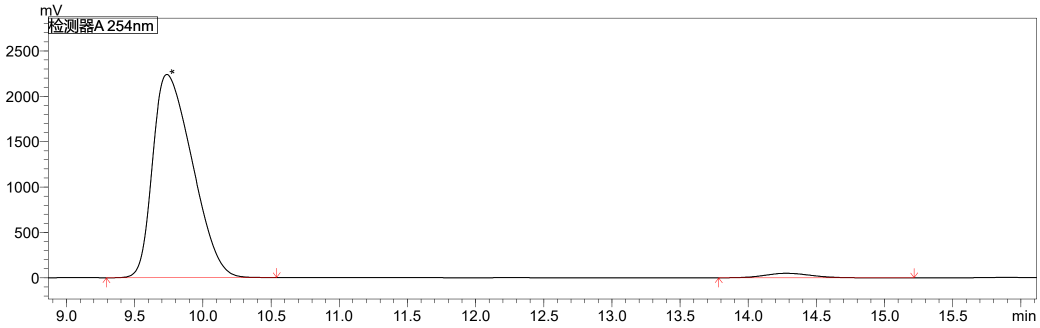

Peak#	Ret. Time	Height	Area%
1	9.736	2239320	97.534
2	14.280	49099	2.466

9. Crystallographic Data

3v

Table S3 Crystal data and structure refinement for 3v.

Identification code	3v
Empirical formula	$C_{38}H_{25}BrO_3$
Formula weight	609.49
Temperature/K	100.00(10)
Crystal system	orthorhombic
Space group	$P2_12_12_1$
a/Å	12.2854(2)
b/Å	13.6262(2)
c/Å	17.0411(2)
α/°	90
β/°	90
γ/°	90
Volume/Å^3	2852.74(7)
Z	4
ρ_{calc}g/cm^3	1.419
μ/mm^{-1}	2.264
F(000)	1248.0
Crystal size/mm^3	0.13 × 0.12 × 0.11
Radiation	Cu Kα (λ = 1.54184)
2Θ range for data collection/°	8.308 to 149.718
Index ranges	$-14 \leq h \leq 15$, $-17 \leq k \leq 15$, $-21 \leq l \leq 19$
Reflections collected	16663
Independent reflections	5644 [R_{int} = 0.0251, R_{sigma} = 0.0263]
Data/restraints/parameters	5644/0/379

Goodness-of-fit on F^2	1.039
Final R indexes [I>=2σ (I)]	R_1 = 0.0237, wR_2 = 0.0611
Final R indexes [all data]	R_1 = 0.0243, wR_2 = 0.0614
Largest diff. peak/hole / e $Å^{-3}$	0.31/-0.24
Flack/Hooft parameter	-0.004(5)/0.014(4)

Crystal structure determination of [3v]

Crystal Data for $C_{38}H_{25}BrO_3$ (M =609.49 g/mol): orthorhombic, space group $P2_12_12_1$ (no. 19), a = 12.2854(2) Å, b = 13.6262(2) Å, c = 17.0411(2) Å, V = 2852.74(7) $Å^3$, Z = 4, T = 100.00(10) K, μ(Cu Kα) = 2.264 mm^{-1}, $Dcalc$ = 1.419 g/cm^3, 16663 reflections measured (8.308° ≤ 2Θ ≤ 149.718°), 5644 unique (R_{int} = 0.0251, R_{sigma} = 0.0263) which were used in all calculations. The final R_1 was 0.0237 (I > 2σ(I)) and wR_2 was 0.0614 (all data).

Refinement model description

Table S4 Fractional Atomic Coordinates (×10^4) and Equivalent Isotropic Displacement Parameters ($Å^2$×10^3) for 3v. U_{eq} is defined as 1/3 of the trace of the orthogonalised U_{IJ} tensor.

Atom	*x*	*y*	*z*	U(eq)
Br1	-666.6(2)	9768.7(2)	3677.6(2)	26.76(8)
O1	7959.7(15)	1700.8(13)	3828.4(11)	27.9(4)
O2	5290.8(14)	5855.8(12)	4045.8(10)	20.8(3)
O3	3642.6(13)	5141.9(13)	3900.1(10)	22.7(3)
C1	7133.1(19)	5762.9(17)	2277.9(13)	18.0(4)
C2	7618(2)	5674.4(19)	1540.0(14)	24.6(5)
C3	8059(2)	6486(2)	1164.8(14)	28.7(5)
C4	7971(2)	7401(2)	1496.9(15)	28.5(5)
C5	7474(2)	7506(2)	2224.5(17)	31.7(6)
C6	7079(2)	6686(2)	2618.1(15)	26.6(5)
C7	6584.6(18)	4906.5(17)	2658.3(13)	18.2(4)
C8	6886.0(19)	4526.3(16)	3353.8(13)	17.9(4)

Table S4 Fractional Atomic Coordinates (×10^4) and Equivalent Isotropic Displacement Parameters (Å^2×10^3) for 3v. U_{eq} is defined as 1/3 of the trace of the orthogonalised U_{IJ} tensor.

Atom	*x*	*y*	*z*	U(eq)
C9	7941.9(17)	4777.4(16)	3736.0(13)	18.4(4)
C10	8904(2)	4795(2)	3296.5(13)	22.2(4)
C11	9901.7(19)	4971.8(19)	3650.8(16)	26.4(5)
C12	9959(2)	5153(2)	4450.0(15)	26.9(5)
C13	9015(2)	5136(2)	4895.8(13)	26.7(5)
C14	8020(2)	4939.2(18)	4542.0(13)	22.4(5)
C15	6169.0(18)	3817.9(16)	3791.1(12)	17.0(4)
C16	5137.2(19)	4096.8(17)	4078.4(13)	18.1(4)
C17	4485(2)	3437.0(18)	4489.8(14)	22.3(5)
C18	4842(2)	2489.3(18)	4642.6(14)	23.7(5)
C19	5862(2)	2204.4(17)	4377.8(13)	21.4(5)
C20	6510.2(19)	2851.3(17)	3949.4(13)	18.5(4)
C21	7564.2(19)	2484.4(16)	3644.8(15)	21.8(4)
C22	4747.0(18)	5128.6(18)	4003.0(12)	17.9(4)
C23	3079.3(19)	6035.1(18)	3941.1(14)	20.6(5)
C24	3207(2)	6670.4(19)	4590.4(14)	22.3(5)
C25	2543(2)	7466.6(19)	4658.9(14)	22.2(5)
C26	1722.4(19)	7661.5(18)	4093.8(14)	20.3(4)
C27	1011(2)	8476.9(18)	4168.1(14)	21.6(5)
C28	240.0(19)	8636.1(18)	3607.1(16)	23.6(5)
C29	108(2)	8003(2)	2959.3(15)	26.2(5)
C30	777(2)	7210.9(19)	2879.8(14)	25.0(5)
C31	1616.7(19)	7026.3(18)	3437.4(13)	20.1(4)
C32	2329(2)	6205.8(18)	3369.1(14)	21.2(5)
C33	5615(2)	4537.4(16)	2213.1(12)	17.9(4)

Table S4 Fractional Atomic Coordinates (×10^4) and Equivalent Isotropic Displacement Parameters (Å^2×10^3) for 3v. U_{eq} is defined as 1/3 of the trace of the orthogonalised U_{IJ} tensor.

Atom	*x*	*y*	*z*	U(eq)
C34	4752.2(19)	5165.5(19)	2040.0(13)	20.7(4)
C35	3875.2(19)	4832(2)	1597.3(13)	23.6(5)
C36	3878(2)	3887.3(19)	1298.9(15)	25.6(5)
C37	4747(2)	3264.1(19)	1444.6(14)	24.7(5)
C38	5605(2)	3584.1(17)	1914.4(13)	20.4(4)

Table S5 Anisotropic Displacement Parameters (Å^2×10^3) for 3v. The Anisotropic displacement factor exponent takes the form: $-2\pi^2[h^2a^{*2}U_{11}+2hka^*b^*U_{12}+\ldots]$.

Atom	U_{11}	U_{22}	U_{33}	U_{23}	U_{13}	U_{12}
Br1	23.29(12)	24.96(13)	32.03(13)	4.2(1)	2.86(10)	8.51(10)
O1	25.4(8)	20.2(8)	38.1(10)	2.1(7)	0.3(7)	7.1(7)
O2	21.4(8)	17.0(8)	23.9(8)	-0.7(6)	3.0(6)	0.5(6)
O3	16.8(7)	17.8(7)	33.7(9)	-0.3(7)	0.3(6)	2.8(7)
C1	17.2(10)	19.0(11)	17.9(10)	2.5(8)	-2.2(8)	0.1(9)
C2	29.7(12)	22.1(11)	22.0(11)	-0.9(9)	4.4(9)	-2.7(10)
C3	33.4(13)	34.6(14)	18.1(11)	2.3(9)	3.1(10)	-6.3(11)
C4	30.9(13)	25.2(12)	29.5(13)	6.5(10)	0.5(10)	-7.0(11)
C5	38.5(16)	19.0(12)	37.7(14)	-1.6(11)	9.0(12)	-4.6(12)
C6	28.9(13)	23.7(12)	27.1(11)	-1.8(10)	8.7(10)	-3.7(10)
C7	16.7(10)	18.1(11)	19.7(9)	-2.4(8)	1.8(8)	2.9(9)
C8	18.9(10)	15.5(10)	19.4(9)	-2.2(8)	2.0(8)	1.4(8)
C9	19.4(10)	14.8(9)	21.0(10)	1.2(10)	-2.1(8)	1.9(8)
C10	20.6(10)	26.2(11)	19.9(10)	0.5(10)	0.6(8)	-2.6(10)
C11	19.7(10)	30.6(13)	28.8(11)	2.5(10)	0.8(10)	-2.8(9)
C12	23.9(11)	27.1(12)	29.7(12)	1.1(10)	-7.9(9)	-4.0(10)

Table S5 Anisotropic Displacement Parameters (Å^2×10^3) for 3v. The Anisotropic displacement factor exponent takes the form: -2π^2[h^{2}a*^{2}U$_{11}$+2hka*b*U$_{12}$+…].

Atom	U_{11}	U_{22}	U_{33}	U_{23}	U_{13}	U_{12}
C13	30.5(12)	30.0(13)	19.7(10)	-1.7(10)	-5.0(9)	-1.9(11)
C14	22.8(11)	23.8(12)	20.5(10)	-0.9(9)	1.2(8)	1.0(9)
C15	18.9(10)	17.5(10)	14.6(9)	0.3(8)	-1.2(8)	1.4(8)
C16	18.4(11)	15.7(11)	20.2(10)	0.1(8)	-1.1(9)	1.1(9)
C17	20.7(12)	20.1(11)	26.1(10)	-0.3(9)	5.4(9)	-0.9(9)
C18	26.9(12)	17.1(11)	27.2(11)	3.2(9)	5.2(10)	-2.3(10)
C19	27.3(13)	15.6(10)	21.3(10)	-0.4(8)	0.1(9)	0.9(9)
C20	20.9(10)	17.8(10)	16.8(9)	-1.0(8)	-1.3(8)	2.0(9)
C21	22.3(11)	17.6(10)	25.5(10)	-0.4(10)	0.1(10)	0.5(9)
C22	18.2(10)	17.6(11)	17.9(9)	-0.3(8)	2.8(8)	1.2(9)
C23	17.8(11)	17.2(11)	26.7(11)	1.9(8)	3.1(9)	1.4(9)
C24	21.3(11)	23.2(11)	22.4(11)	3.3(9)	0.9(9)	2.8(9)
C25	21.7(12)	24.1(11)	20.8(10)	0.4(9)	1.3(9)	0.4(10)
C26	18.6(10)	20.7(11)	21.6(10)	0.7(9)	4.1(9)	0.0(9)
C27	22.3(11)	20.4(11)	22.2(10)	0.9(9)	4.7(9)	1.3(9)
C28	18.8(10)	22.0(11)	30.2(12)	4.9(10)	4.2(10)	3.8(9)
C29	22.4(11)	30.2(13)	25.9(12)	3.8(10)	-3.5(10)	0.3(10)
C30	23.1(12)	25.6(12)	26.4(11)	-0.6(9)	0.3(10)	-0.4(10)
C31	18.9(10)	21.0(11)	20.5(10)	2.6(8)	2.3(8)	-2.6(9)
C32	19.4(11)	19.4(11)	24.7(10)	-2.4(9)	3.4(9)	-2.4(9)
C33	17.7(10)	20.7(11)	15.3(9)	0.0(7)	1.0(8)	-2.0(9)
C34	22.5(10)	17.4(10)	22.2(10)	2.6(9)	0.5(8)	0.0(10)
C35	19.1(10)	27.8(12)	23.9(10)	4.7(10)	-1.8(8)	-0.1(10)
C36	22.4(11)	32.1(12)	22.2(10)	0.2(11)	-1.4(10)	-8.6(10)
C37	27.0(11)	20.8(11)	26.3(12)	-5.0(9)	2.3(10)	-5.8(9)

Table S5 Anisotropic Displacement Parameters (Å^2×10^3) for 3v. The Anisotropic displacement factor exponent takes the form: -2π^2[h^{2}a*^{2}U$_{11}$+2hka*b*U$_{12}$+…].

Atom	U_{11}	U_{22}	U_{33}	U_{23}	U_{13}	U_{12}
C38	20.3(10)	19.1(11)	21.9(10)	-1.6(8)	3.0(9)	0.6(9)

Table S6 Bond Lengths for 3v.

Atom	Atom	Length/Å	Atom	Atom	Length/Å
Br1	C28	1.907(2)	C16	C17	1.394(3)
O1	C21	1.214(3)	C16	C22	1.491(3)
O2	C22	1.197(3)	C17	C18	1.389(4)
O3	C22	1.368(3)	C18	C19	1.387(4)
O3	C23	1.402(3)	C19	C20	1.395(3)
C1	C2	1.397(3)	C20	C21	1.482(3)
C1	C6	1.387(4)	C23	C24	1.414(4)
C1	C7	1.495(3)	C23	C32	1.362(4)
C2	C3	1.388(4)	C24	C25	1.363(4)
C3	C4	1.374(4)	C25	C26	1.419(3)
C4	C5	1.389(4)	C26	C27	1.419(3)
C5	C6	1.391(4)	C26	C31	1.420(3)
C7	C8	1.345(3)	C27	C28	1.363(4)
C7	C33	1.499(3)	C28	C29	1.410(4)
C8	C9	1.491(3)	C29	C30	1.364(4)
C8	C15	1.504(3)	C30	C31	1.425(4)
C9	C10	1.399(3)	C31	C32	1.424(3)
C9	C14	1.395(3)	C33	C34	1.394(3)
C10	C11	1.388(3)	C33	C38	1.395(3)
C11	C12	1.386(4)	C34	C35	1.392(3)
C12	C13	1.387(4)	C35	C36	1.384(4)

Table S5 Anisotropic Displacement Parameters (Å^2×10^3) for 3v. The Anisotropic displacement factor exponent takes the form: -2π^2[h^{2}a*^{2}U$_{11}$+2hka*b*U$_{12}$+…].

Atom	U_{11}	U_{22}	U_{33}	U_{23}	U_{13}	U_{12}
C13	C14	1.389(3)		C36	C37	1.386(4)
C15	C16	1.411(3)		C37	C38	1.394(4)
C15	C20	1.408(3)				

Table S7 Bond Angles for 3v.

Atom	Atom	Atom	Angle/°	Atom	Atom	Atom	Angle/°
C22	O3	C23	119.6(2)	C19	C20	C15	121.4(2)
C2	C1	C7	121.0(2)	C19	C20	C21	118.0(2)
C6	C1	C2	118.4(2)	O1	C21	C20	123.8(2)
C6	C1	C7	120.3(2)	O2	C22	O3	123.4(2)
C3	C2	C1	120.9(2)	O2	C22	C16	126.6(2)
C4	C3	C2	120.2(2)	O3	C22	C16	110.0(2)
C3	C4	C5	119.8(2)	O3	C23	C24	121.0(2)
C4	C5	C6	120.0(2)	C32	C23	O3	116.5(2)
C1	C6	C5	120.7(2)	C32	C23	C24	122.1(2)
C1	C7	C33	113.61(19)	C25	C24	C23	119.2(2)
C8	C7	C1	123.9(2)	C24	C25	C26	121.1(2)
C8	C7	C33	122.4(2)	C25	C26	C27	121.6(2)
C7	C8	C9	122.4(2)	C25	C26	C31	119.0(2)
C7	C8	C15	121.5(2)	C27	C26	C31	119.4(2)
C9	C8	C15	116.12(19)	C28	C27	C26	119.3(2)
C10	C9	C8	120.3(2)	C27	C28	Br1	119.37(19)
C14	C9	C8	121.7(2)	C27	C28	C29	122.1(2)
C14	C9	C10	117.8(2)	C29	C28	Br1	118.50(19)
C11	C10	C9	121.1(2)	C30	C29	C28	119.5(2)

Table S7 Bond Angles for 3v.

Atom	Atom	Atom	Angle/°	Atom	Atom	Atom	Angle/°
C12	C11	C10	120.2(2)	C29	C30	C31	120.7(2)
C11	C12	C13	119.5(2)	C26	C31	C30	118.9(2)
C12	C13	C14	120.1(2)	C26	C31	C32	119.1(2)
C13	C14	C9	121.2(2)	C32	C31	C30	121.9(2)
C16	C15	C8	121.7(2)	C23	C32	C31	119.4(2)
C20	C15	C8	121.4(2)	C34	C33	C7	120.3(2)
C20	C15	C16	116.9(2)	C34	C33	C38	119.2(2)
C15	C16	C22	120.9(2)	C38	C33	C7	120.3(2)
C17	C16	C15	121.2(2)	C35	C34	C33	120.2(2)
C17	C16	C22	117.8(2)	C36	C35	C34	120.1(2)
C18	C17	C16	120.8(2)	C35	C36	C37	120.4(2)
C19	C18	C17	119.0(2)	C36	C37	C38	119.6(2)
C18	C19	C20	120.6(2)	C37	C38	C33	120.5(2)
C15	C20	C21	120.6(2)				

Table S8 Torsion Angles for 3v.

A	B	C	D	Angle/°	A	B	C	D	Angle/°
Br1	C28	C29	C30	177.31(19)	C15	C16	C22	O3	146.74(19)
O3	C23	C24	C25	-171.5(2)	C15	C20	C21	O1	171.7(2)
O3	C23	C32	C31	170.8(2)	C16	C15	C20	C19	-0.7(3)
C1	C2	C3	C4	-3.6(4)	C16	C15	C20	C21	177.7(2)
C1	C7	C8	C9	-13.6(3)	C16	C17	C18	C19	-0.2(4)
C1	C7	C8	C15	166.4(2)	C17	C16	C22	O2	140.3(2)
C1	C7	C33	C34	-57.0(3)	C17	C16	C22	O3	-37.7(3)
C1	C7	C33	C38	117.9(2)	C17	C18	C19	C20	-1.4(4)
C2	C1	C6	C5	1.6(4)	C18	C19	C20	C15	1.8(3)

Table S8 Torsion Angles for 3v.

A	B	C	D	Angle/°	A	B	C	D	Angle/°
C2	C1	C7	C8	119.9(3)	C18	C19	C20	C21	-176.6(2)
C2	C1	C7	C33	-63.4(3)	C19	C20	C21	O1	-9.8(4)
C2	C3	C4	C5	2.5(4)	C20	C15	C16	C17	-0.9(3)
C3	C4	C5	C6	0.7(4)	C20	C15	C16	C22	174.5(2)
C4	C5	C6	C1	-2.7(4)	C22	O3	C23	C24	-52.1(3)
C6	C1	C2	C3	1.6(4)	C22	O3	C23	C32	134.4(2)
C6	C1	C7	C8	-66.3(3)	C22	C16	C17	C18	-174.2(2)
C6	C1	C7	C33	110.4(3)	C23	O3	C22	O2	-8.2(3)
C7	C1	C2	C3	175.5(2)	C23	O3	C22	C16	169.85(18)
C7	C1	C6	C5	-172.3(2)	C23	C24	C25	C26	0.5(4)
C7	C8	C9	C10	-44.9(3)	C24	C23	C32	C31	-2.6(4)
C7	C8	C9	C14	139.2(2)	C24	C25	C26	C27	178.7(2)
C7	C8	C15	C16	-63.9(3)	C24	C25	C26	C31	-1.6(3)
C7	C8	C15	C20	117.7(2)	C25	C26	C27	C28	179.6(2)
C7	C33	C34	C35	177.2(2)	C25	C26	C31	C30	178.6(2)
C7	C33	C38	C37	-174.6(2)	C25	C26	C31	C32	0.7(3)
C8	C7	C33	C34	119.7(2)	C26	C27	C28	Br1	-176.79(18)
C8	C7	C33	C38	-65.3(3)	C26	C27	C28	C29	1.4(4)
C8	C9	C10	C11	-176.3(2)	C26	C31	C32	C23	1.4(3)
C8	C9	C14	C13	177.5(2)	C27	C26	C31	C30	-1.6(3)
C8	C15	C16	C17	-179.3(2)	C27	C26	C31	C32	-179.6(2)
C8	C15	C16	C22	-4.0(3)	C27	C28	C29	C30	-0.9(4)
C8	C15	C20	C19	177.8(2)	C28	C29	C30	C31	-0.9(4)
C8	C15	C20	C21	-3.8(3)	C29	C30	C31	C26	2.2(4)
C9	C8	C15	C16	116.2(2)	C29	C30	C31	C32	-179.9(2)
C9	C8	C15	C20	-62.2(3)	C30	C31	C32	C23	-176.5(2)

Table S8 Torsion Angles for 3v.

A	B	C	D	Angle/°	A	B	C	D	Angle/°
C9	C10	C11	C12	-1.2(4)	C31	C26	C27	C28	-0.1(3)
C10	C9	C14	C13	1.5(4)	C32	C23	C24	C25	1.7(4)
C10	C11	C12	C13	1.4(4)	C33	C7	C8	C9	170.0(2)
C11	C12	C13	C14	-0.1(4)	C33	C7	C8	C15	-10.0(3)
C12	C13	C14	C9	-1.4(4)	C33	C34	C35	C36	-2.8(3)
C14	C9	C10	C11	-0.2(4)	C34	C33	C38	C37	0.4(3)
C15	C8	C9	C10	135.1(2)	C34	C35	C36	C37	0.7(4)
C15	C8	C9	C14	-40.8(3)	C35	C36	C37	C38	1.9(4)
C15	C16	C17	C18	1.3(4)	C36	C37	C38	C33	-2.4(4)
C15	C16	C22	O2	-35.3(3)	C38	C33	C34	C35	2.2(3)

Table S9 Hydrogen Atom Coordinates (Å×10^4) and Isotropic Displacement Parameters (Å^2×10^3) for 3v.

Atom	*x*	*y*	*z*	U(eq)
H2	7645.88	5050.63	1291.79	30
H3	8423.82	6408.31	676.97	34
H4	8248.27	7959.57	1230.05	34
H5	7404.32	8138.78	2453.3	38
H6	6768.85	6759.13	3125.42	32
H10	8873.12	4684.7	2746.55	27
H11	10547.79	4968.55	3344.46	32
H12	10640.34	5287.79	4690.74	32
H13	9048.63	5258.44	5444.06	32
H14	7380.32	4914.61	4855	27
H17	3786.62	3638.1	4667.84	27
H18	4395.08	2042.79	4924.33	28

Table S9 Hydrogen Atom Coordinates (Å×10^4) and Isotropic Displacement Parameters (Å^2×10^3) for 3v.

Atom	*x*	*y*	*z*	U(eq)
H19	6120.69	1562.42	4489.34	26
H21	7952.87	2885.48	3285.09	26
H24	3750.11	6543.83	4974.33	27
H25	2630.38	7898.26	5092.06	27
H27	1071.62	8907.4	4604.35	26
H29	-443.27	8127.56	2581.16	31
H30	682.88	6777.77	2448.24	30
H32	2280.75	5781.31	2928.28	25
H34	4762.83	5823.17	2224.72	25
H35	3273.84	5252.58	1499.99	28
H36	3281.37	3664.68	992.3	31
H37	4757	2622.92	1225.79	30
H38	6188.01	3149.92	2031.97	25

10. Reference

[1] W. Bao, S.-Q. Qiu, S.-H. Wang, H.-H. Liu, S.-H. Xiang, B. Tan, *Angew. Chem. Int. Ed.* **2025**, *64*, e202519340.

[2] R. Pan, H. Shen, Y. Wu, Y. Wang, X. Lin, *Org. Lett.* **2026**, *28*, 10.1021/acs.orglett.5c04553.
